# Supplementary material for: Targeting USP11 regulation by a novel lithium-organic coordination compound improves neuropathologies and cognitive functions in Alzheimer transgenic mice
Source: EMBO Mol Med. 2024 Oct 11;16(11):2856–81. doi: 10.1038/s44321-024-00146-7 (PMC11555261; doi:10.1038/s44321-024-00146-7)
Supplement: Supplementary file 1 — Appendix [file 44321_2024_146_MOESM1_ESM.docx]

**[**Research Article (EMM-2024-20081-V2)]

**Targeting USP11 regulation by a novel lithium-organic coordination compound improves neuropathologies and cognitive functions in Alzheimer transgenic mice**

*Yi Guo^1^, Chuanbin Cai^1^, Bingjie Zhang^2^, Bo Tan^3^, Qinmin Tang^1^, Zhifeng Lei^1^, Xiaolan Qi^1^, Jiang Chen^1,4^, Xiaojiang Zheng^2^, Dan Zi^5^, Song Li^6^***, Jun Tan^1,2,7^*^*^

^1^Key Laboratory of Endemic and Ethnic Diseases, Ministry of Education; Key Laboratory of Molecular Biology, Guizhou Medical University, Guiyang 550025, Guizhou, China;

^2^Anyu Biotechnology (Hangzhou) Co., Ltd.; Hangzhou, 310000, Zhejiang, China;

^3^Department of Biomedical Sciences, City University of Hong Kong, Hong Kong SAR, China;

^4^Department of Pharmacy, School of Medicine, Zhejiang University, Hangzhou 310058, Zhejiang, China;

^5^Department of Gynecology, Guizhou Provincial People's Hospital, Guiyang 550025, Guizhou, China;

^6^First Affiliated Hospital of Dalian Medical University, Dalian 116021, Liaoning, China;

^7^Institute of Translational Medicine; Key Laboratory of Novel Targets and Drug Study for Neural Repair of Zhejiang Province, School of Medicine, Hangzhou City University, Hangzhou 310015, Zhejiang, China.

^*^Corresponding authors.

Email address [tanjun@anyuhz.cn](mailto:tanjun@anyuhz.cn) (TJ) and [lisong@dmu.edu.cn](mailto:lisong@dmu.edu.cn) (LS)

**Appendix**

**Table of contents**

**Appendix Fig. S1………………………………………………3**

**Appendix Fig. S2………………………………………………4**

**Appendix Fig. S3………………………………………………5**

**Appendix Fig. S4………………………………………………6**

**Appendix Fig. S5………………………………………………7**

**Appendix Fig. S6………………………………………………8**

**Appendix Tab. S1………………………………………………9**

**Appendix Tab. S2………………………………………………10**

**Appendix Tab. S3………………………………………………11**

**Appendix Tab. S4………………………………………………12-13**

**Appendix Figures**

**
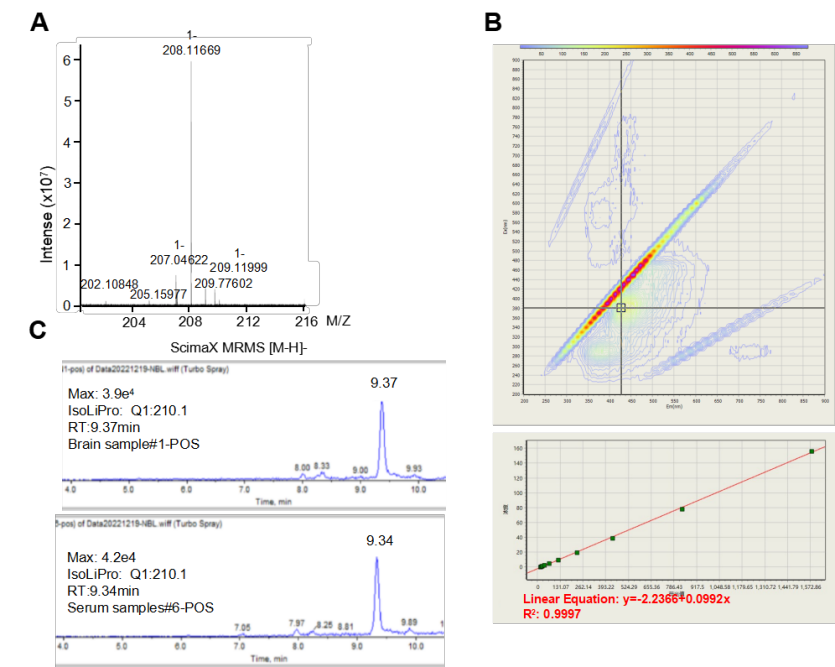
**

**Appendix Fig. S1 Characterization of IsoLiPro.** **(A)** *In vitro* scimax MRMS [M-H]- spectrometry of IsoLiPro. **(B)** Two-dimensional contour map and linear standard curve of IsoLiPro's fluorescence in aqueous solution. **(C)** Detection of IsoLiPro by metabolic MRM MS [M-H]+ spectrometry in brain & serum samples of rats.


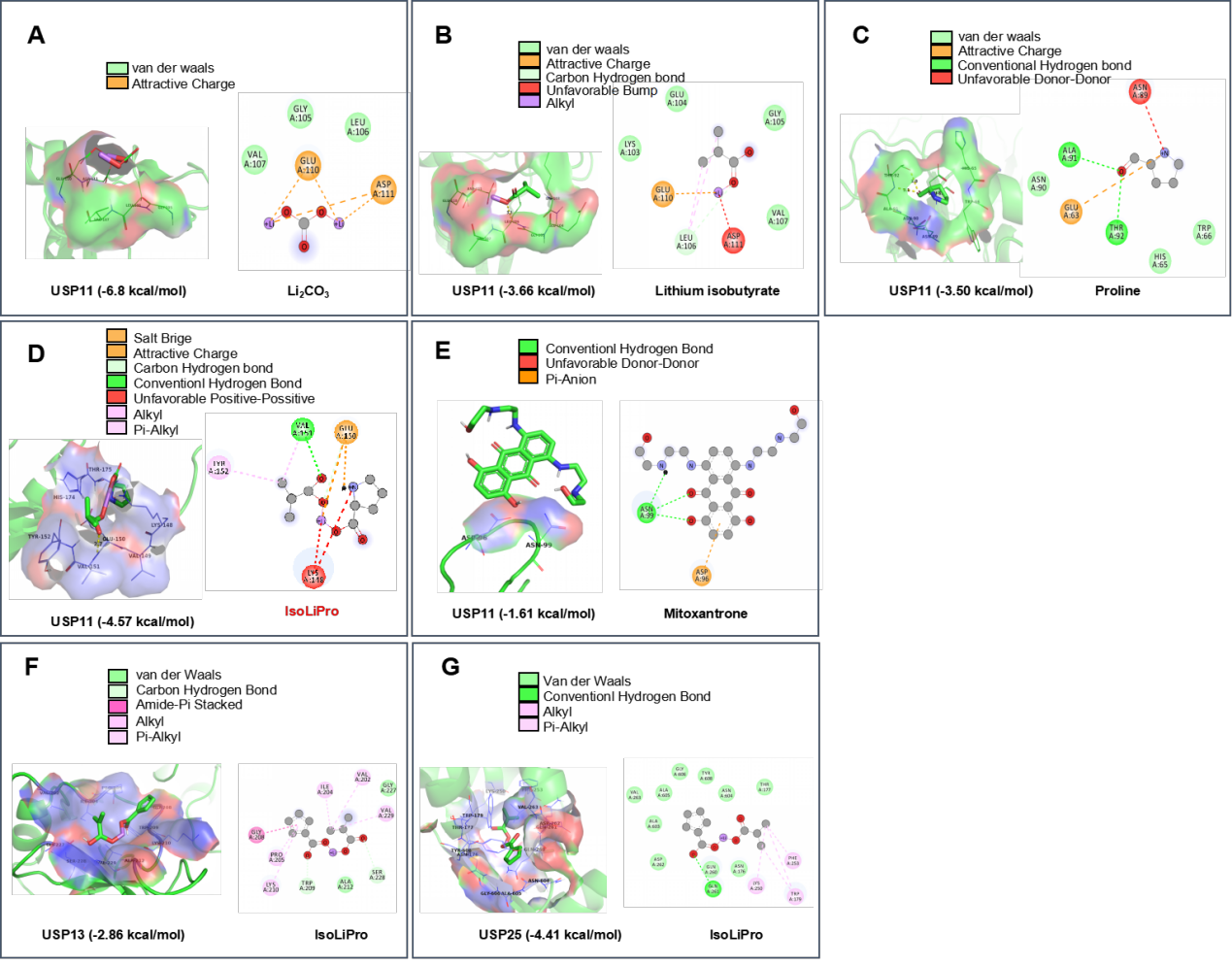


**Appendix Fig. S2 Binding affinity of IsoLiPro to USP11.** Visual depictions of how various small molecules interact with the USP11 protein, offering both 3D and 2D perspectives for detailed analysis.

**
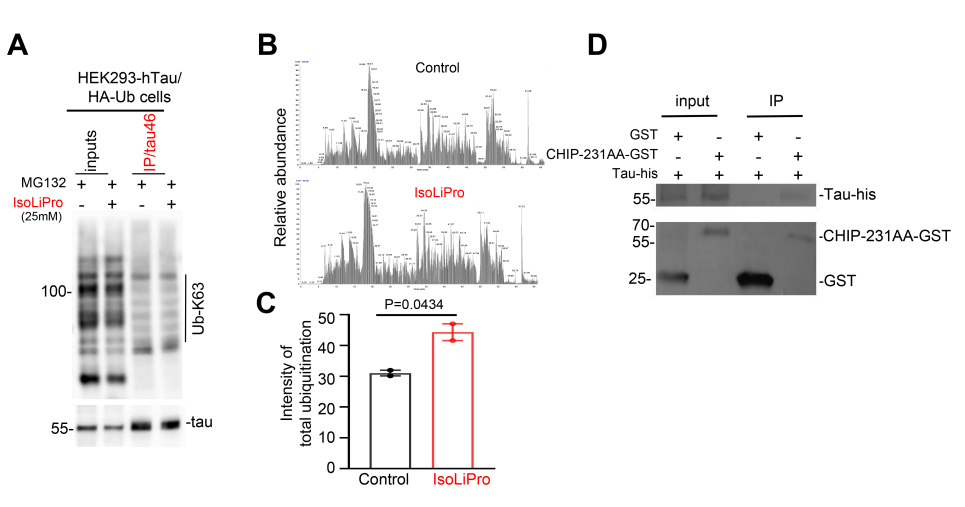
**

**Appendix Fig. S3 IsoLiPro markedly increases tau ubiquitination levels in HEK293-hTau cells. (A)** IP analysis shows no effect on Ubiquitin-K63 conjugates and tau in HEK293-hTau cells treated with 25 mM IsoLiPro and 10 µM MG132. **(B)** Total ion chromatogram displaying the profile of diGly-ub peptide signatures, as detected by LC-MS/MS. **(C)** IsoLiPro markedly intensifies the overall ubiquitination signal. **(D)** The IP experiment of CHIP-231AA-GST with Tau-his demonstrates their mutual interaction, indicating a functional interplay between these proteins. Data are represented as means ± SEM (n = 2 samples per group). *P* values were calculated using two-tailed t-test, with comparisons made against the control group.

**
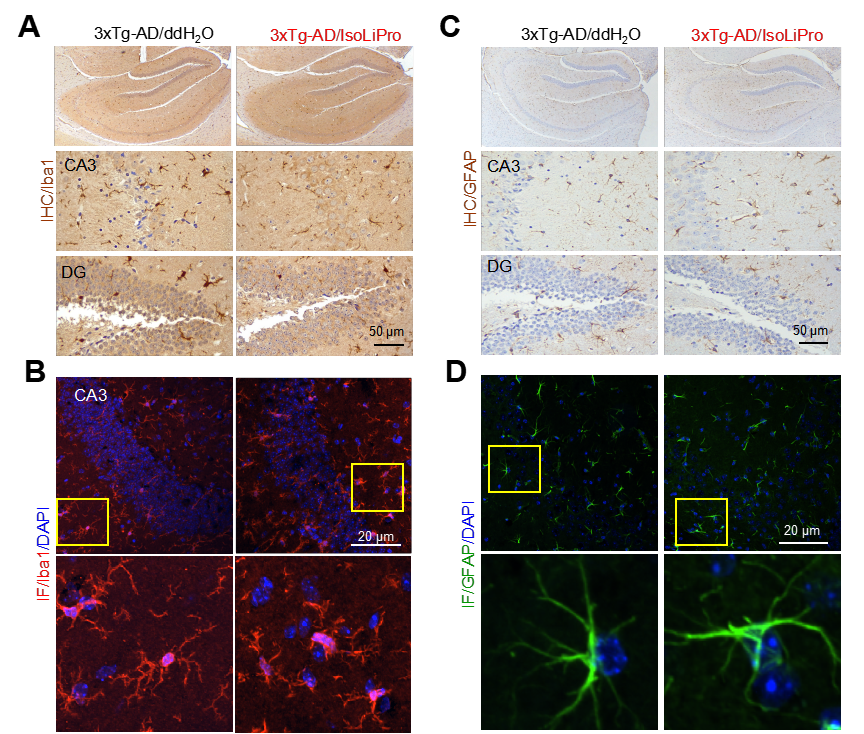
**

**Appendix Fig. S4 IsoLiPro markedly reduces gliosis in the hippocampus of 3xTg-AD mice.** Representative images of Iba1 **(A-B)**, GFAP **(C-D)** measured by IHC/IF in the hippocampus of 3xTg-AD mice following IsoLiPro treatment.


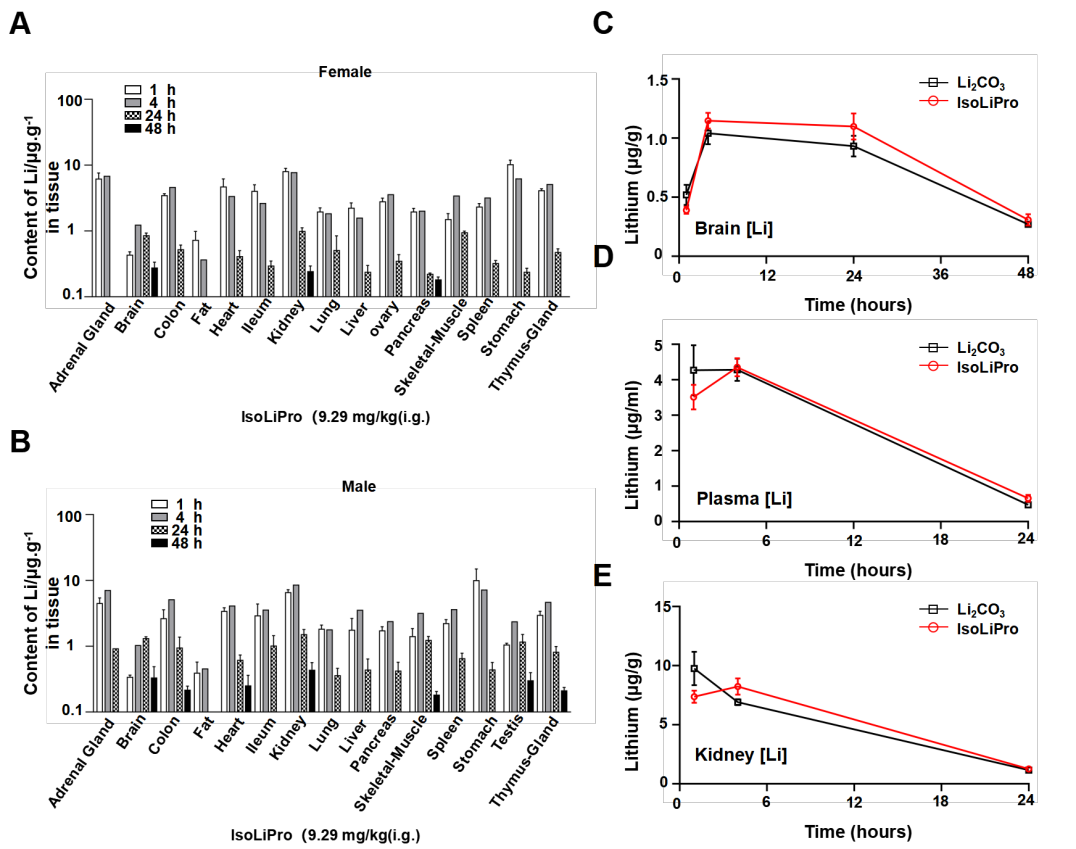


**Appendix Fig. S5 Tissue distribution and pharmacokinetics of lithium following oral treatment with IsoLiPro and Li_2_CO_3_ in SD Rat.** Tissue distribution of Li of IsoLiPro in females **(A)** and males **(B)**. Lithium concentration-time profiles of IsoLiPro and Li_2_CO_3_ in profiles brain **(C)**, plasma **(D)** and kidney **(E)**. Data are represented as means ± SEM (n=6, 3 female/3 male per group).

**
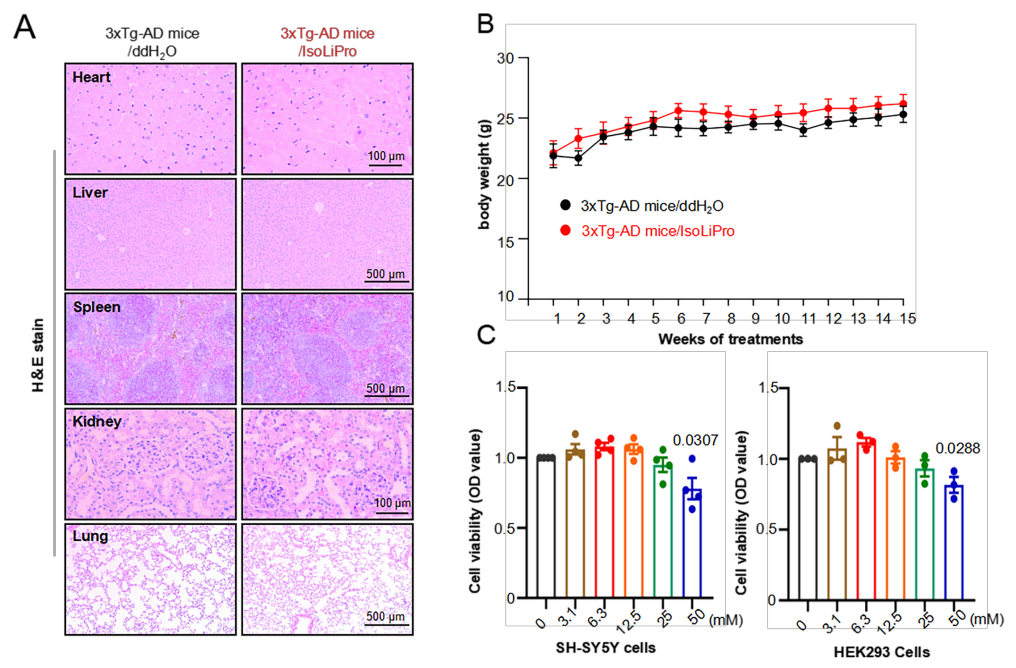
**

**Appendix Fig. S6 Toxicity evaluation of IsoLiPro. (A)** Heart, liver, spleen, lung and kidney of mice administered 560 mg/kg IsoLiPro for 16 weeks were stained by HE. **(B)** Monitoring body weights of mice during the course of treatments. Data are represented as means ± SEM (n = 8 mice per group). **(C)** SH-SY5Y and HEK293 cells viability was detected by CCK-8 assay after IsoLiPro exposure (up to 50 mM) for 24 hours. Data are represented as means ± SEM (n = 3 to 4 samples per group). *P* values were calculated using multiple t-tests, with comparisons made against the 0 mM group.

**Appendix Tab. S1 Crystallographic data and structure**

**refinement parameters**

| **Compound** | **IsoLiPro** |
| --- | --- |
| Formula | C_9_H_15_LiNO_4_ |
| unit cell | 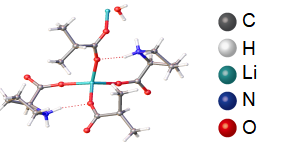 |
| *MW (g.mol-1)* | 209.17 |
| *Crystal System* | monoclinic crystals |
| *space group* | P21 |
| *a (Å)* | 10.3529 (7) |
| *b (Å)* | 10.4917 (6) |
| *c (Å)* | 10.4024 (8) |
| *alpha (° )* | 90 |
| *beta (° )* | 90.894 (5) |
| *gamma (° )* | 90 |
| *V (Å^3^)* | 1129.77 (13) |
| *Dx (Mg m-3)* | 1.256 |
| *Z* | 1 |
| *2θ range (° )* | 7.909 - 33.451 (n-butanol);  8.255 - 35.886  (ethanol and tetrahydrofuran) |
| *Nref/Npara* | 4813/337 |
| *Tmin, Tmax* | 0.593, 0.746 |
| *R1 [I > 2δ (I)]* | 0.0691 ( 4017) |
| *wR2* (reflections) | 0.1936 (4813) |

**Appendix Tab. S2 Binding free energies of the complex (kcal/mol)**

|  | **IsoLiPro** | **Li_2_CO_3_** | **LiIB** | **Pro** |
| --- | --- | --- | --- | --- |
| *Docking Score* | -4.57 | -6.8 | -3.66 | -3.5 |
| *∆Eelec* | -104.03 | -26.49 | -41.45 | -7.51 |
| *∆Evdw* | -13.3 | 0 | -0.64 | -0.05 |
| *∆EGAS (elec + vdw)* | -117.39 | -26.49 | -42.10 | -7.56 |
| *PB-SOL (Polar + np)* | 96.85 | -0.07 | 11.59 | 1.16 |
| *∆G(Total)* | -20.54 | -26.56 | -30.49 | -6.39 |

**Appendix Tab. S3 The sequences of the primers used in the study**

| **Primer Name** | **Primer Sequence 5’-3’** |
| --- | --- |
| USP11-F | GTTCCACTGCCTATCAGCCACAAG |
| USP11-R | GAAGACATCAGCCACCATCATCCTC |
| tau-F | AGAACGCCAAAGCCAAGACAGA |
| tau-R | CATTGCTGAGATGCCGTGGAGAG |
| GAPDH-F | TCAAGGCTGAGAACGGGAAG |
| GAPDH-R | CGCCCCACTTGATTTTGGAG |

**Appendix Tab. S4 Antibodies and probes used in the study**

| **Antibodies** | **Application** | **Dilution** | **Manufacturer** |
| --- | --- | --- | --- |
| Mouse-anti-tau46 | IP  WB | 1:50  1:2000 | Cell Signaling Technology  (4019) |
| Mouse-anti-tau12 | WB  IF  IHC | 1:10000  1:10000  1:10000 | Biolegend  (806501) |
| Mouse-anti-ptau181 | WB  IF  IHC | 1:1000  1:200  1:200 | Thermo Fisher Scientific  (MN1050) |
| Rabbit-anti-ptau202/205 | WB | 1:1000 | Abcam  (ab109930) |
| Mouse-anti-ptau202/205 | WB  IHC | 1:1000  1:200 | Thermo Fisher Scientific  (MN1020) |
| Rabbit-anti-ptau231 | WB | 1:5000 | Abcam  (ab151559) |
| Rabbit-anti-ptau396 | WB | 1:10000 | Abcam  (210703) |
| Mouse-anti-β-Amyloid,17-24 Antibody | IHC  IF | 1:500  1:500 | Biolegend  (800703) |
| Rabbit-anti-Iba1 | IHC  IF | 1:1000  1:1000 | Abcam  (ab178846) |
| Mouse-anti-GFAP | IF | 1:400 | Cell Signaling  Technology(3670) |
| Rabbit-anti-SYN1 | IHC | 1:500 | Abcam  （20258-1-AP） |
| Rabbit-anti-PSD95 | IHC | 1:500 | Abcam  (ab18258) |
| Rabbit-anti-Ubiquitin | WB | 1:1000 | Proteintech  (10201-2-AP) |
| Rabbit-[anti-Ubiquitin](https://www.abcam.cn/products/primary-antibodies/ubiquitin-linkage-specific-k48-antibody-ep8589-ab140601.html)  [(linkage-specific K48)](https://www.abcam.cn/products/primary-antibodies/ubiquitin-linkage-specific-k48-antibody-ep8589-ab140601.html) | WB | 1:5000 | Abcam  (ab140601) |
| Rabbit-anti-Ubiquitin  (linkage-specific K63) | WB | 1:5000 | Abcam  (ab179434) |
| Rabbit-anti-GAPDH | WB | 1:10000 | HUABIO  (EM1101) |
| Rabbit-anti-USP11 | WB | 1:10000 | Abcam  (ab109232) |
| Rabbit-anti-USP11 | IF  IHC | 1:100  1:100 | Abcam  (ab109232) |
| Rabbit-anti-USP25 | WB | 1:10000 | Abcam  (ab187156) |
| Anti-rabbit IgG | WB  IF  IHC | 1:10000  1:500  1:500 | HUABIO  (HA1001) |
| Anti-mouse IgG | WB  IF  IHC | 1:10000  1:500  1:500 | HUABIO  (HA1006) |
| Anti-mouse IgG, Alexa Fluor™ 488 | IF | 1:500 | Thermo Fisher Scientific  (A-11001) |
| Anti-rabbit IgG, Alexa Fluor™ 555 | IF | 1:500 | Thermo Fisher Scientific  (A-21428) |
