# Supplementary material for: Targeting USP11 regulation by a novel lithium-organic coordination compound improves neuropathologies and cognitive functions in Alzheimer transgenic mice
Source: EMBO Mol Med. 2024 Oct 11;16(11):2856–81. doi: 10.1038/s44321-024-00146-7 (PMC11555261; doi:10.1038/s44321-024-00146-7)
Supplement: Supplementary file 3 — Source data Fig. 1 [file 44321_2024_146_MOESM3_ESM.zip › Fig. 1/Fig. 1 pptx.pdf]

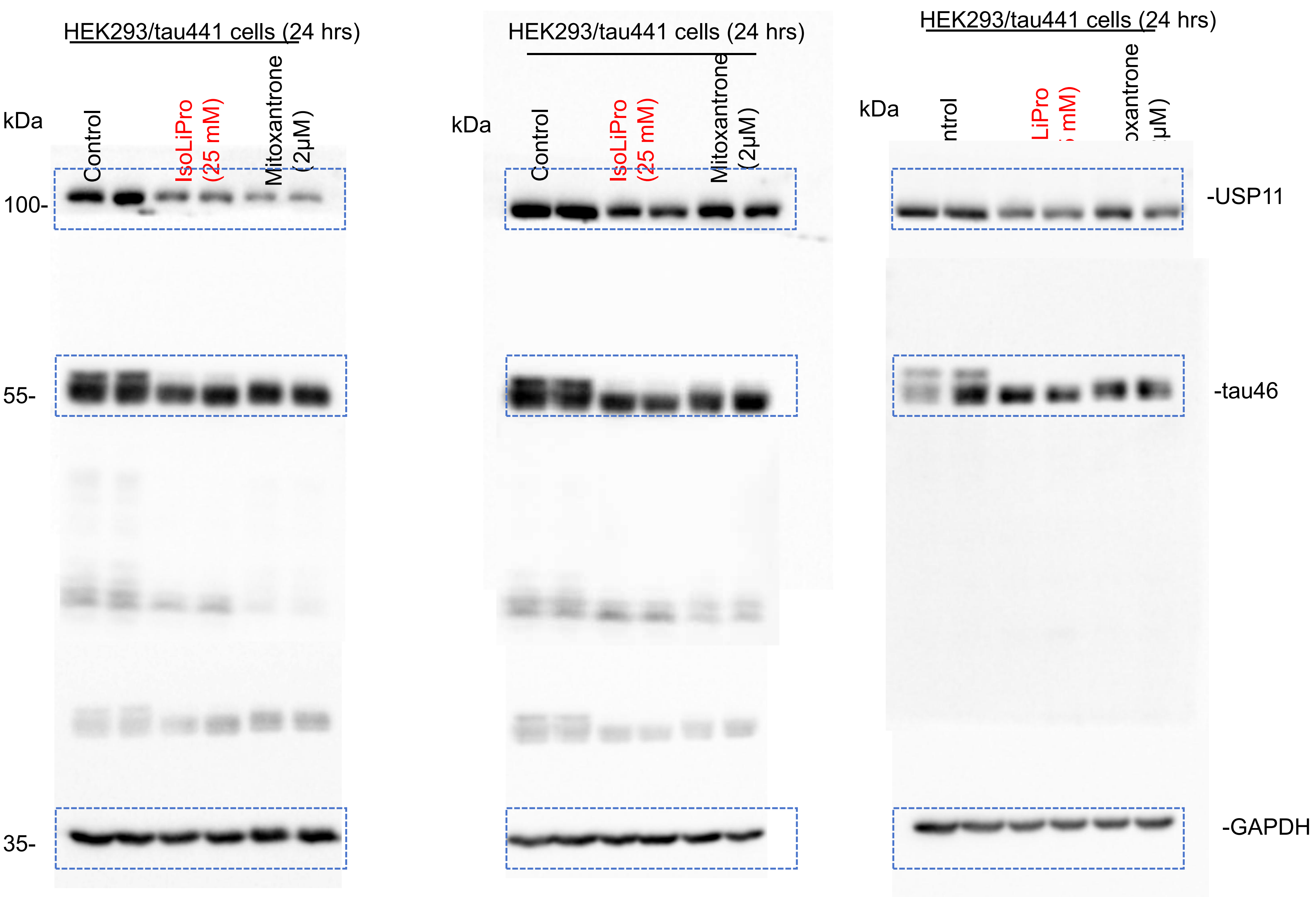

Full unedited gel for Fig. 1 A

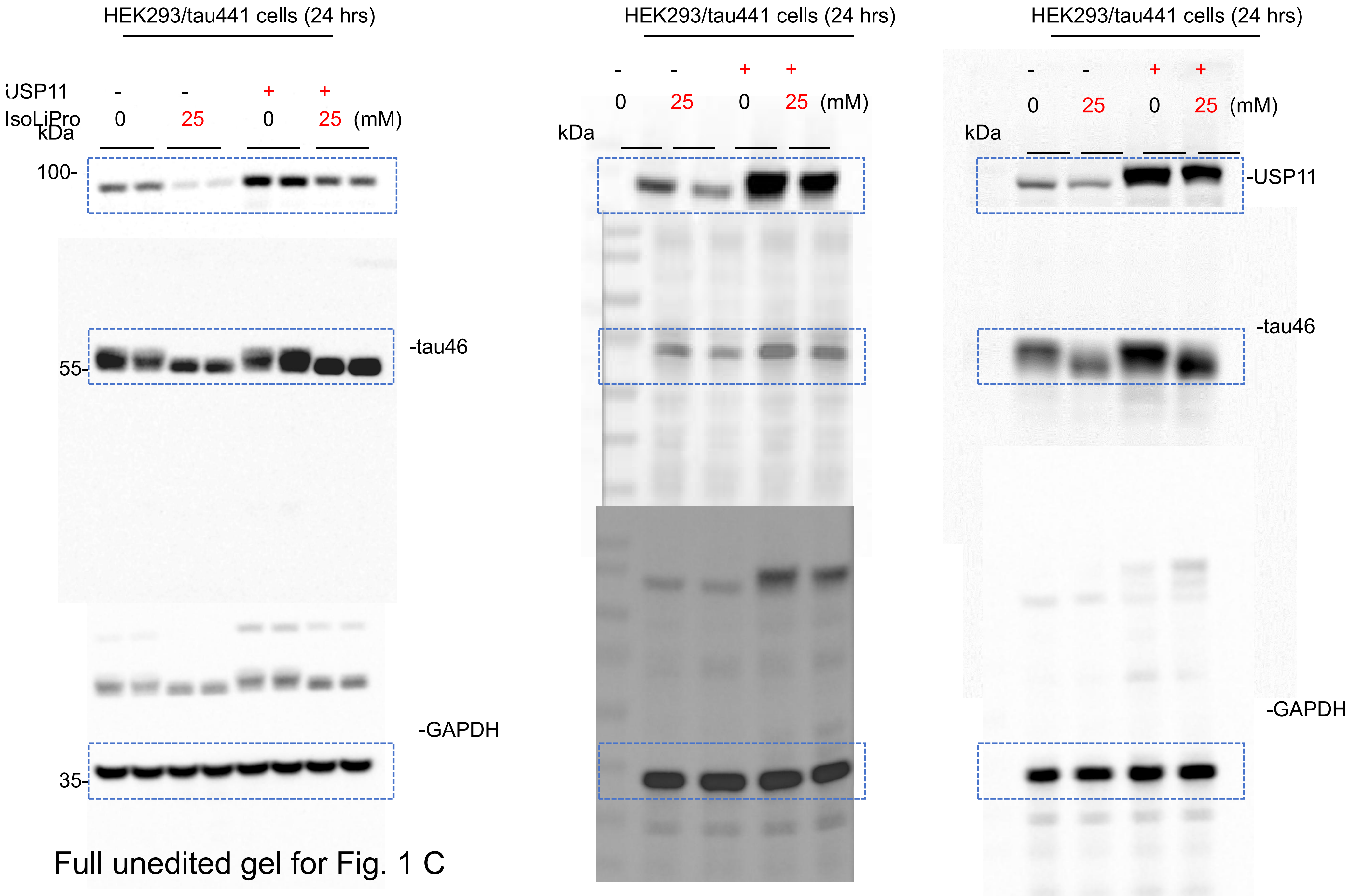

Full unedited gel for Fig. 1 C

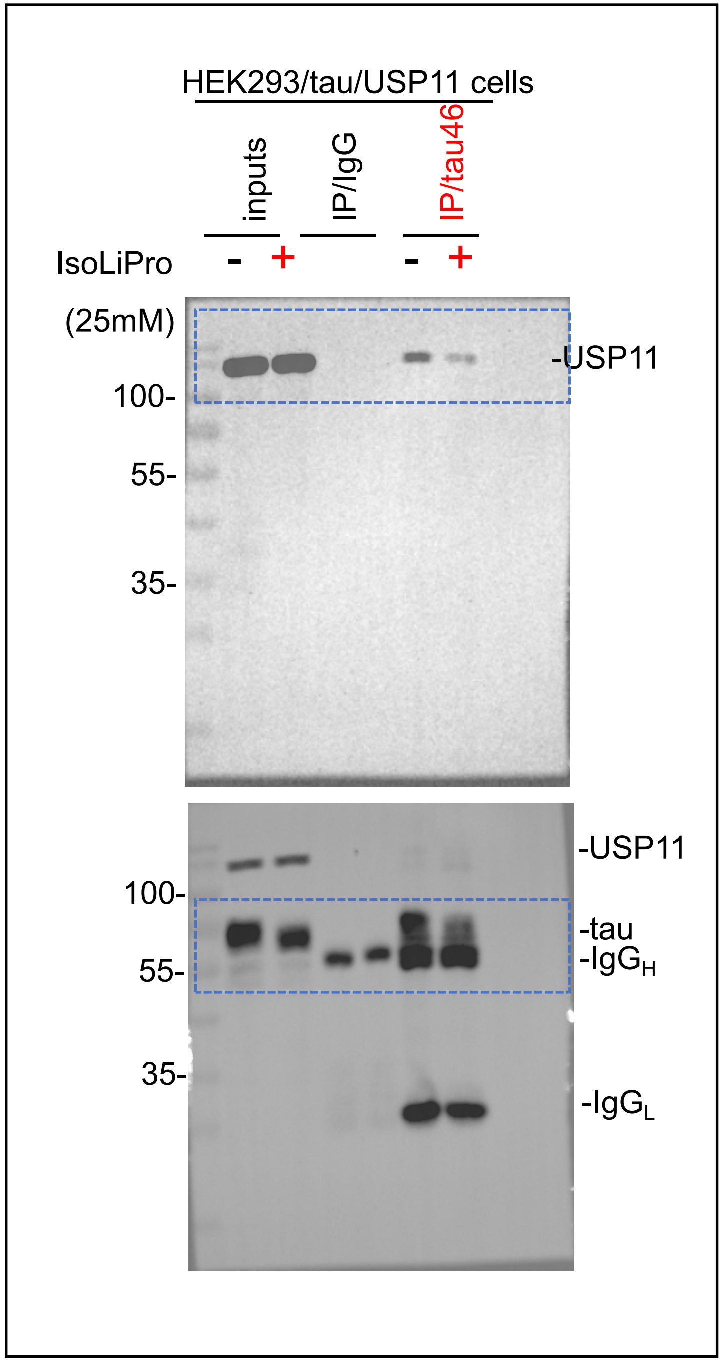
HEK293/tau/USP11 cells
inputs
IP/IgG
IP/tau46
IsoLiPro
-
+
-
+
(25mM)
-USP11
100-
55-
35-
-USP11
100-
-tau
-IgGH
55-
35-
-IgGL

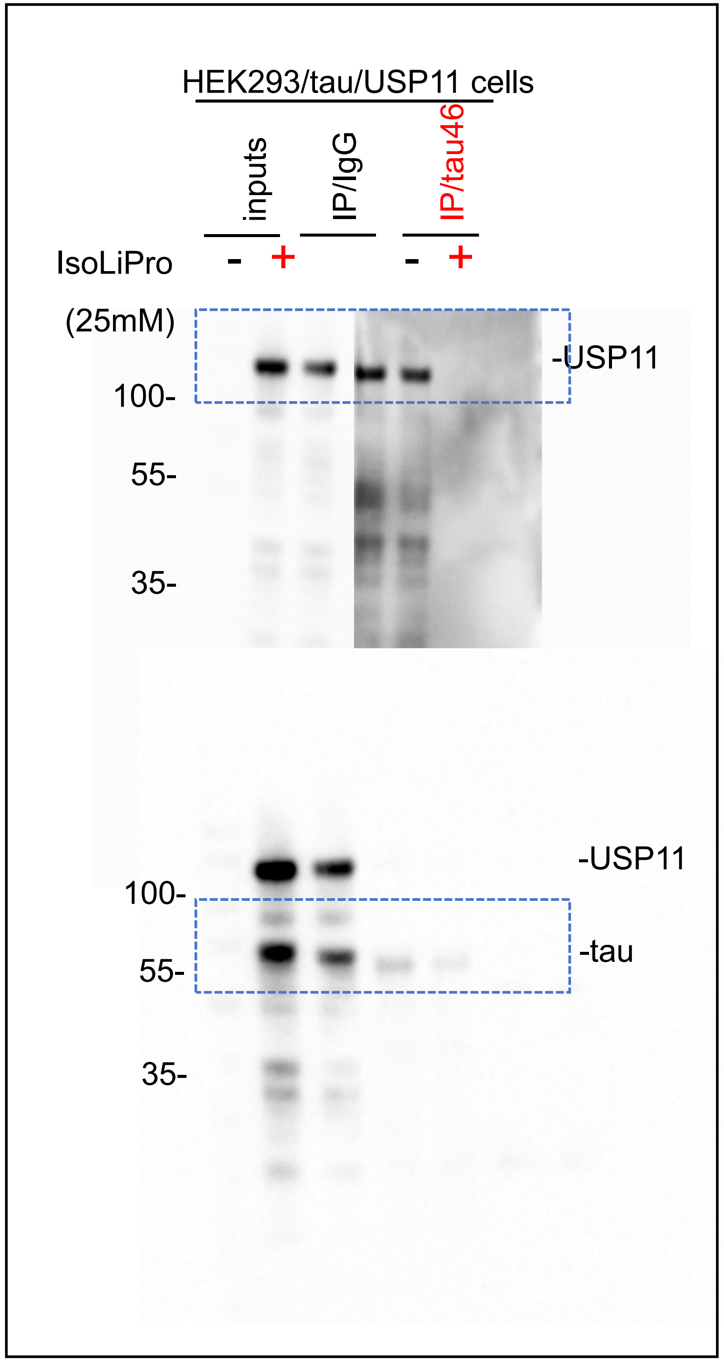
HEK293/tau/USP11 cells
inputs
IP/IgG
IP/tau46
IsoLiPro
-
+
-
+
(25mM)
-USP11
100-
55-
35-
-USP11
100-
-tau
55-
35-

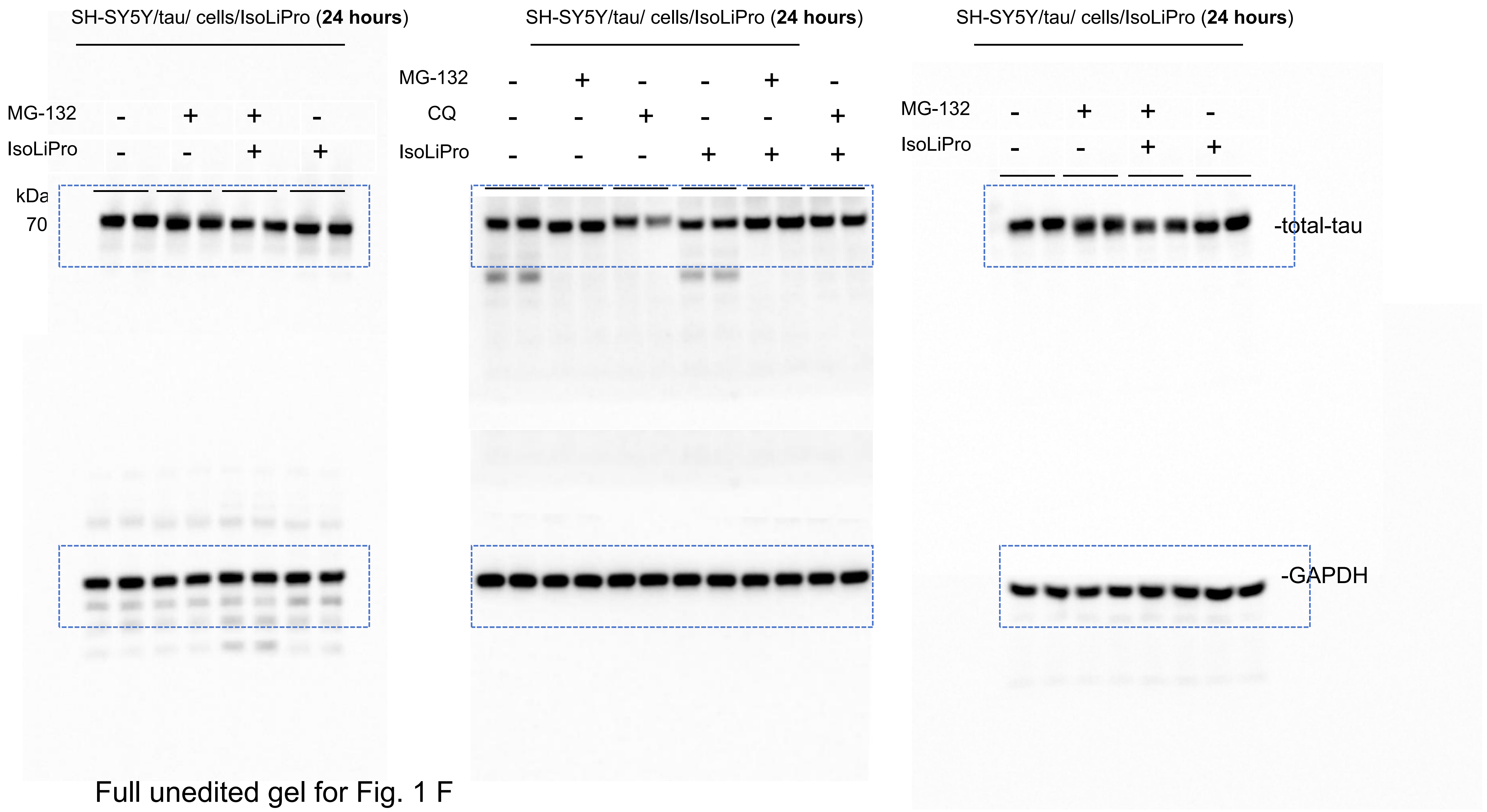

Full unedited gel for Fig. 1 F

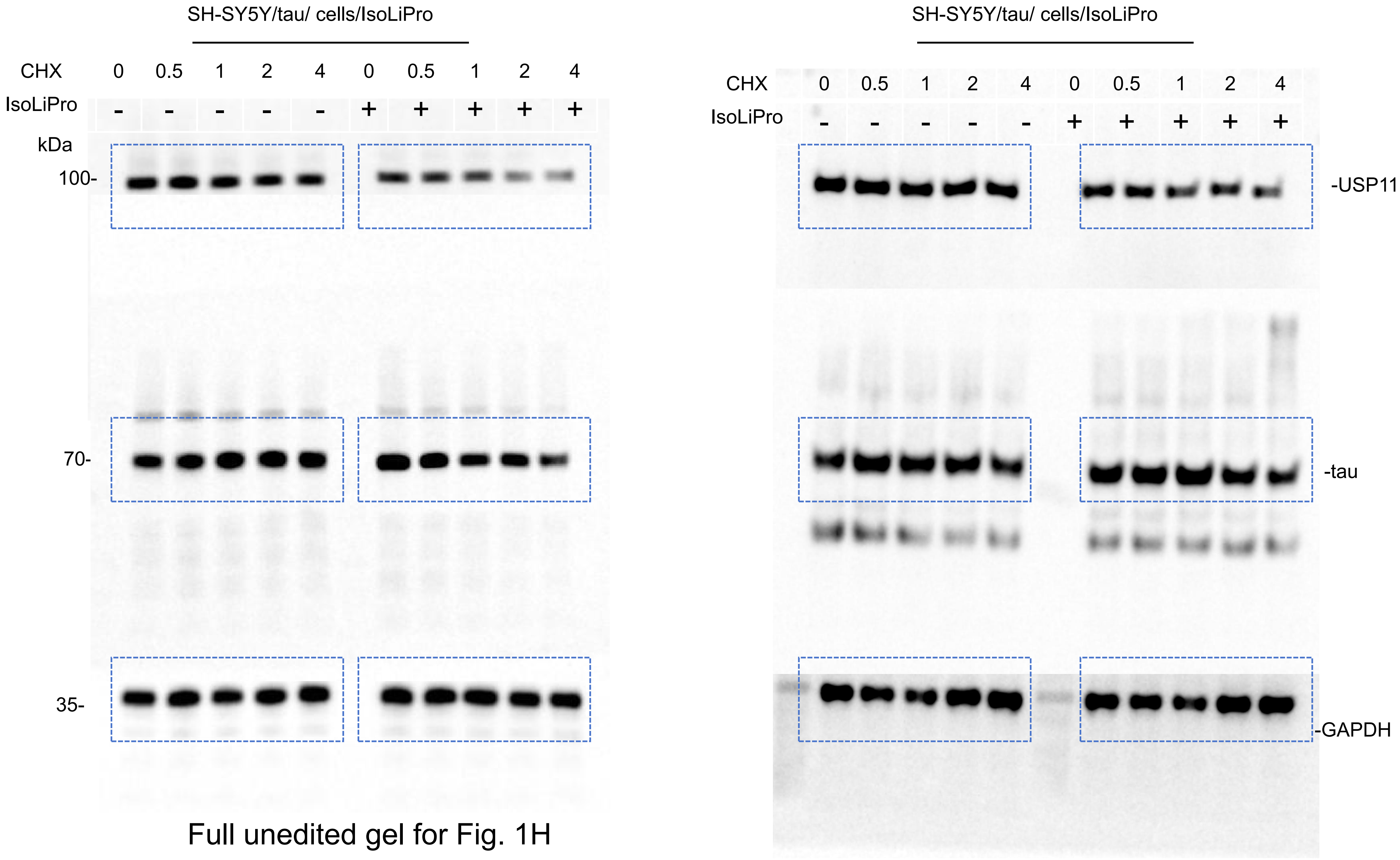

Full unedited gel for Fig. 1H

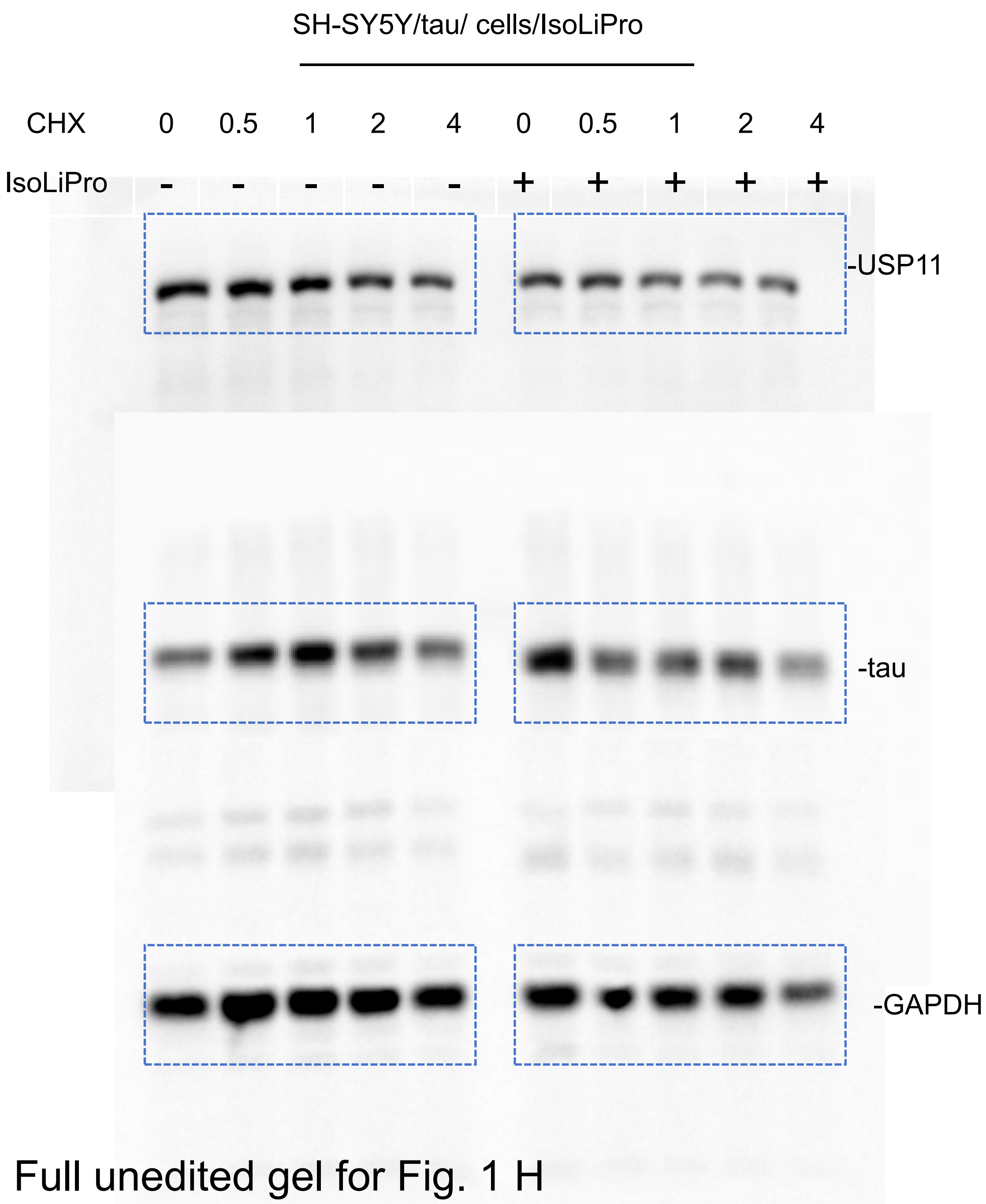

Full unedited gel for Fig. 1 H

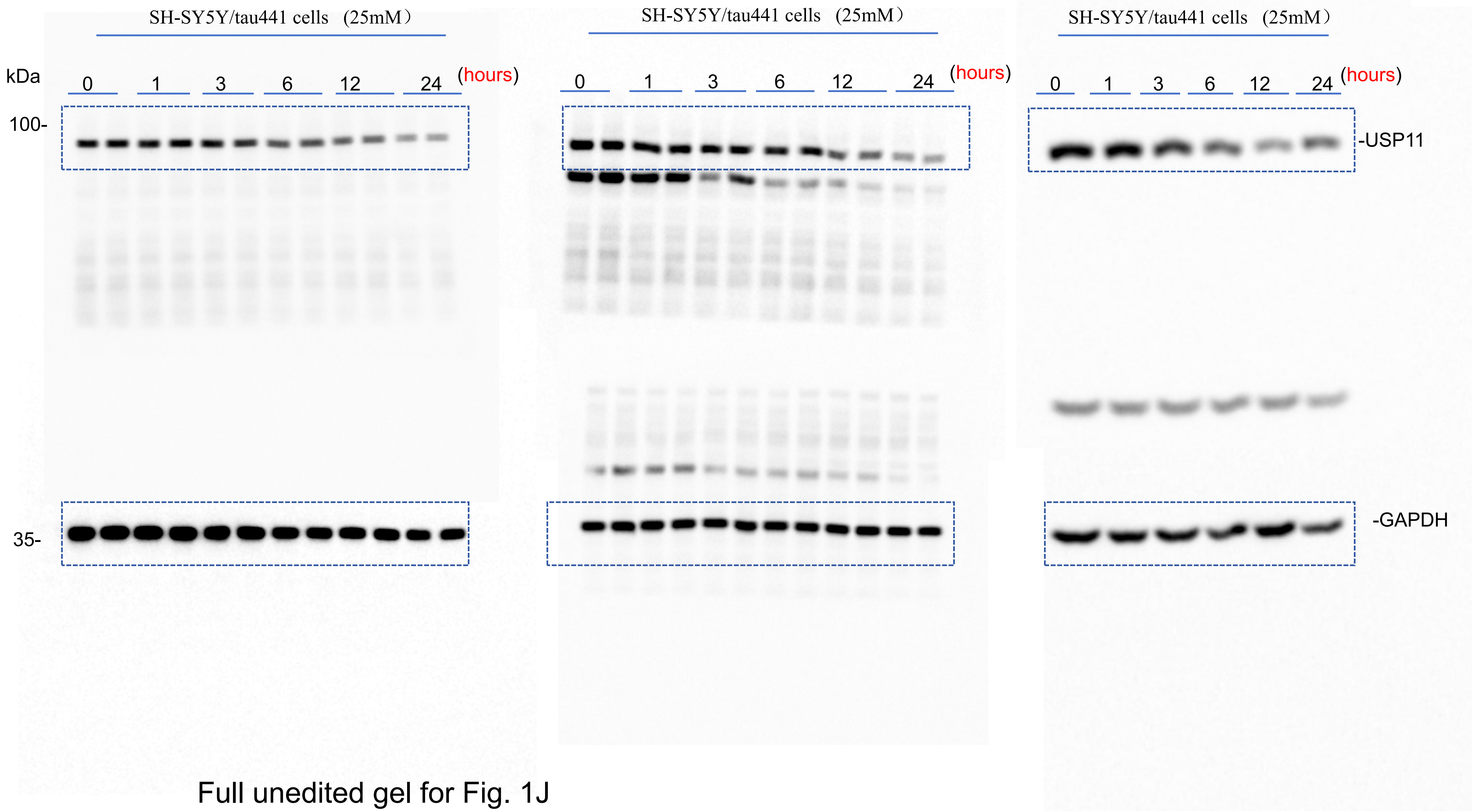

Full unedited gel for Fig. 1J

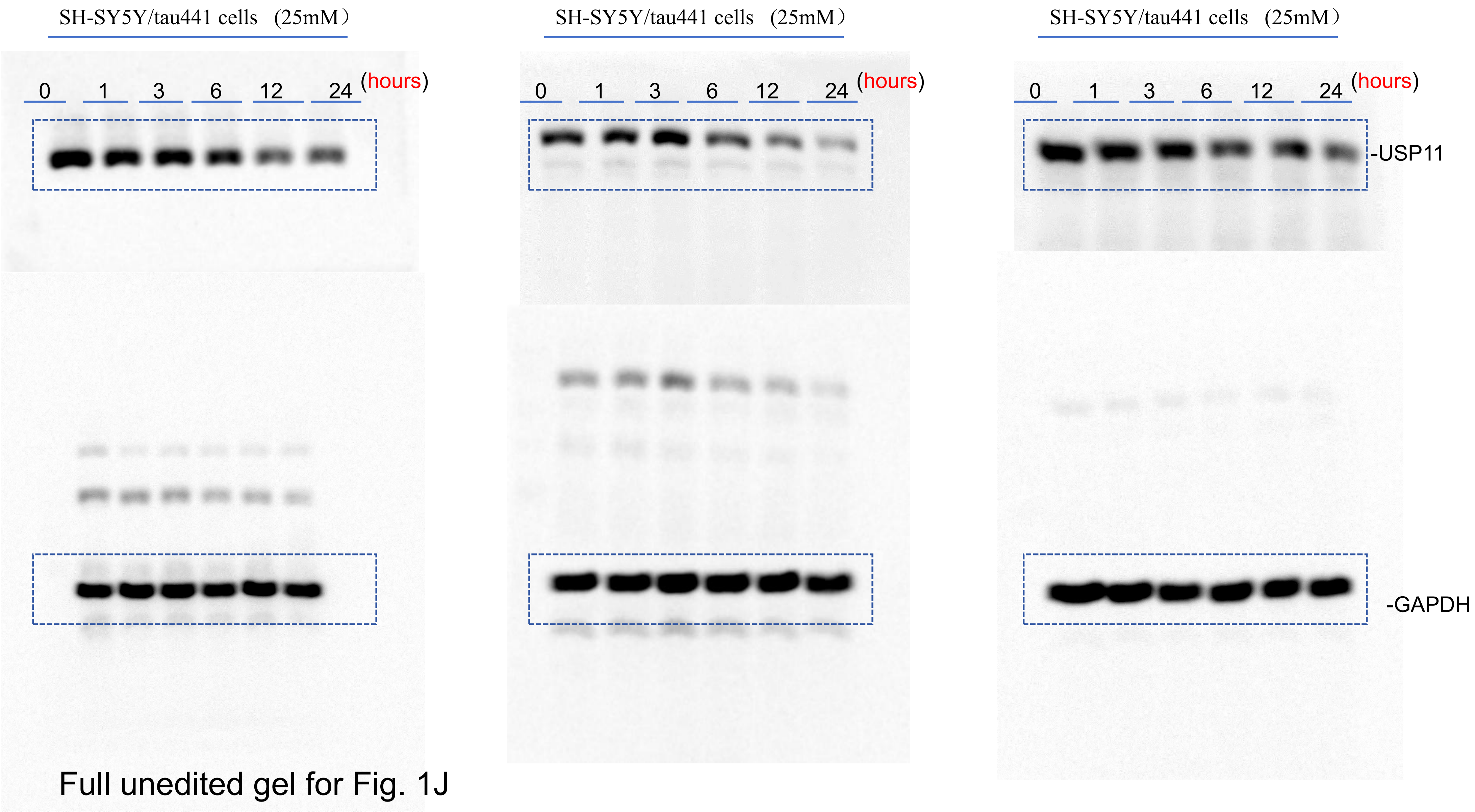

Full unedited gel for Fig. 1J

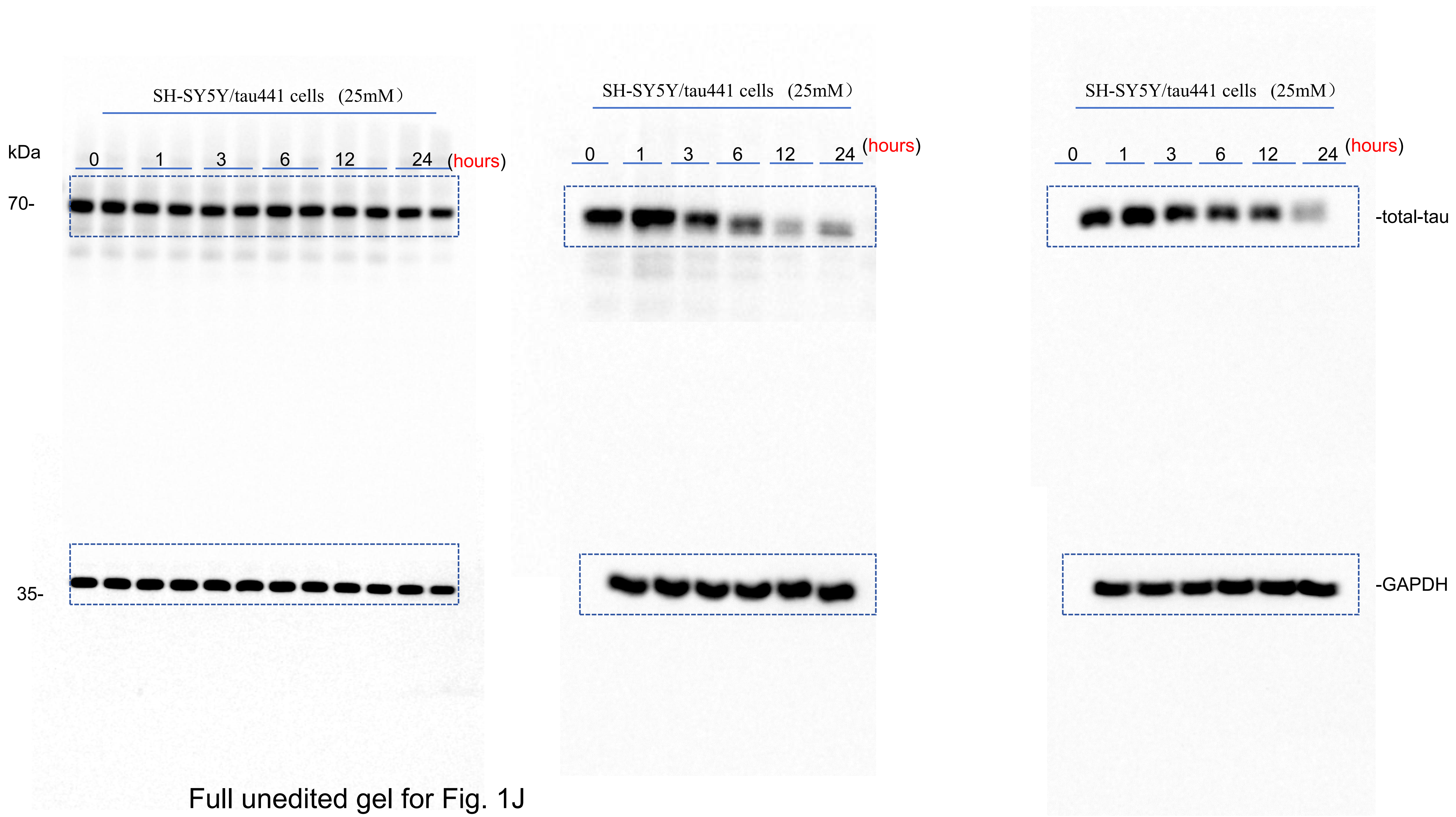

Full unedited gel for Fig. 1J

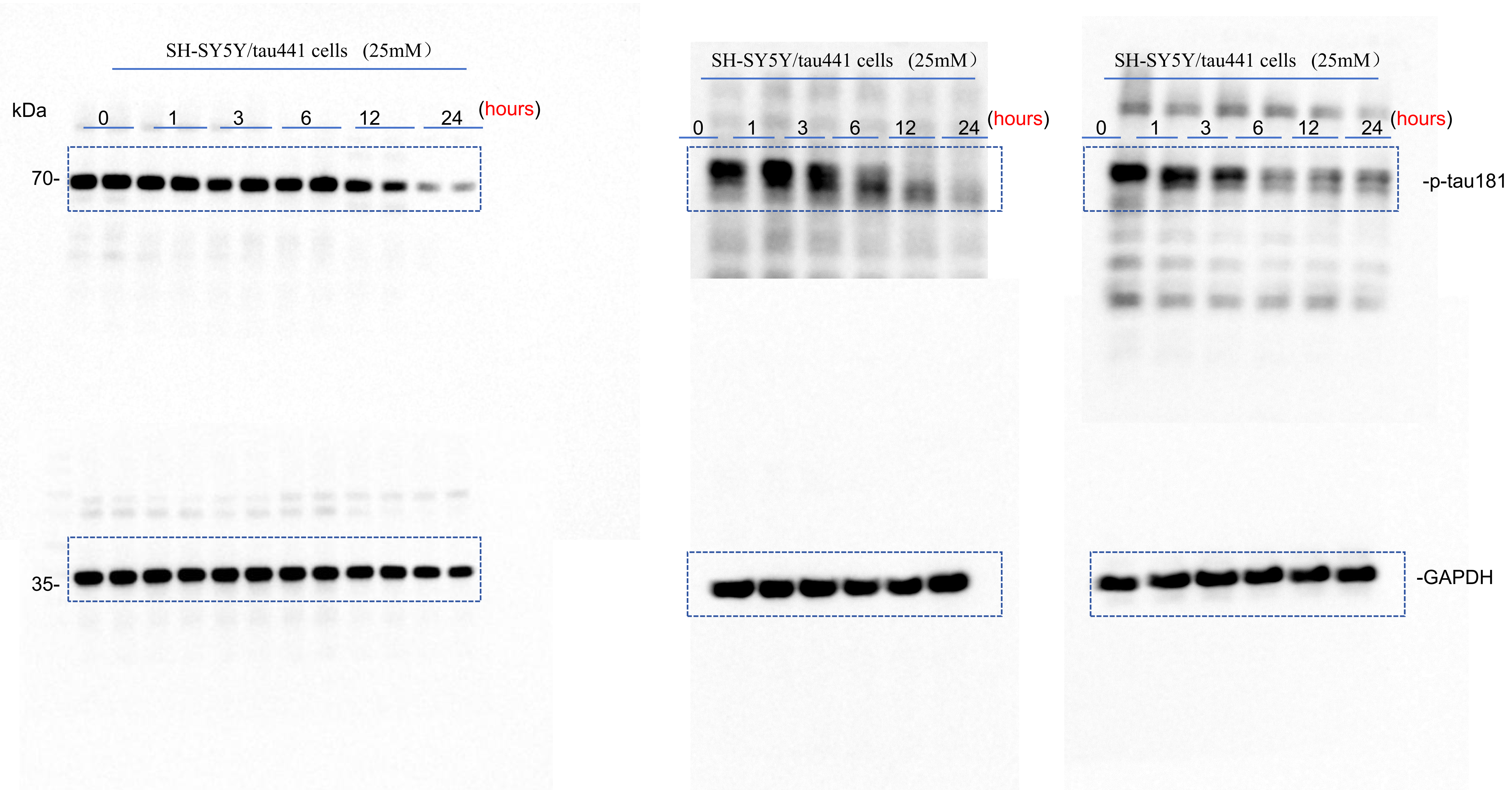

Full unedited gel for Fig. 1F

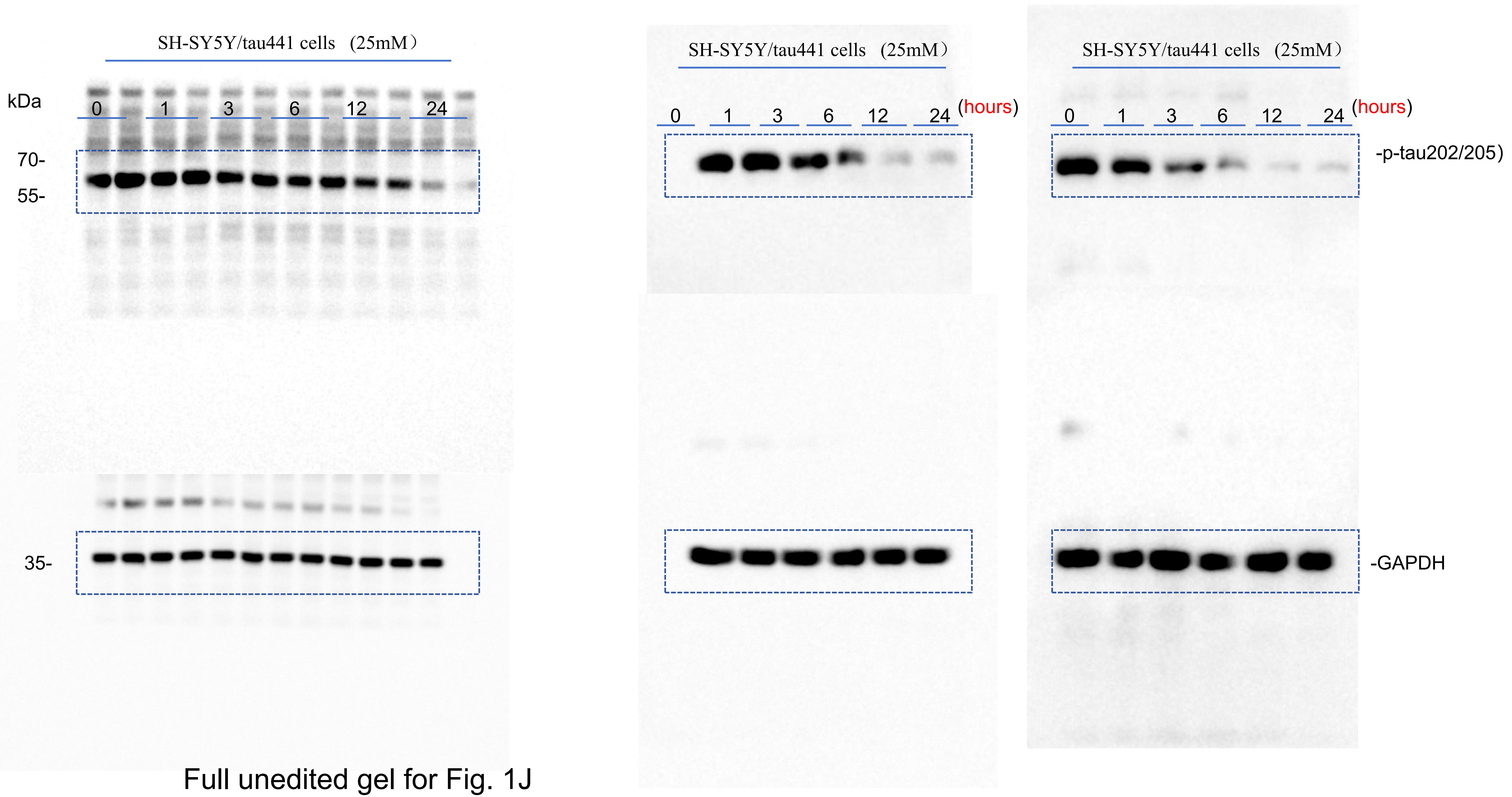

Full unedited gel for Fig. 1J

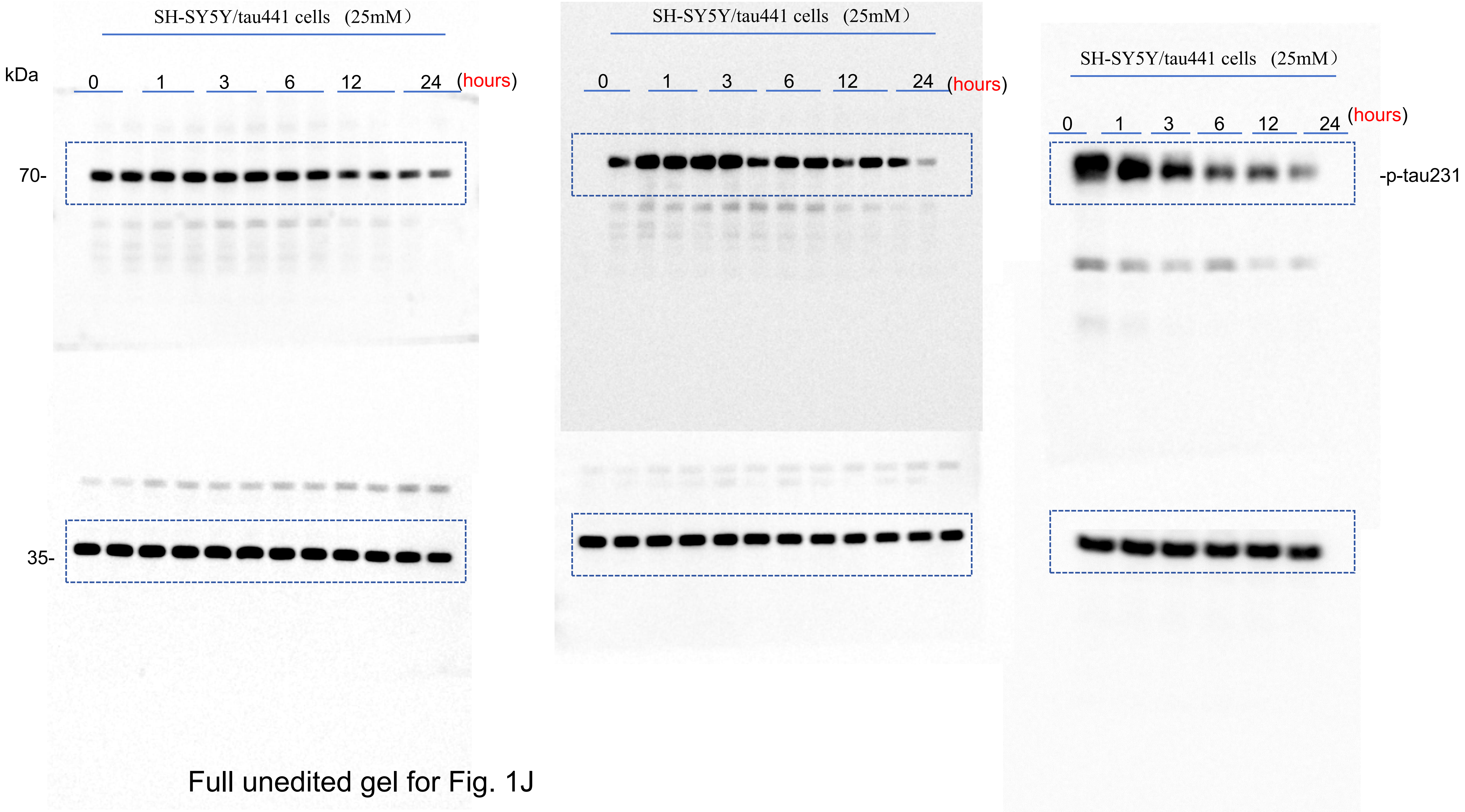

Full unedited gel for Fig. 1J

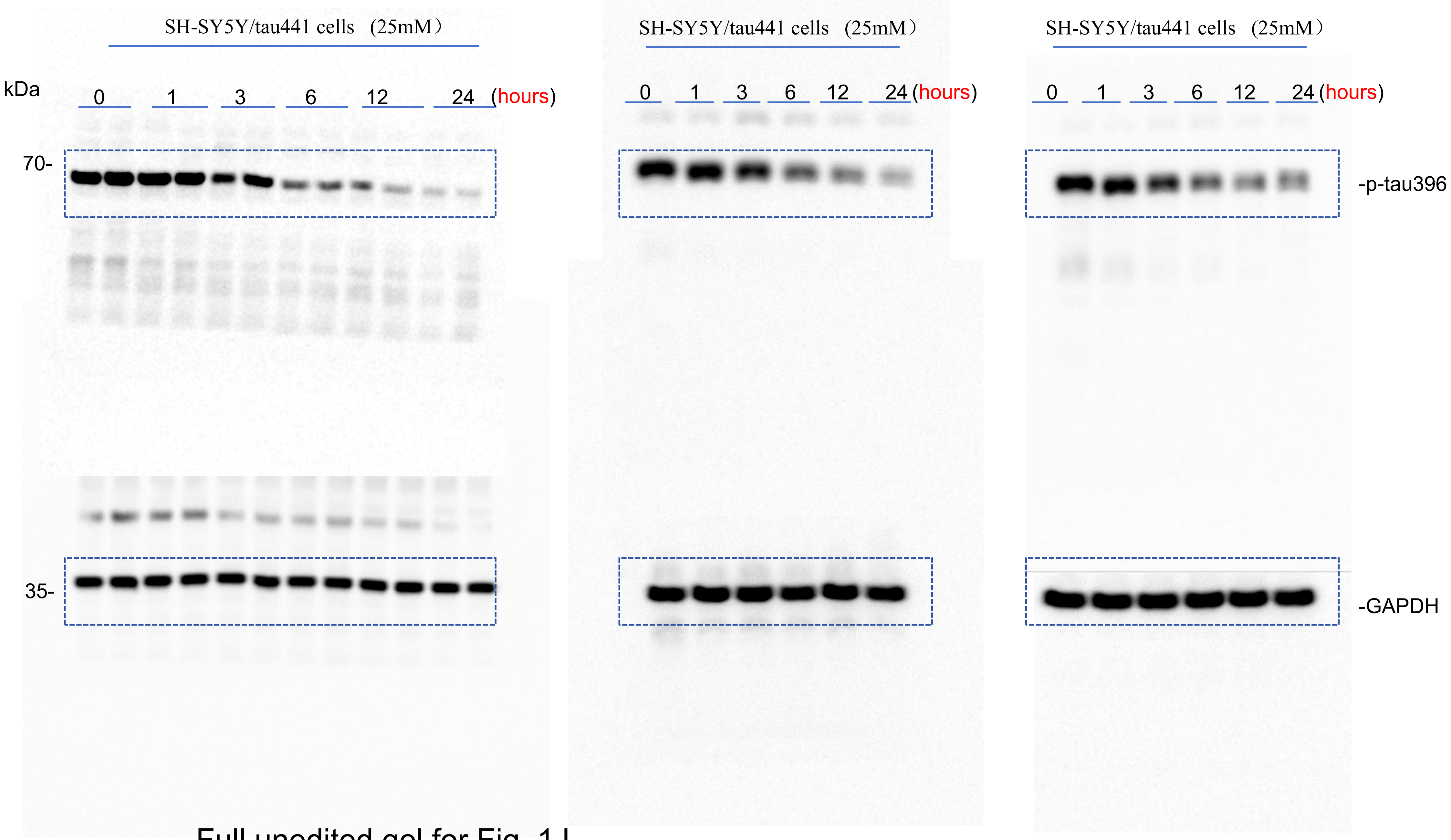

Full unedited gel for Fig. 1J

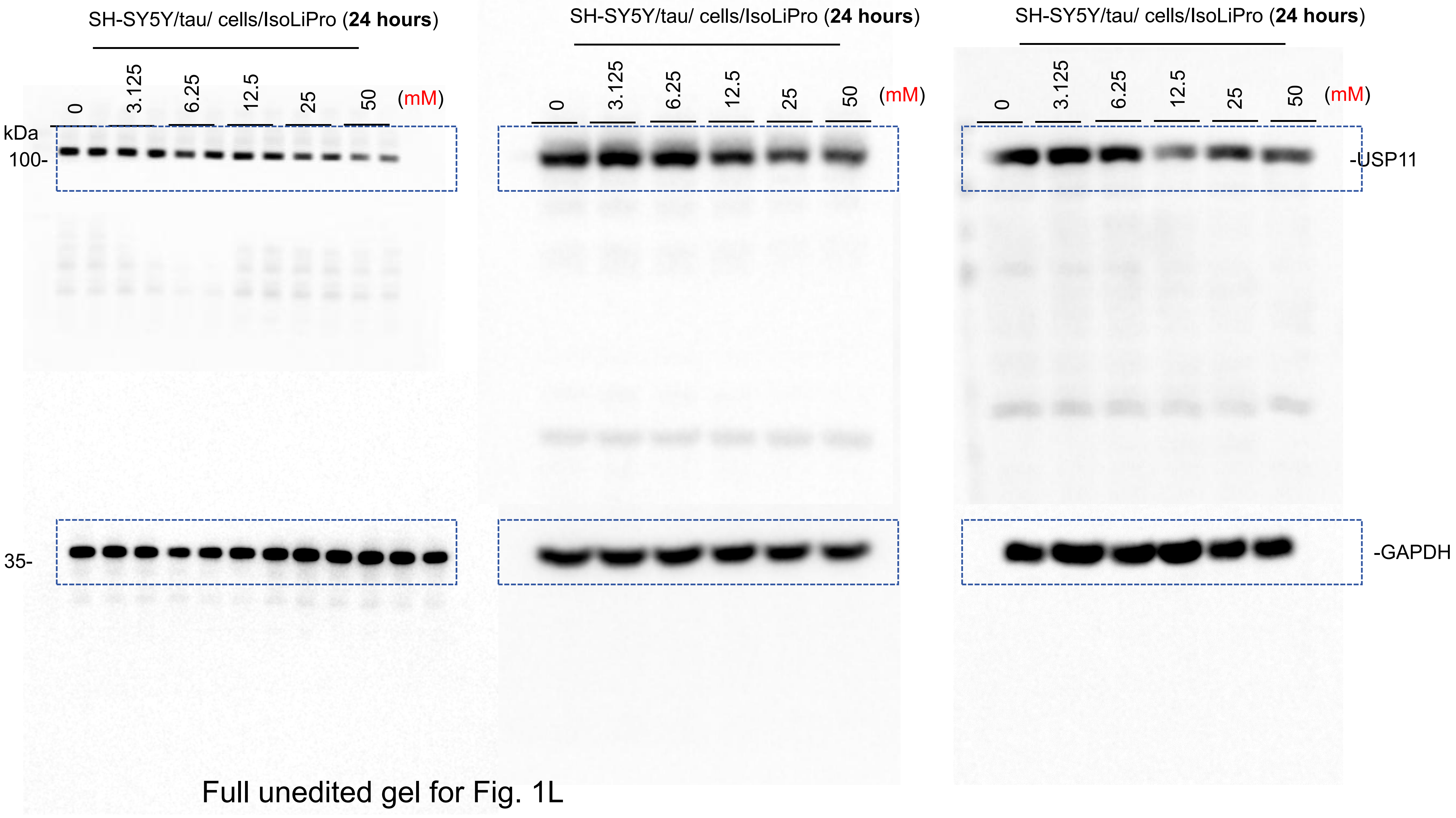

Full unedited gel for Fig. 1L

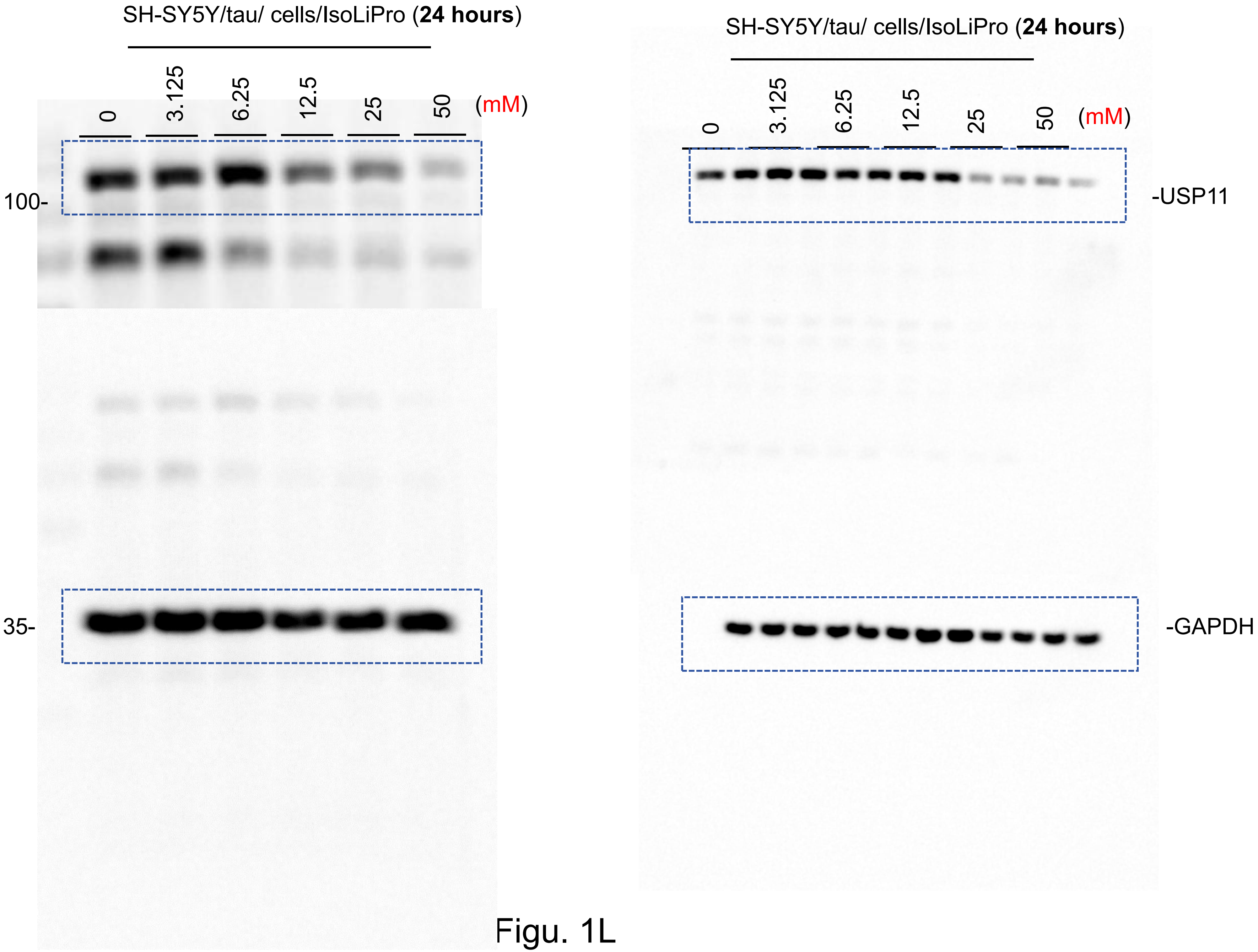
SH-SY5Y/tau/ cells/IsoLiPro (24 hours)
0
3.125
6.25
12.5
25
50
(mM)
100-
35-
SH-SY5Y/tau/ cells/IsoLiPro (24 hours)
0
3.125
6.25
12.5
25
50
(mM)
-USP11
-GAPDH

Figu. 1L

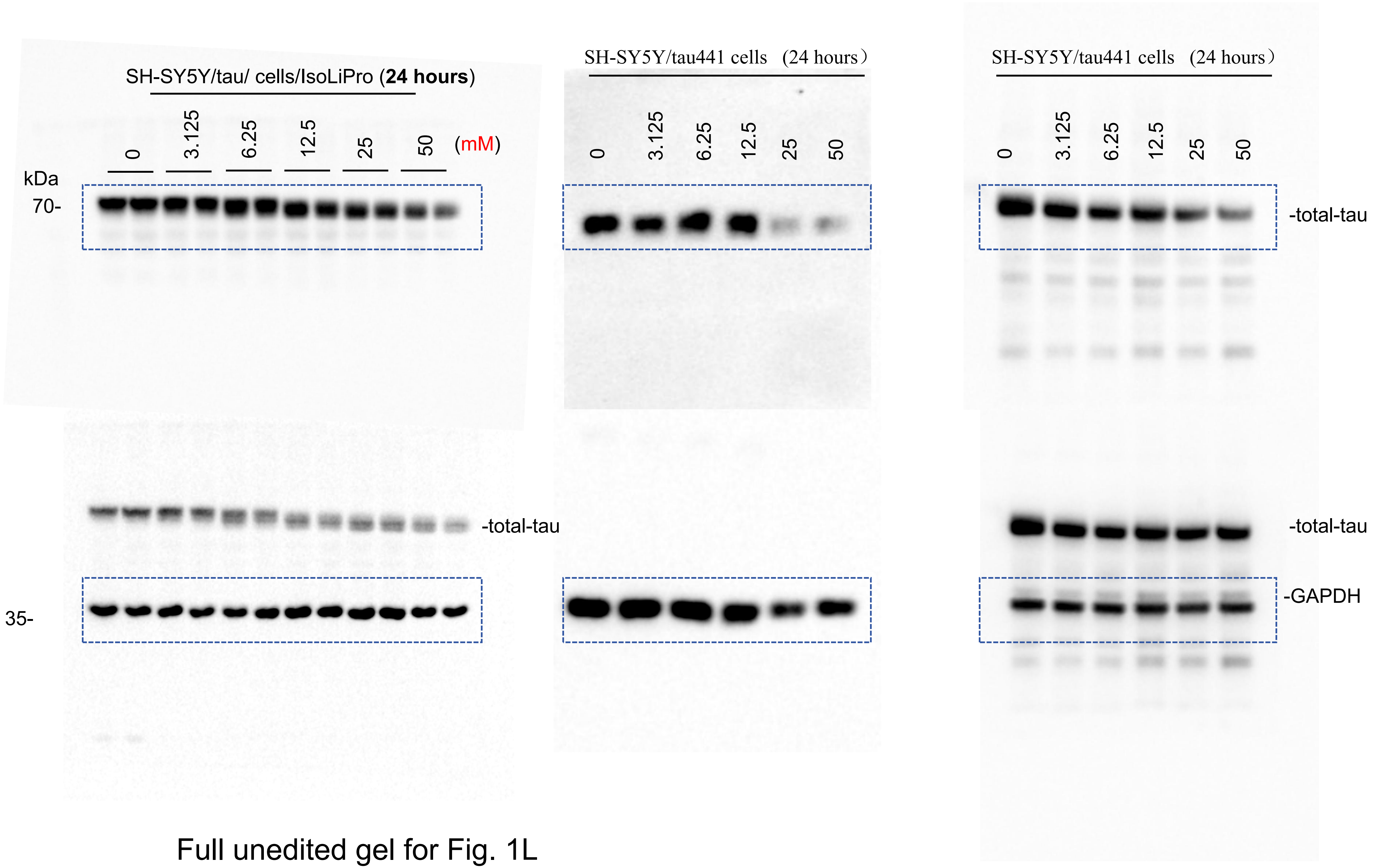

Full unedited gel for Fig. 1L

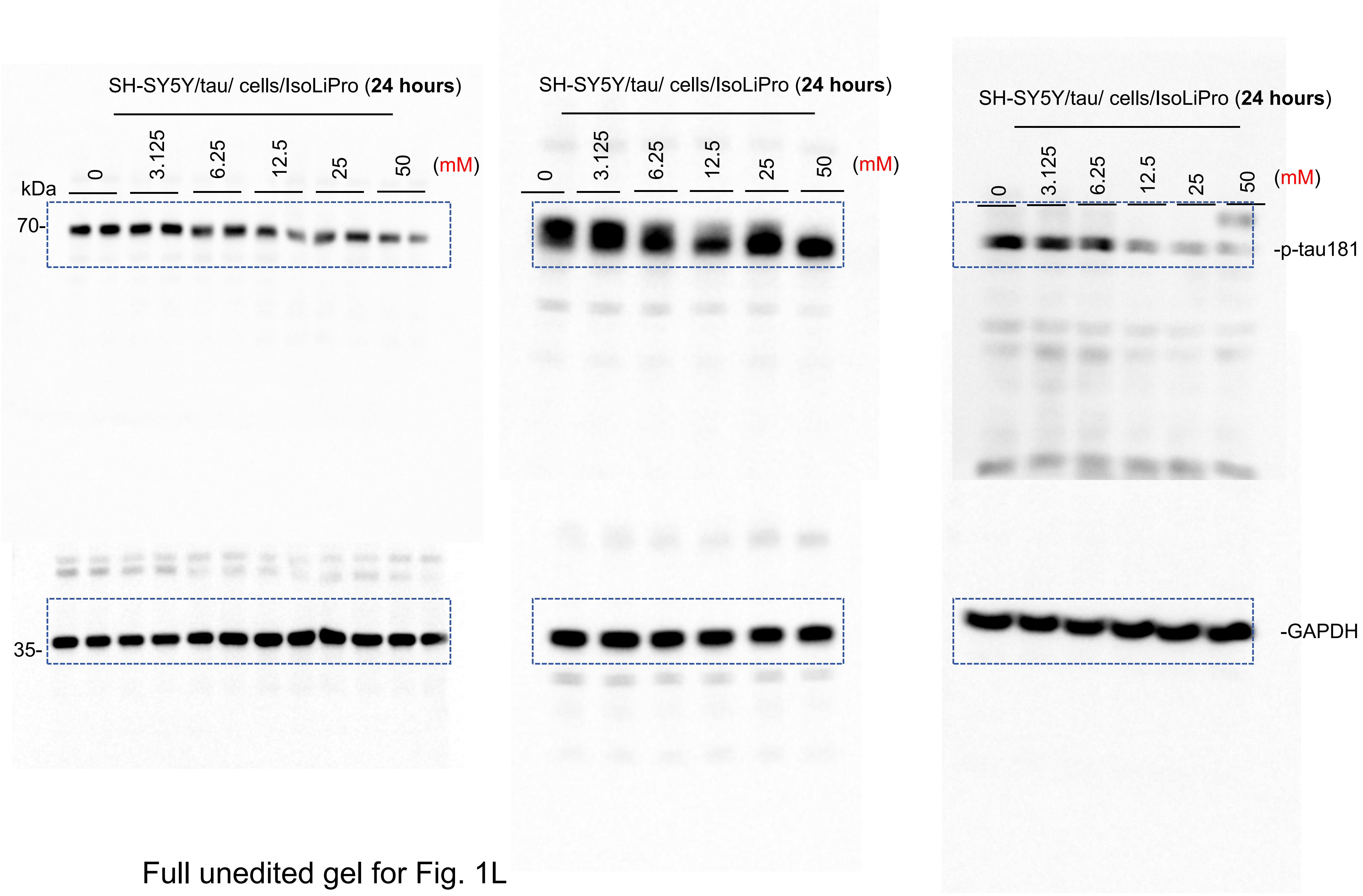

Full unedited gel for Fig. 1L

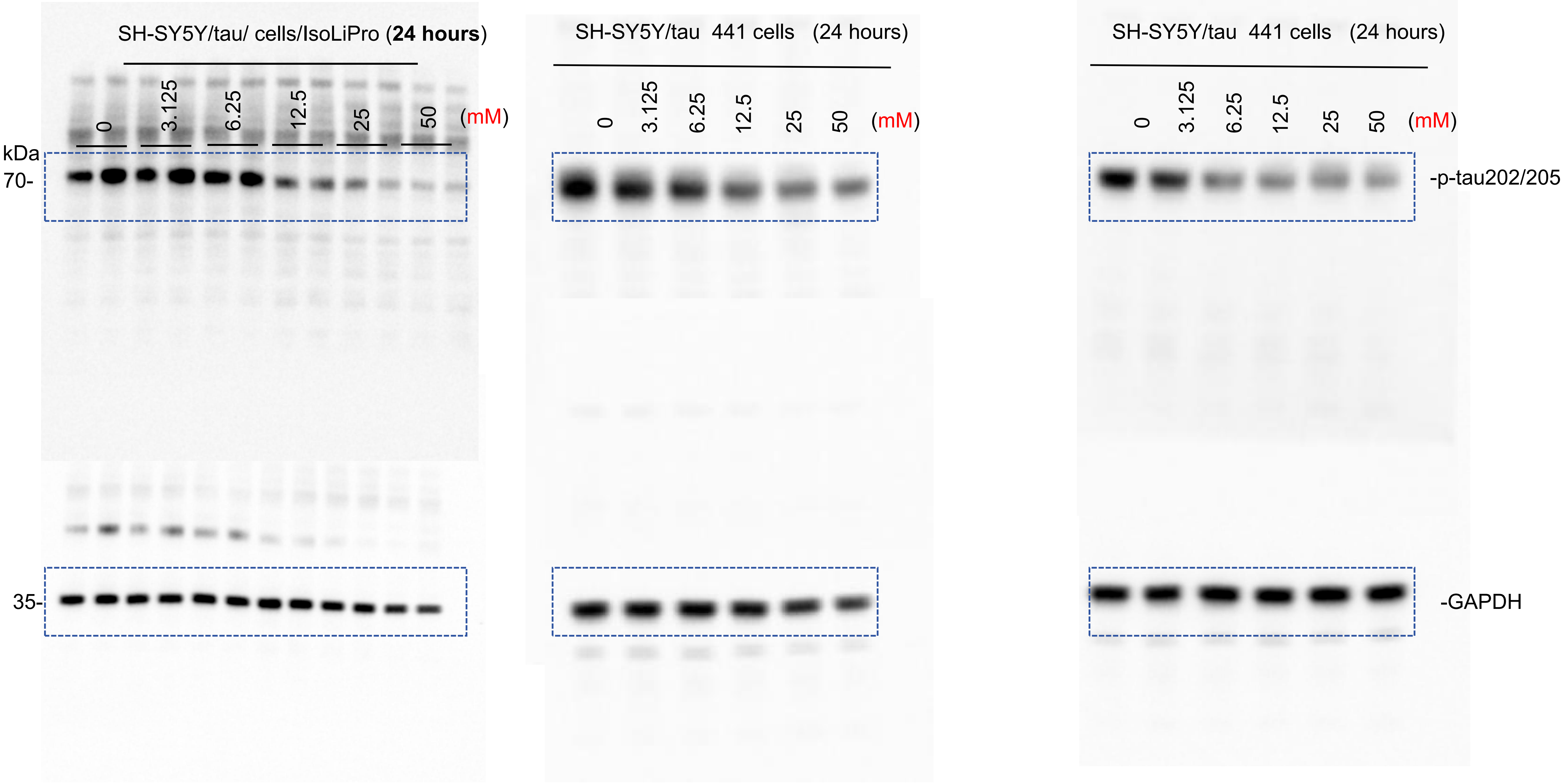

Full unedited gel for Fig. 1L

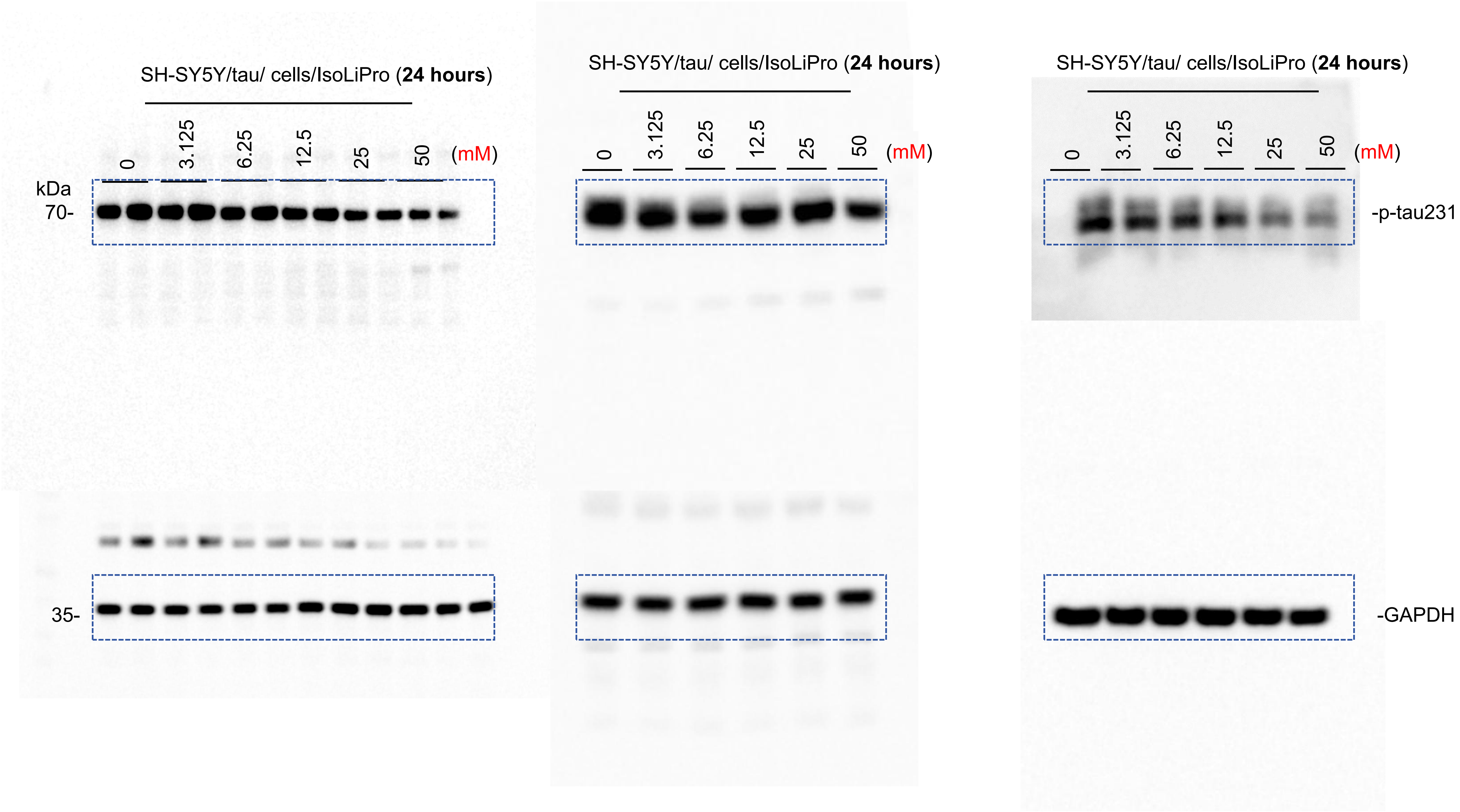

Full unedited gel for Fig. 1L

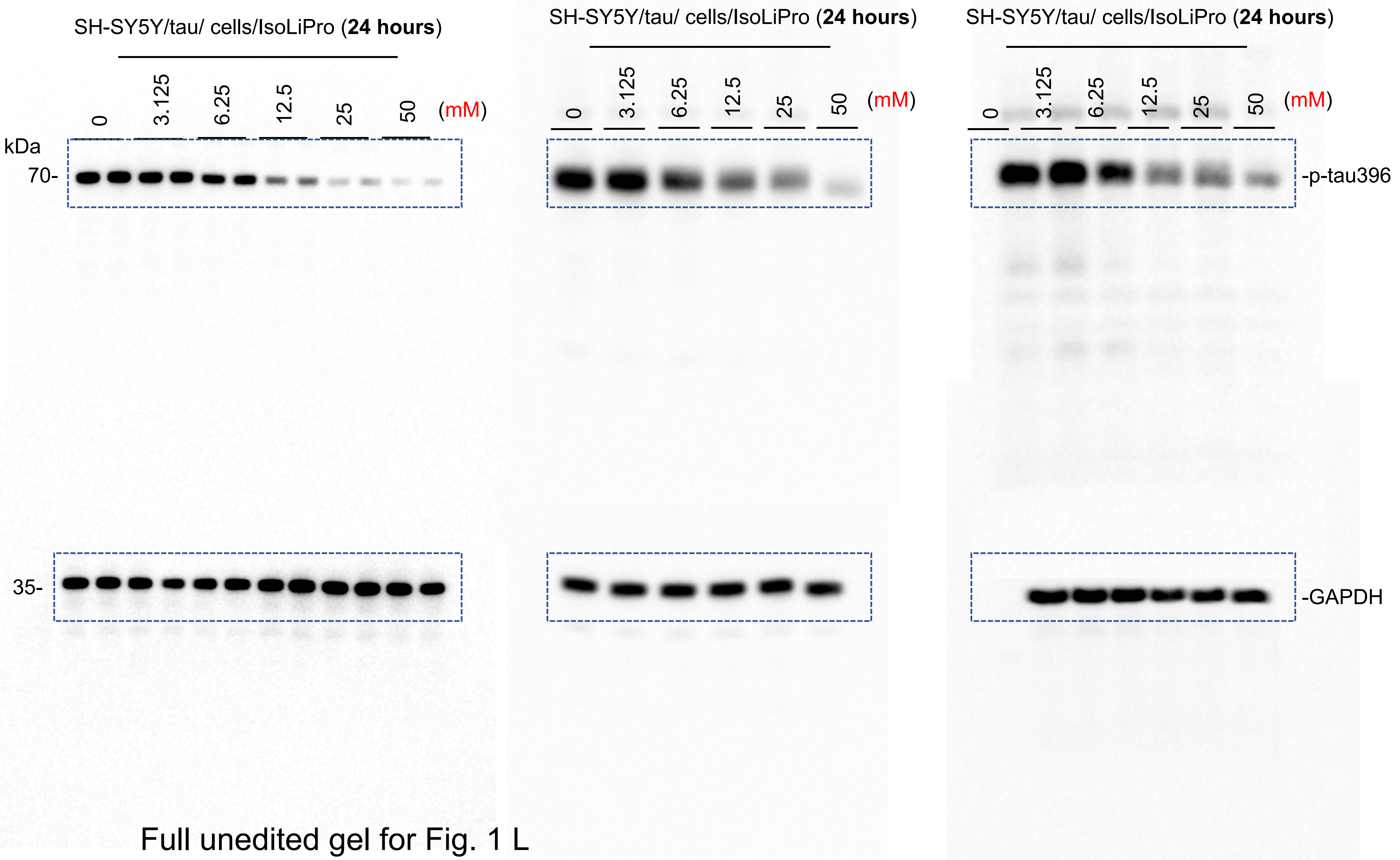

Full unedited gel for Fig. 1 L
