# Supplementary figures and images for: Targeting USP11 regulation by a novel lithium-organic coordination compound improves neuropathologies and cognitive functions in Alzheimer transgenic mice (part 1 of 2)
Source: EMBO Mol Med. 2024 Oct 11;16(11):2856–81. doi: 10.1038/s44321-024-00146-7 (PMC11555261; doi:10.1038/s44321-024-00146-7)

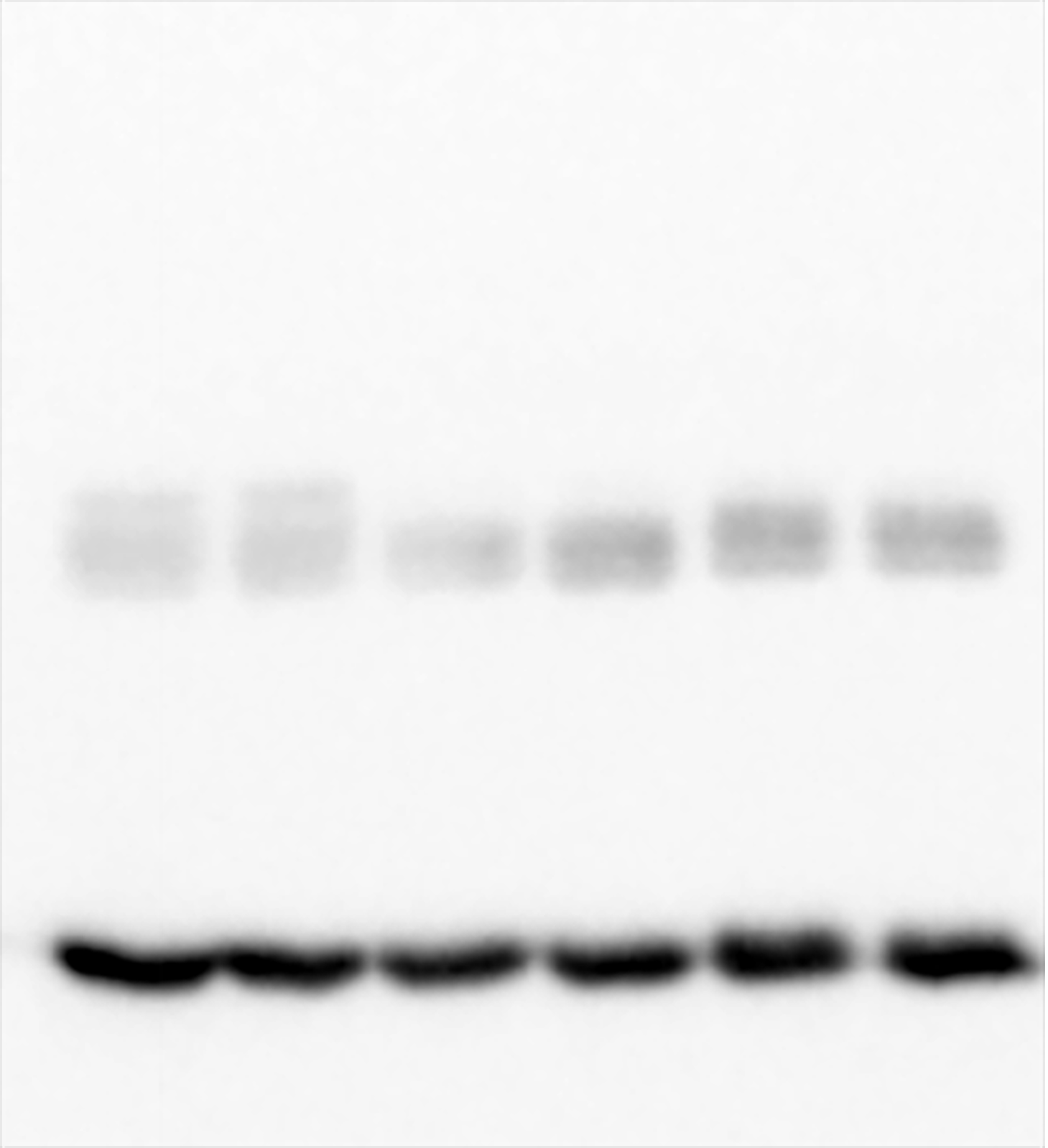

Supplement: Supplementary file 3 — Source data Fig. 1 [file 44321_2024_146_MOESM3_ESM.zip › Fig. 1/Fig. 1A/Fig. 1A-GAPDH.tif]

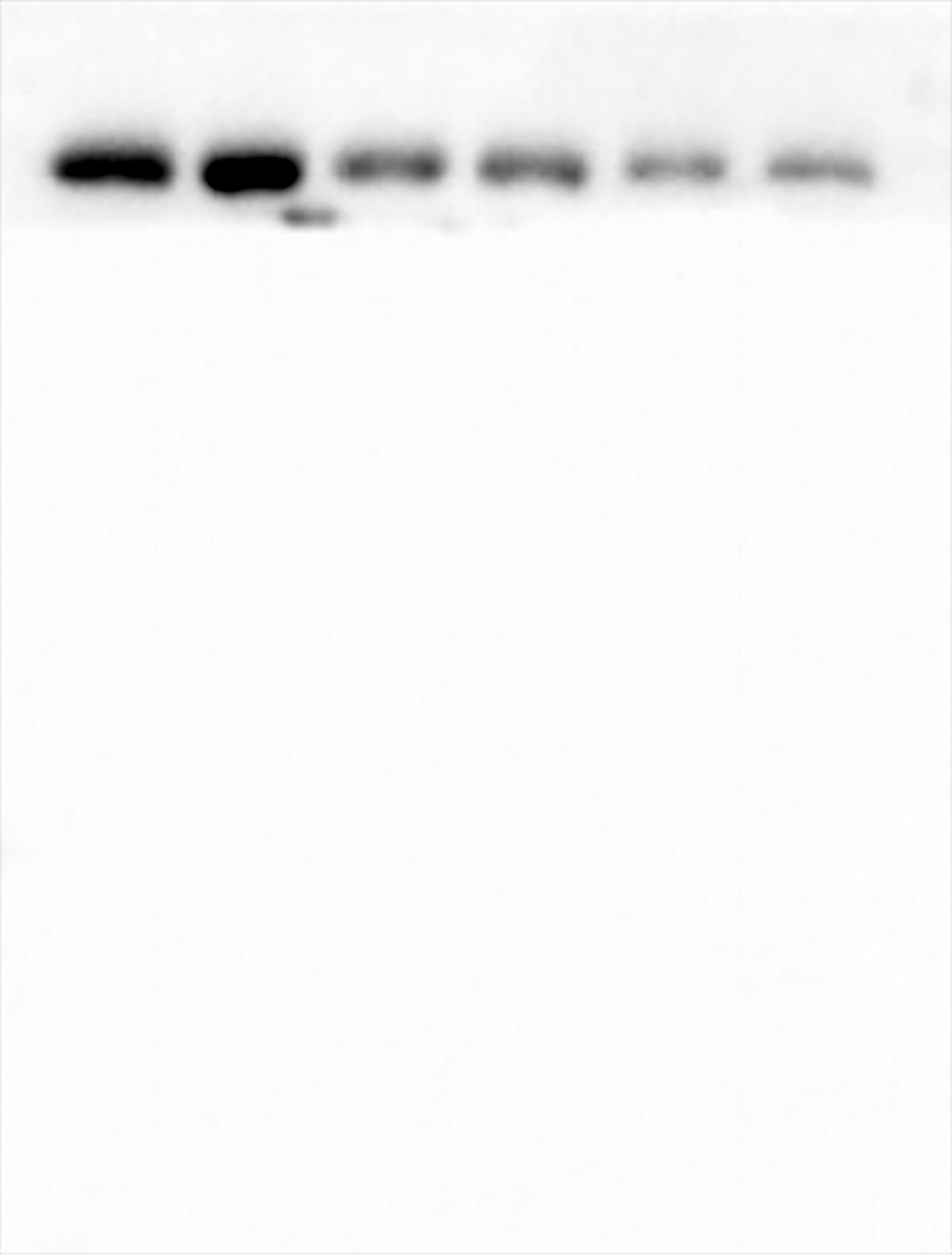

Supplement: Supplementary file 3 — Source data Fig. 1 [file 44321_2024_146_MOESM3_ESM.zip › Fig. 1/Fig. 1A/Fig. 2A-USP11.tif]

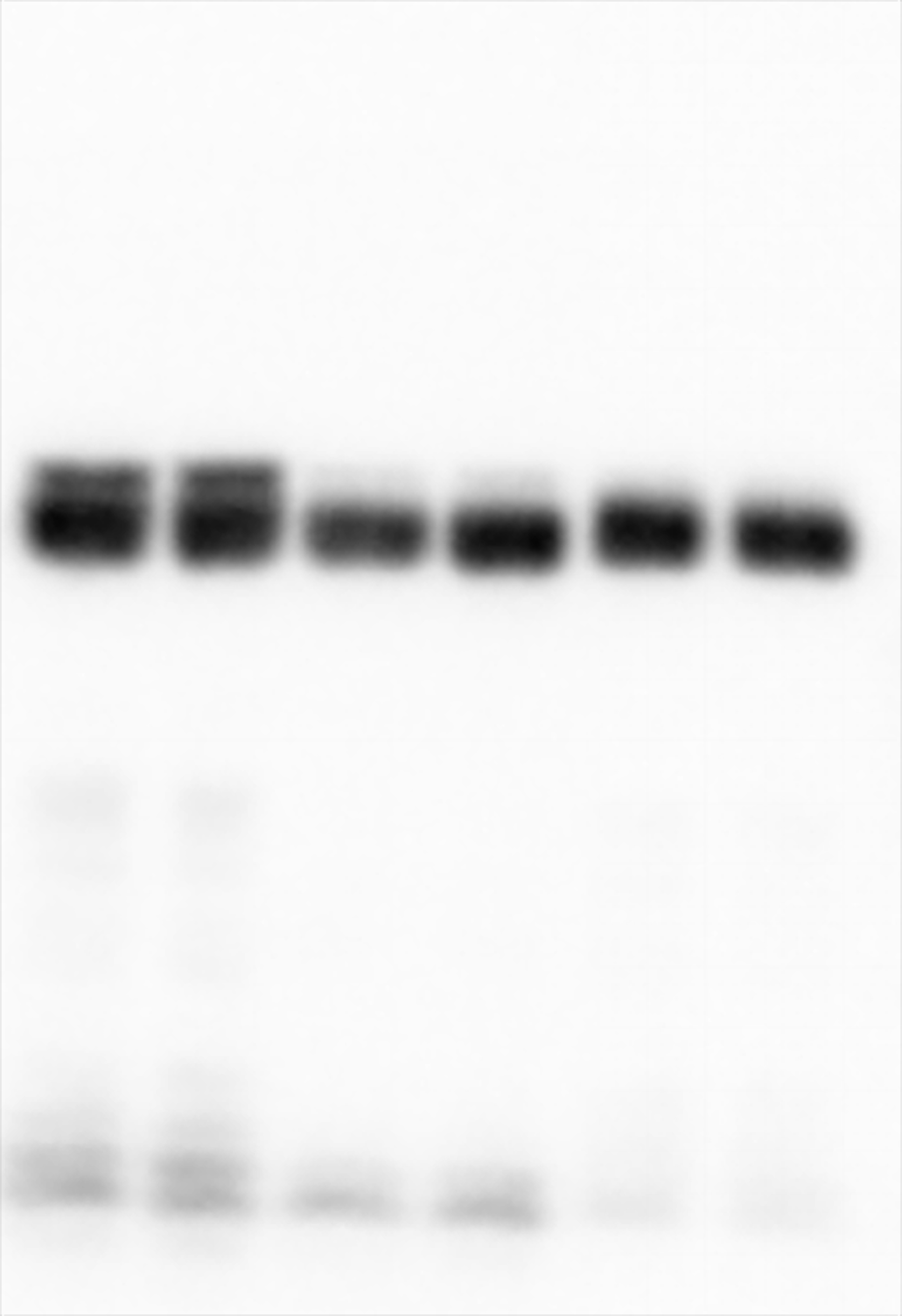

Supplement: Supplementary file 3 — Source data Fig. 1 [file 44321_2024_146_MOESM3_ESM.zip › Fig. 1/Fig. 1A/Fig. 1A-total-tau.tif]

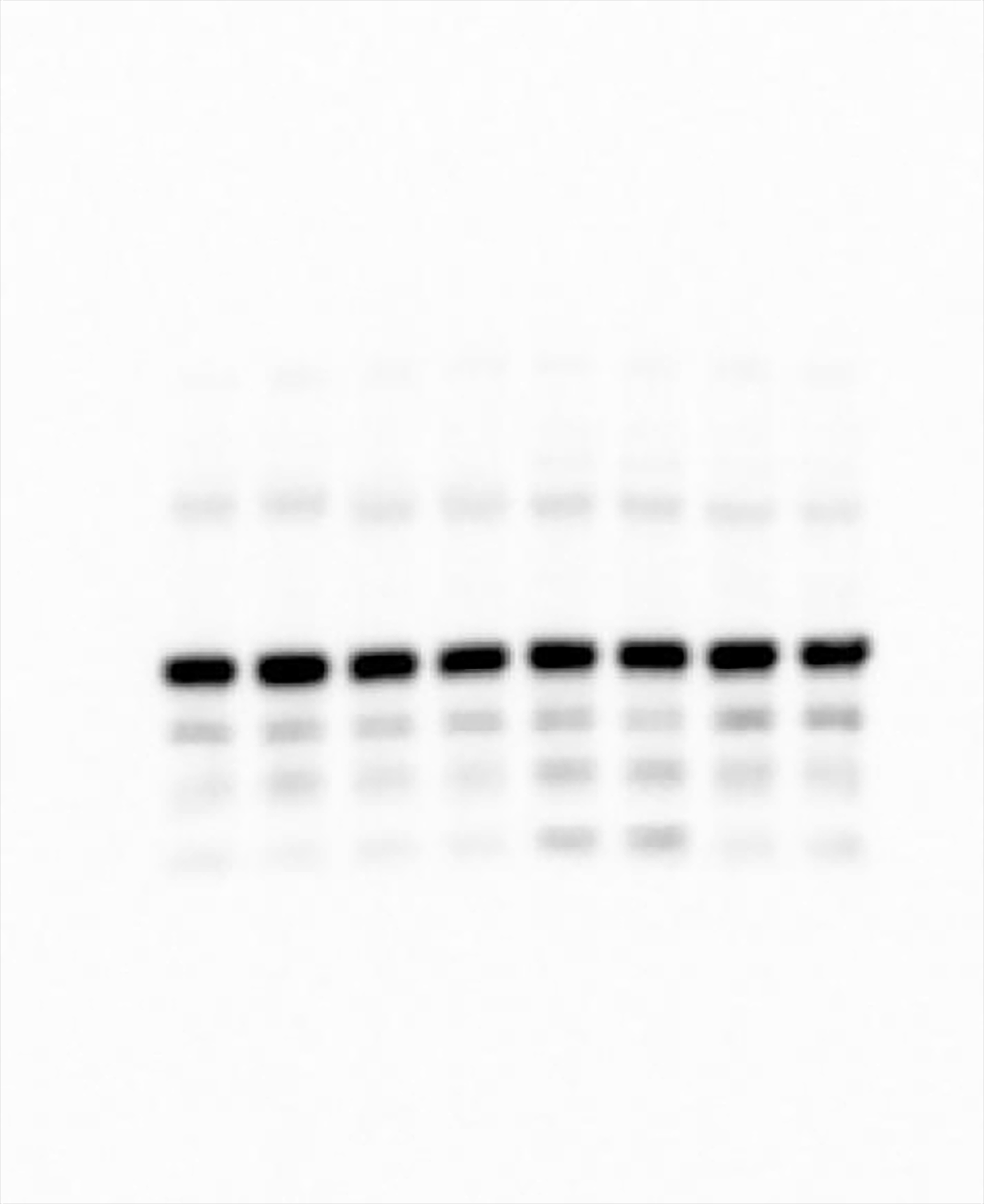

Supplement: Supplementary file 3 — Source data Fig. 1 [file 44321_2024_146_MOESM3_ESM.zip › Fig. 1/Fig. 1F/Fig. 1F-GAPDH.tif]

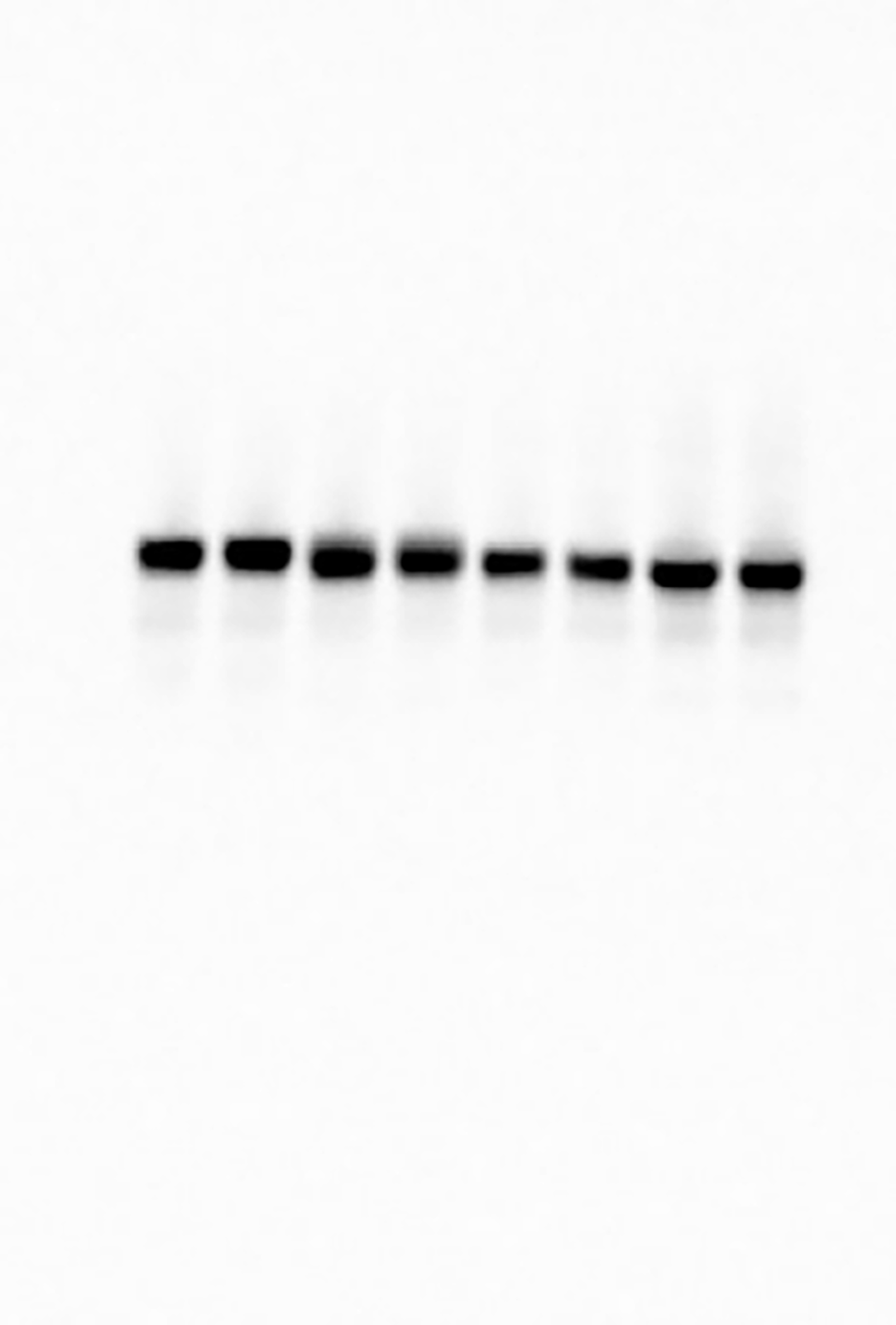

Supplement: Supplementary file 3 — Source data Fig. 1 [file 44321_2024_146_MOESM3_ESM.zip › Fig. 1/Fig. 1F/Fig. 1F-tau.tif]

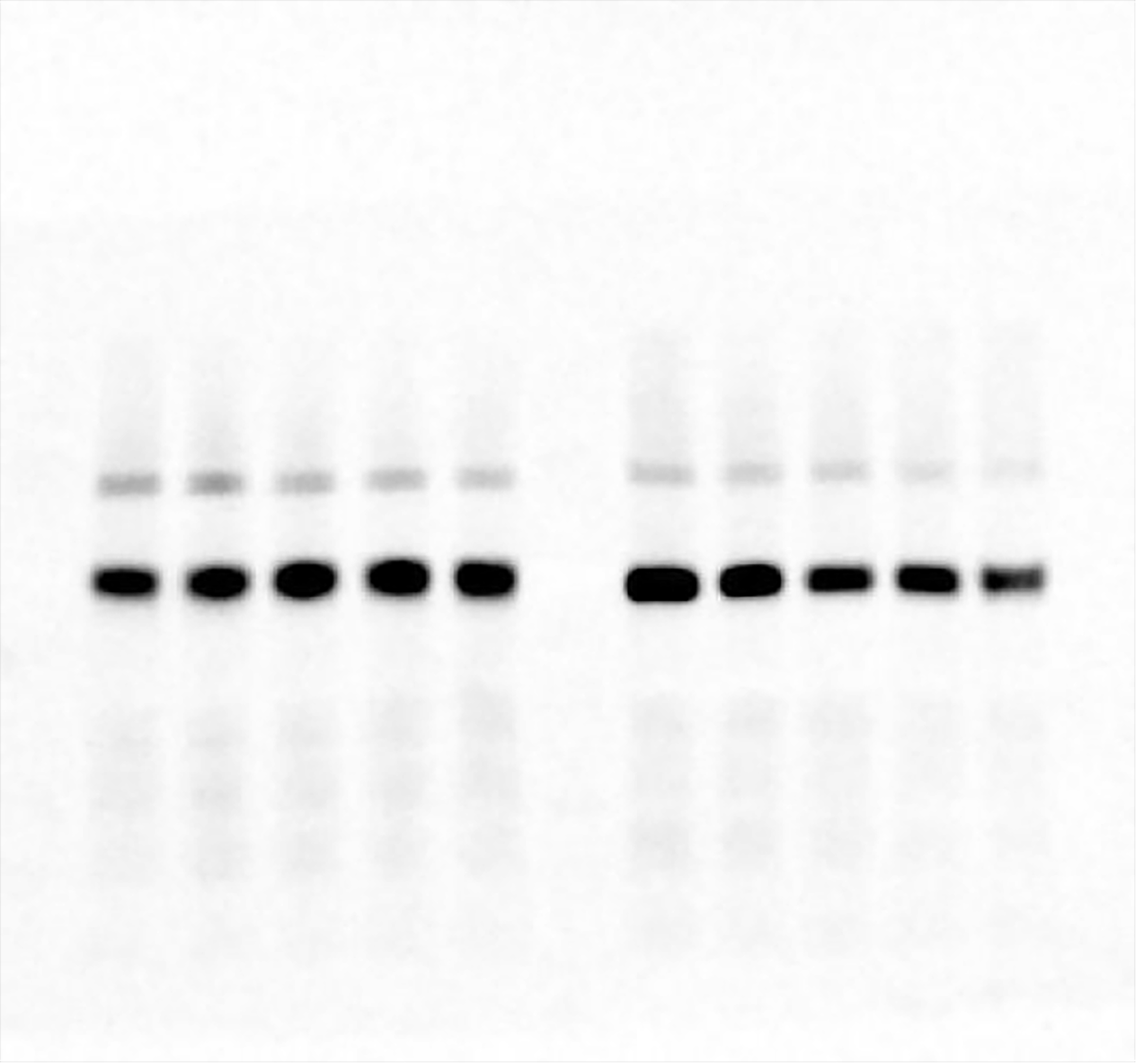

Supplement: Supplementary file 3 — Source data Fig. 1 [file 44321_2024_146_MOESM3_ESM.zip › Fig. 1/Fig. 1H/Fig. 1H-tau.tif]

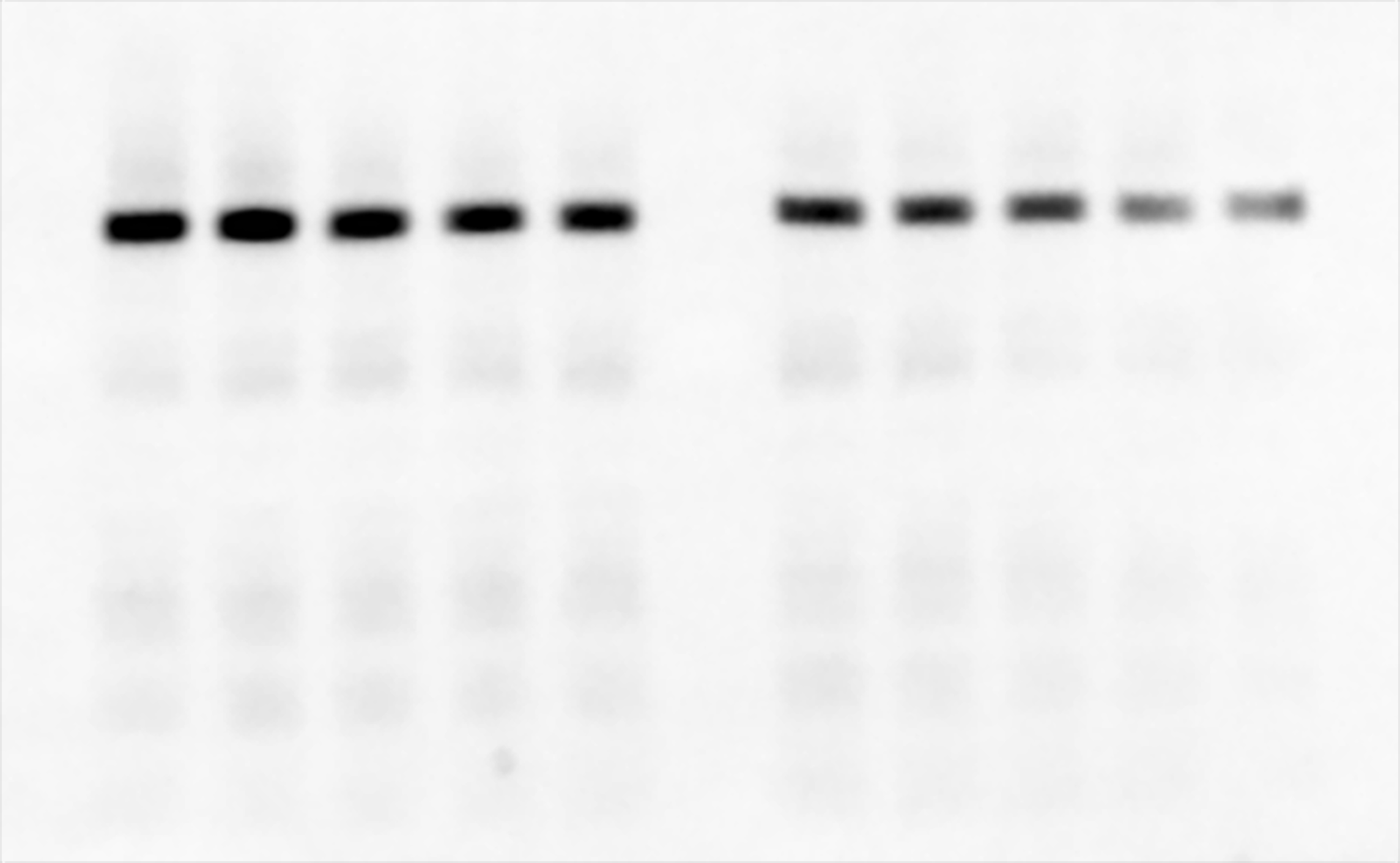

Supplement: Supplementary file 3 — Source data Fig. 1 [file 44321_2024_146_MOESM3_ESM.zip › Fig. 1/Fig. 1H/Fig. 1H-USP11.tif]

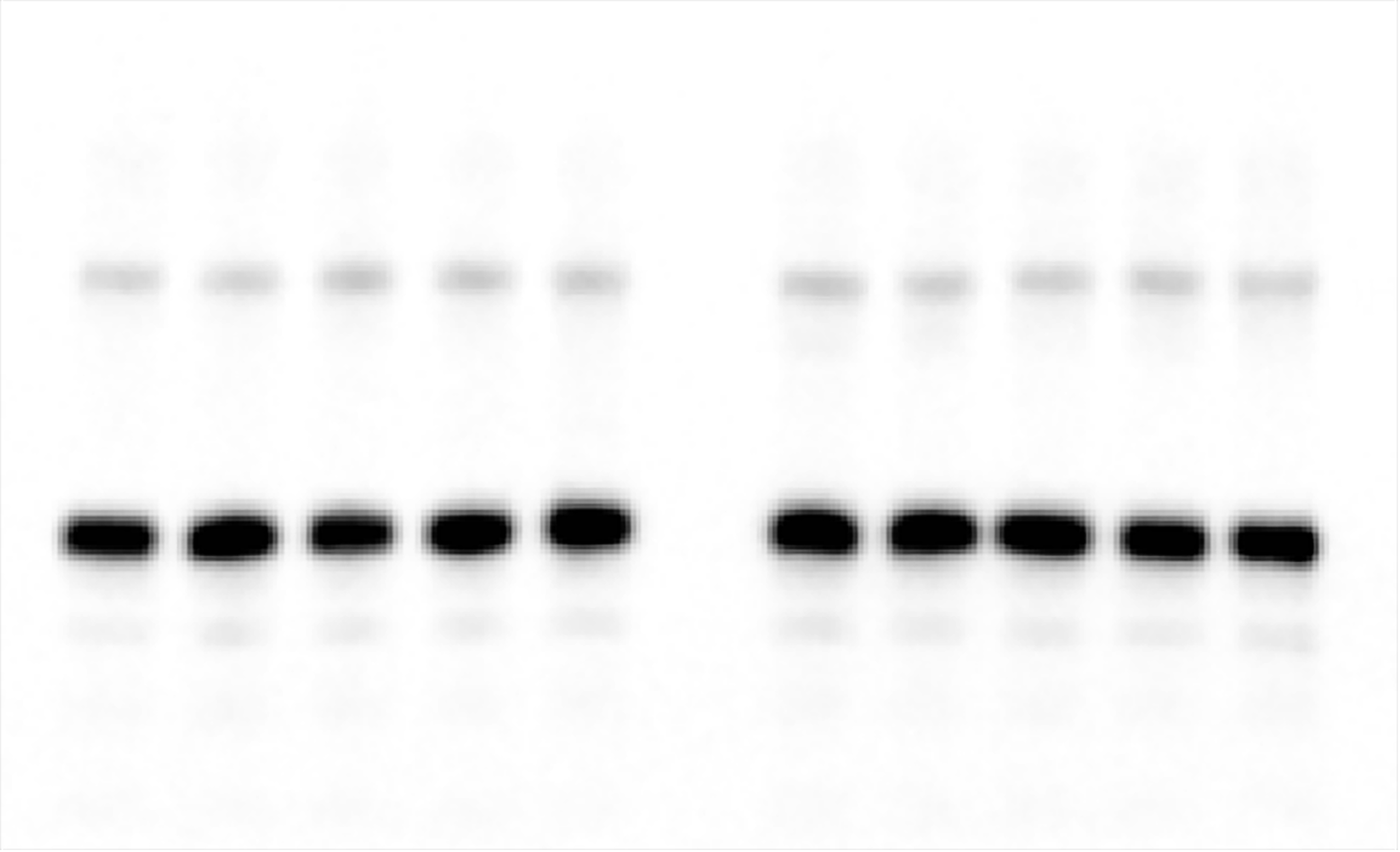

Supplement: Supplementary file 3 — Source data Fig. 1 [file 44321_2024_146_MOESM3_ESM.zip › Fig. 1/Fig. 1H/Fig. 1H-GAPDH.tif]

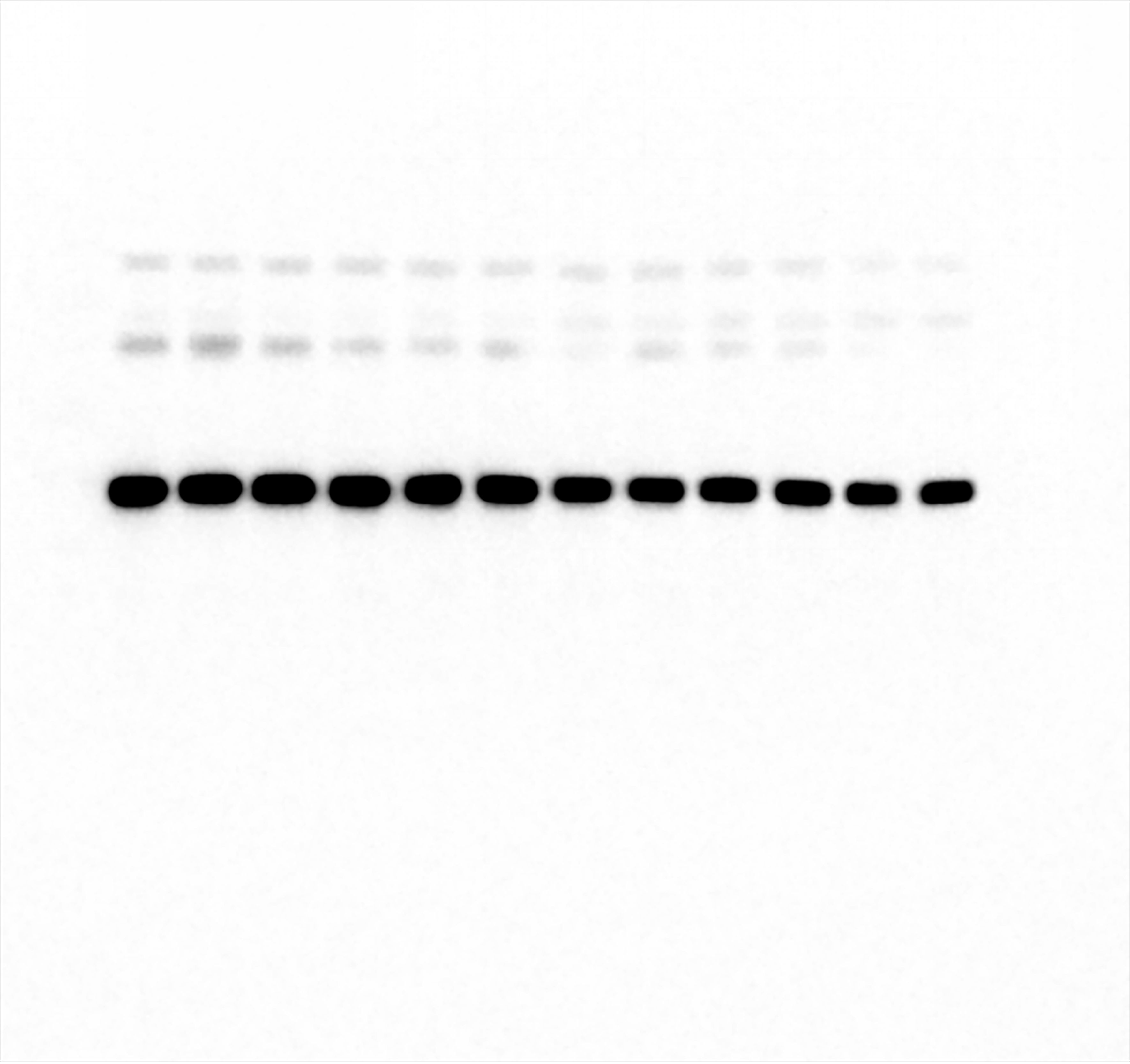

Supplement: Supplementary file 3 — Source data Fig. 1 [file 44321_2024_146_MOESM3_ESM.zip › Fig. 1/Fig. 1J/Fig. 1J-USP11-GAPDH-Time.tif]

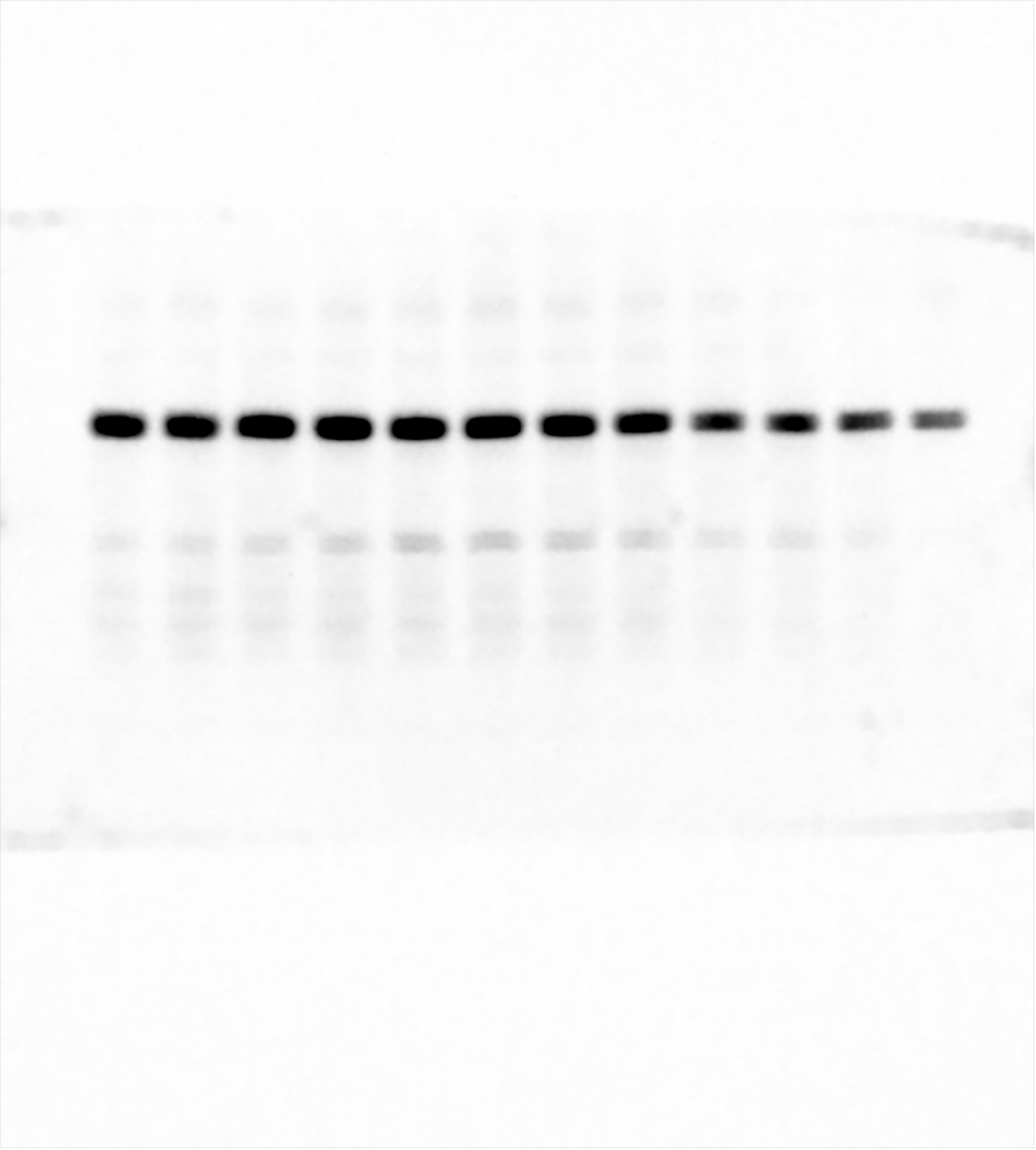

Supplement: Supplementary file 3 — Source data Fig. 1 [file 44321_2024_146_MOESM3_ESM.zip › Fig. 1/Fig. 1J/Fig. 1J-p-tau231-Time.tif]

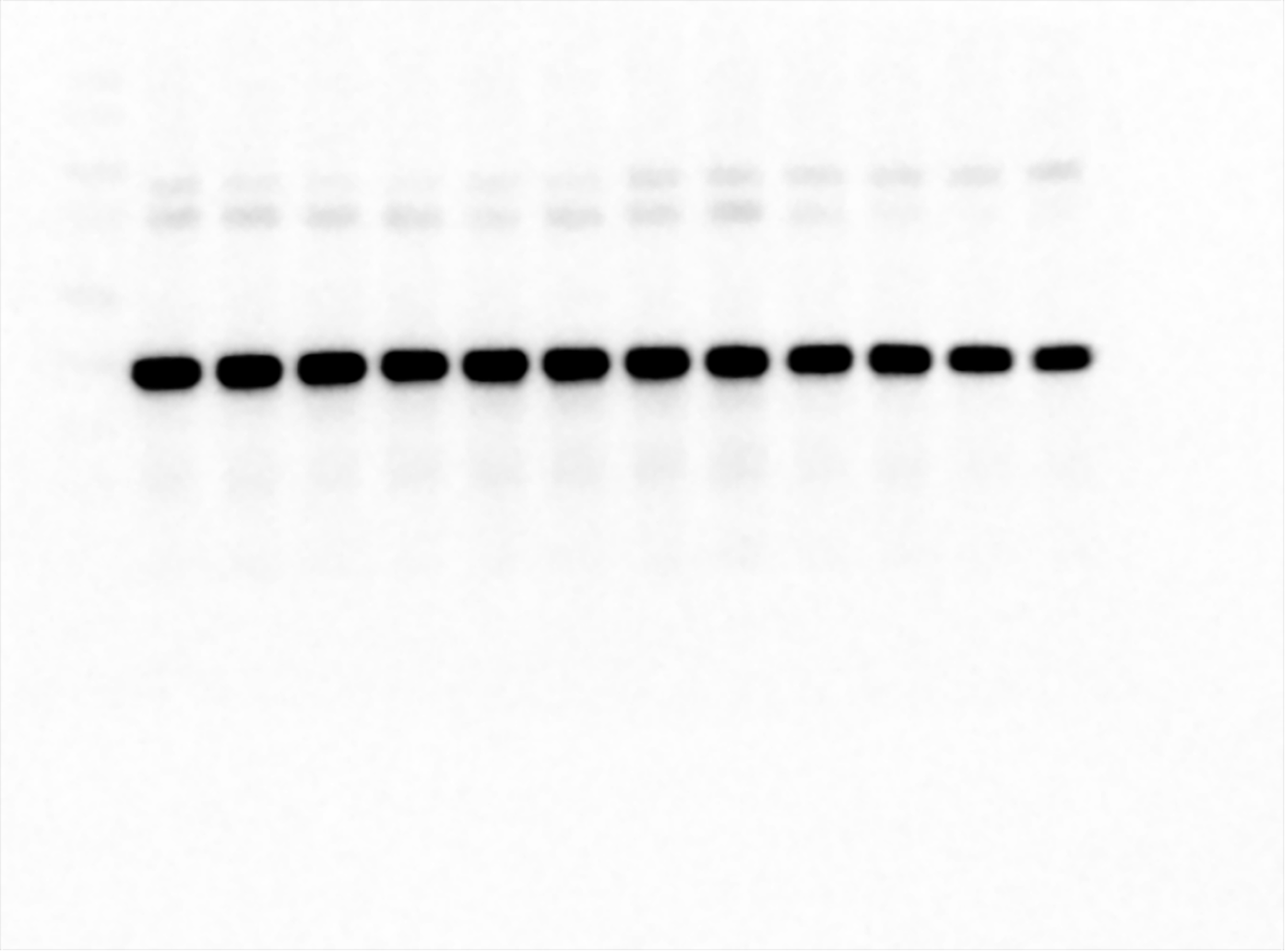

Supplement: Supplementary file 3 — Source data Fig. 1 [file 44321_2024_146_MOESM3_ESM.zip › Fig. 1/Fig. 1J/Fig. 1J-p-tau-181-GAPDH-Time.tif]

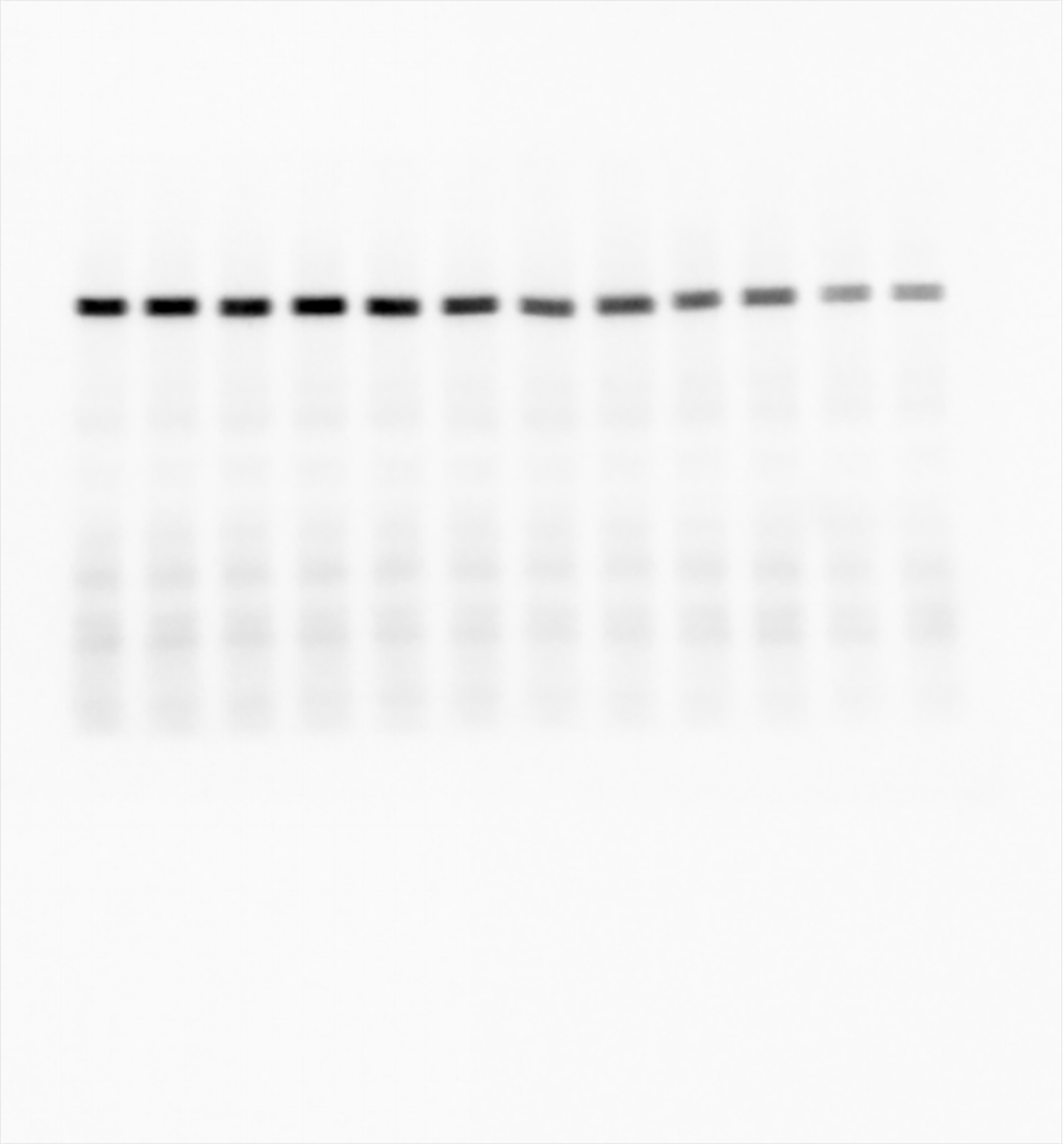

Supplement: Supplementary file 3 — Source data Fig. 1 [file 44321_2024_146_MOESM3_ESM.zip › Fig. 1/Fig. 1J/Fig. 1J-USP11-Time.tif]

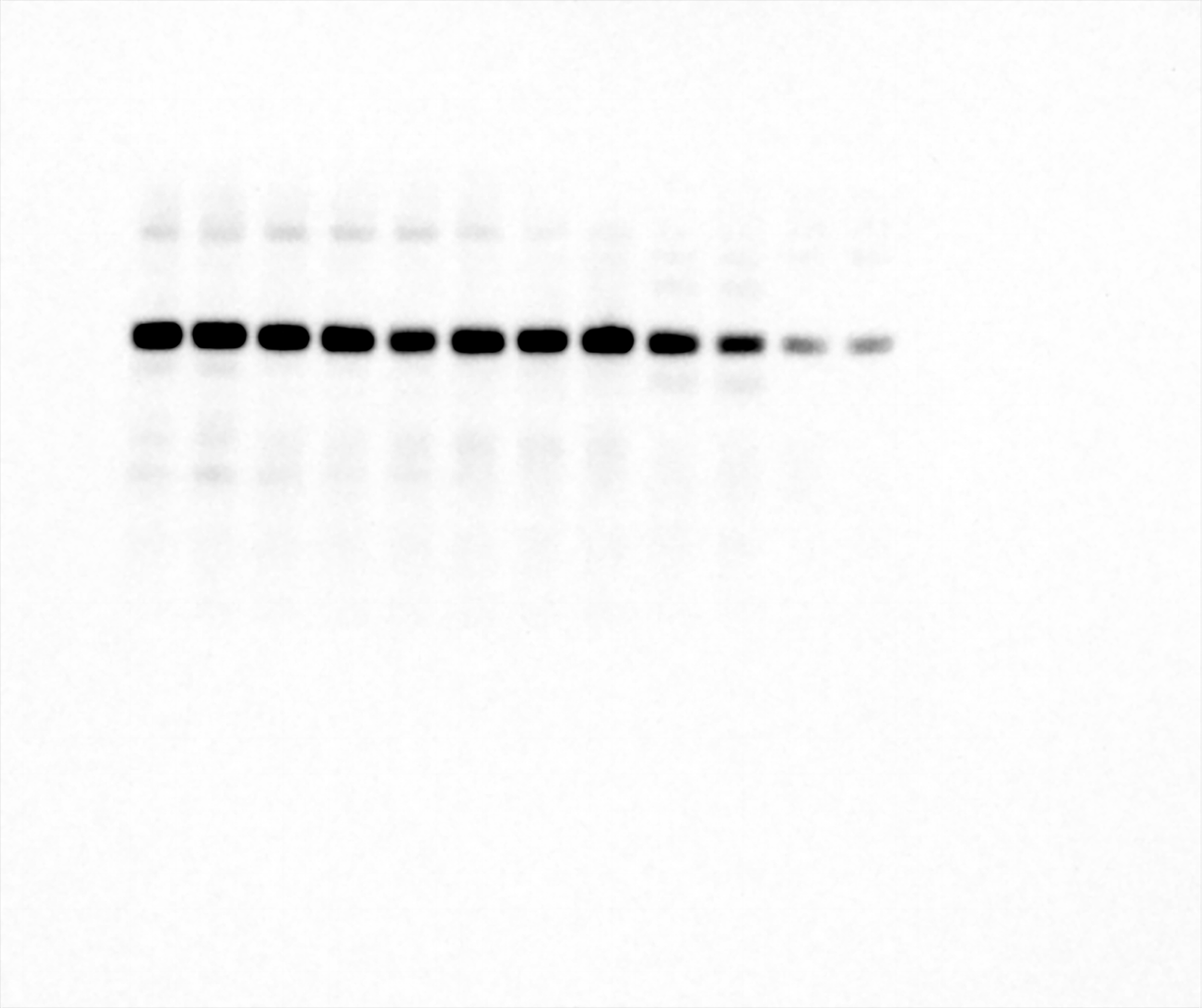

Supplement: Supplementary file 3 — Source data Fig. 1 [file 44321_2024_146_MOESM3_ESM.zip › Fig. 1/Fig. 1J/Fig. 1J-p-tau-181-Time.tif]

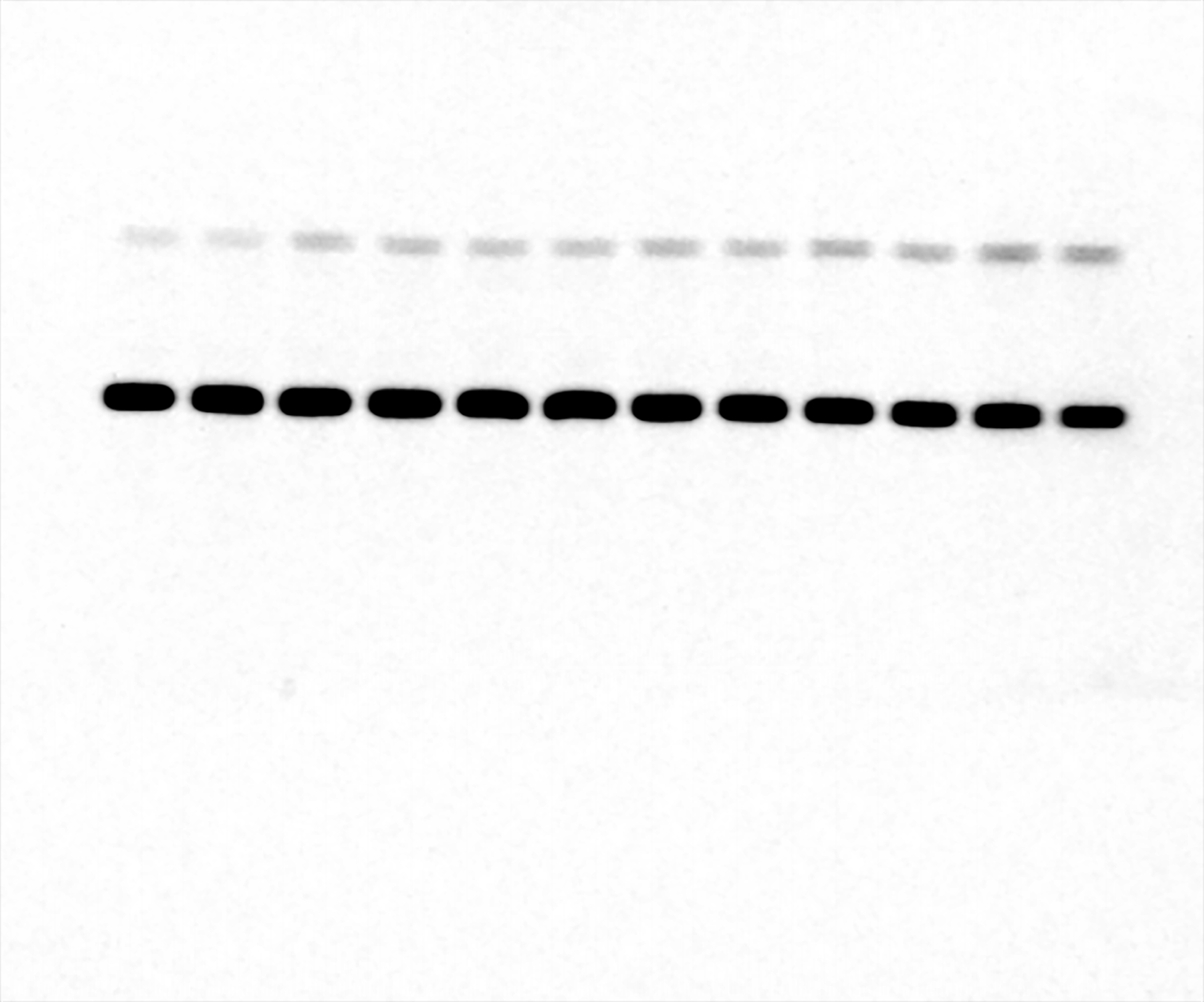

Supplement: Supplementary file 3 — Source data Fig. 1 [file 44321_2024_146_MOESM3_ESM.zip › Fig. 1/Fig. 1J/Fig. 1J-total-tau-GAPDH-Time.tif]

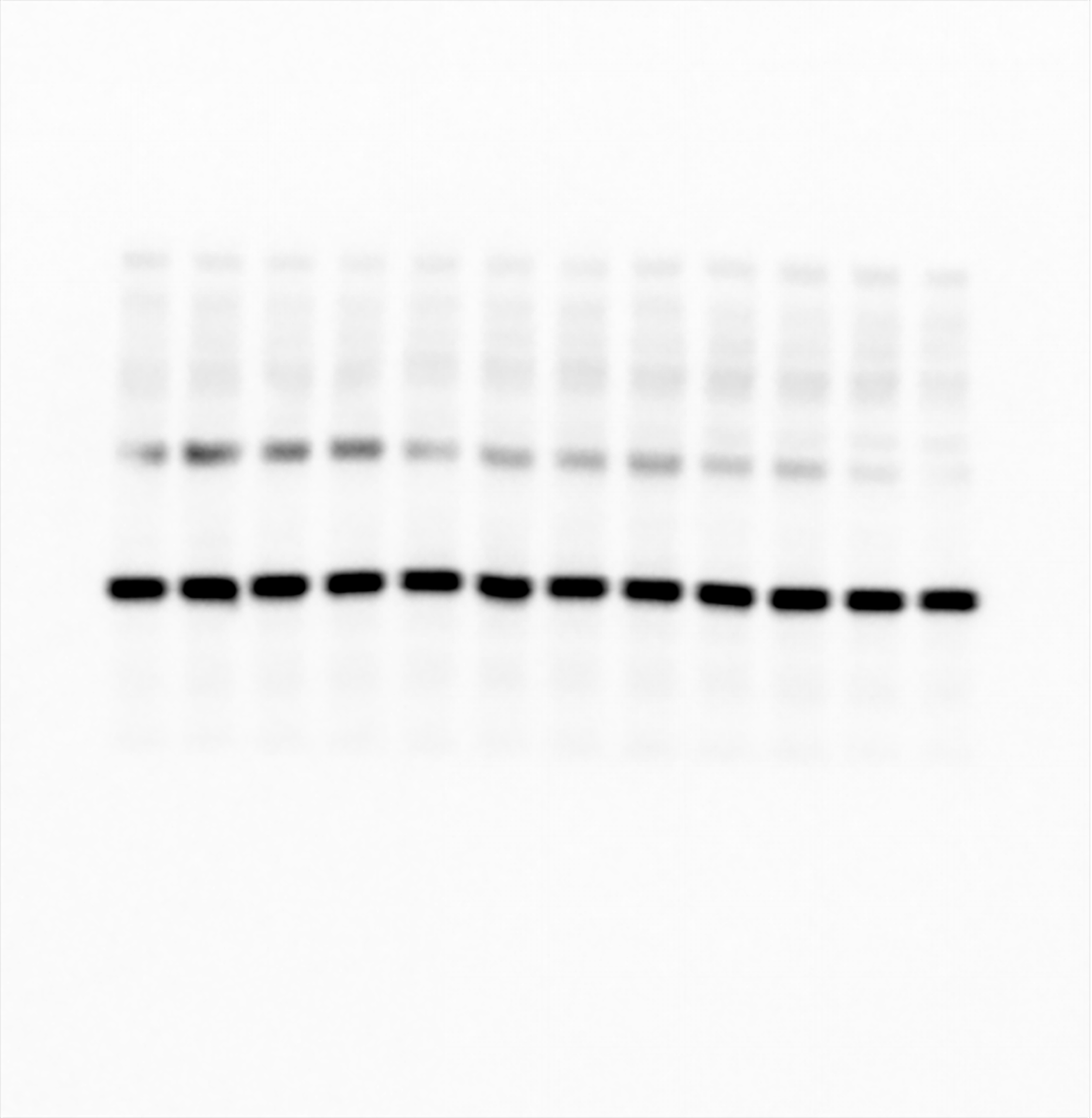

Supplement: Supplementary file 3 — Source data Fig. 1 [file 44321_2024_146_MOESM3_ESM.zip › Fig. 1/Fig. 1J/Fig. 1J-p-tau396-GAPDH-Time.tif]

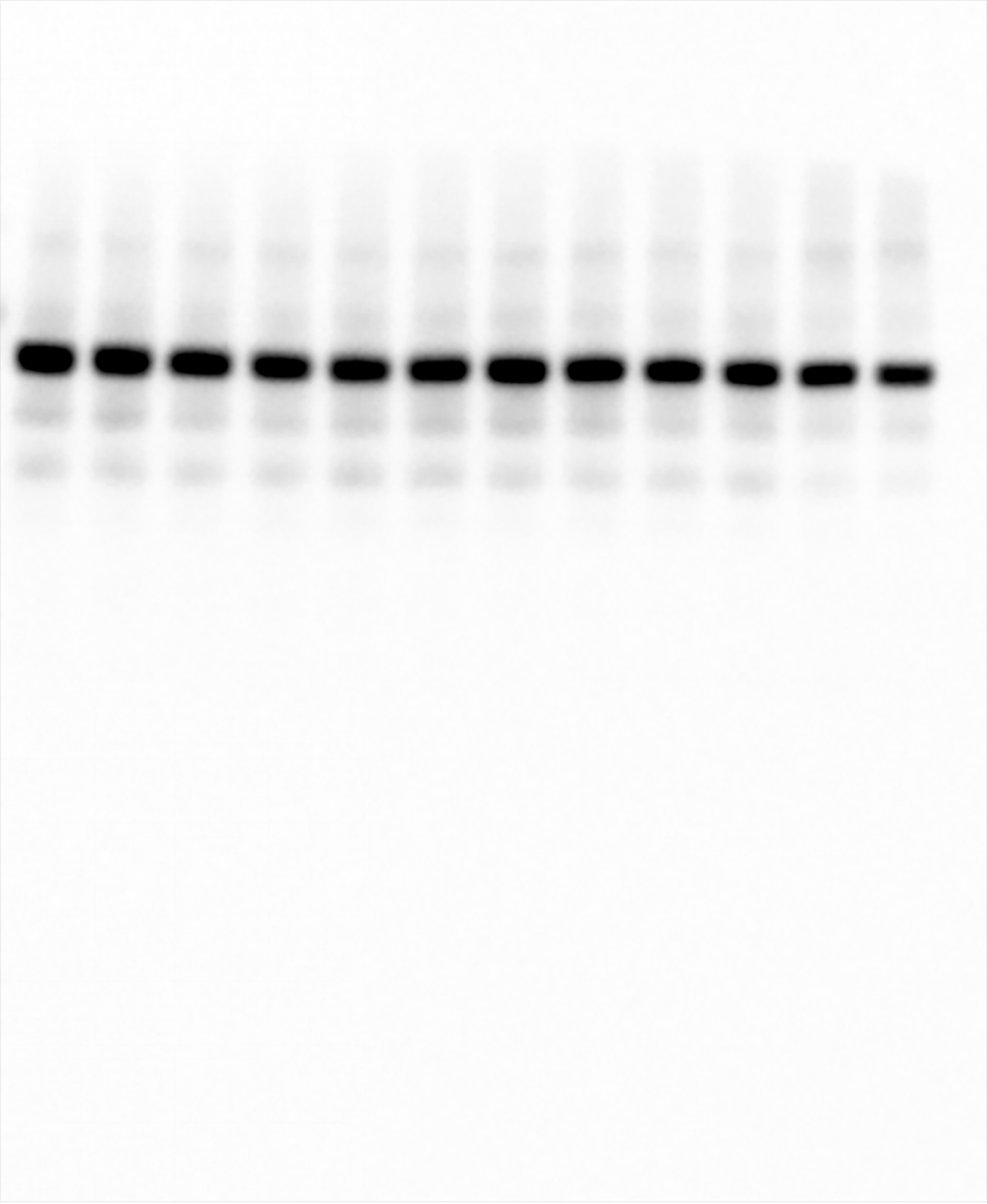

Supplement: Supplementary file 3 — Source data Fig. 1 [file 44321_2024_146_MOESM3_ESM.zip › Fig. 1/Fig. 1J/Fig. 1J-total-tau-Time.tif]

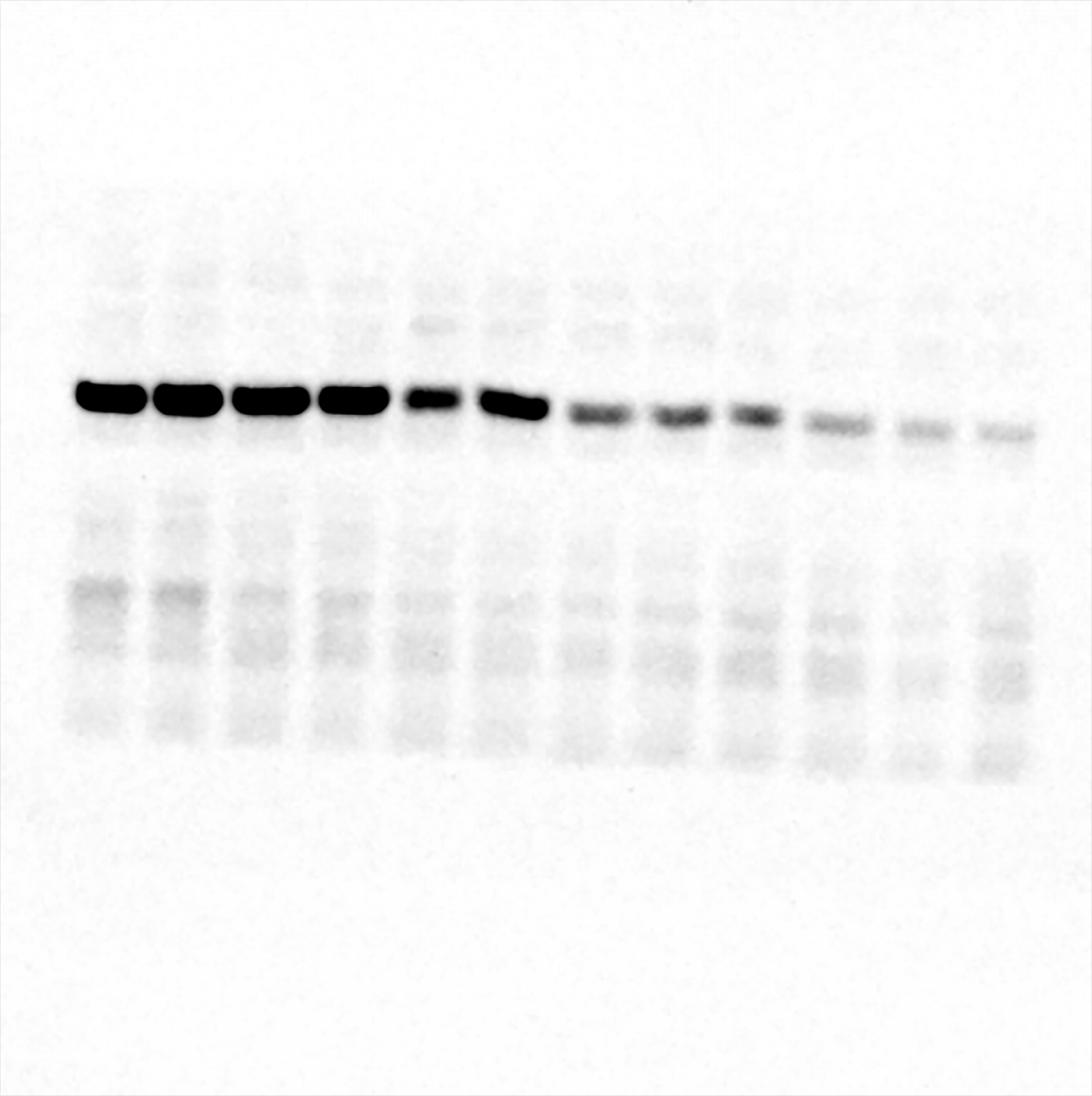

Supplement: Supplementary file 3 — Source data Fig. 1 [file 44321_2024_146_MOESM3_ESM.zip › Fig. 1/Fig. 1J/Fig. 1J-p-tau396-Time.tif]

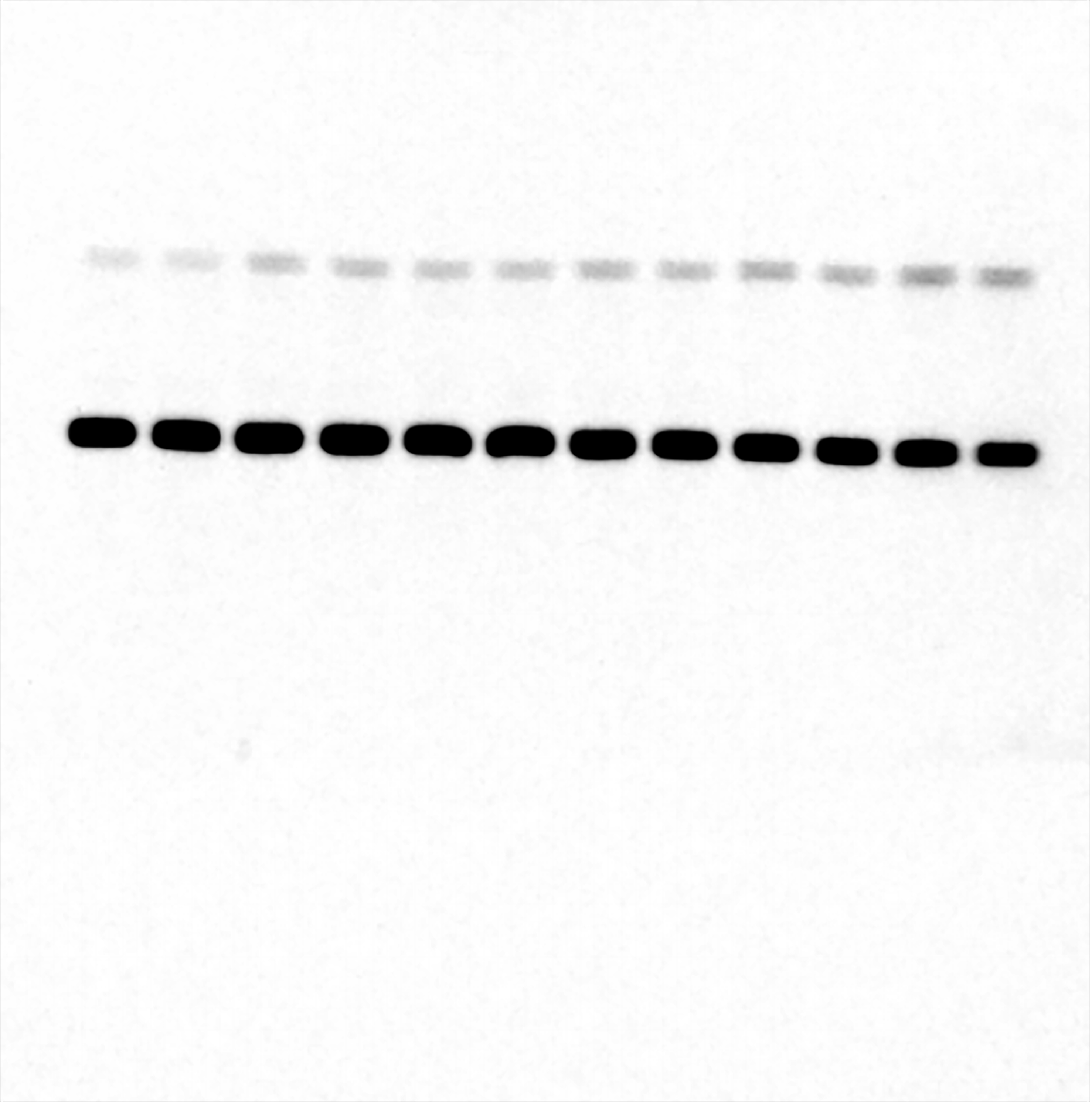

Supplement: Supplementary file 3 — Source data Fig. 1 [file 44321_2024_146_MOESM3_ESM.zip › Fig. 1/Fig. 1J/Fig. 1J-p-tau231-GAPDH-Time.tif]

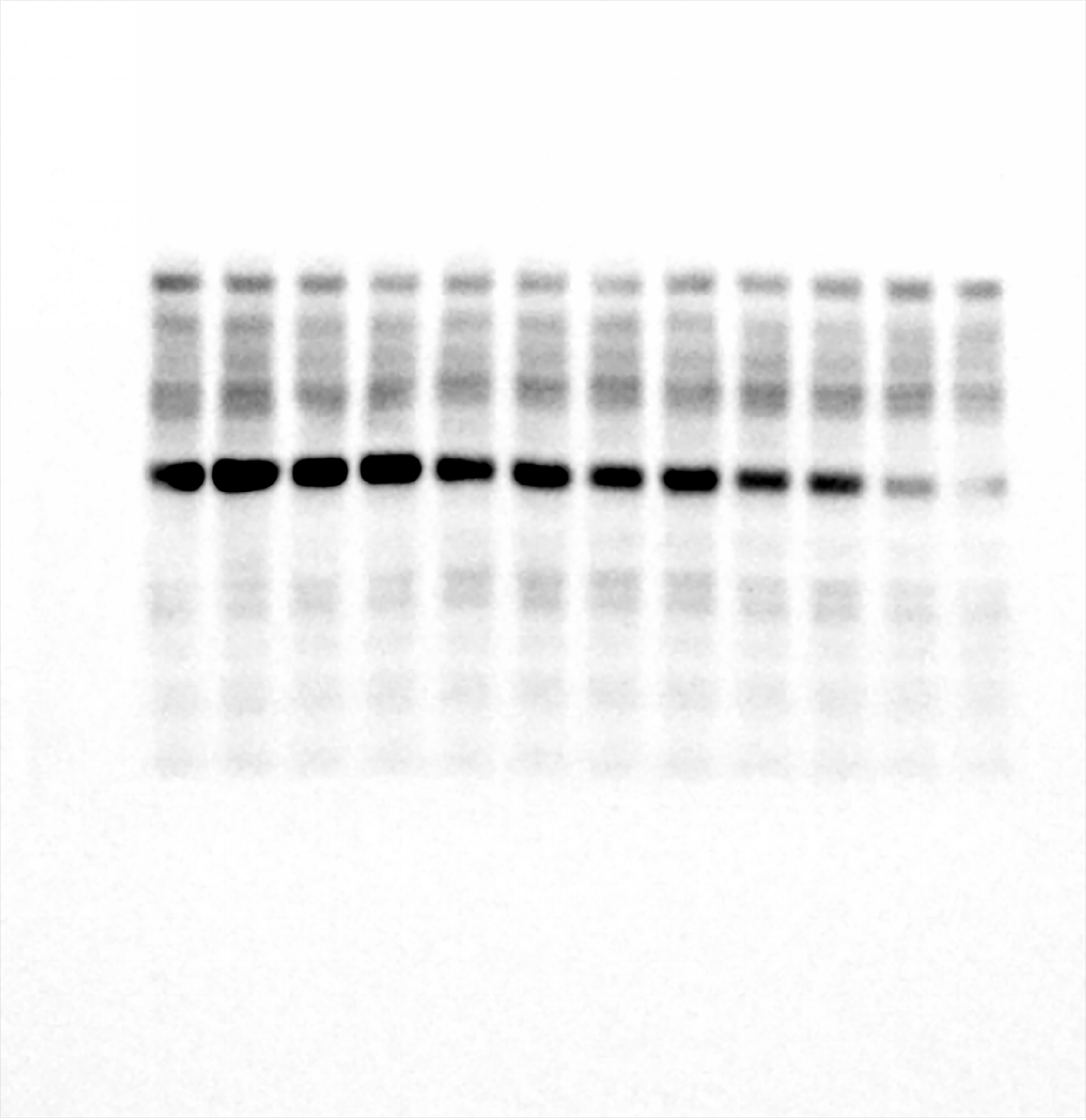

Supplement: Supplementary file 3 — Source data Fig. 1 [file 44321_2024_146_MOESM3_ESM.zip › Fig. 1/Fig. 1J/Fig. 1J-p-tau202-205-Time.tif]

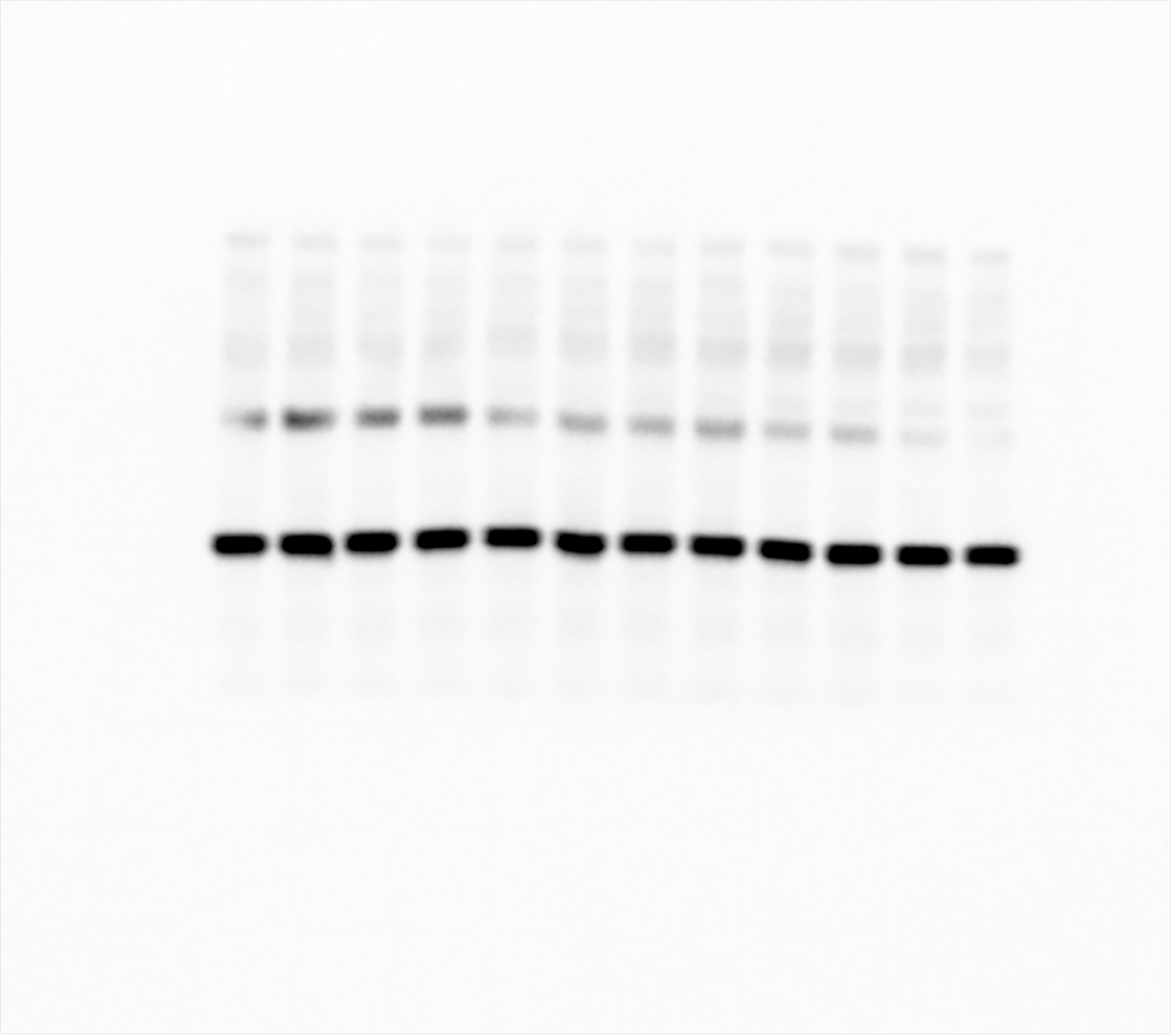

Supplement: Supplementary file 3 — Source data Fig. 1 [file 44321_2024_146_MOESM3_ESM.zip › Fig. 1/Fig. 1J/Fig. 1J-p-tau202-205-GAPDHTime.tif]

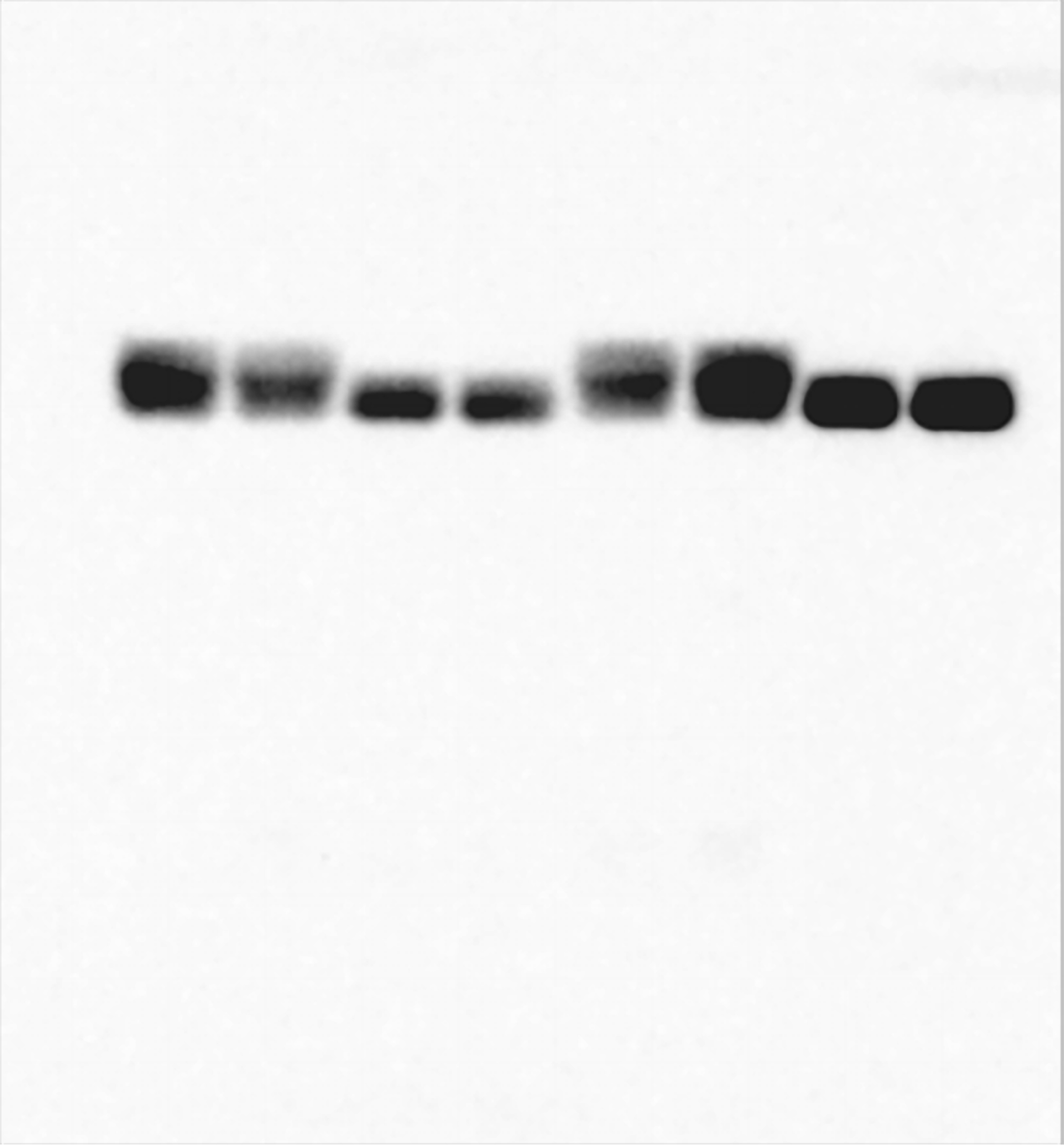

Supplement: Supplementary file 3 — Source data Fig. 1 [file 44321_2024_146_MOESM3_ESM.zip › Fig. 1/Fig. 1C/Fig. 1C-total-tau.tif]

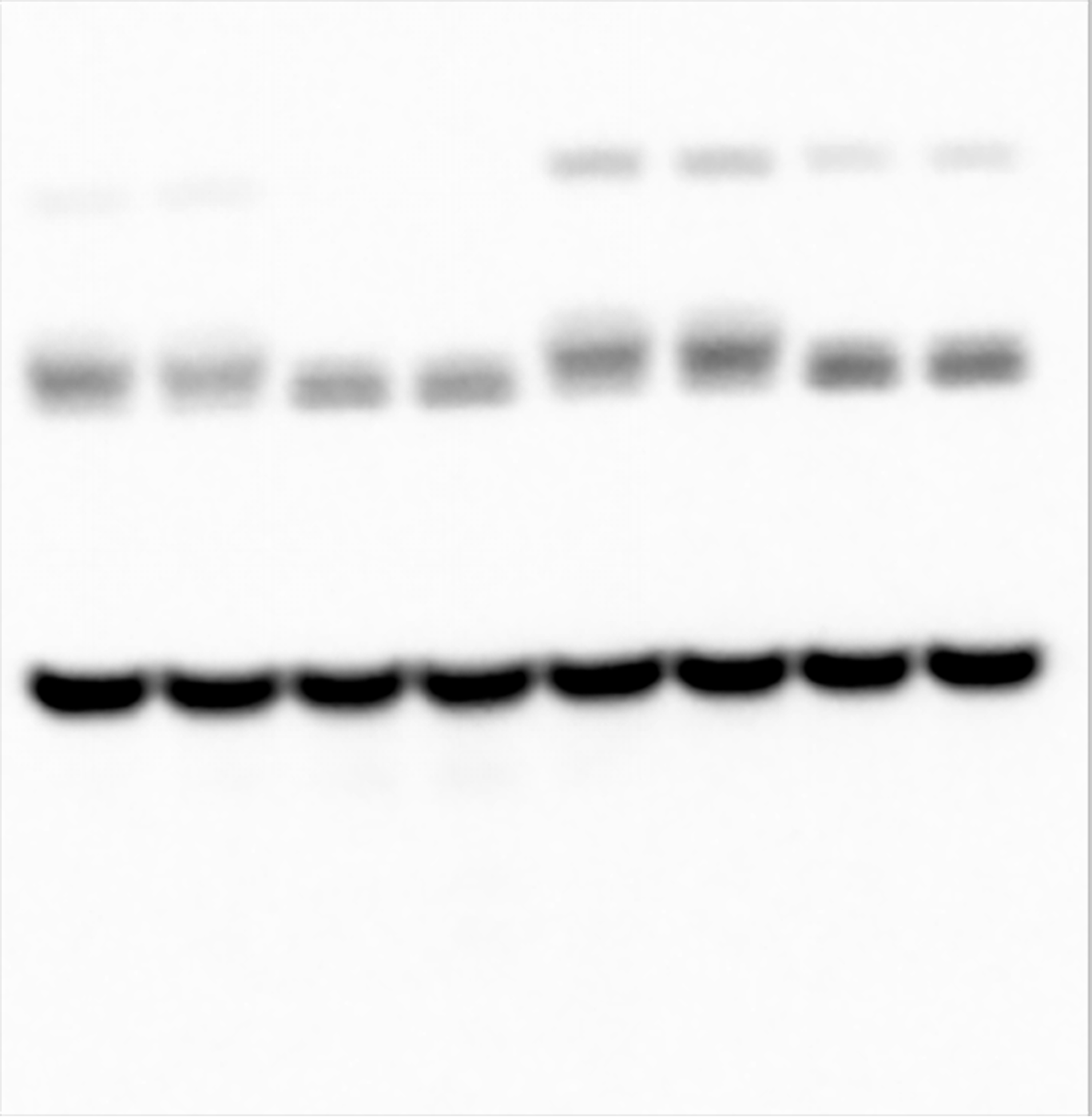

Supplement: Supplementary file 3 — Source data Fig. 1 [file 44321_2024_146_MOESM3_ESM.zip › Fig. 1/Fig. 1C/Fig. 1C-GAPDH.tif]

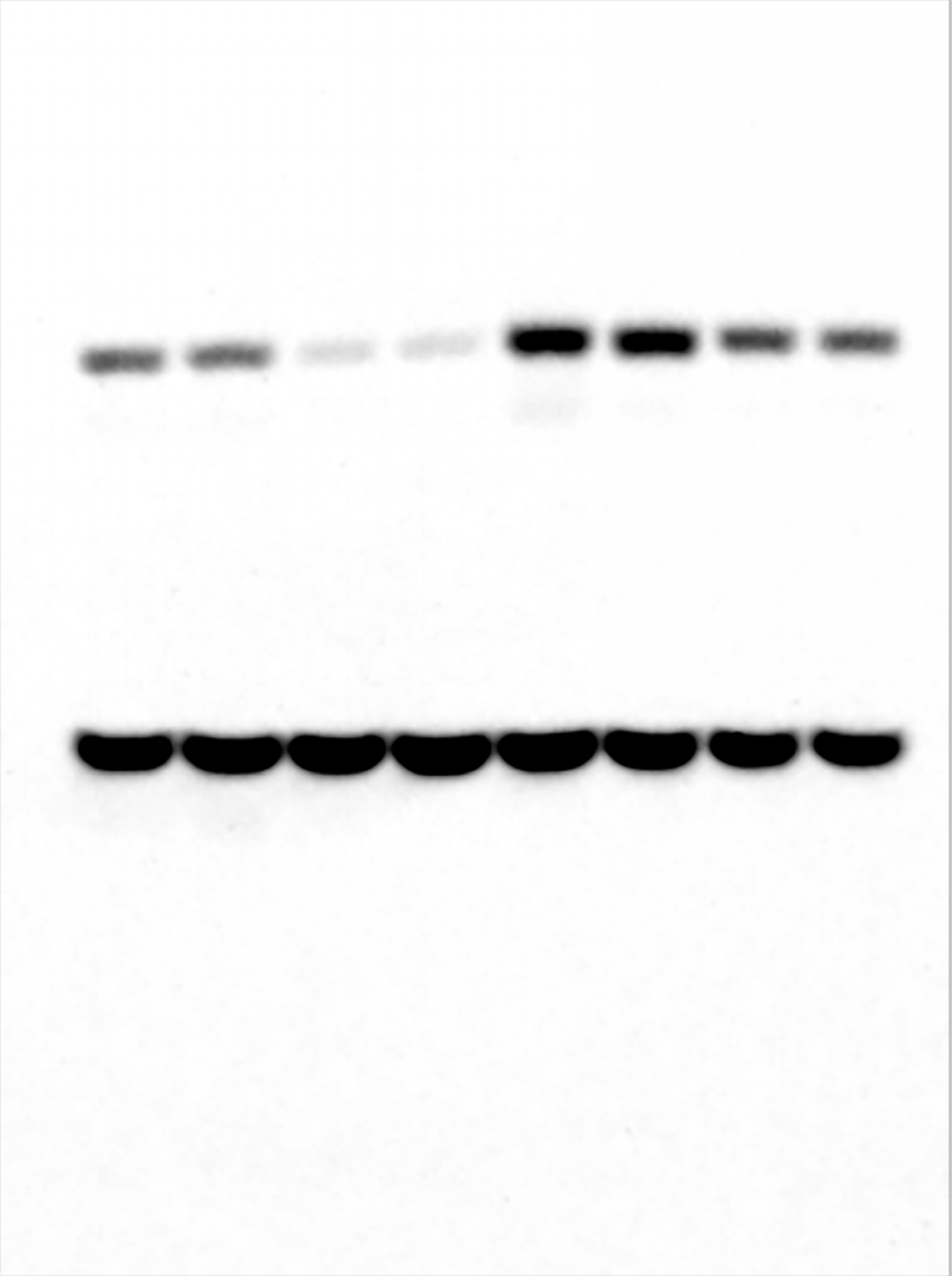

Supplement: Supplementary file 3 — Source data Fig. 1 [file 44321_2024_146_MOESM3_ESM.zip › Fig. 1/Fig. 1C/Fig. 2C-USP11.tif]

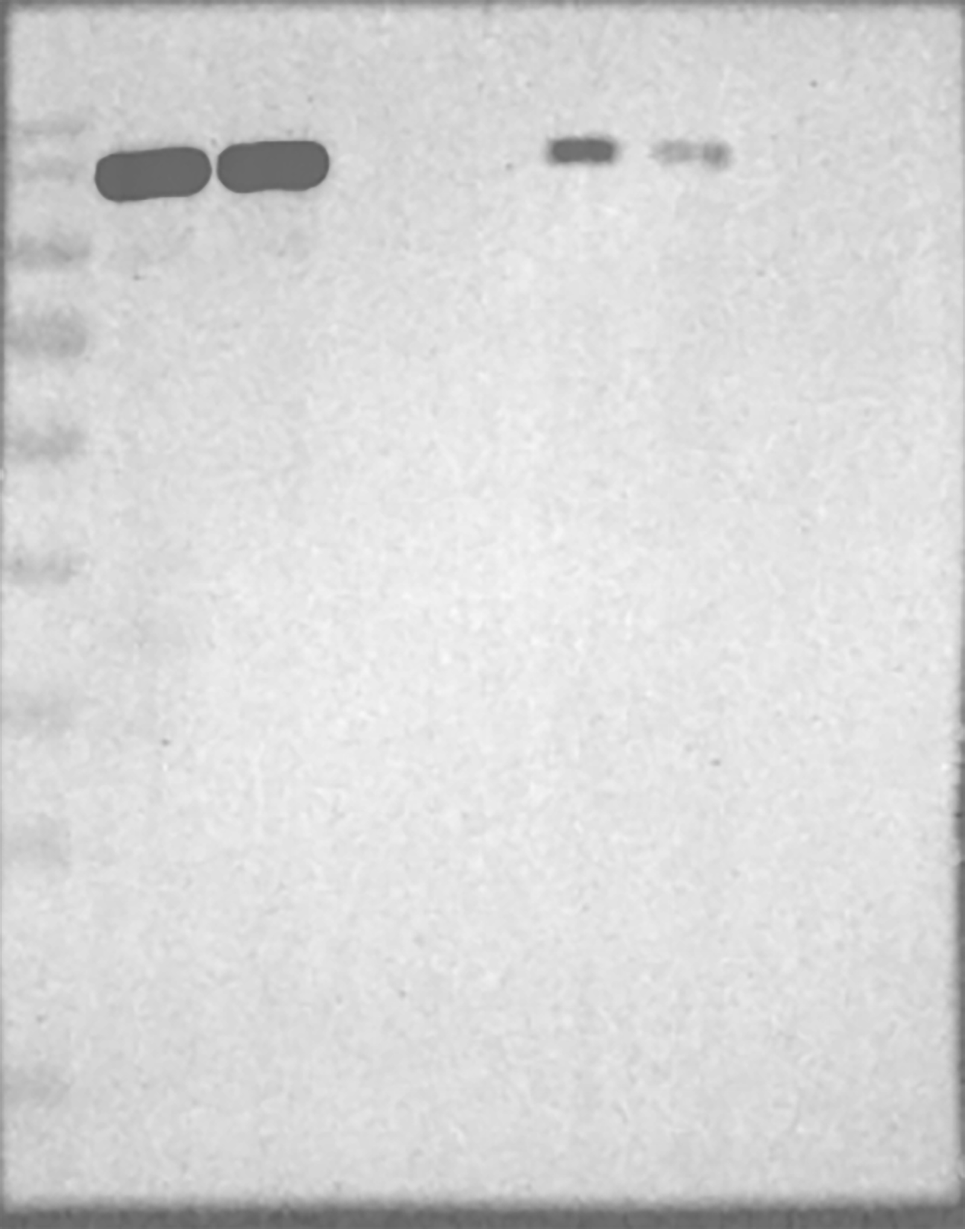

Supplement: Supplementary file 3 — Source data Fig. 1 [file 44321_2024_146_MOESM3_ESM.zip › Fig. 1/Fig. 1E/Fig. 2E-USP11.tif]

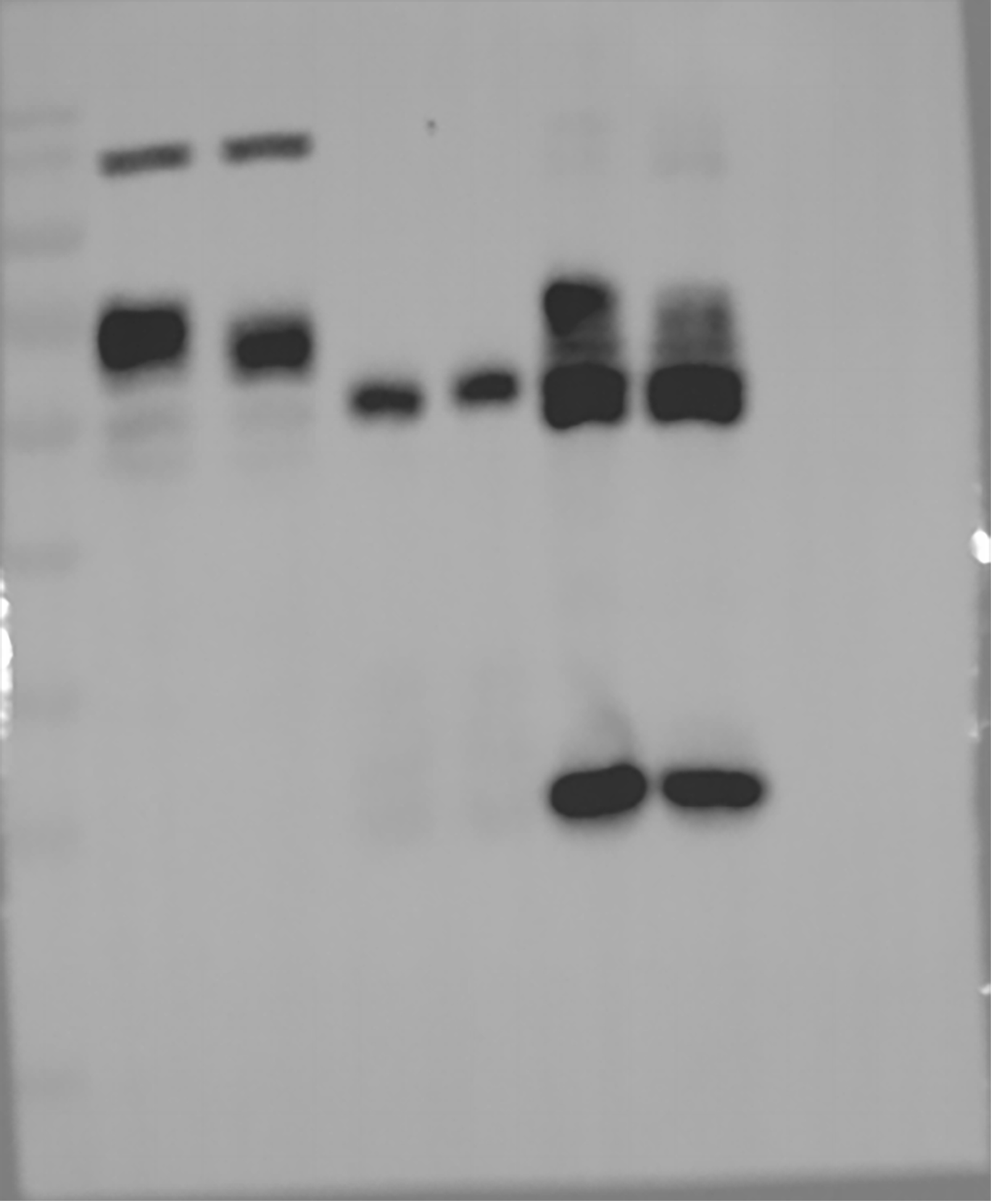

Supplement: Supplementary file 3 — Source data Fig. 1 [file 44321_2024_146_MOESM3_ESM.zip › Fig. 1/Fig. 1E/Fig. 1E-tau.tif]

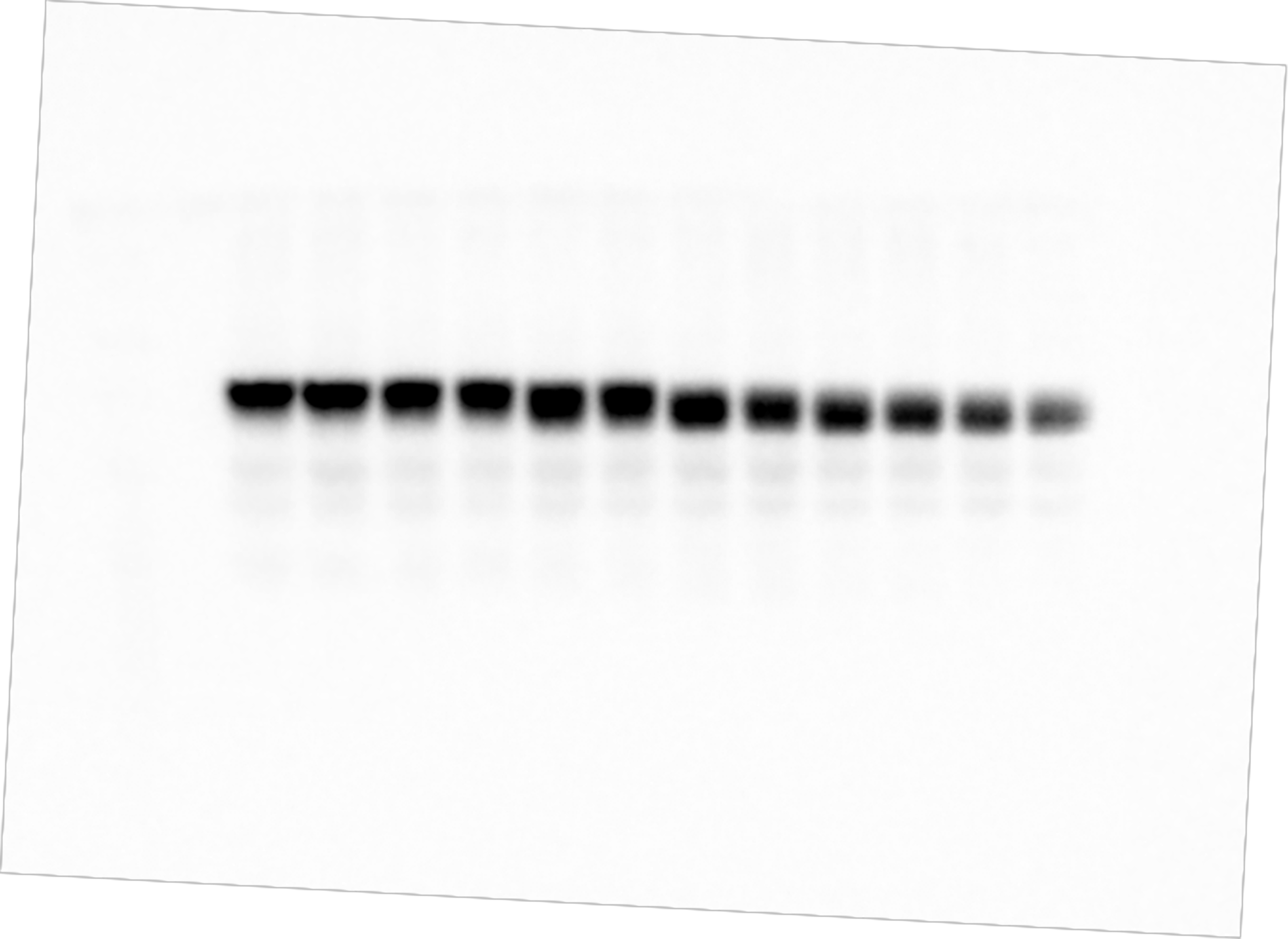

Supplement: Supplementary file 3 — Source data Fig. 1 [file 44321_2024_146_MOESM3_ESM.zip › Fig. 1/Fig. 1K/Fig. 1K-total-tau-concentration.tif]

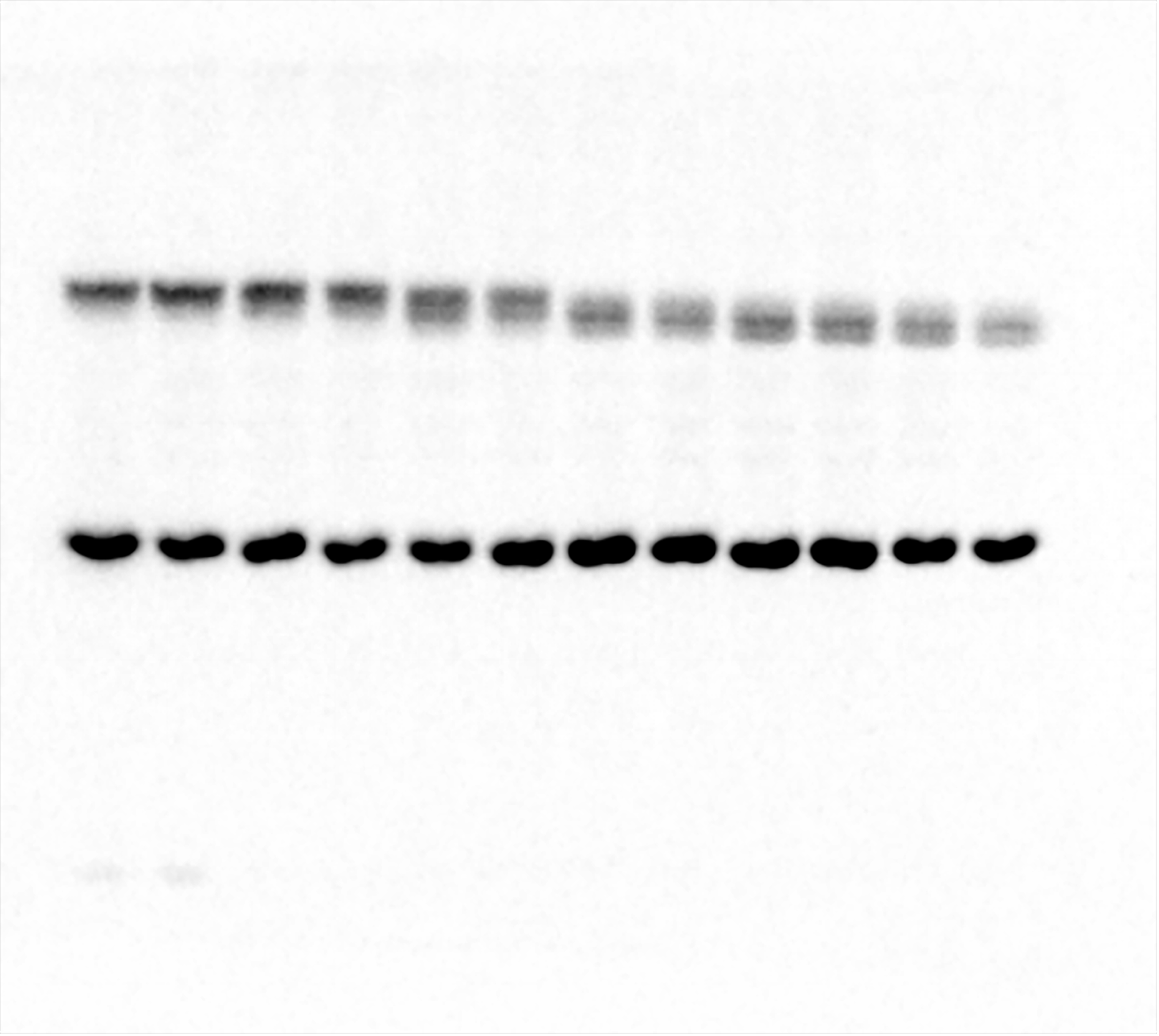

Supplement: Supplementary file 3 — Source data Fig. 1 [file 44321_2024_146_MOESM3_ESM.zip › Fig. 1/Fig. 1K/Fig. 1K-total-tau-GAPDH-concentration.tif]

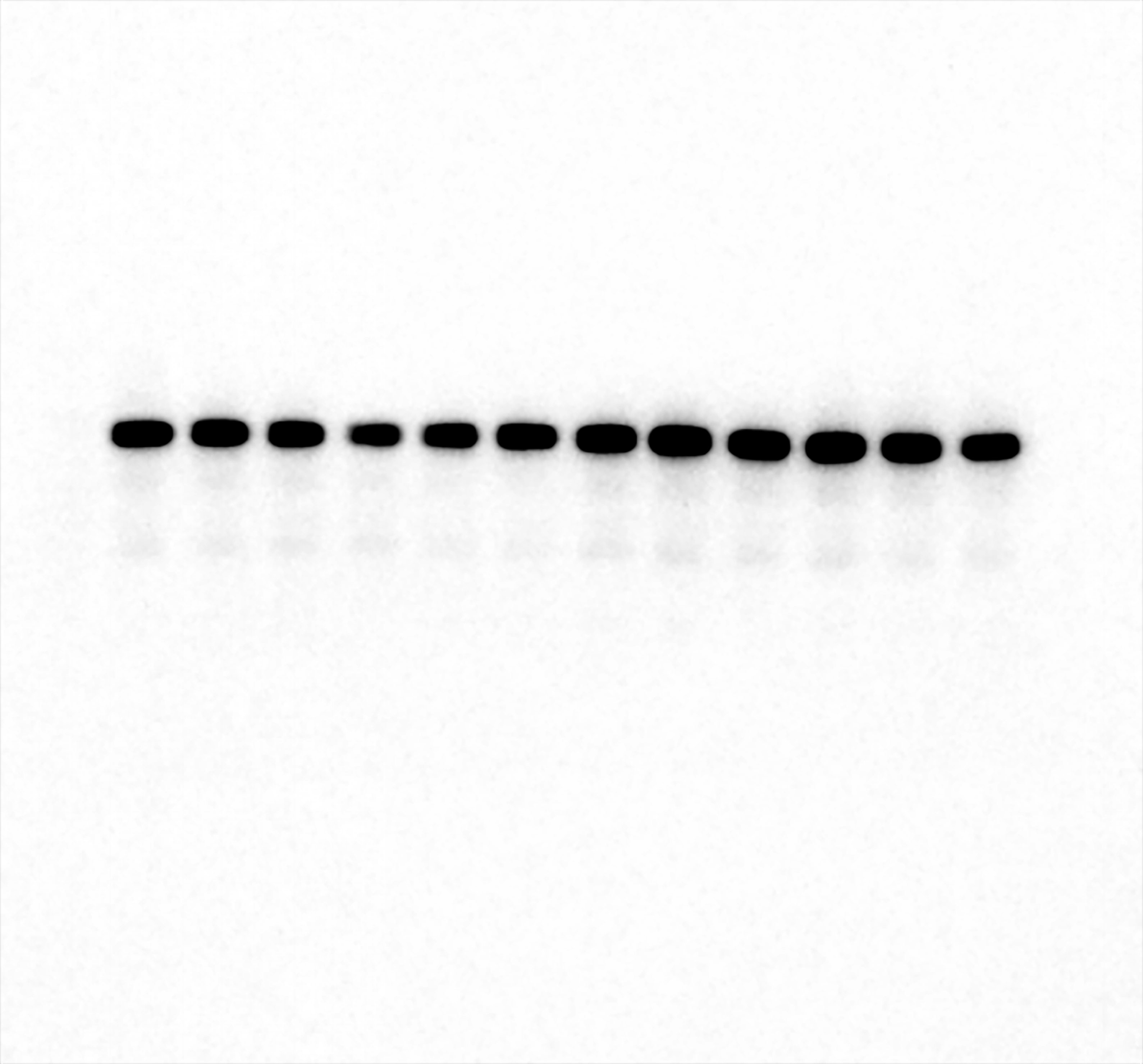

Supplement: Supplementary file 3 — Source data Fig. 1 [file 44321_2024_146_MOESM3_ESM.zip › Fig. 1/Fig. 1K/Fig. 1K-USP11-GAPDH-concentration.tif]

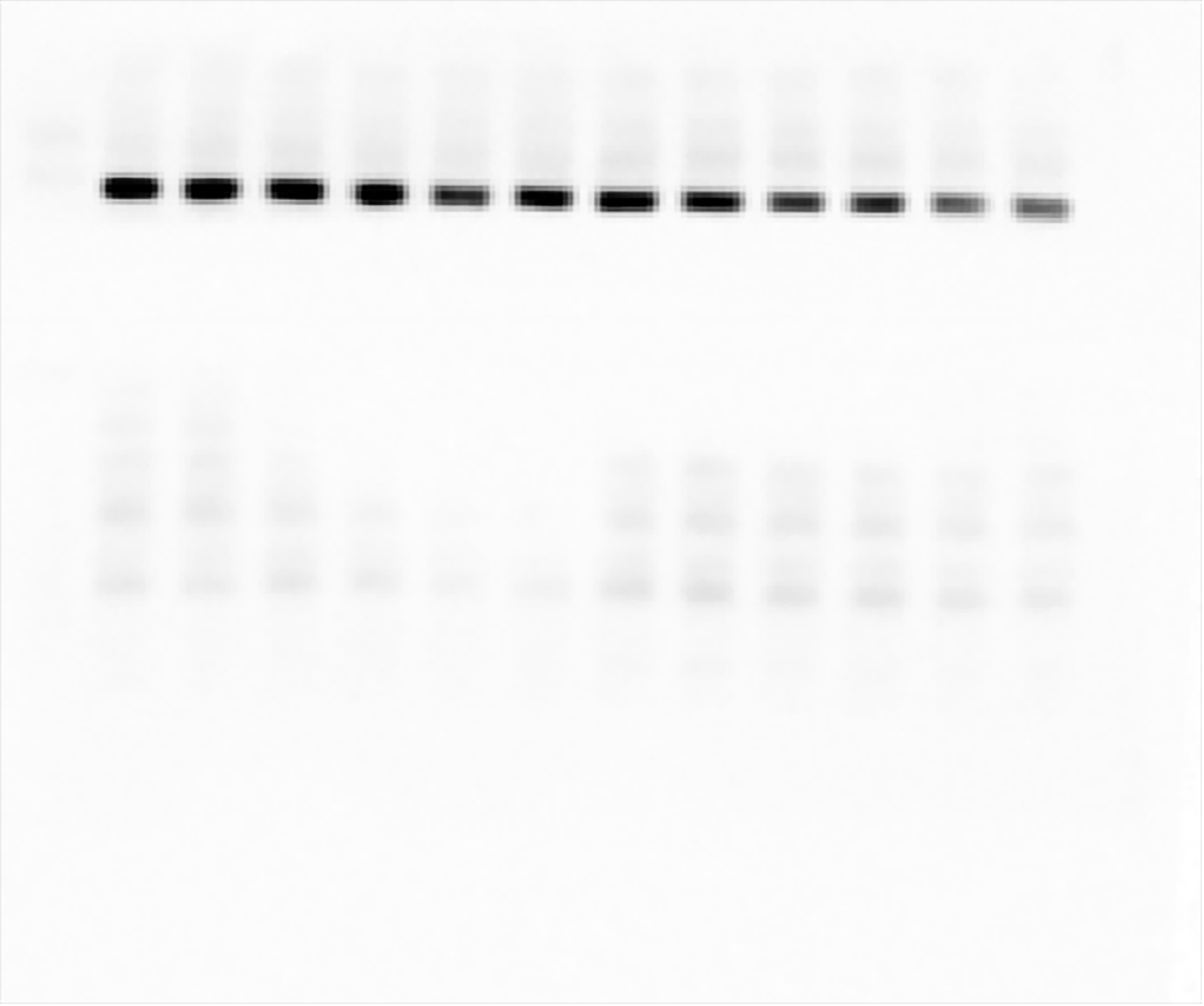

Supplement: Supplementary file 3 — Source data Fig. 1 [file 44321_2024_146_MOESM3_ESM.zip › Fig. 1/Fig. 1K/Fig. 1K-USP11-concentration.tif]

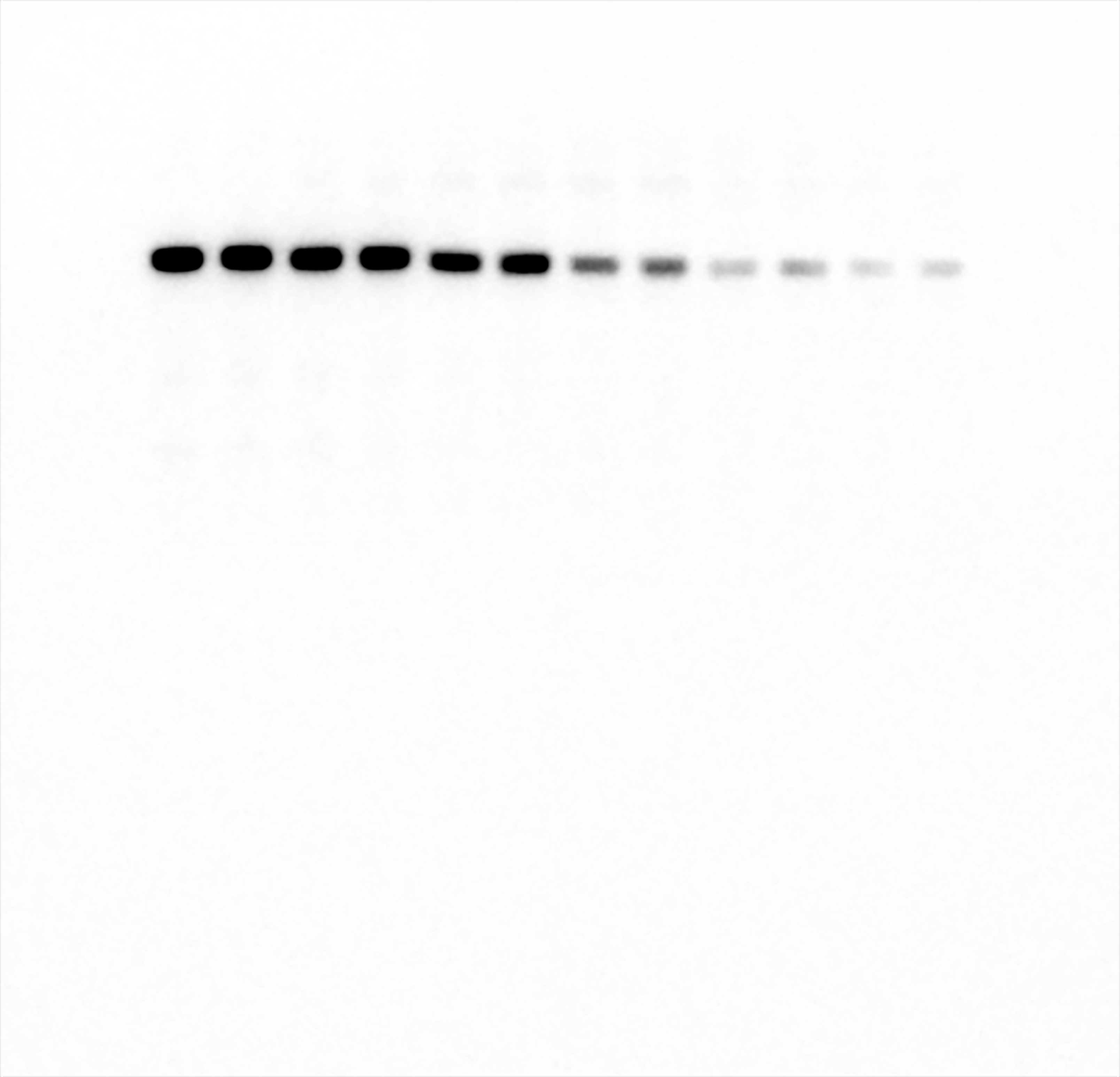

Supplement: Supplementary file 3 — Source data Fig. 1 [file 44321_2024_146_MOESM3_ESM.zip › Fig. 1/Fig. 1K/Fig. 1K-ptau396-concentration.tif]

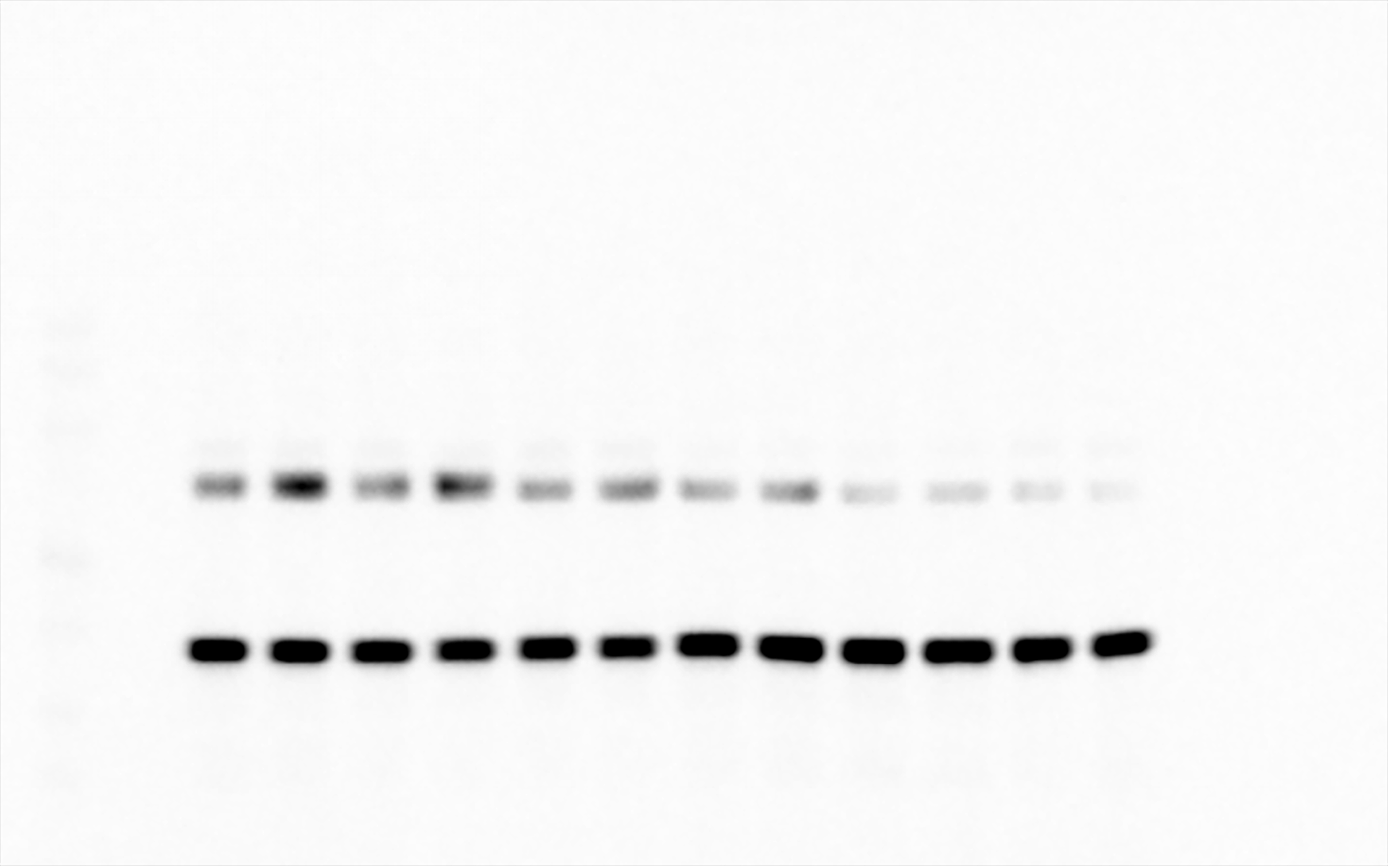

Supplement: Supplementary file 3 — Source data Fig. 1 [file 44321_2024_146_MOESM3_ESM.zip › Fig. 1/Fig. 1K/Fig. 1K-ptau231-GAPDH-concentration.tif]

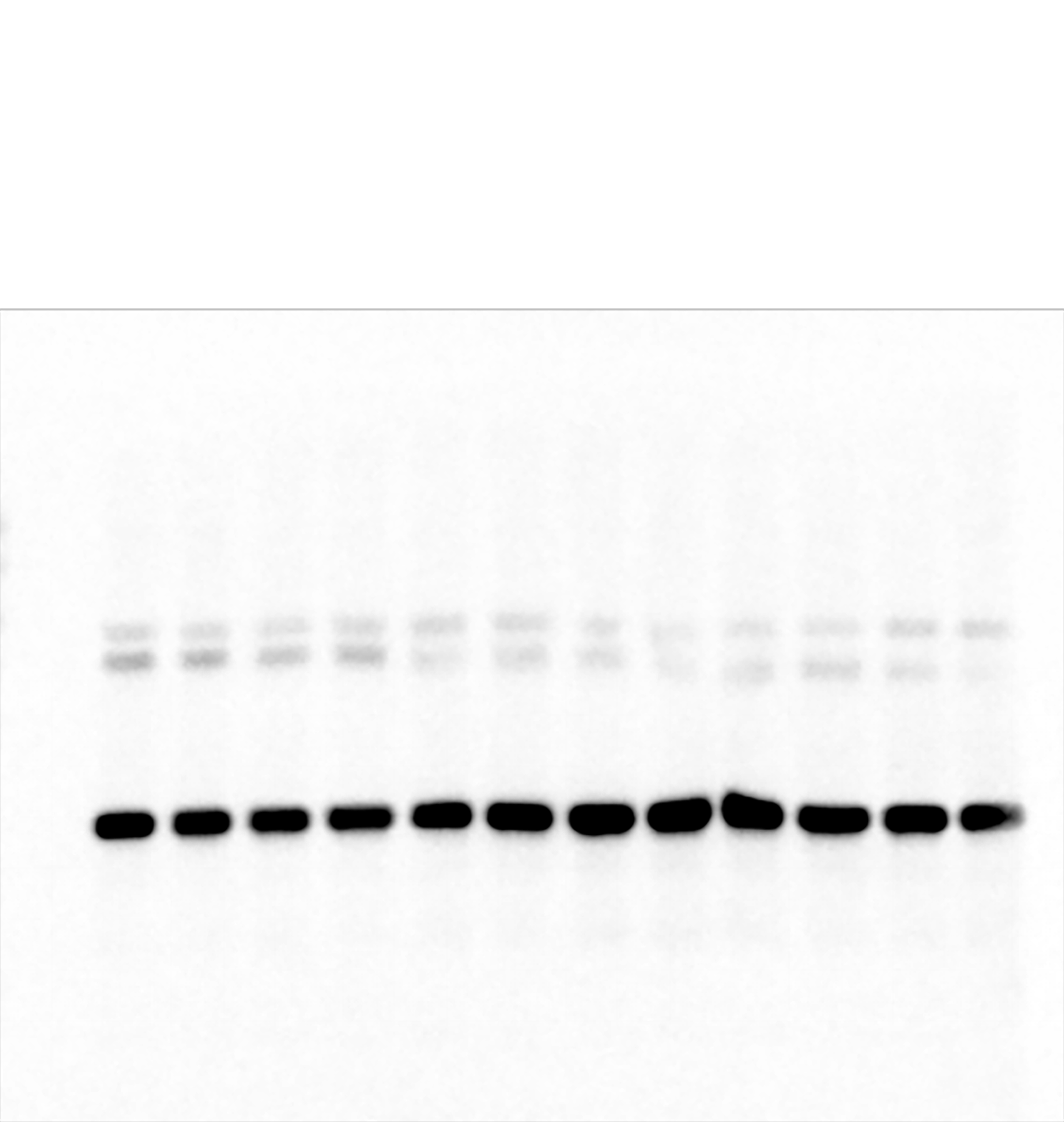

Supplement: Supplementary file 3 — Source data Fig. 1 [file 44321_2024_146_MOESM3_ESM.zip › Fig. 1/Fig. 1K/Fig. 1K-ptau181-GAPDH-concentration.tif]

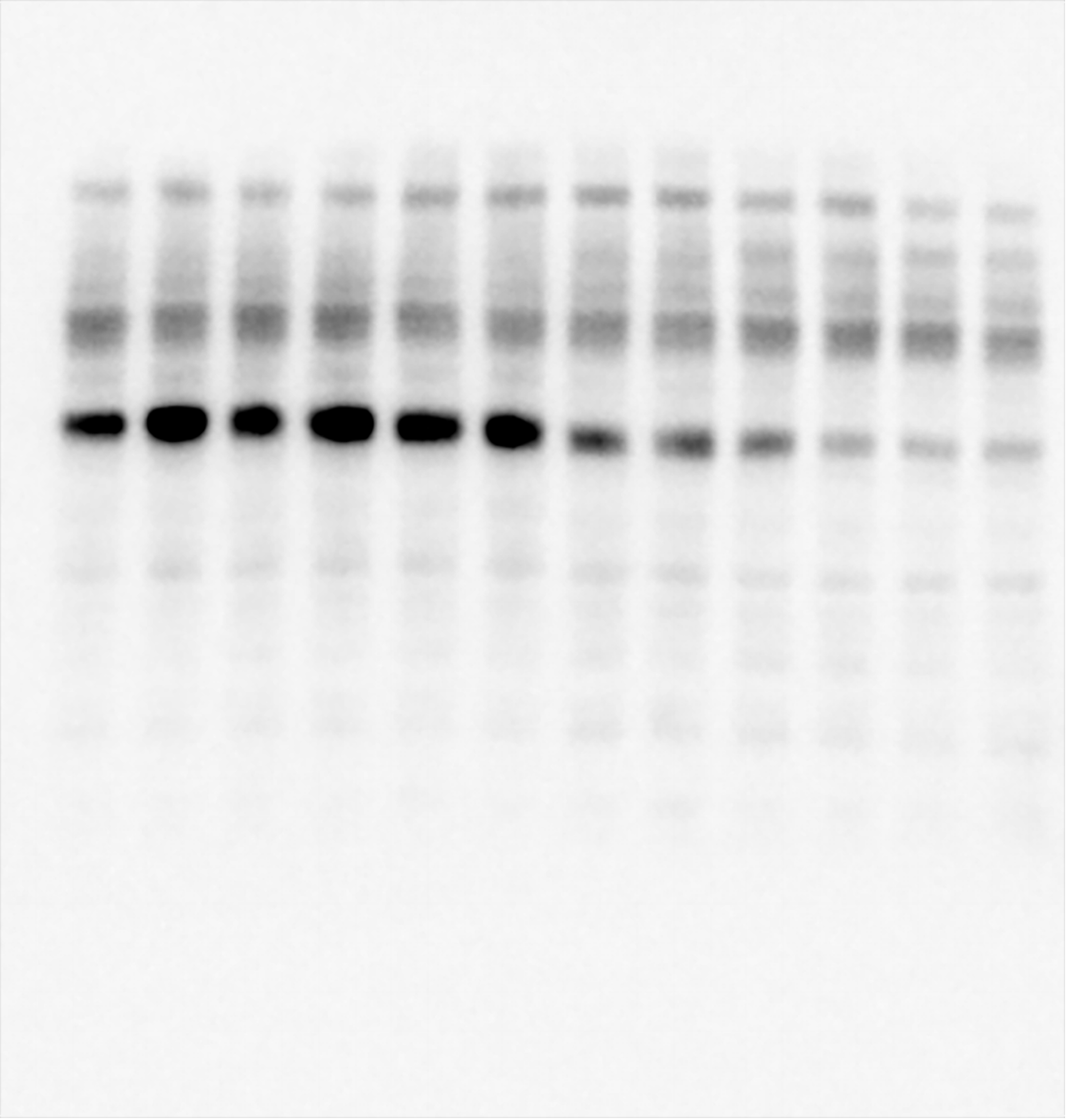

Supplement: Supplementary file 3 — Source data Fig. 1 [file 44321_2024_146_MOESM3_ESM.zip › Fig. 1/Fig. 1K/Fig. 1K-ptau202-205-concentration.tif]

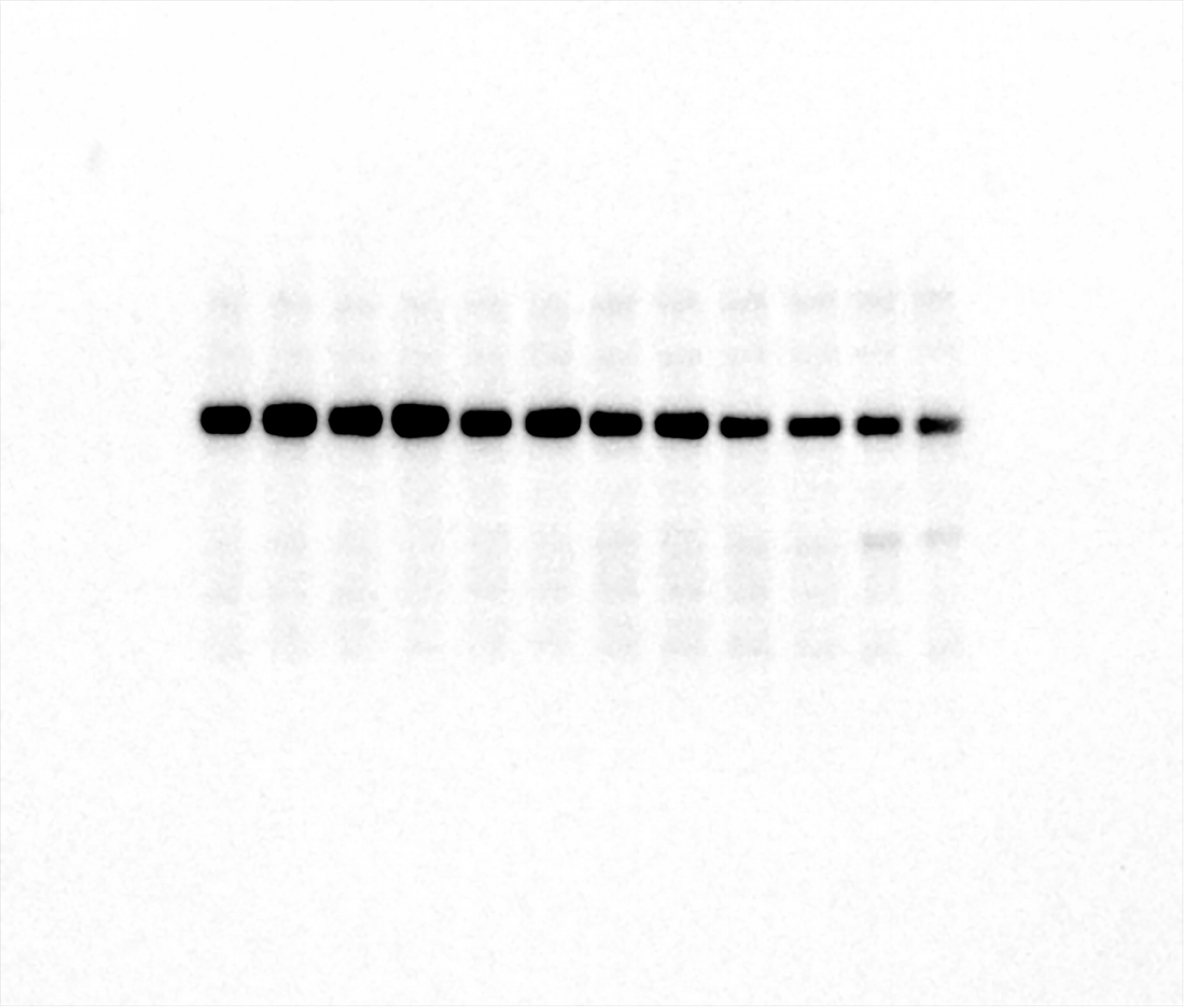

Supplement: Supplementary file 3 — Source data Fig. 1 [file 44321_2024_146_MOESM3_ESM.zip › Fig. 1/Fig. 1K/Fig. 1K-ptau231-concentration.tif]

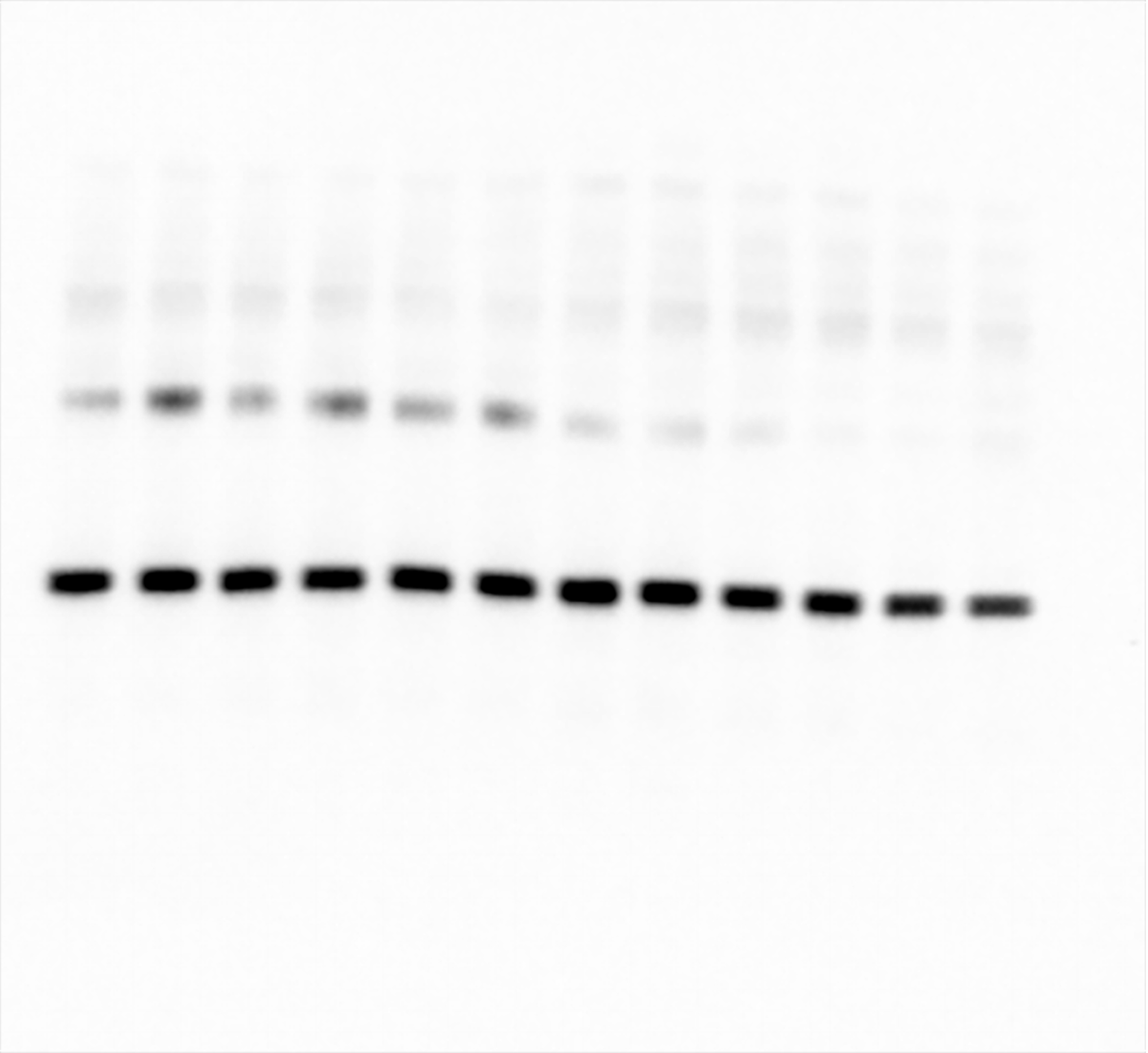

Supplement: Supplementary file 3 — Source data Fig. 1 [file 44321_2024_146_MOESM3_ESM.zip › Fig. 1/Fig. 1K/Fig. 1K-ptau202-205-GAPDH-concentration.tif]

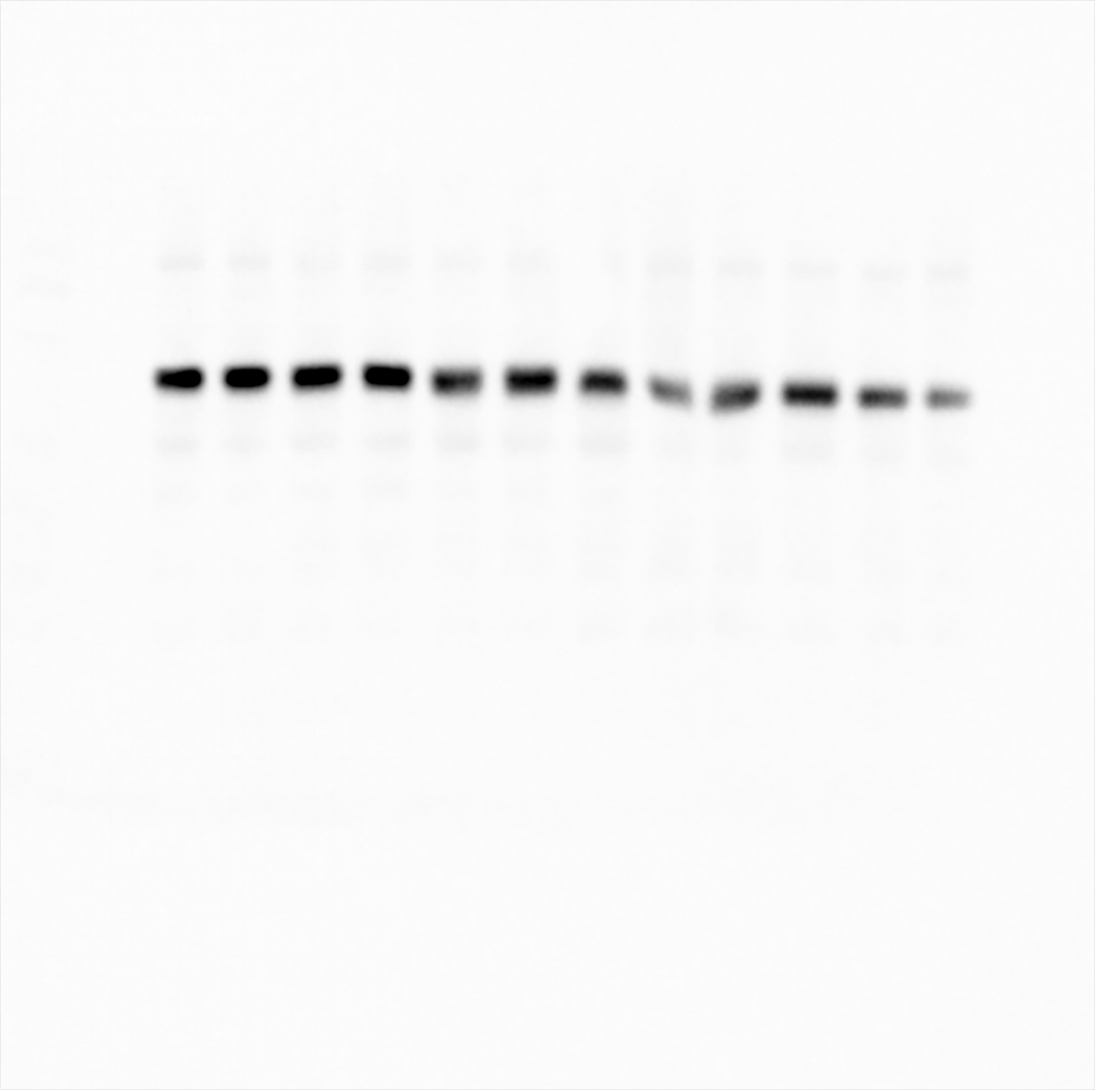

Supplement: Supplementary file 3 — Source data Fig. 1 [file 44321_2024_146_MOESM3_ESM.zip › Fig. 1/Fig. 1K/Fig. 1K-ptau181-concentration.tif]

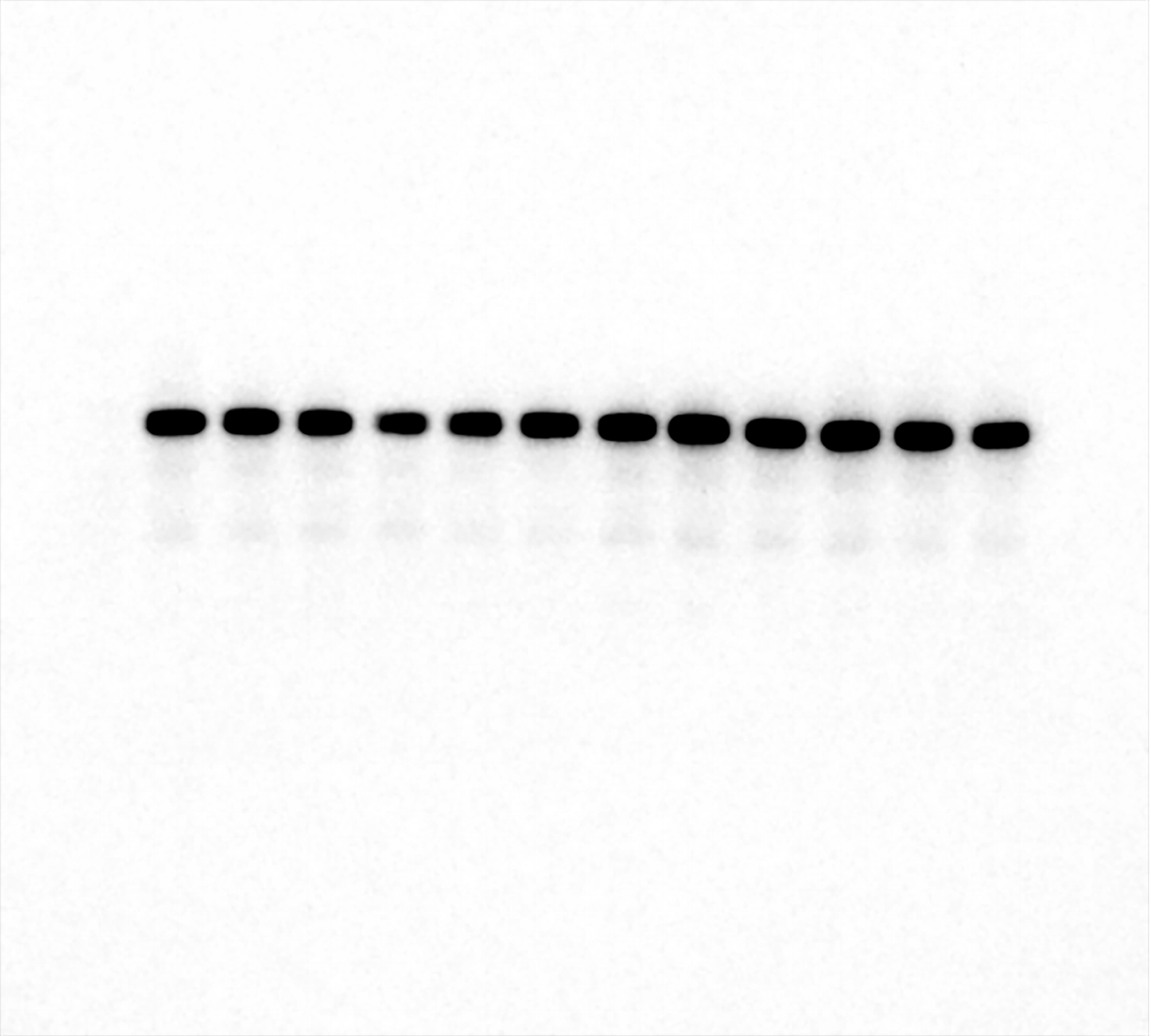

Supplement: Supplementary file 3 — Source data Fig. 1 [file 44321_2024_146_MOESM3_ESM.zip › Fig. 1/Fig. 1K/Fig. 1K-ptau396-GAPDH-concentration.tif]

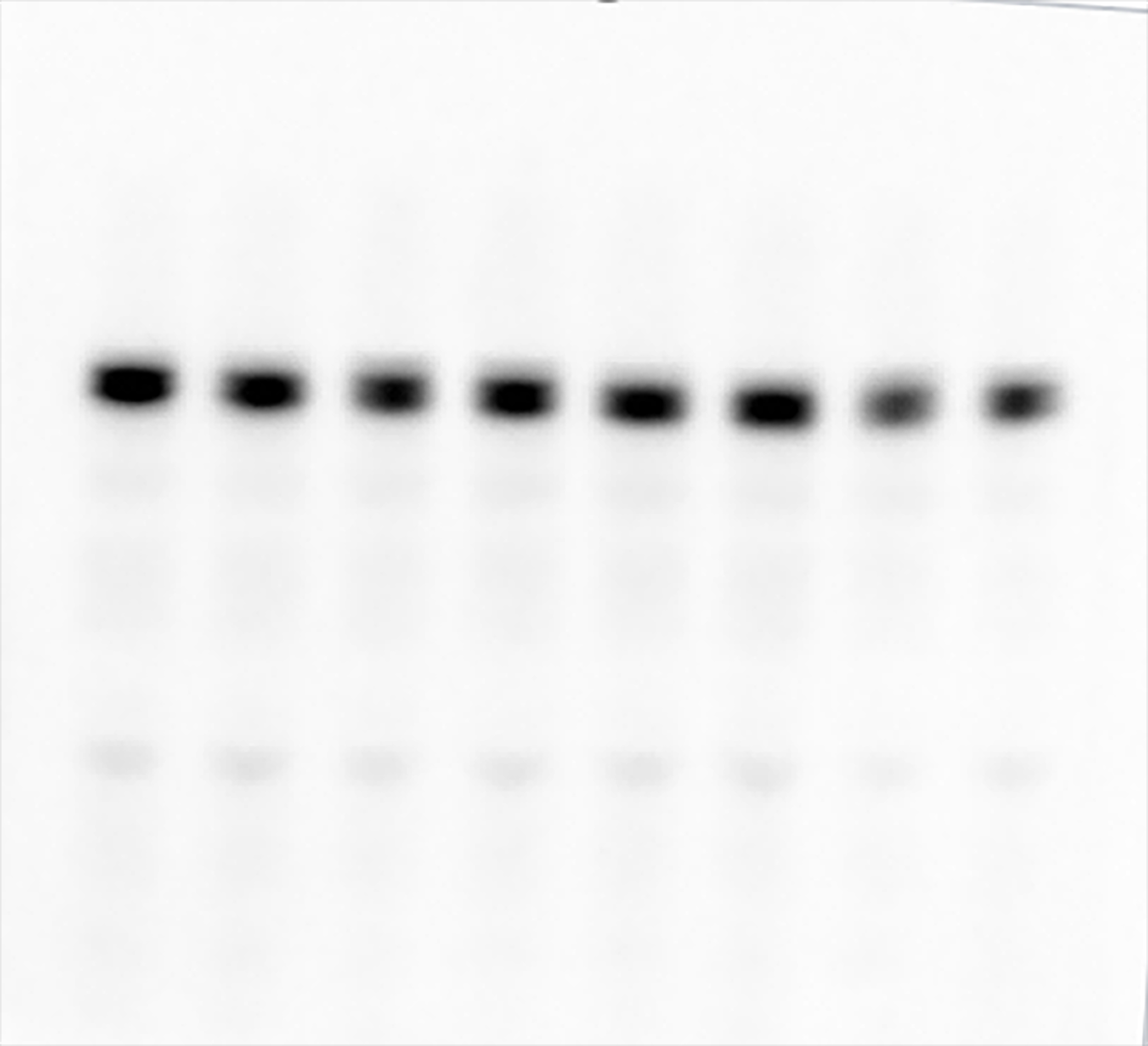

Supplement: Supplementary file 4 — Source data Fig. 2 [file 44321_2024_146_MOESM4_ESM.zip › Fig. 2/Fig. 2F/Fig. 2F-USP11-solution.tif]

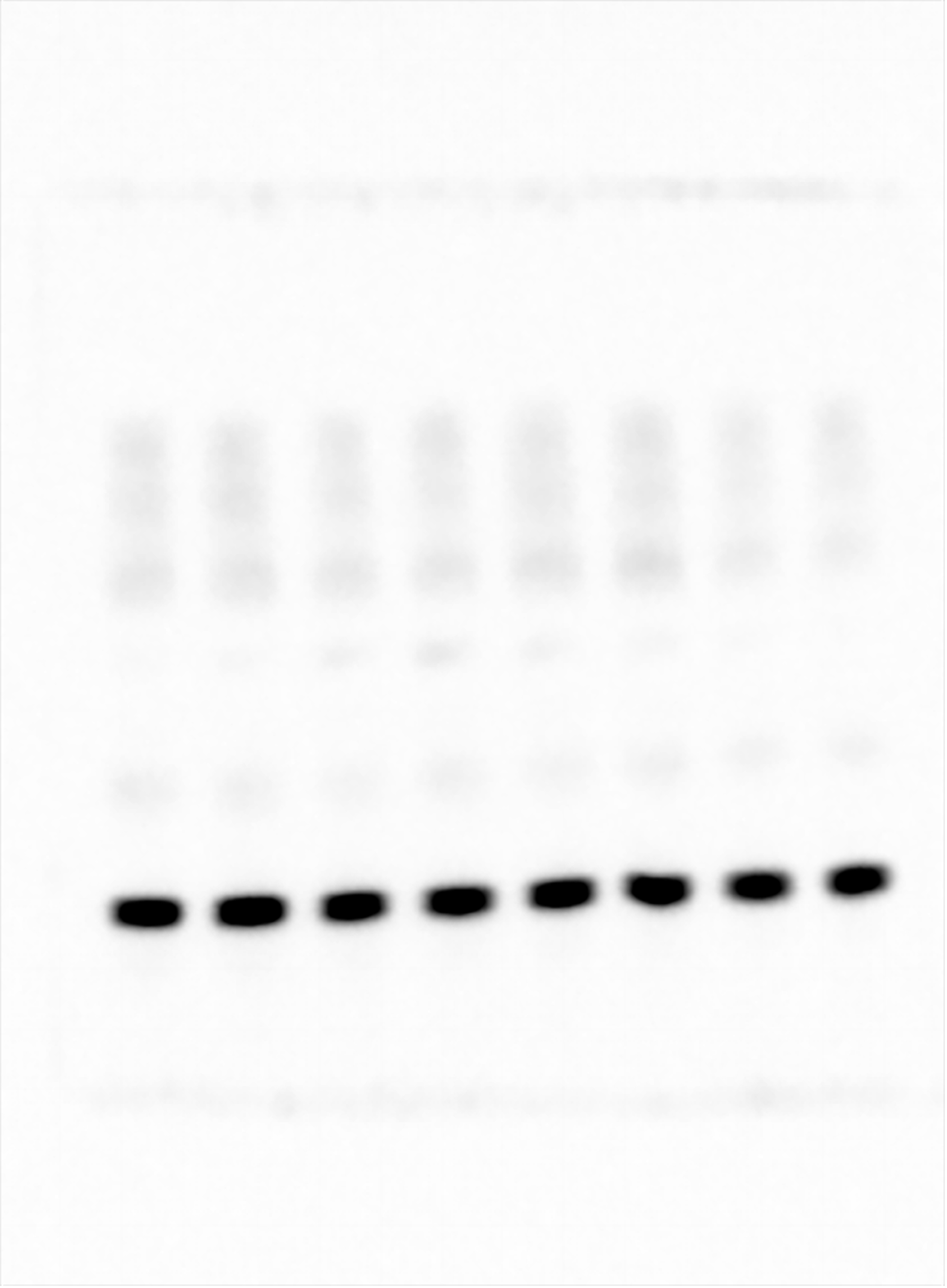

Supplement: Supplementary file 4 — Source data Fig. 2 [file 44321_2024_146_MOESM4_ESM.zip › Fig. 2/Fig. 2F/Fig. 2F-GAPDH-solution.tif]

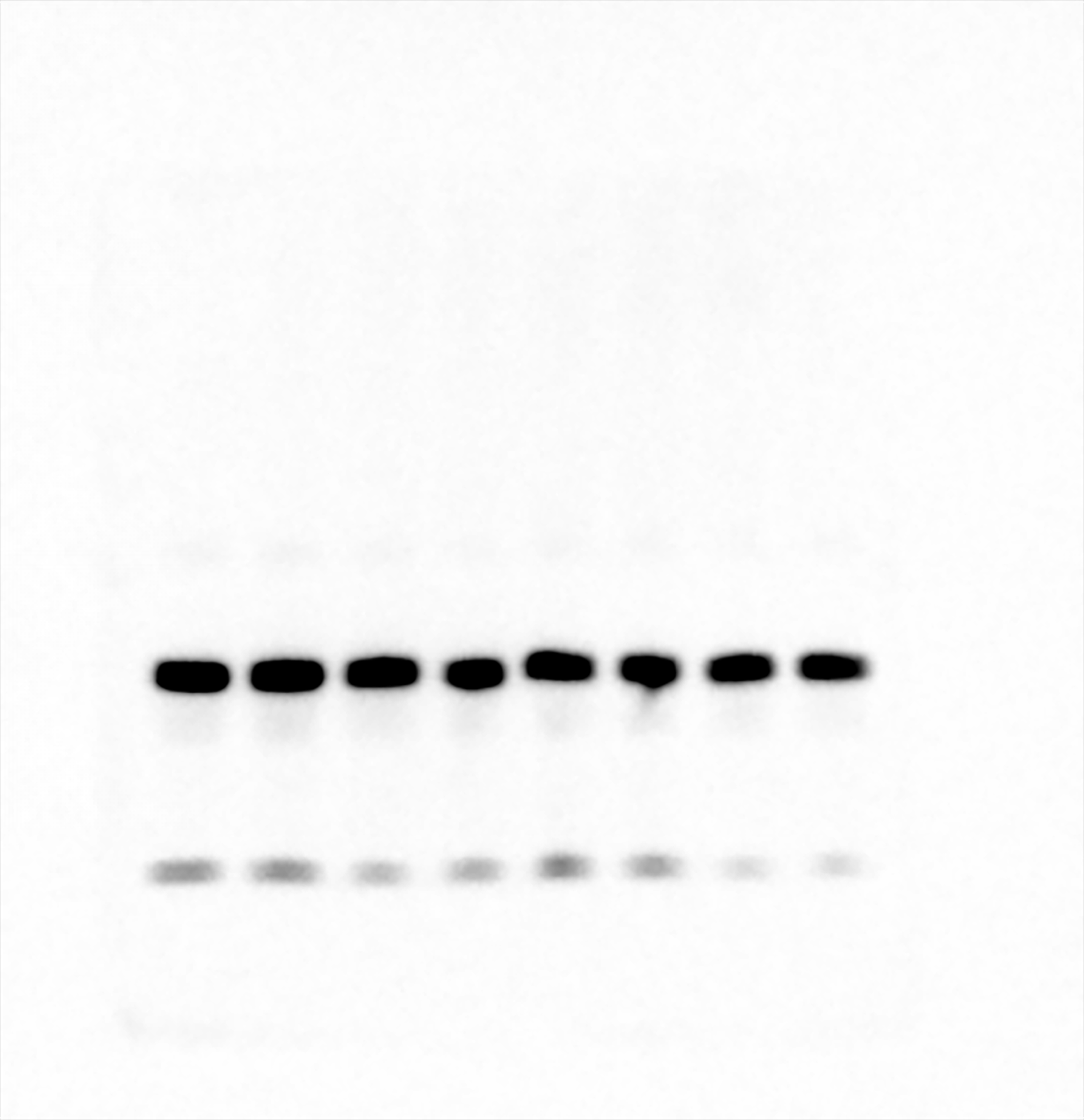

Supplement: Supplementary file 4 — Source data Fig. 2 [file 44321_2024_146_MOESM4_ESM.zip › Fig. 2/Fig. 2F/Fig. 2F-GAPDH-Insolution.tif]

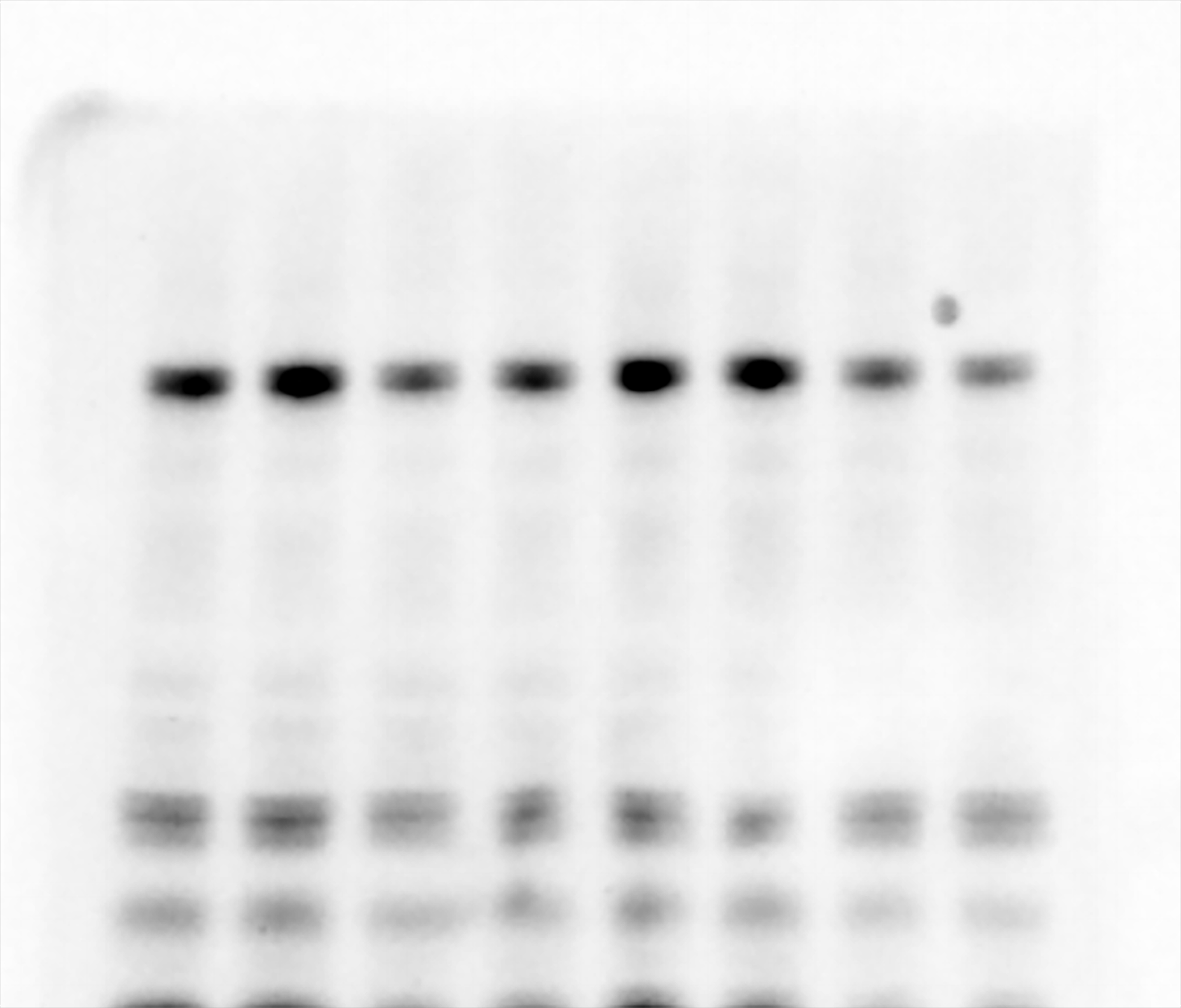

Supplement: Supplementary file 4 — Source data Fig. 2 [file 44321_2024_146_MOESM4_ESM.zip › Fig. 2/Fig. 2F/Fig. 2F-USP11-Insolution.tif]

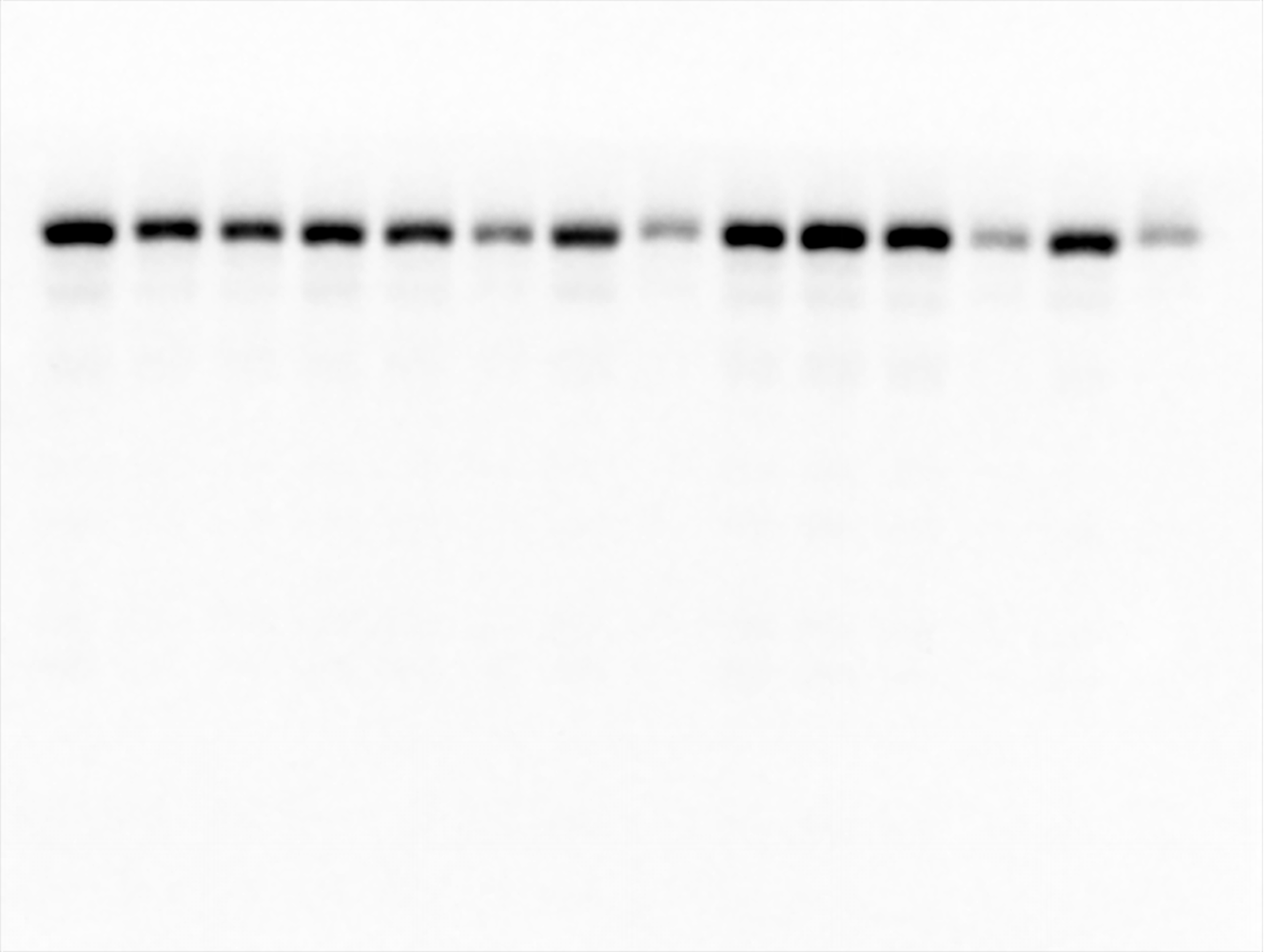

Supplement: Supplementary file 4 — Source data Fig. 2 [file 44321_2024_146_MOESM4_ESM.zip › Fig. 2/Fig. 2A/Fig. 2A-USP11.tif]

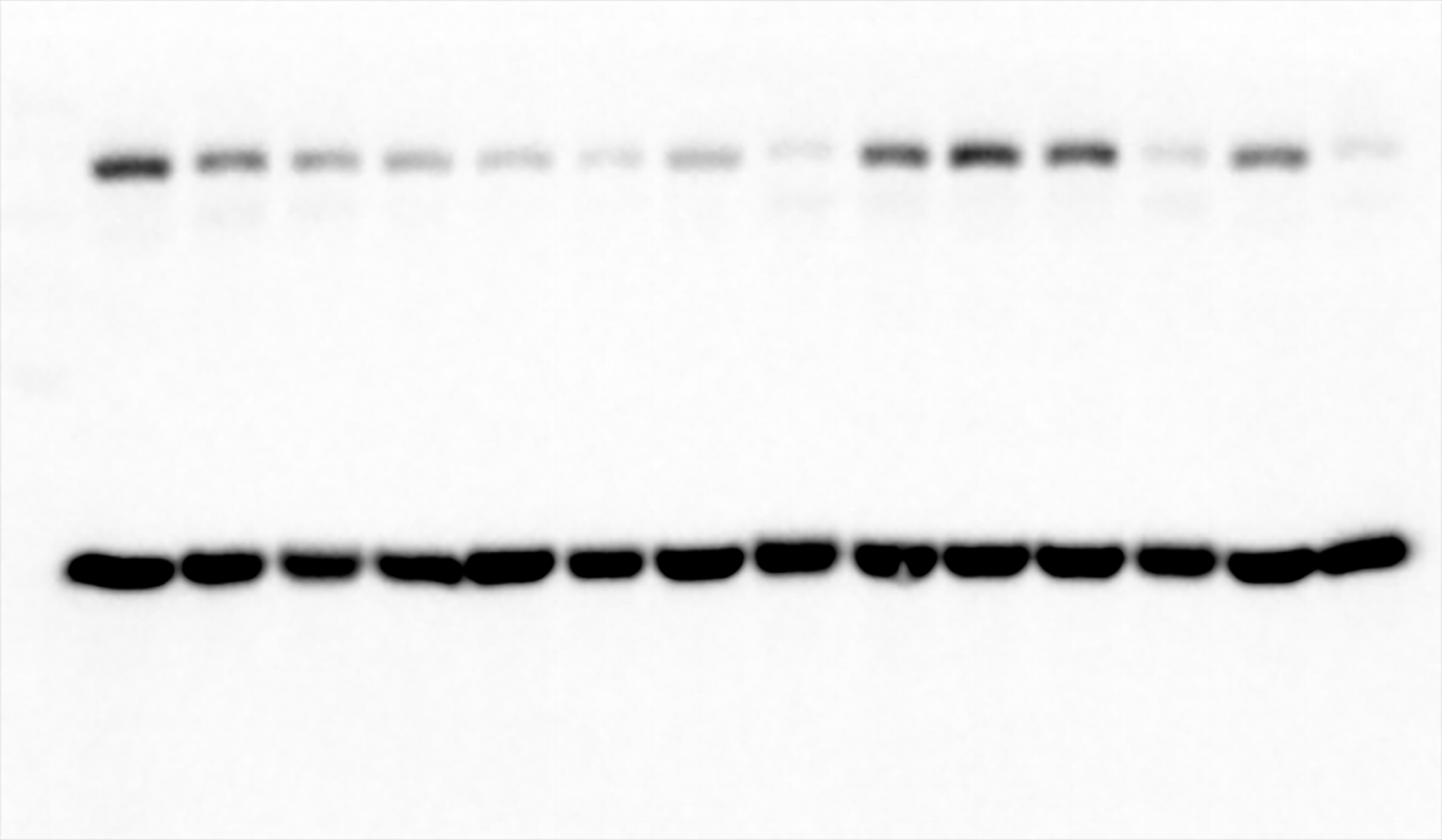

Supplement: Supplementary file 4 — Source data Fig. 2 [file 44321_2024_146_MOESM4_ESM.zip › Fig. 2/Fig. 2A/Fig. 2A-GAPDH.tif]

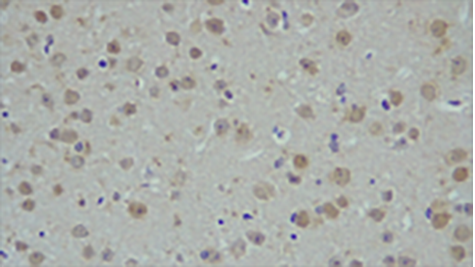

Supplement: Supplementary file 4 — Source data Fig. 2 [file 44321_2024_146_MOESM4_ESM.zip › Fig. 2/Fig. 2G/Fig. 2G-3xTg-mice-ddH2O-cortex.tif]

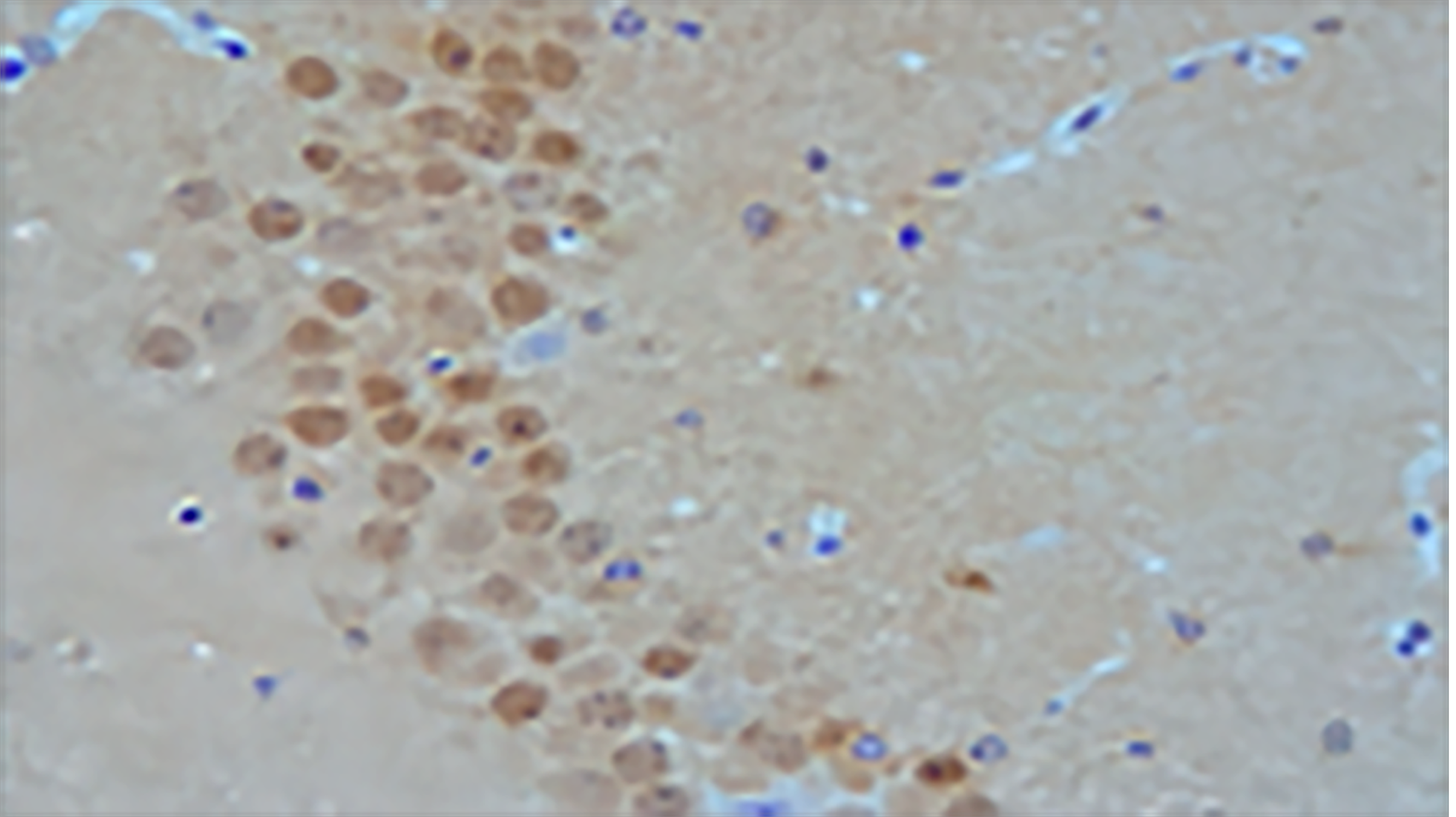

Supplement: Supplementary file 4 — Source data Fig. 2 [file 44321_2024_146_MOESM4_ESM.zip › Fig. 2/Fig. 2G/Fig. 2G-3xTg-mice-ddH2O-CA3.tif]

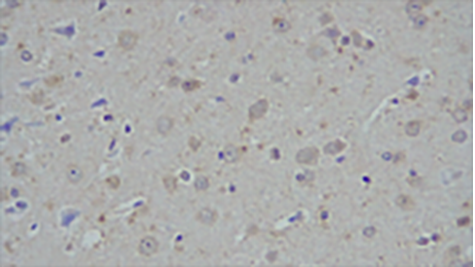

Supplement: Supplementary file 4 — Source data Fig. 2 [file 44321_2024_146_MOESM4_ESM.zip › Fig. 2/Fig. 2G/Fig. 2G-3xTg-mice-IsoLiPro-cortex.tif]

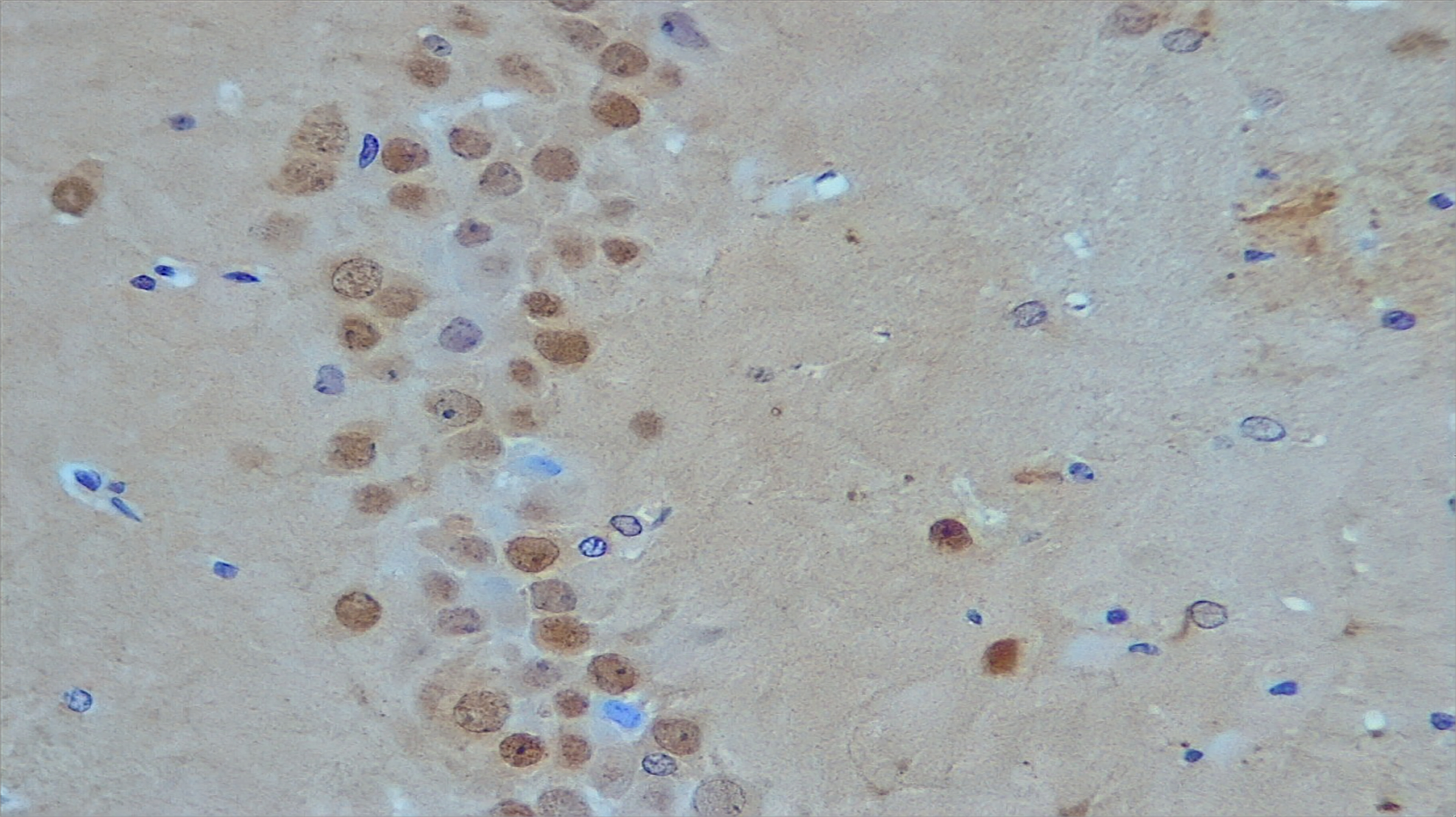

Supplement: Supplementary file 4 — Source data Fig. 2 [file 44321_2024_146_MOESM4_ESM.zip › Fig. 2/Fig. 2G/Fig. 2G-3xTg-mice-IsoLiPro-CA3.tif]

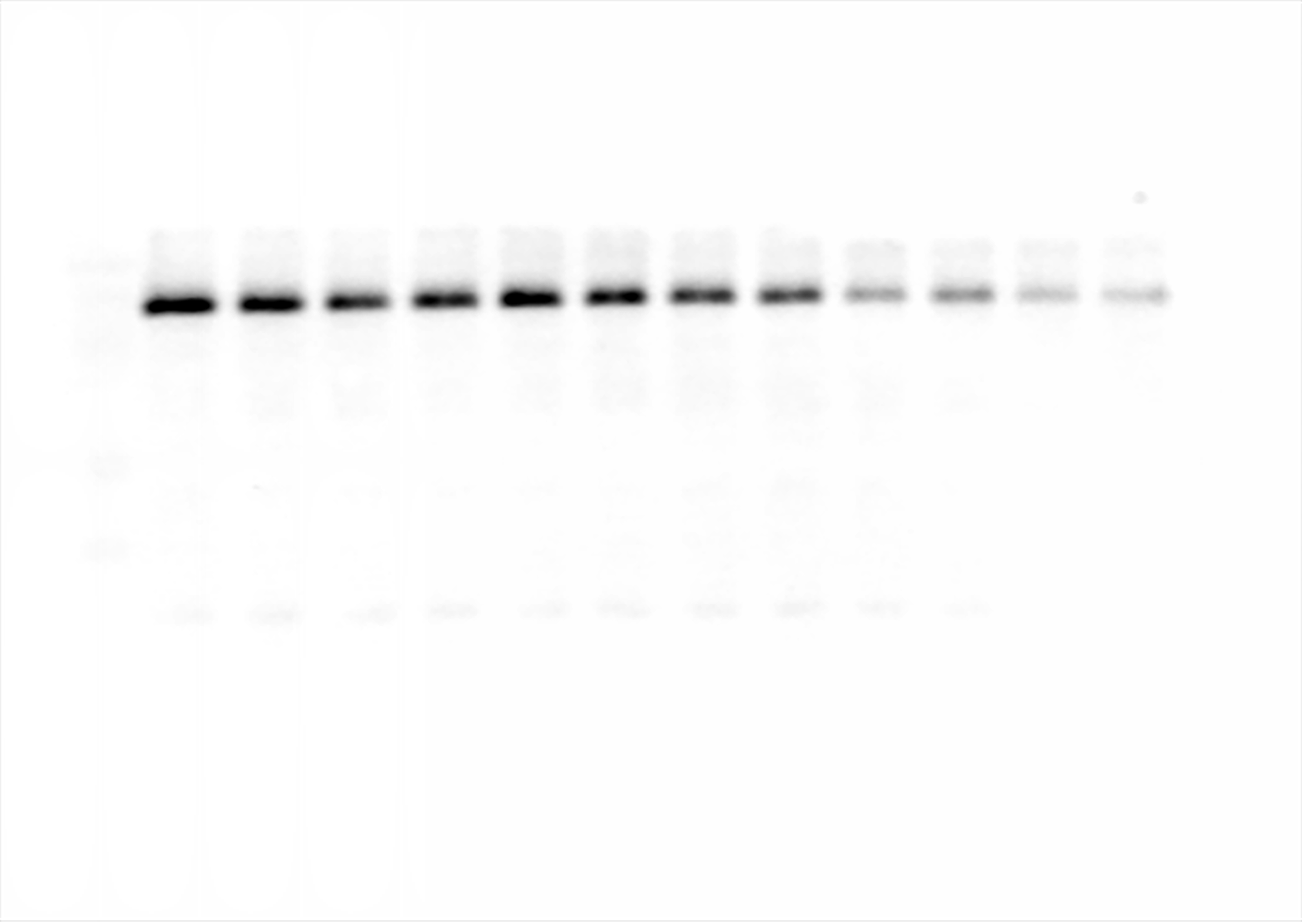

Supplement: Supplementary file 4 — Source data Fig. 2 [file 44321_2024_146_MOESM4_ESM.zip › Fig. 2/Fig. 2B/Fig. 2B-USP11-concentration.tif]

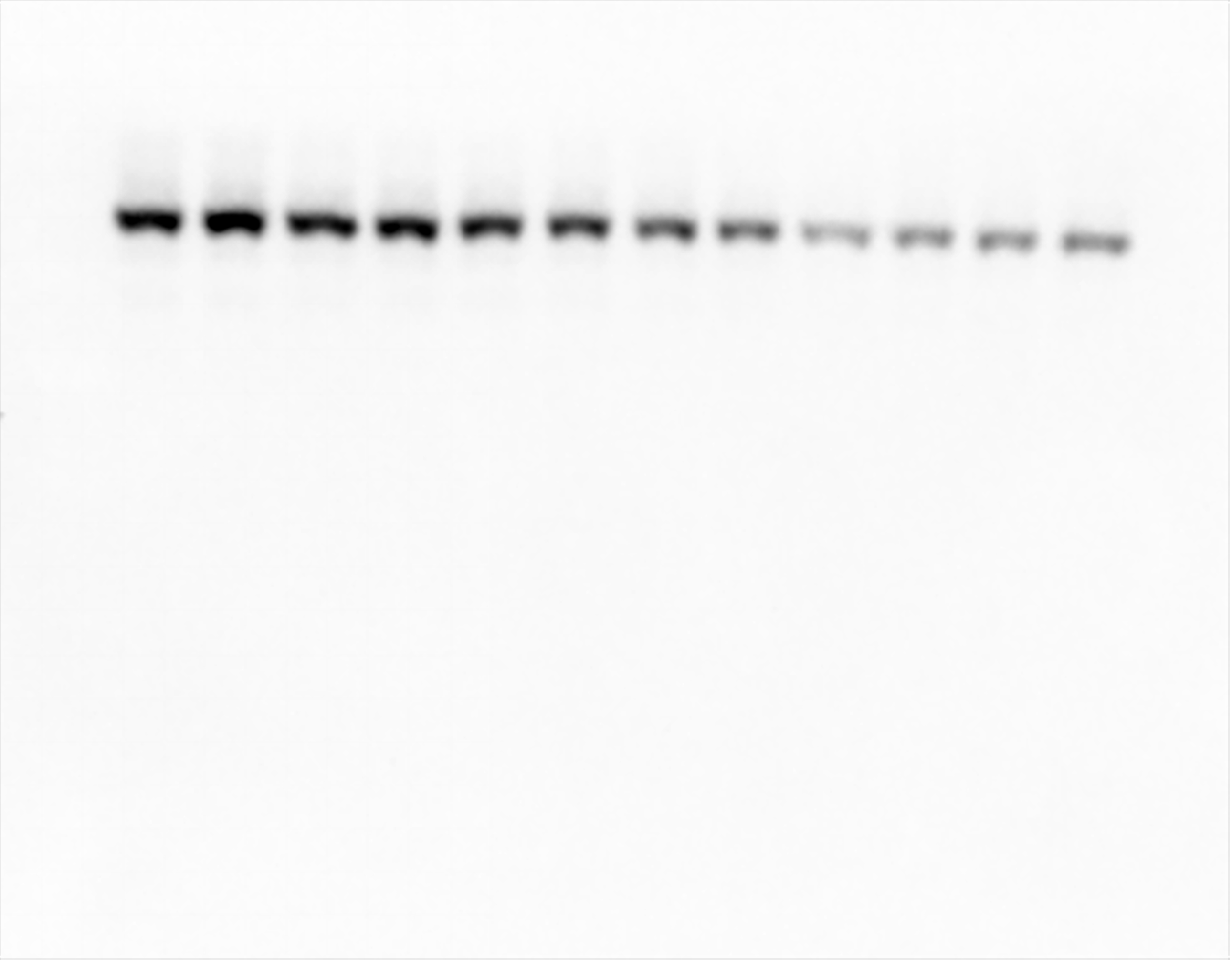

Supplement: Supplementary file 4 — Source data Fig. 2 [file 44321_2024_146_MOESM4_ESM.zip › Fig. 2/Fig. 2B/Fig. 2B-USP11-Time.tif]

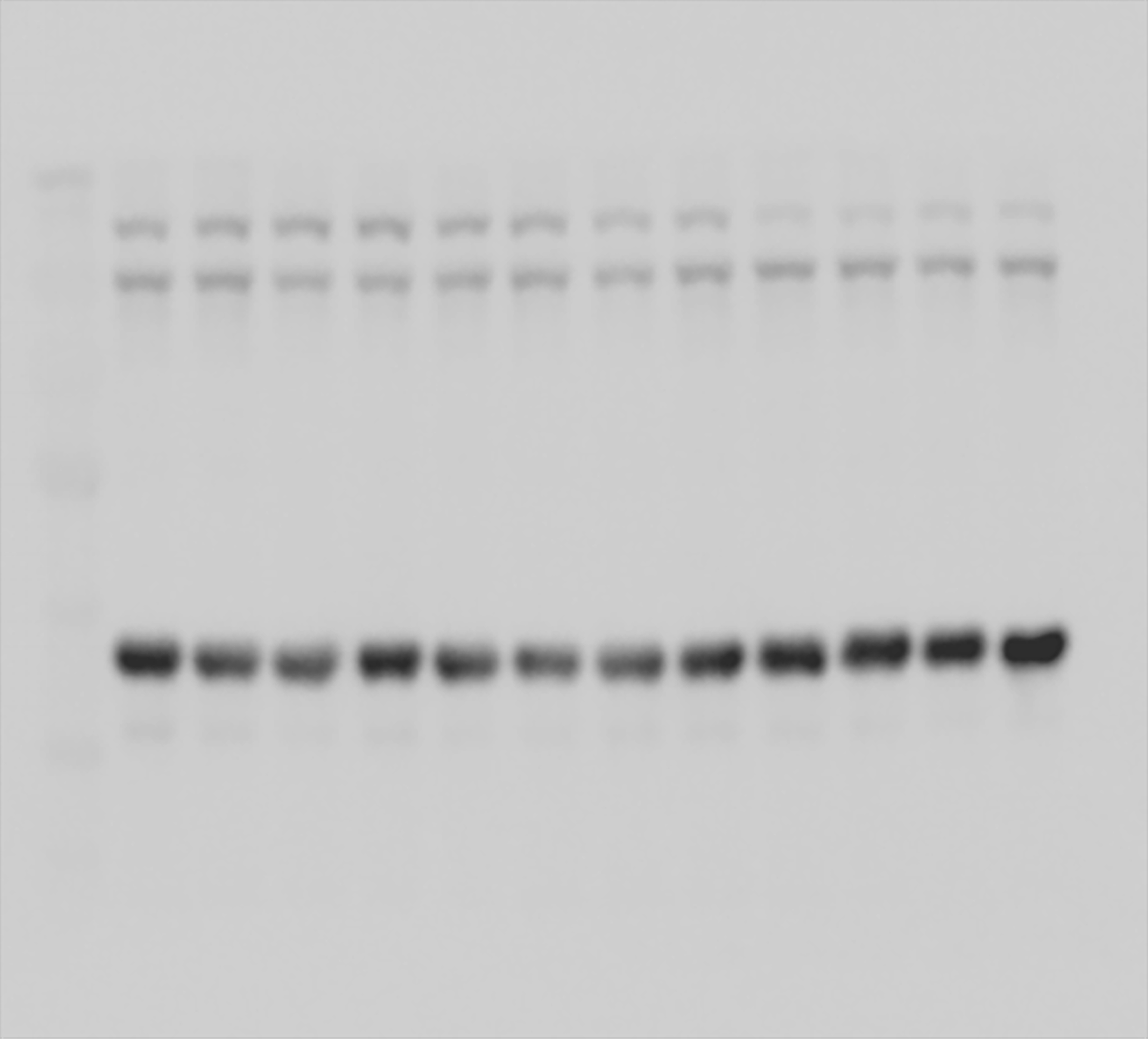

Supplement: Supplementary file 4 — Source data Fig. 2 [file 44321_2024_146_MOESM4_ESM.zip › Fig. 2/Fig. 2B/Fig. 2B-GAPDH-Time.tif]

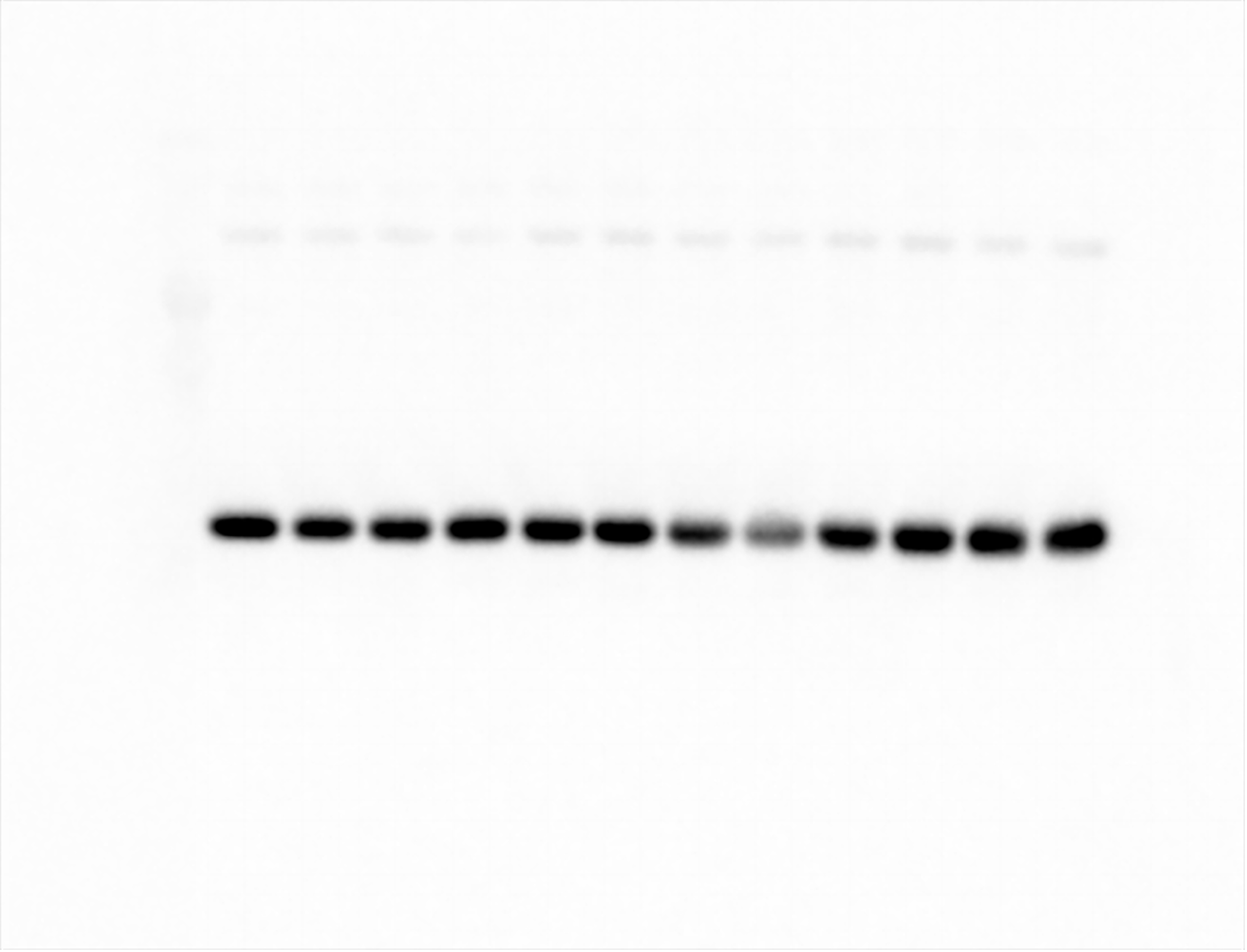

Supplement: Supplementary file 4 — Source data Fig. 2 [file 44321_2024_146_MOESM4_ESM.zip › Fig. 2/Fig. 2B/Fig. 2B-GAPDH-concentration.tif]

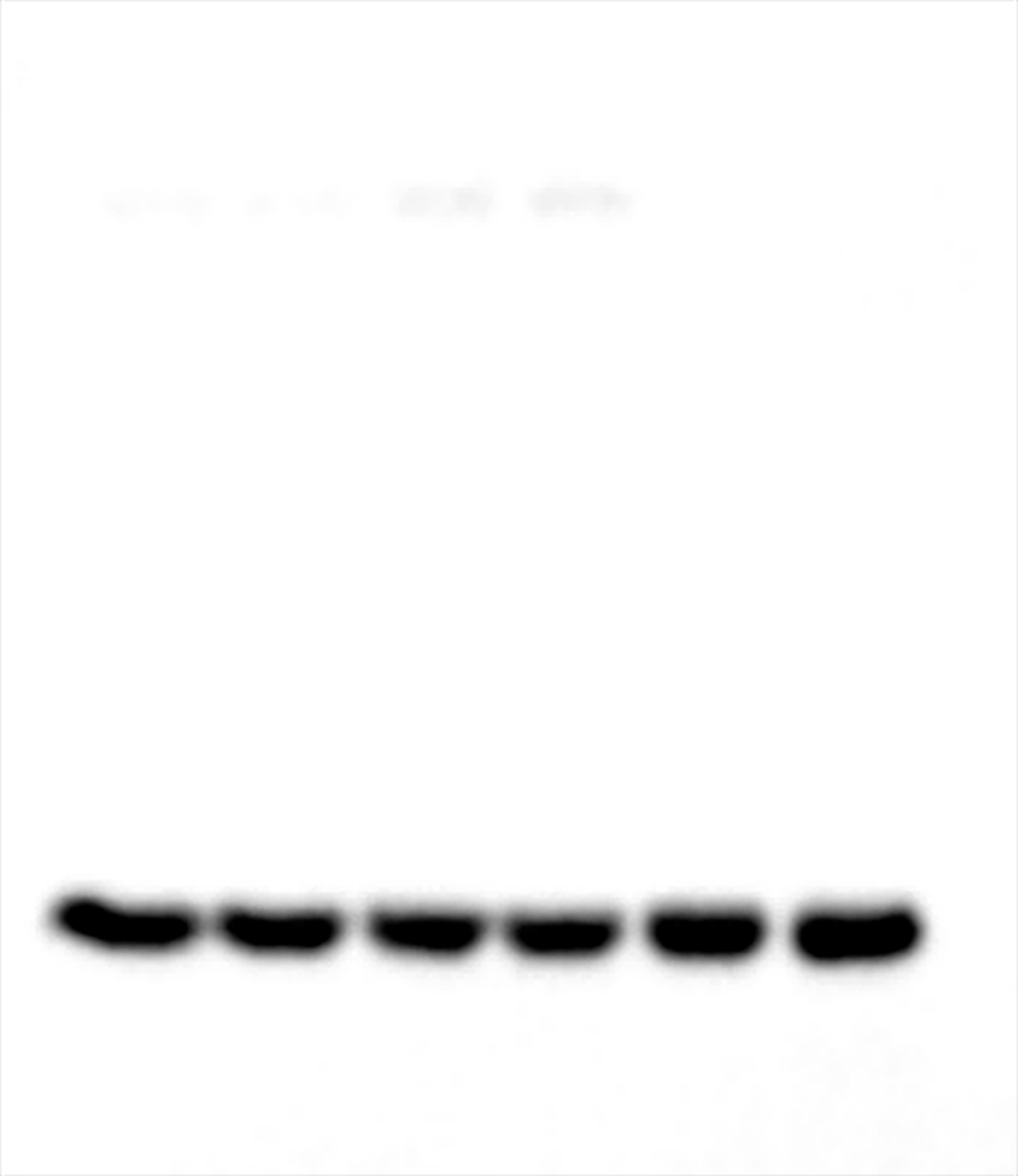

Supplement: Supplementary file 4 — Source data Fig. 2 [file 44321_2024_146_MOESM4_ESM.zip › Fig. 2/Fig. 2D/Fig. 2D-GAPDH.tif]

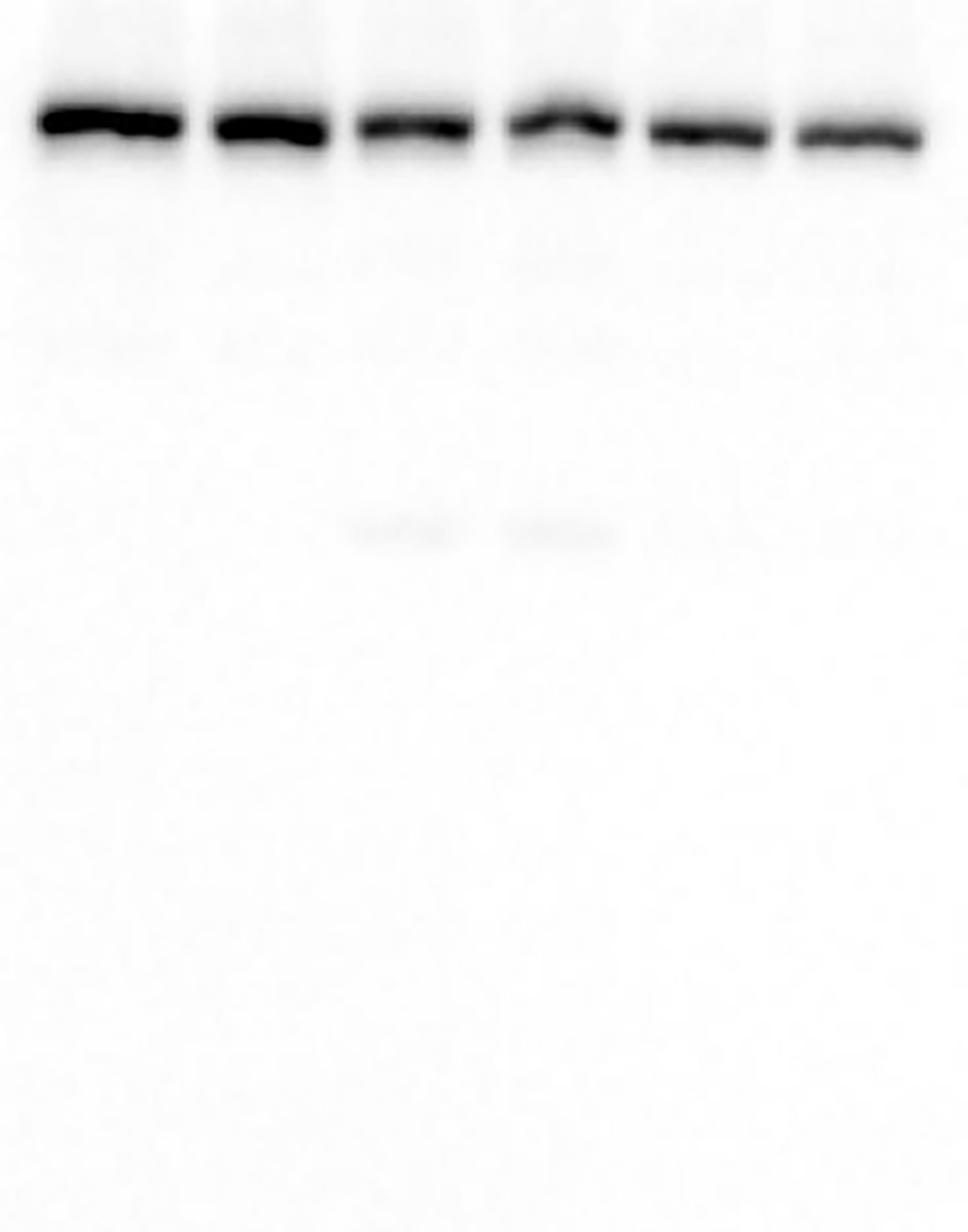

Supplement: Supplementary file 4 — Source data Fig. 2 [file 44321_2024_146_MOESM4_ESM.zip › Fig. 2/Fig. 2D/Fig. 2D-USP25.tif]

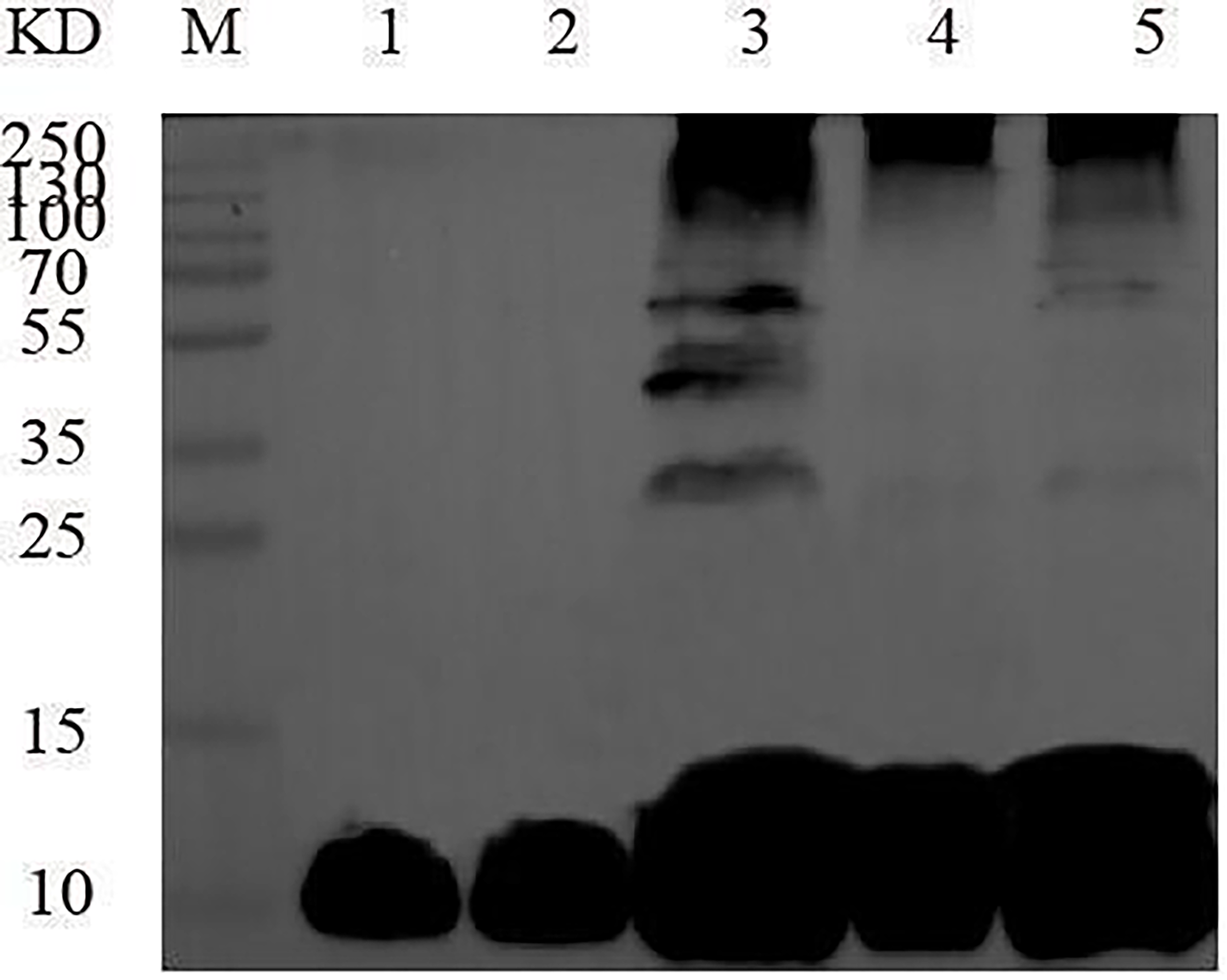

Supplement: Supplementary file 5 — Source data Fig. 3 [file 44321_2024_146_MOESM5_ESM.zip › Fig. 3/Fig. 3D/Fig. 3D.tif]

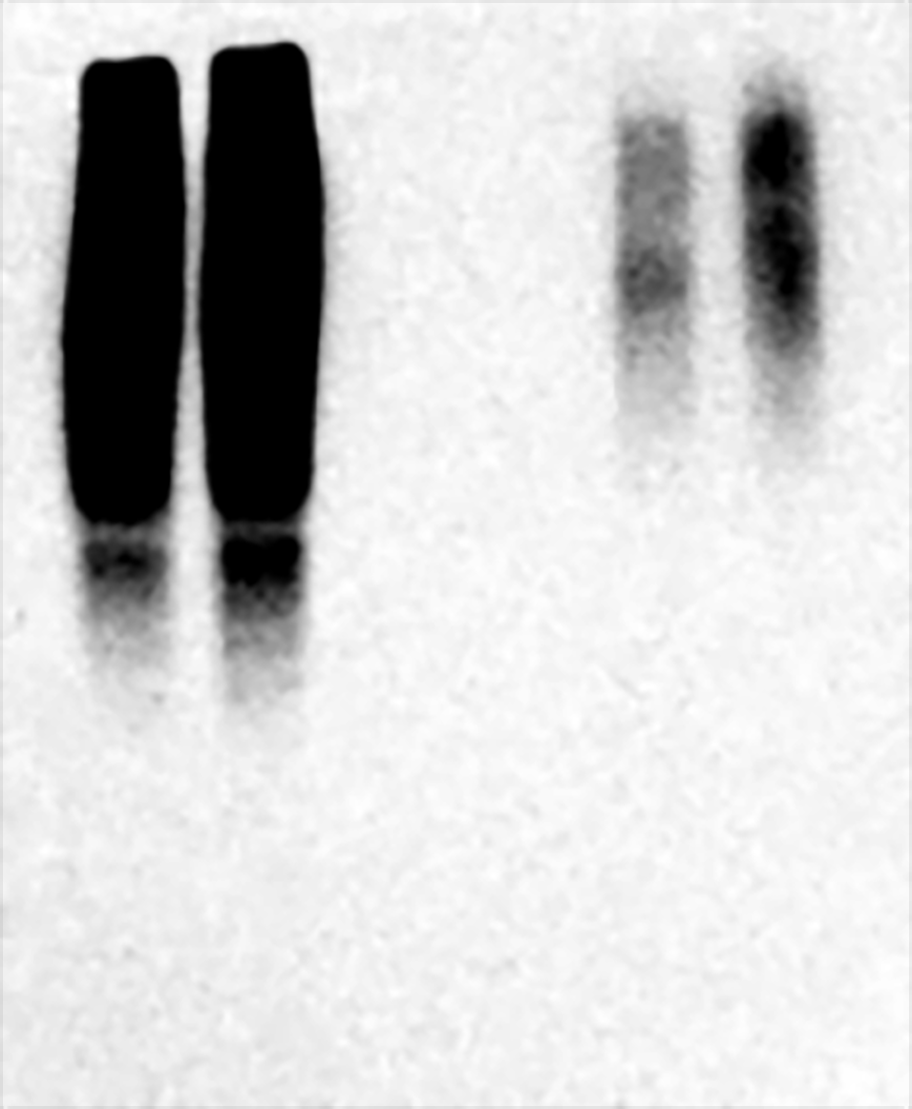

Supplement: Supplementary file 5 — Source data Fig. 3 [file 44321_2024_146_MOESM5_ESM.zip › Fig. 3/Fig. 3A/Fig. 3A-ub-K48-right.tif]

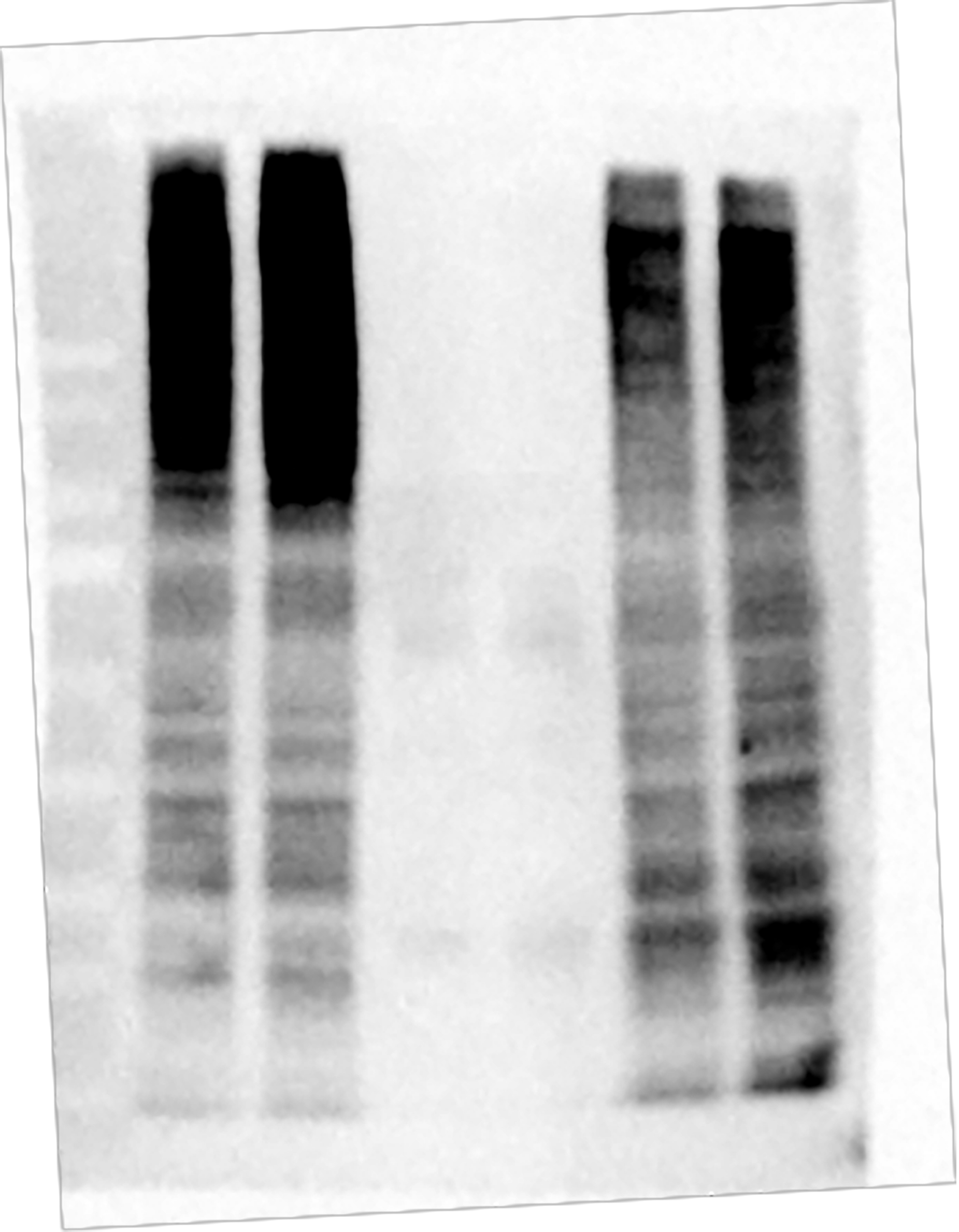

Supplement: Supplementary file 5 — Source data Fig. 3 [file 44321_2024_146_MOESM5_ESM.zip › Fig. 3/Fig. 3A/Fig. 3A-ub-K48.tif]

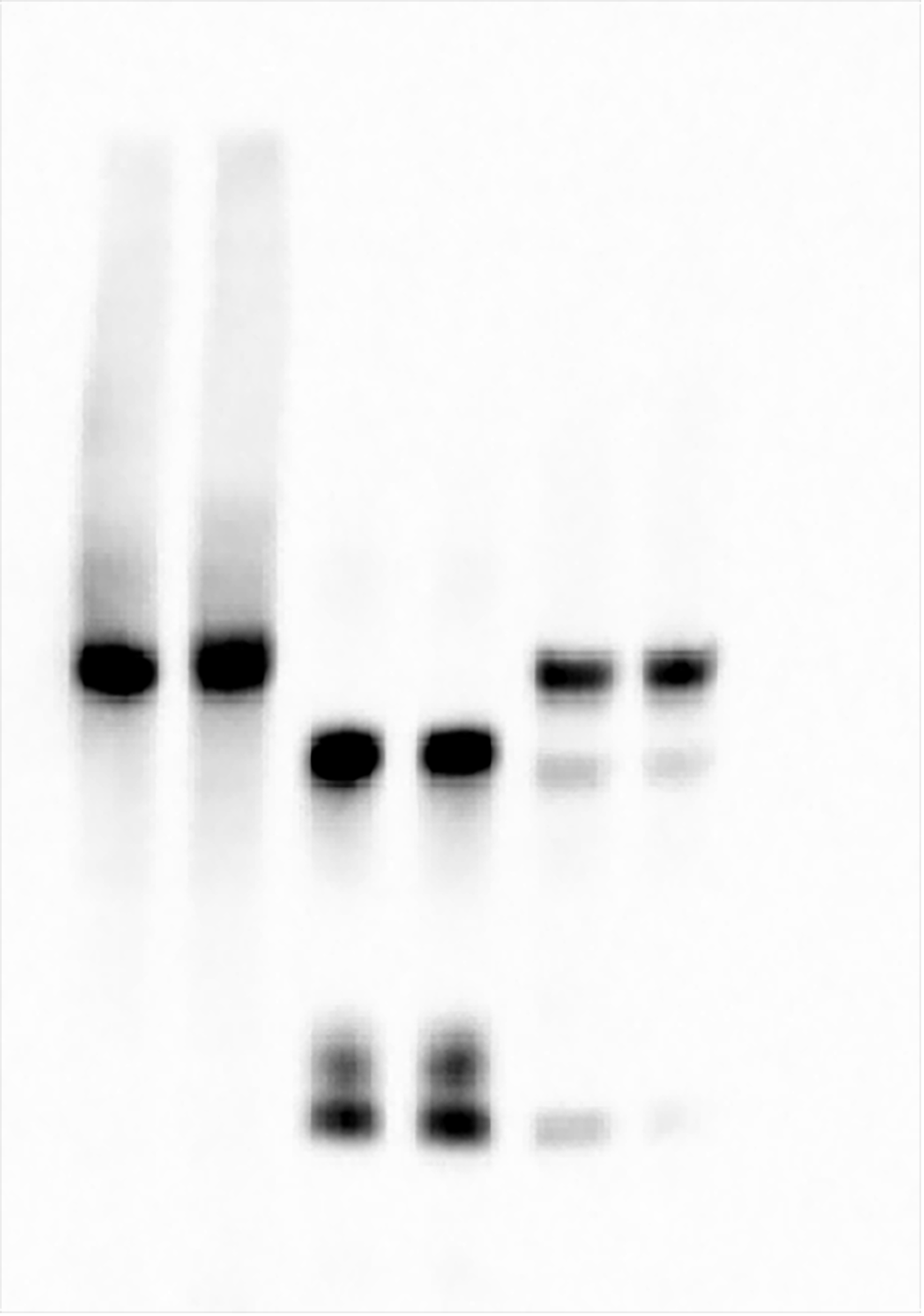

Supplement: Supplementary file 5 — Source data Fig. 3 [file 44321_2024_146_MOESM5_ESM.zip › Fig. 3/Fig. 3A/Fig. 3A-ub-K48-tau-right.tif]

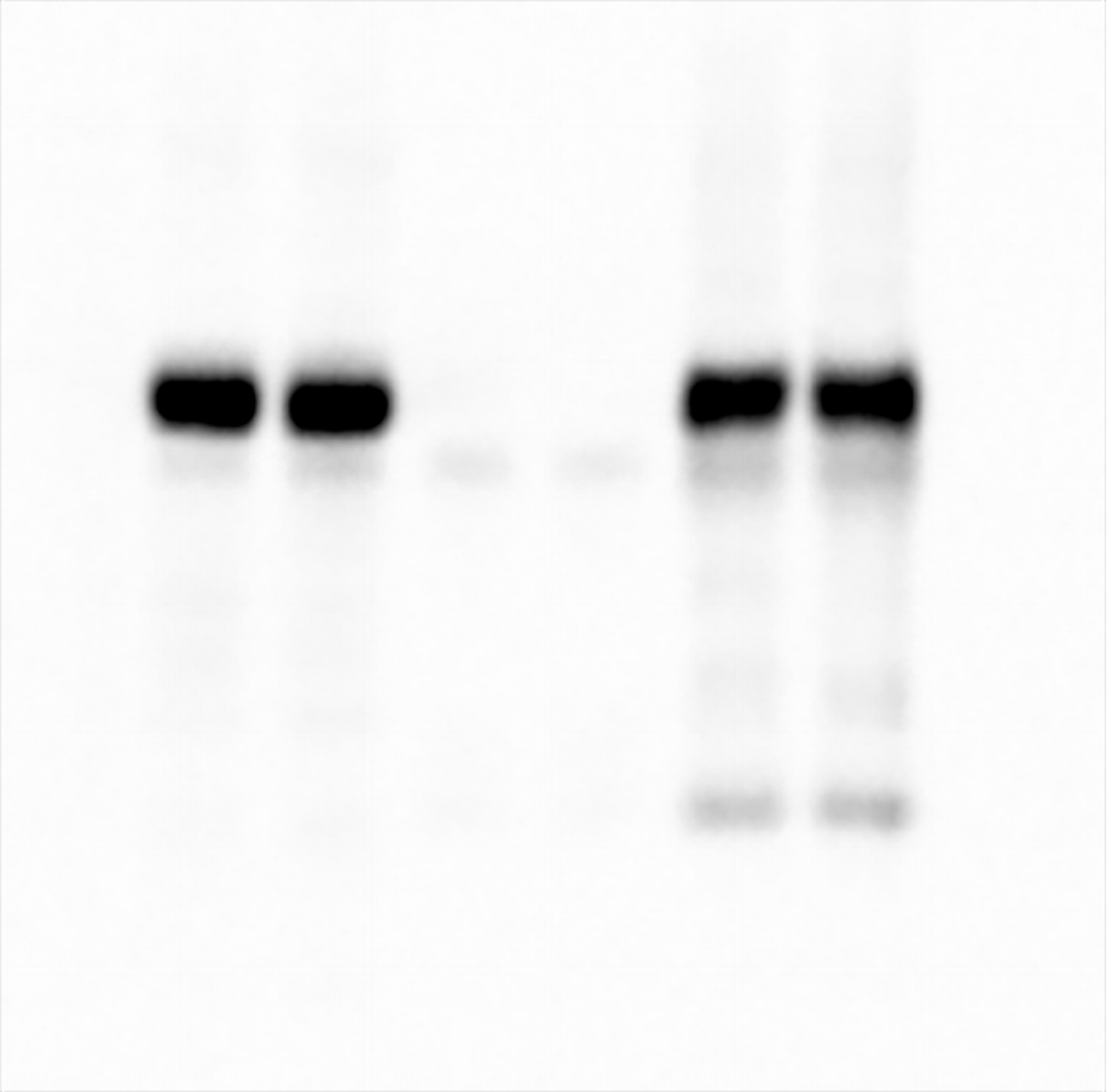

Supplement: Supplementary file 5 — Source data Fig. 3 [file 44321_2024_146_MOESM5_ESM.zip › Fig. 3/Fig. 3A/Fig. 3A-ub-tau.tif]

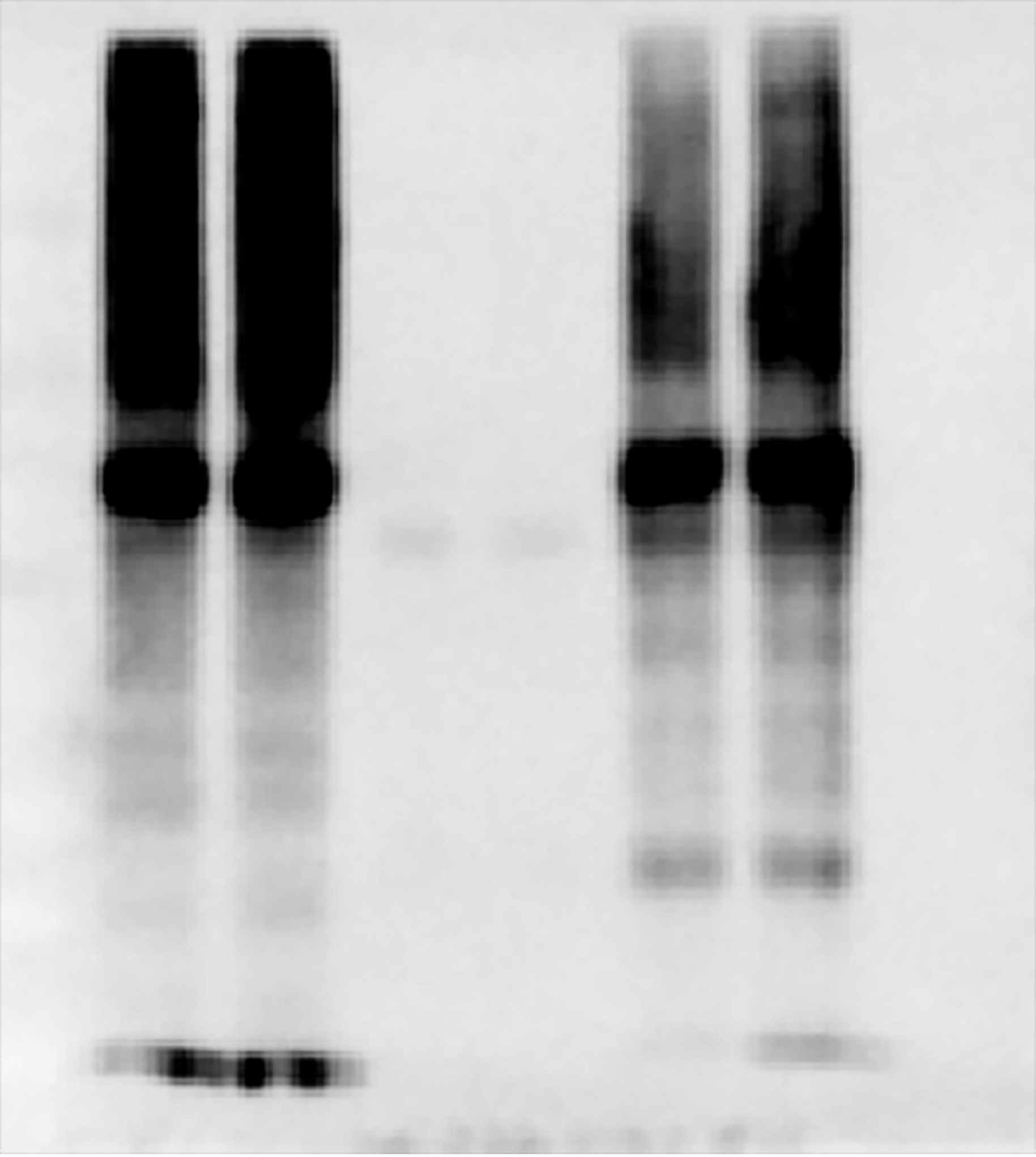

Supplement: Supplementary file 5 — Source data Fig. 3 [file 44321_2024_146_MOESM5_ESM.zip › Fig. 3/Fig. 3A/Fig. 3A-Ub.tif]

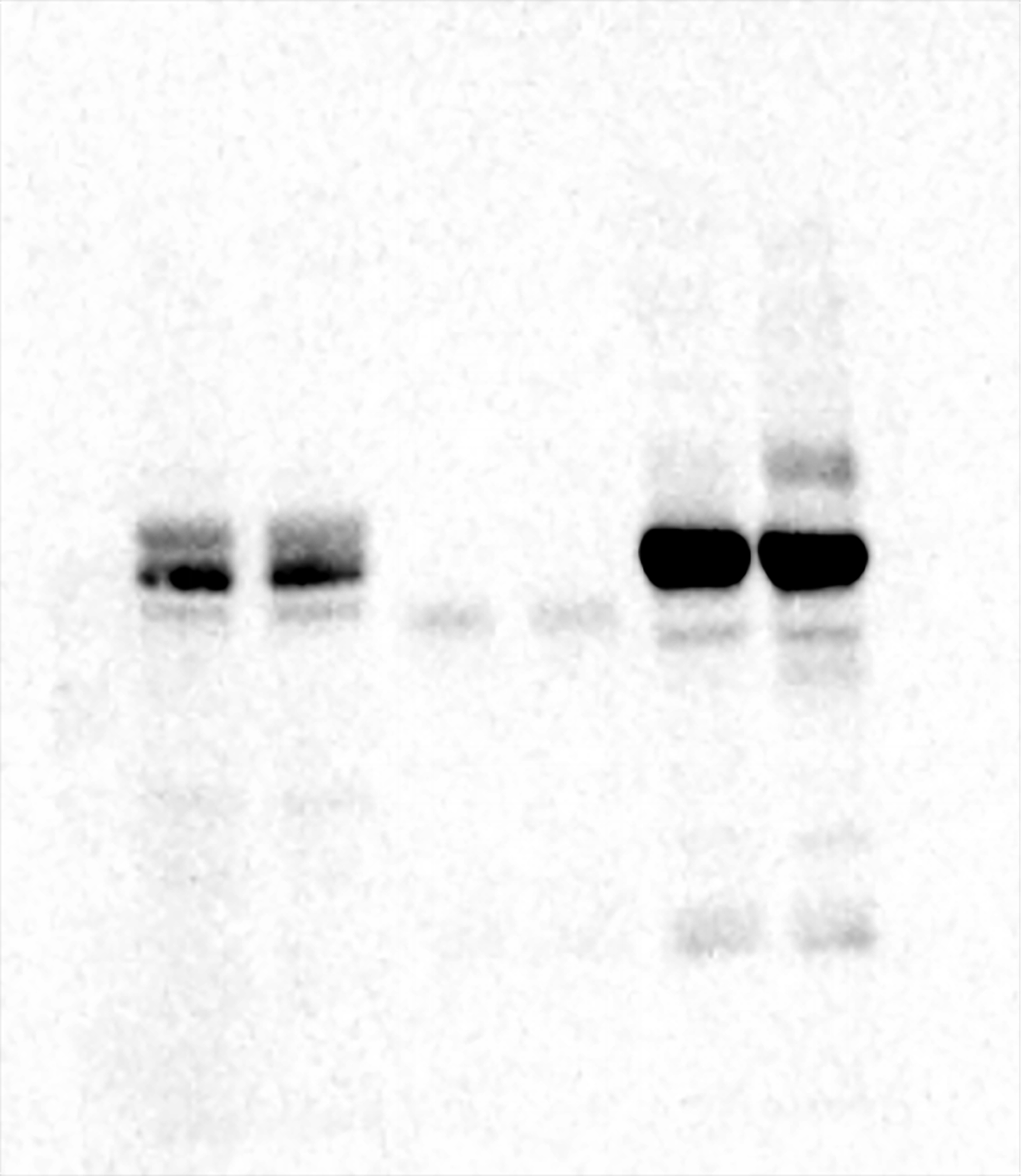

Supplement: Supplementary file 5 — Source data Fig. 3 [file 44321_2024_146_MOESM5_ESM.zip › Fig. 3/Fig. 3A/Fig. 3A-ub-K48-tau.tif]

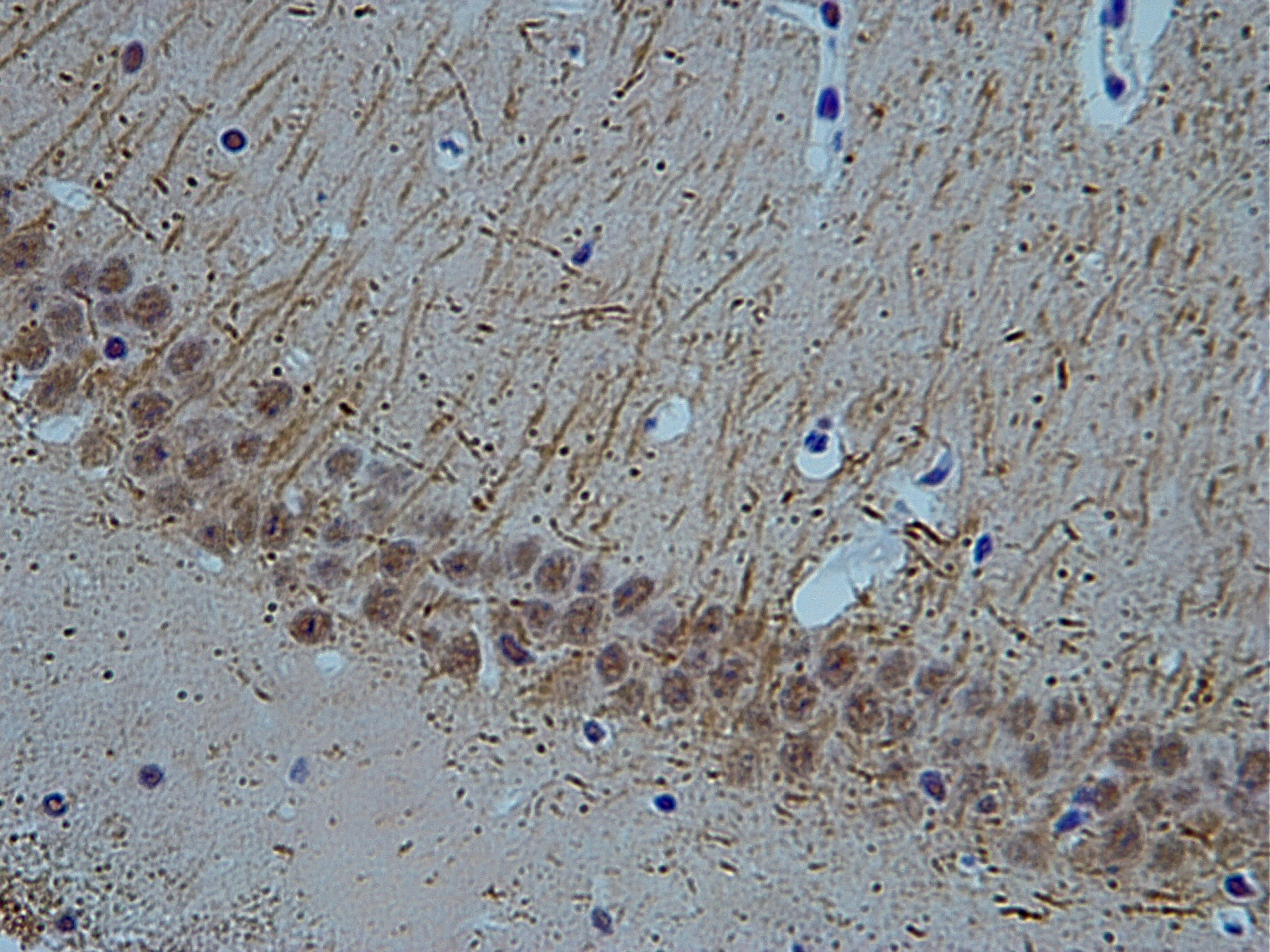

Supplement: Supplementary file 7 — Source data Fig. 4E [file 44321_2024_146_MOESM7_ESM.zip › Fig. 4E/3xTg-mice-ddH2O-IHC-1.tif]

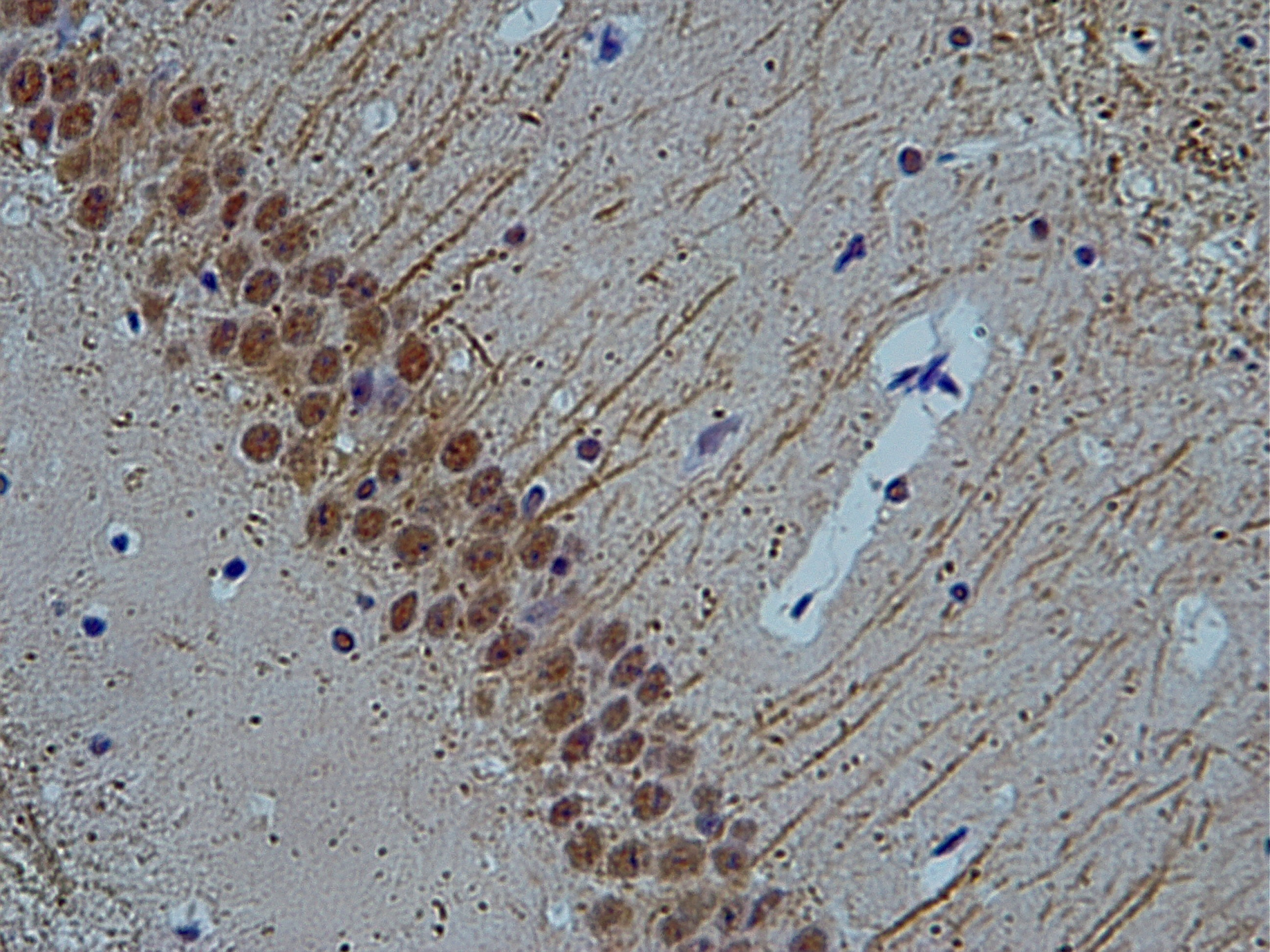

Supplement: Supplementary file 7 — Source data Fig. 4E [file 44321_2024_146_MOESM7_ESM.zip › Fig. 4E/3xTg-mice-ddH2O-IHC.tif]

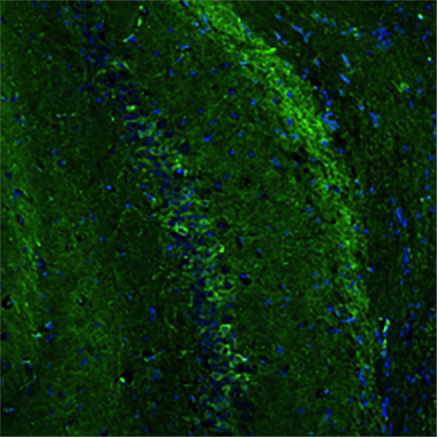

Supplement: Supplementary file 7 — Source data Fig. 4E [file 44321_2024_146_MOESM7_ESM.zip › Fig. 4E/3xTg-mice-ddH2O-IF-1.tif]

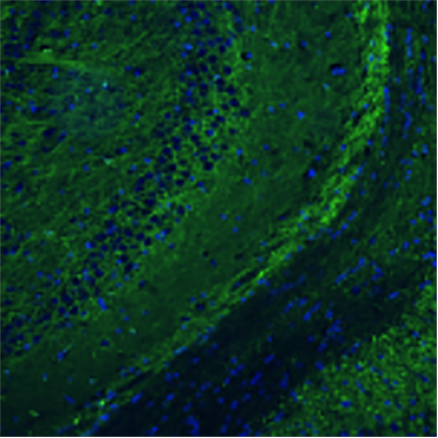

Supplement: Supplementary file 7 — Source data Fig. 4E [file 44321_2024_146_MOESM7_ESM.zip › Fig. 4E/3xTg-mice-IsoLiPro-IF-1.tif]

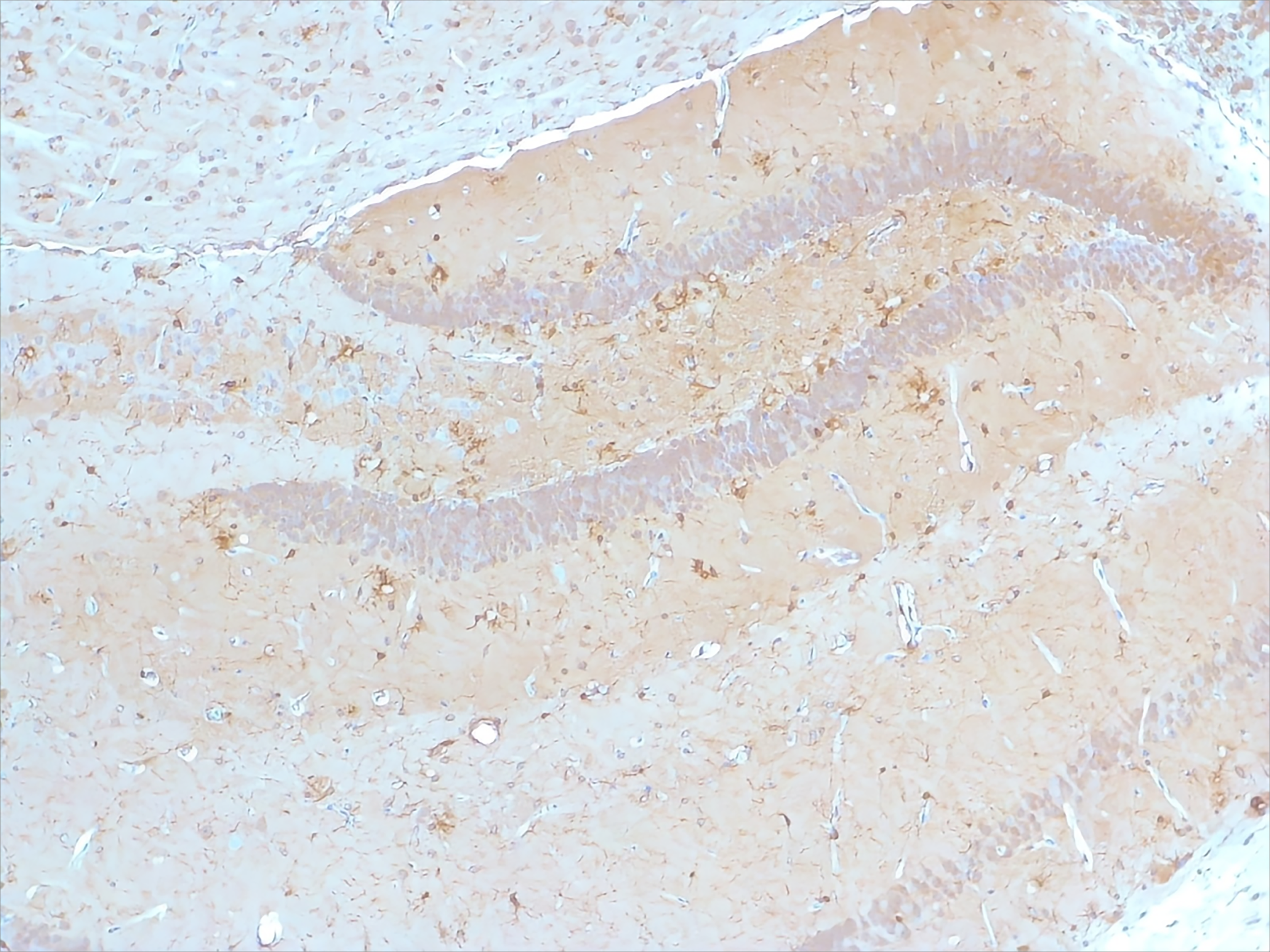

Supplement: Supplementary file 8 — Source data Fig. 5 [file 44321_2024_146_MOESM8_ESM.zip › Fig. 5/Fig. 5A/5xFAD mice-ddH2O-Iba1-1.tif]

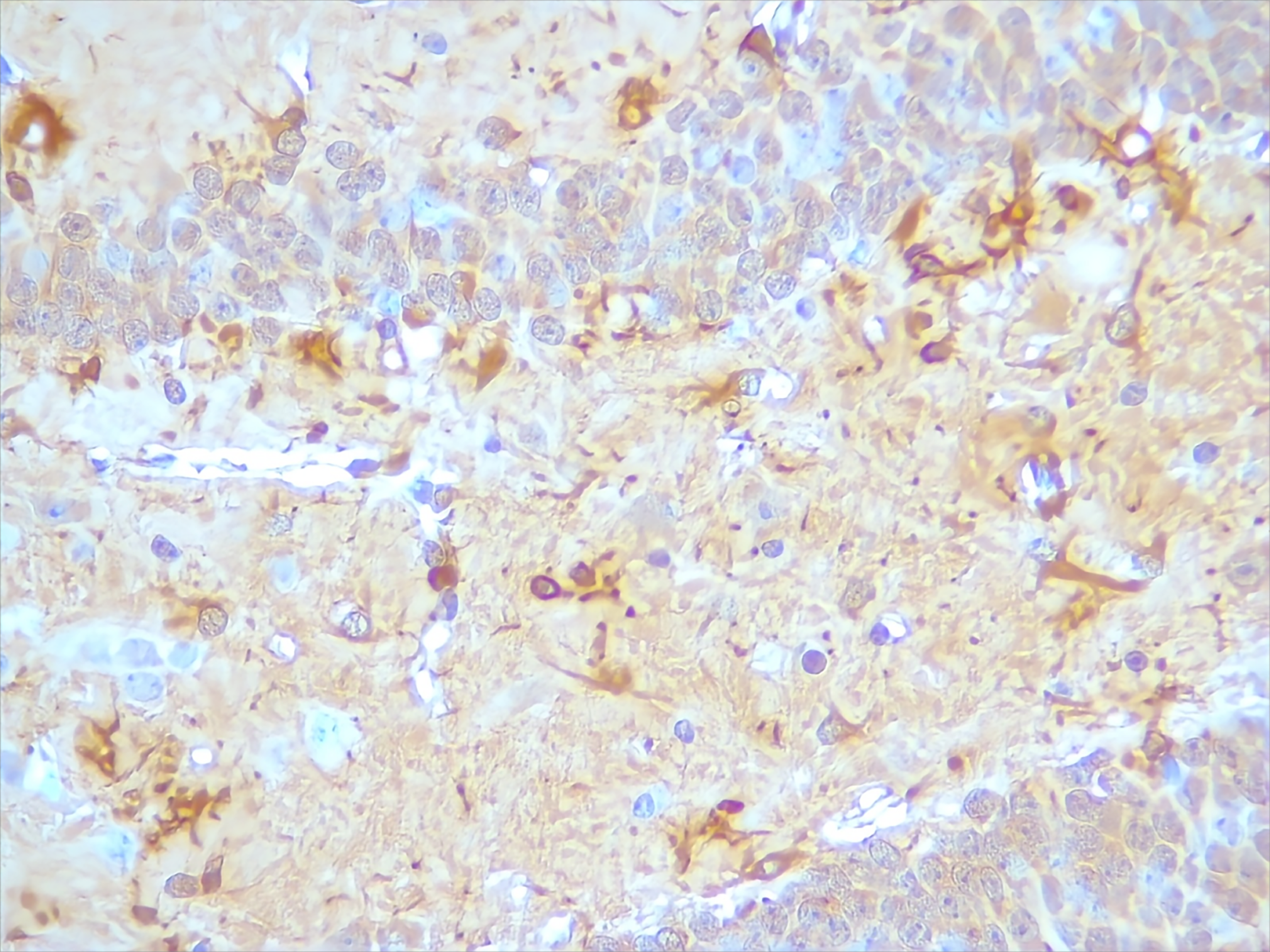

Supplement: Supplementary file 8 — Source data Fig. 5 [file 44321_2024_146_MOESM8_ESM.zip › Fig. 5/Fig. 5A/5xFAD mice-ddH2O-Iba1-2.tif]

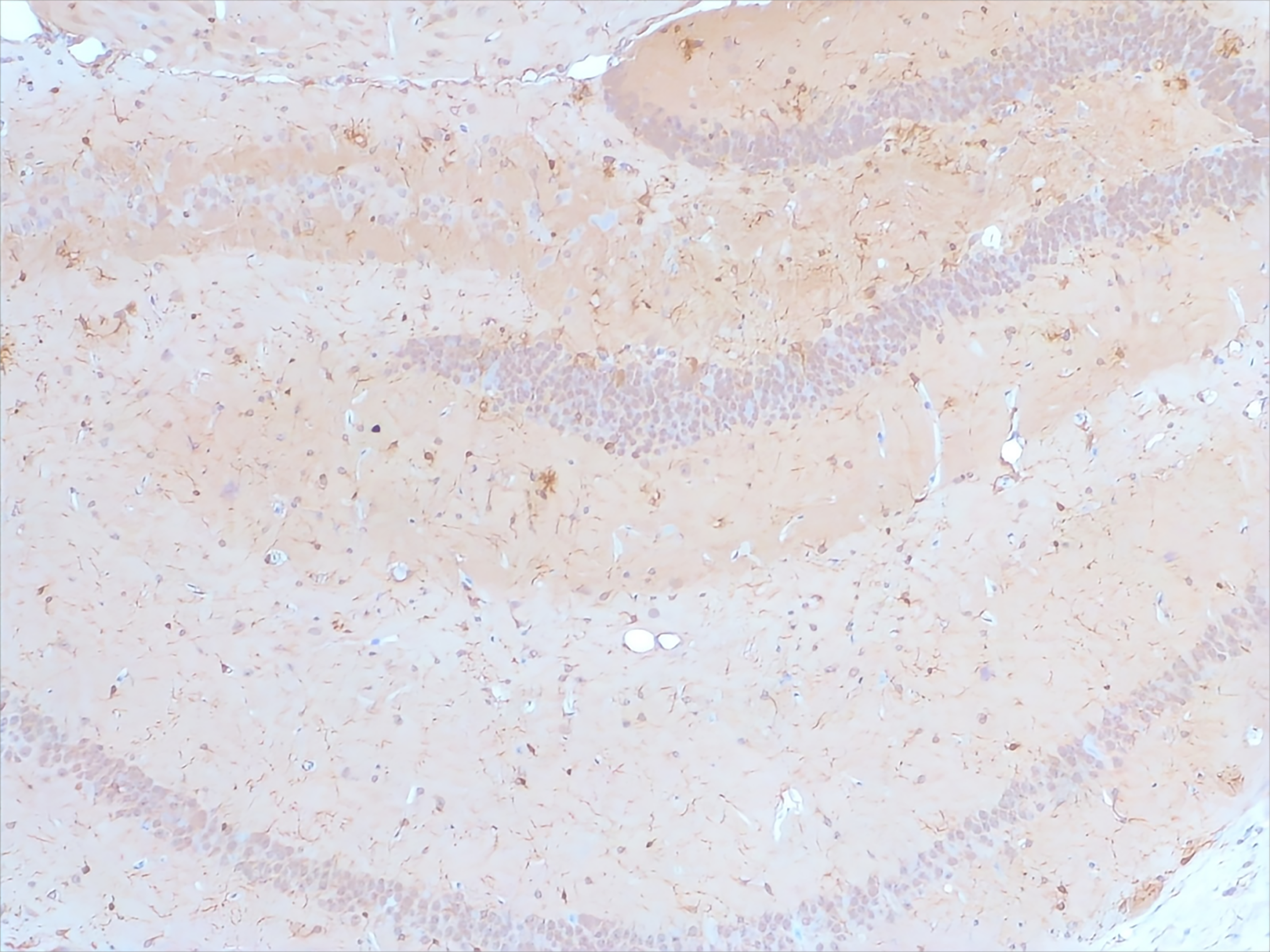

Supplement: Supplementary file 8 — Source data Fig. 5 [file 44321_2024_146_MOESM8_ESM.zip › Fig. 5/Fig. 5A/5xFAD mice-IsoLiPro-Iba1-1.tif]

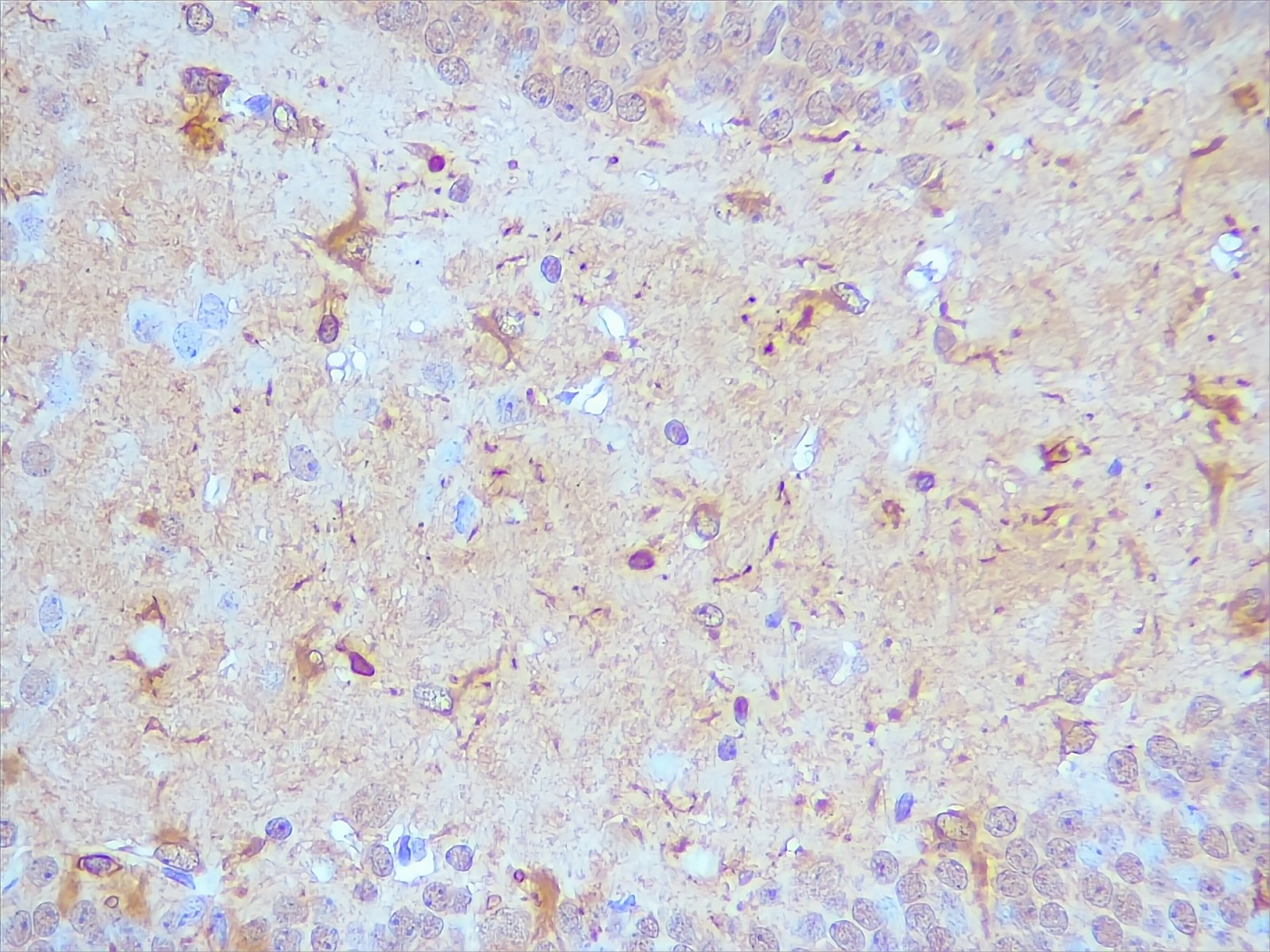

Supplement: Supplementary file 8 — Source data Fig. 5 [file 44321_2024_146_MOESM8_ESM.zip › Fig. 5/Fig. 5A/5xFAD mice-IsoLiPro-Iba1-2.tif]

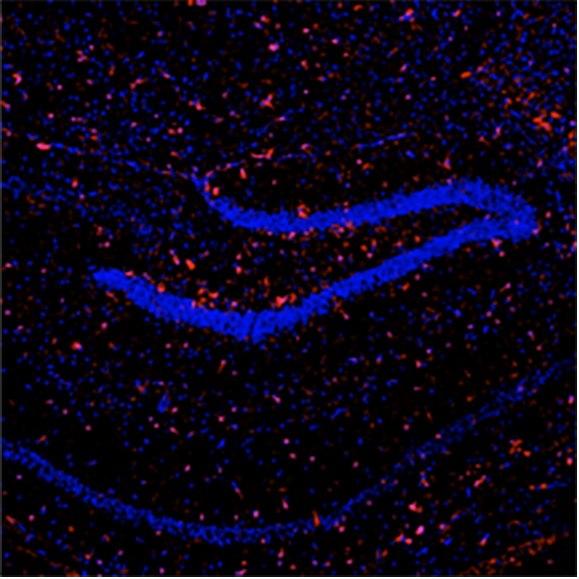

Supplement: Supplementary file 8 — Source data Fig. 5 [file 44321_2024_146_MOESM8_ESM.zip › Fig. 5/Fig. 5A/5xFAD mice-IsoLiPro-Iba1-IF.tif]

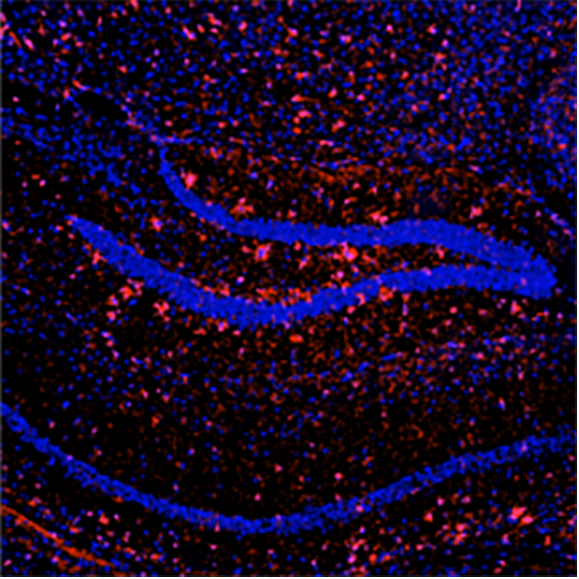

Supplement: Supplementary file 8 — Source data Fig. 5 [file 44321_2024_146_MOESM8_ESM.zip › Fig. 5/Fig. 5A/5xFAD mice-ddH2O-Iba1-IF.tif]

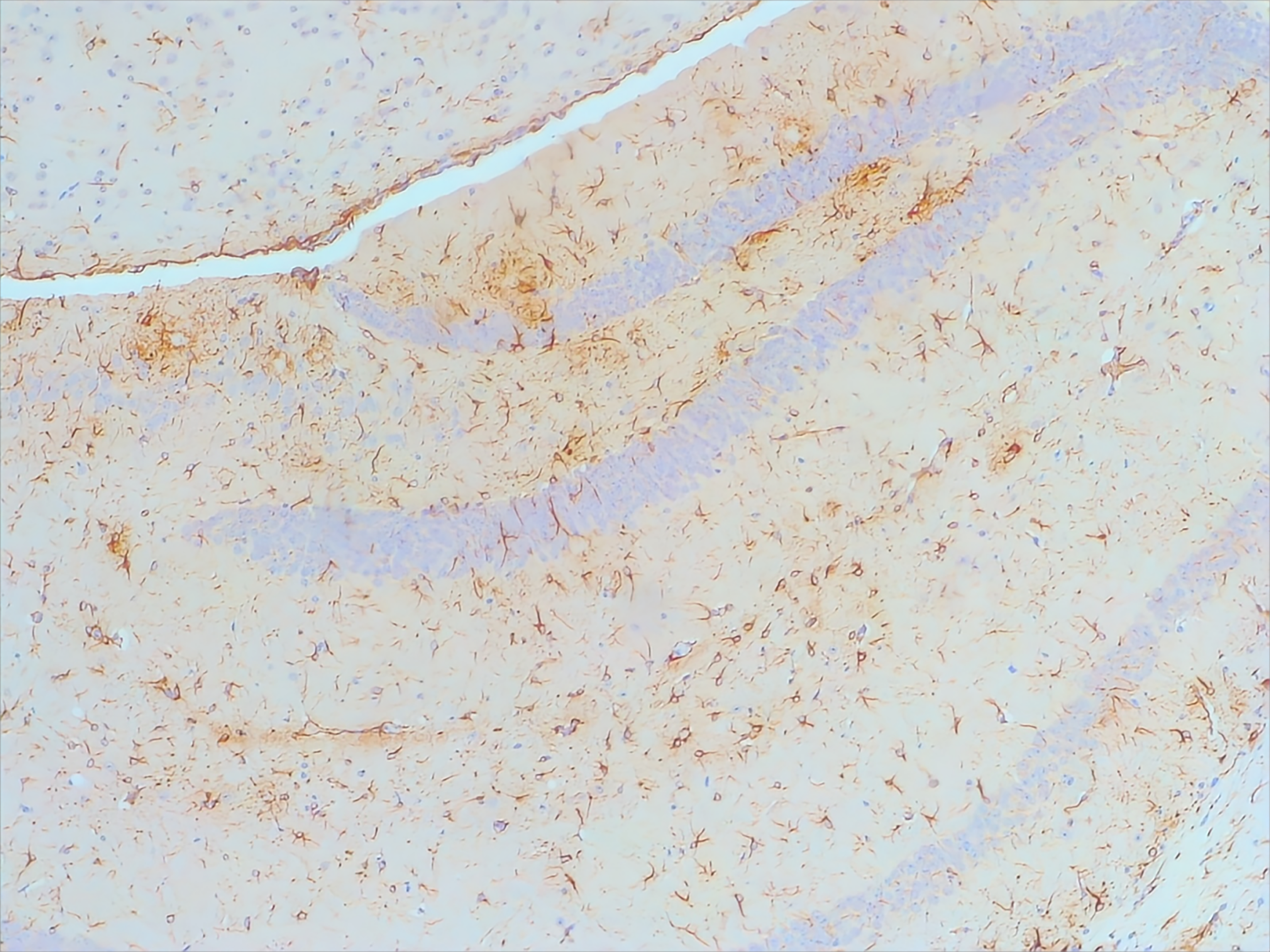

Supplement: Supplementary file 8 — Source data Fig. 5 [file 44321_2024_146_MOESM8_ESM.zip › Fig. 5/Fig. 5B/5xFAD mice-IsoLiPro-GFAP-1.tif]

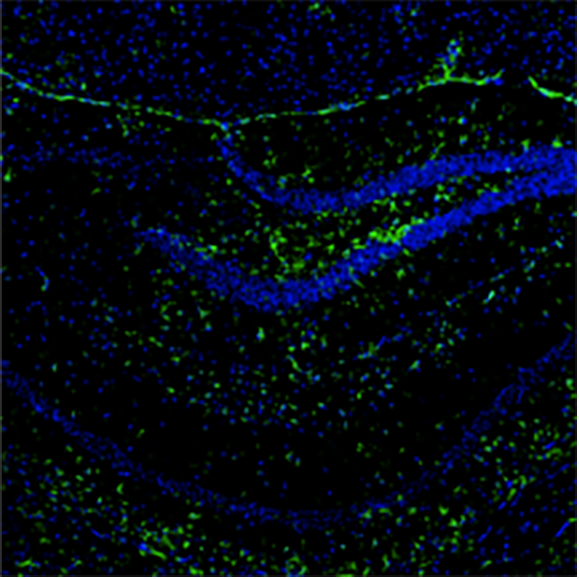

Supplement: Supplementary file 8 — Source data Fig. 5 [file 44321_2024_146_MOESM8_ESM.zip › Fig. 5/Fig. 5B/5xFAD mice-ddH2O-GFAP-IF.tif]

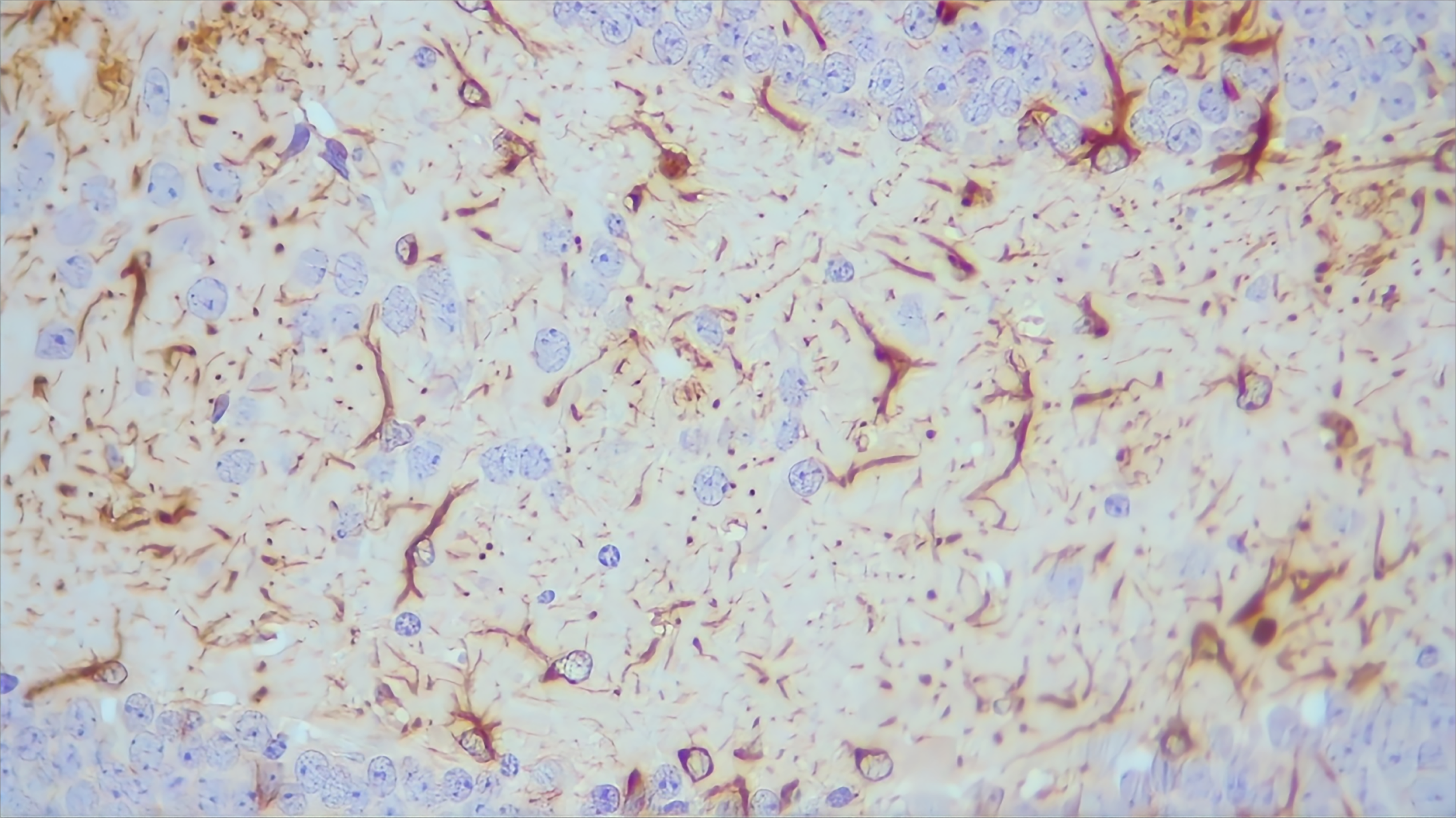

Supplement: Supplementary file 8 — Source data Fig. 5 [file 44321_2024_146_MOESM8_ESM.zip › Fig. 5/Fig. 5B/5xFAD mice-IsoLiPro-GFAP-2.tif]

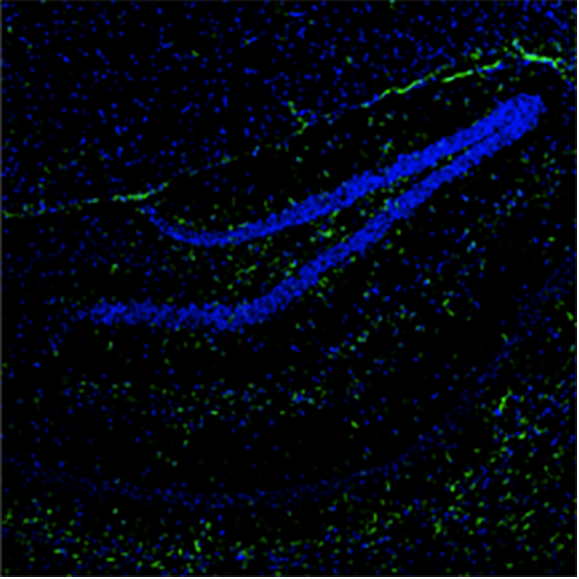

Supplement: Supplementary file 8 — Source data Fig. 5 [file 44321_2024_146_MOESM8_ESM.zip › Fig. 5/Fig. 5B/5xFAD mice-IsoLiPro-GFAP-IF.tif]

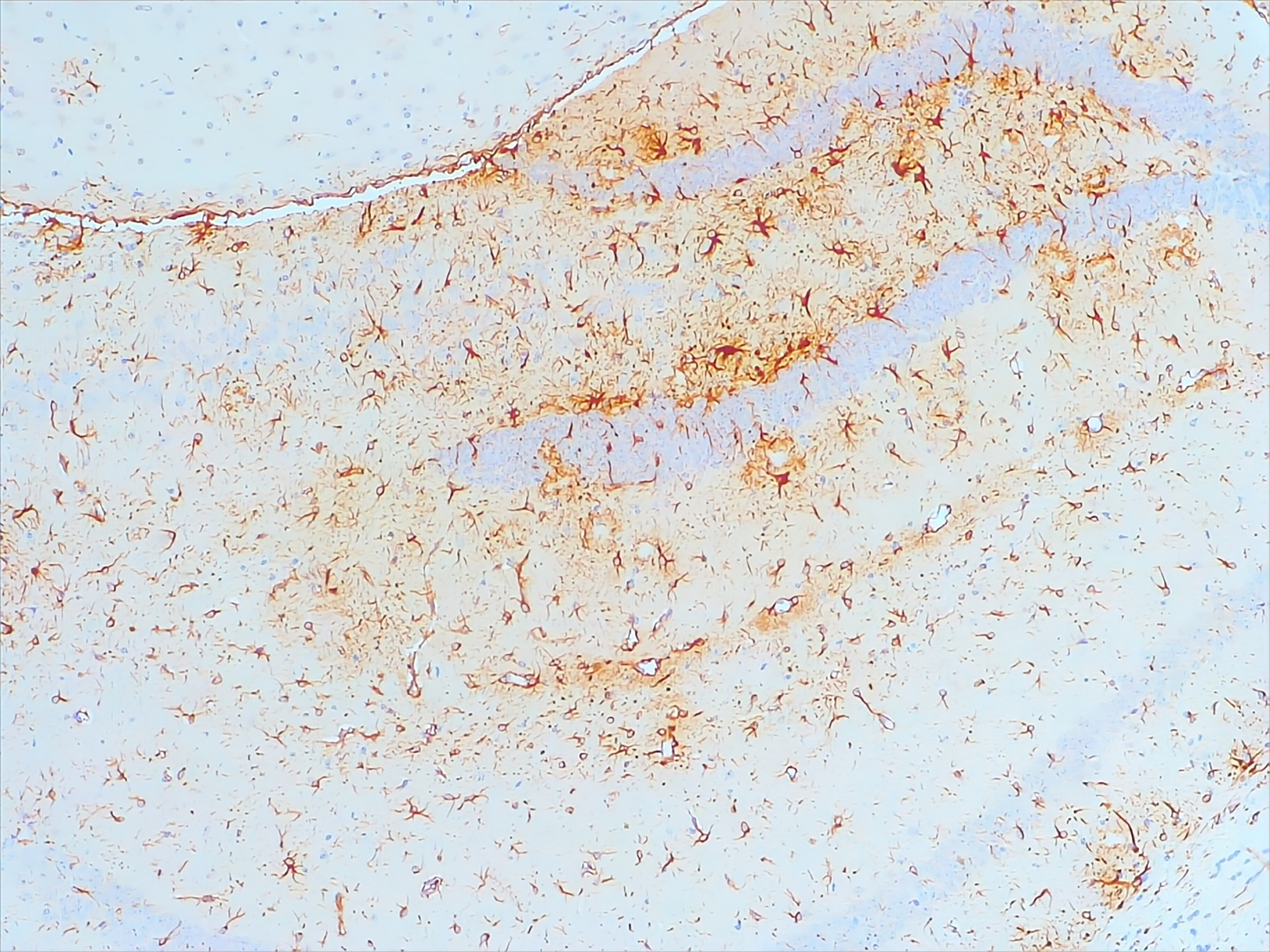

Supplement: Supplementary file 8 — Source data Fig. 5 [file 44321_2024_146_MOESM8_ESM.zip › Fig. 5/Fig. 5B/5xFAD mice-ddH2O-GFAP-1.tif]

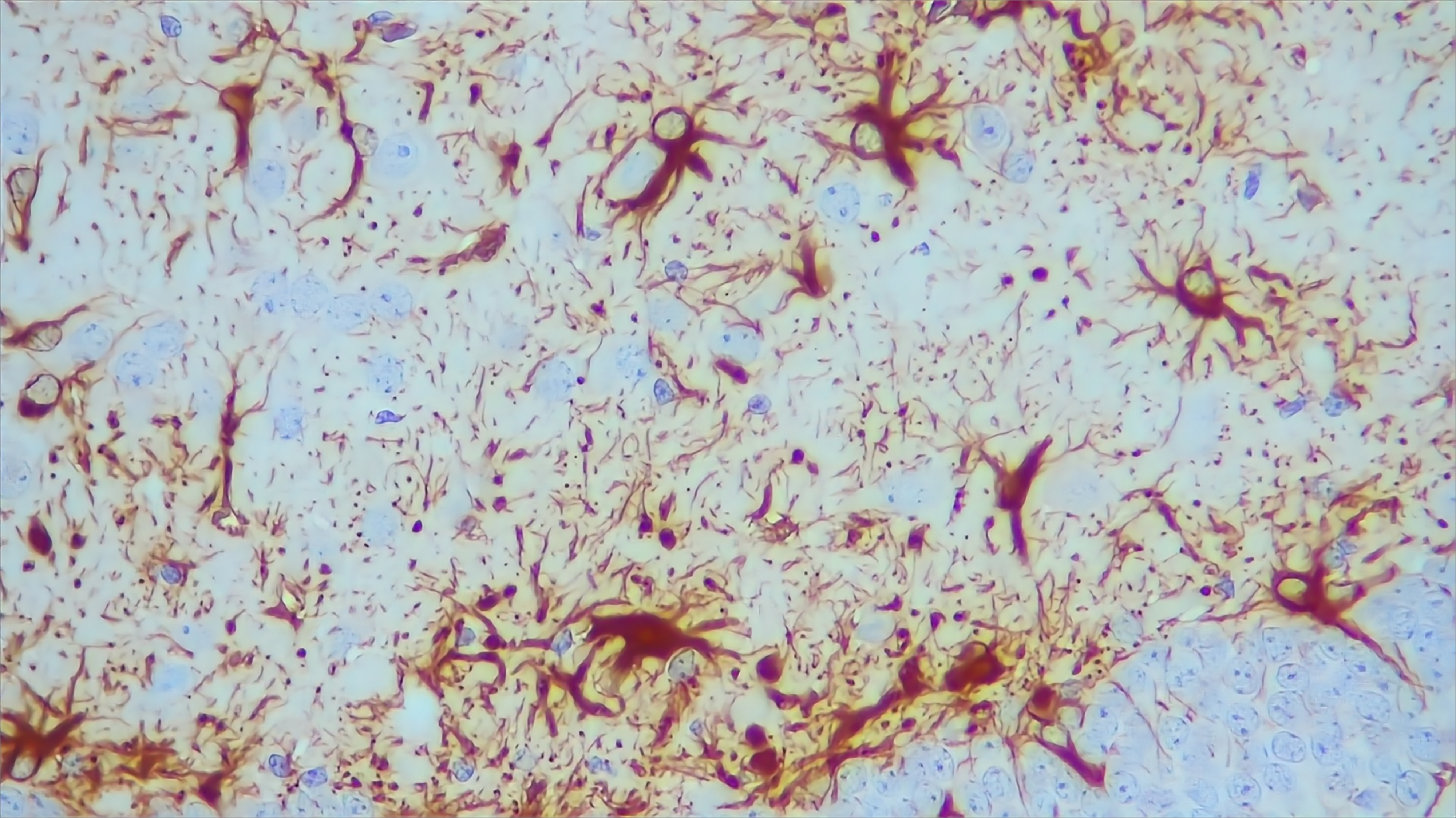

Supplement: Supplementary file 8 — Source data Fig. 5 [file 44321_2024_146_MOESM8_ESM.zip › Fig. 5/Fig. 5B/5xFAD mice-ddH2O-GFAP-2.tif]

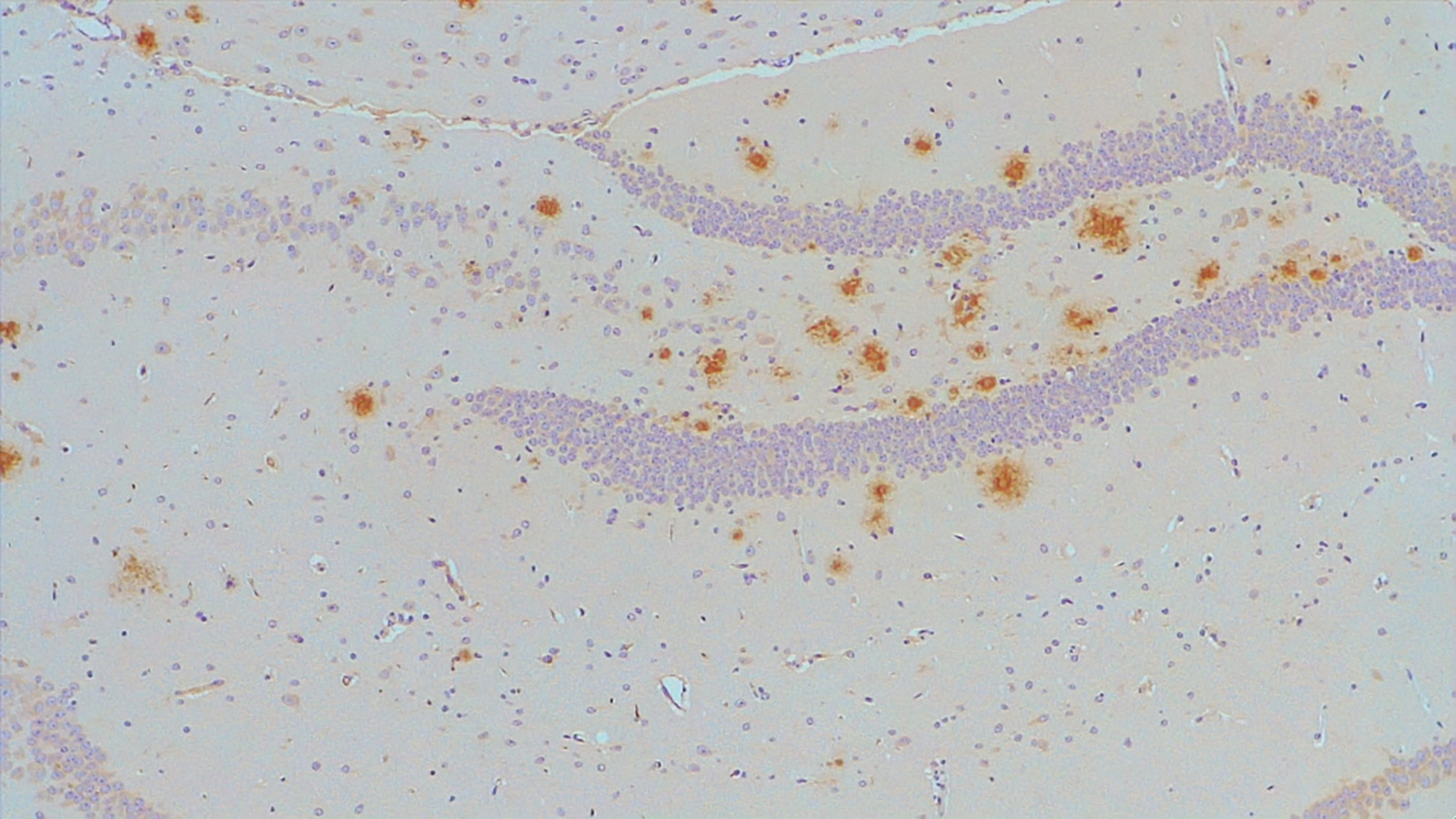

Supplement: Supplementary file 8 — Source data Fig. 5 [file 44321_2024_146_MOESM8_ESM.zip › Fig. 5/Fig. 5E/5xFAD mice-ddH2O-4G8.tif]

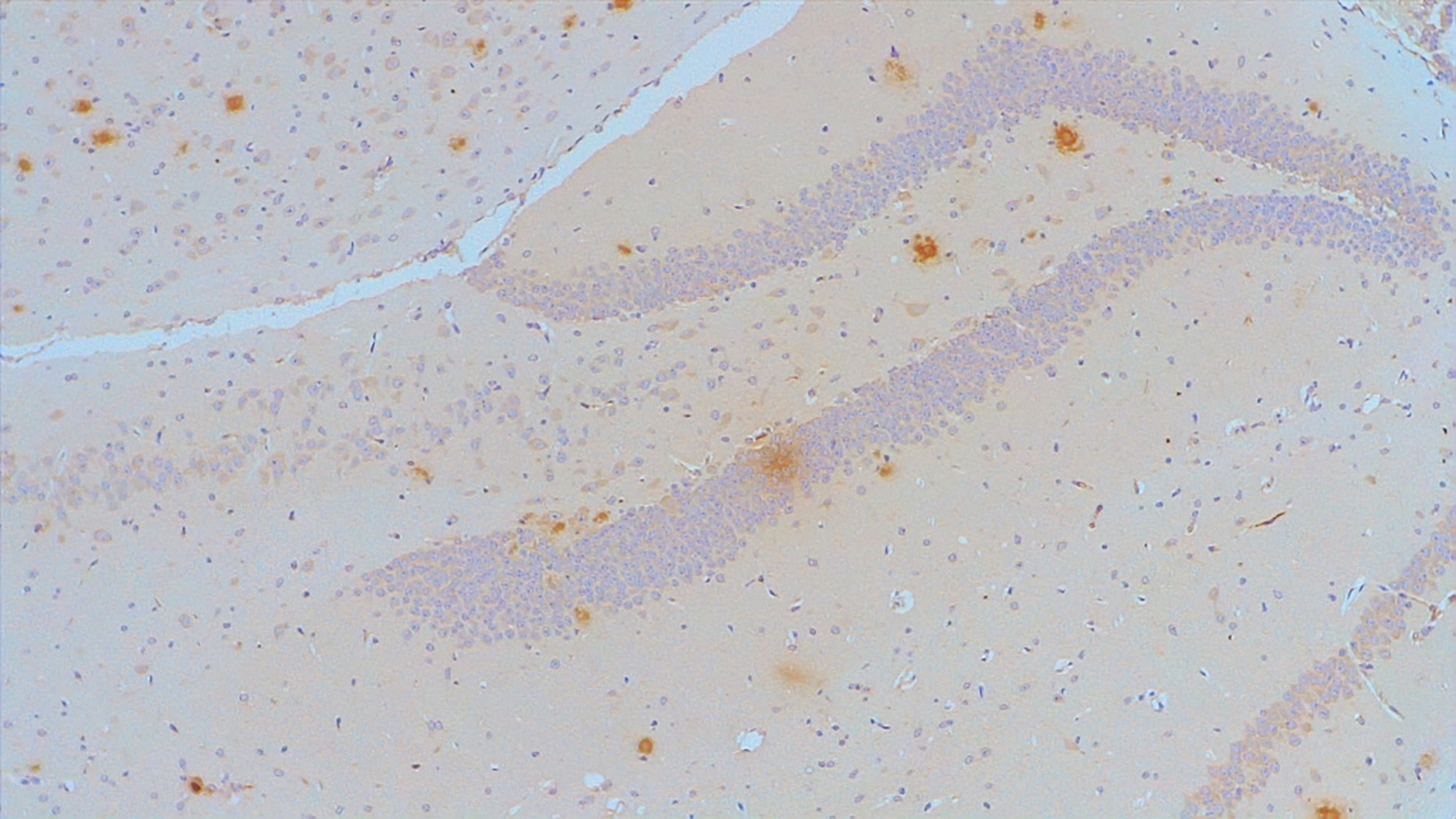

Supplement: Supplementary file 8 — Source data Fig. 5 [file 44321_2024_146_MOESM8_ESM.zip › Fig. 5/Fig. 5E/5xFAD mice-IsoLiPro-4G8.tif]

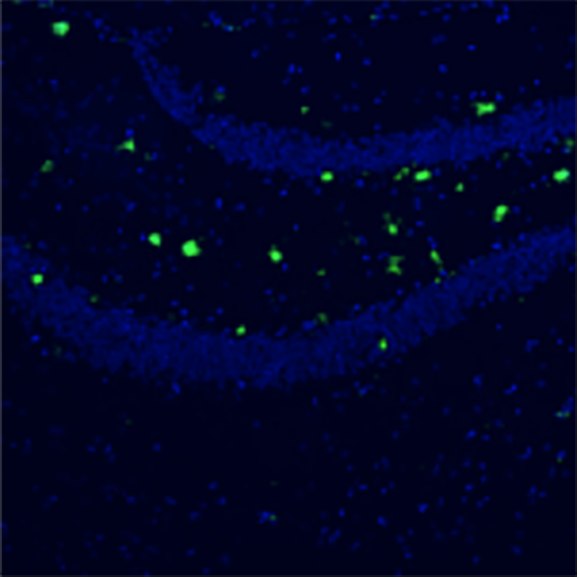

Supplement: Supplementary file 8 — Source data Fig. 5 [file 44321_2024_146_MOESM8_ESM.zip › Fig. 5/Fig. 5E/5xFAD mice-ddH2O-4G8-IF.tif]

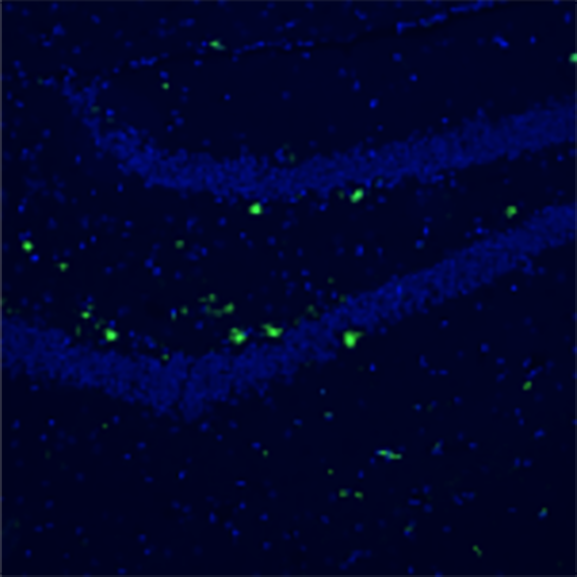

Supplement: Supplementary file 8 — Source data Fig. 5 [file 44321_2024_146_MOESM8_ESM.zip › Fig. 5/Fig. 5E/5xFAD mice-IsoLiPro-4G8-IF.tif]

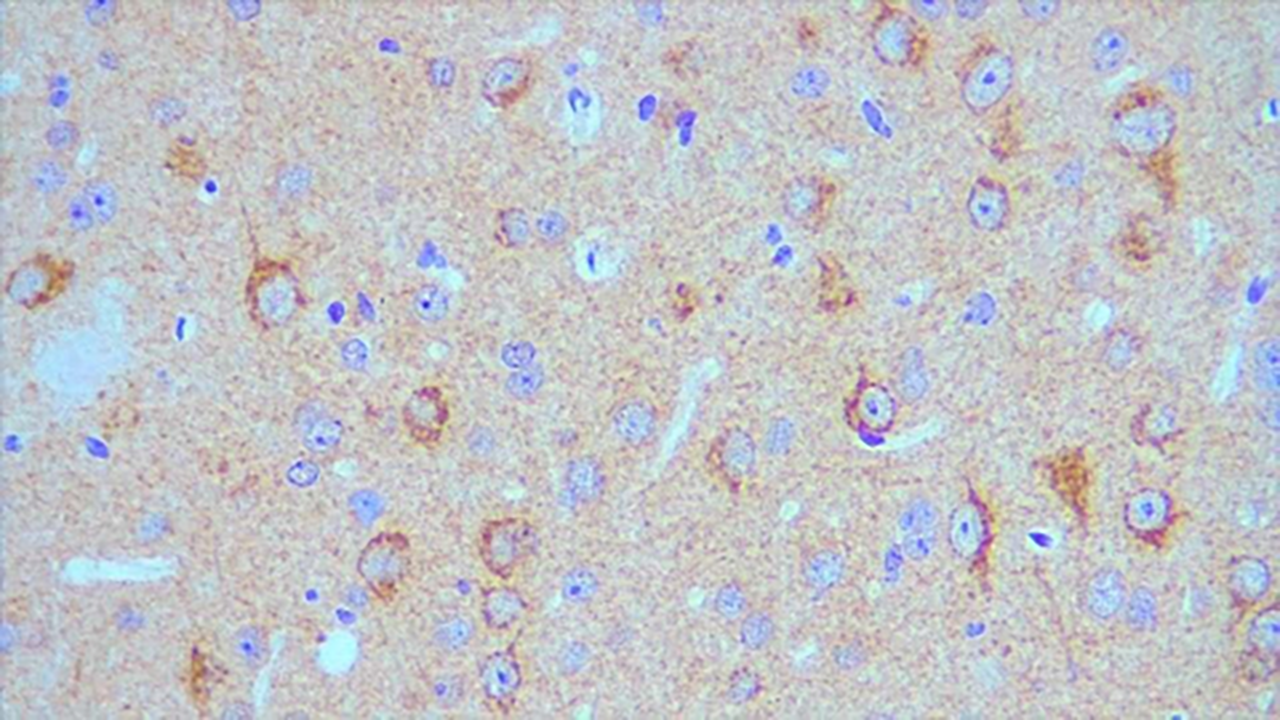

Supplement: Supplementary file 9 — Source data Fig. 6 [file 44321_2024_146_MOESM9_ESM.zip › Source data Fig. 6 (MOESM9)/Fig. 6A/3xTg-AD mice-ddH2O-PSD95.tif]

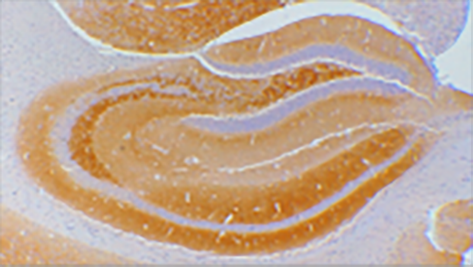

Supplement: Supplementary file 9 — Source data Fig. 6 [file 44321_2024_146_MOESM9_ESM.zip › Source data Fig. 6 (MOESM9)/Fig. 6A/3xTg-AD mice-ddH2O-SYN-1.tif]

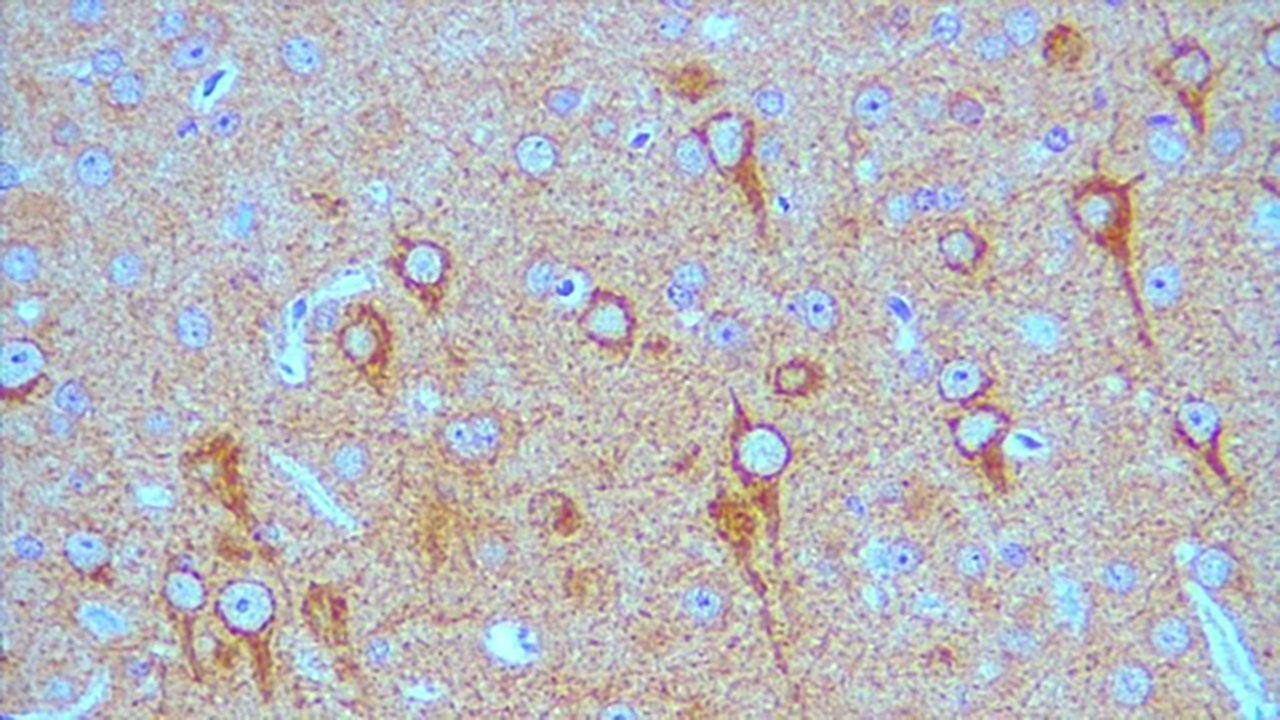

Supplement: Supplementary file 9 — Source data Fig. 6 [file 44321_2024_146_MOESM9_ESM.zip › Source data Fig. 6 (MOESM9)/Fig. 6A/3xTg-AD mice-IsoLiPro-PSD95.tif]

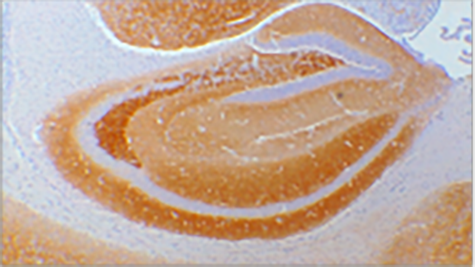

Supplement: Supplementary file 9 — Source data Fig. 6 [file 44321_2024_146_MOESM9_ESM.zip › Source data Fig. 6 (MOESM9)/Fig. 6A/3xTg-AD mice-IsoLiPro-SYN-1.tif]

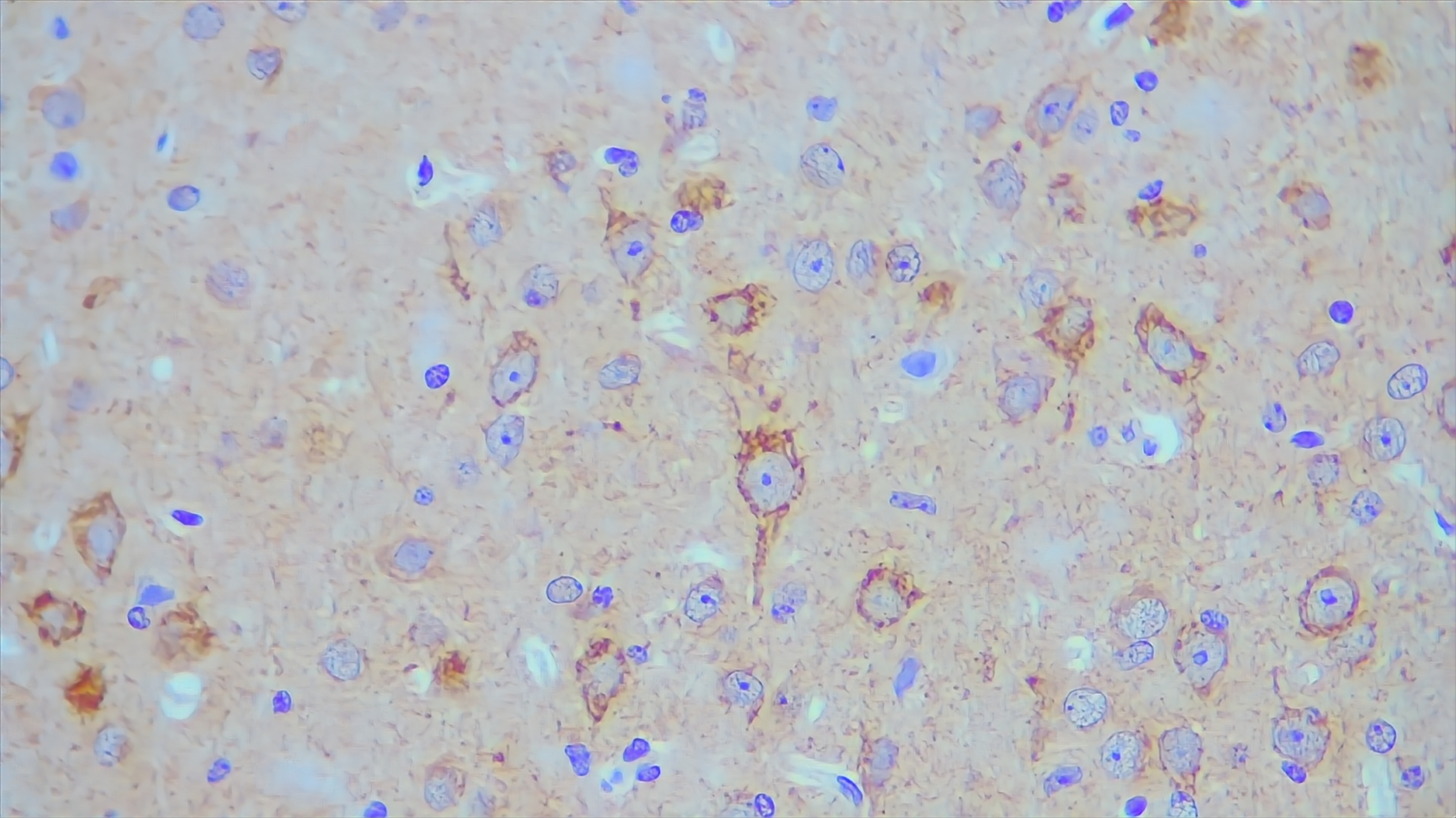

Supplement: Supplementary file 9 — Source data Fig. 6 [file 44321_2024_146_MOESM9_ESM.zip › Source data Fig. 6 (MOESM9)/Fig. 6C/5xFAD mice-ddH2O-PSD95.tif]

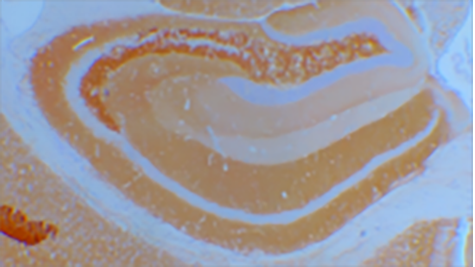

Supplement: Supplementary file 9 — Source data Fig. 6 [file 44321_2024_146_MOESM9_ESM.zip › Source data Fig. 6 (MOESM9)/Fig. 6C/5xFAD mice-ddH2O-SYN-1.tif]

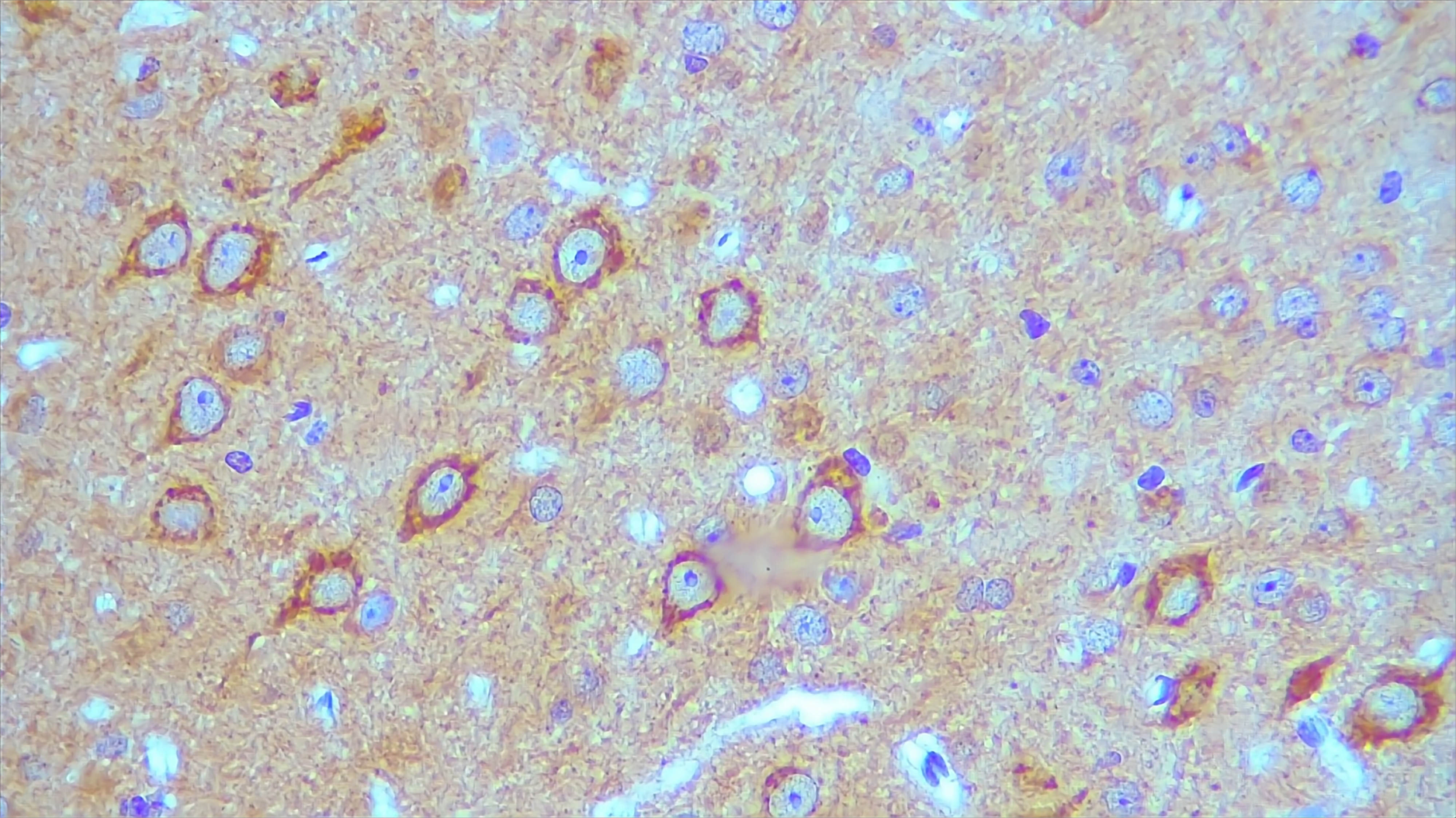

Supplement: Supplementary file 9 — Source data Fig. 6 [file 44321_2024_146_MOESM9_ESM.zip › Source data Fig. 6 (MOESM9)/Fig. 6C/5xFAD mice-IsoLiPro-PSD95.tif]

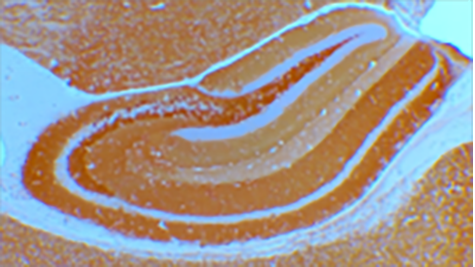

Supplement: Supplementary file 9 — Source data Fig. 6 [file 44321_2024_146_MOESM9_ESM.zip › Source data Fig. 6 (MOESM9)/Fig. 6C/5xFAD mice-IsoLiPro-SYN-1.tif]

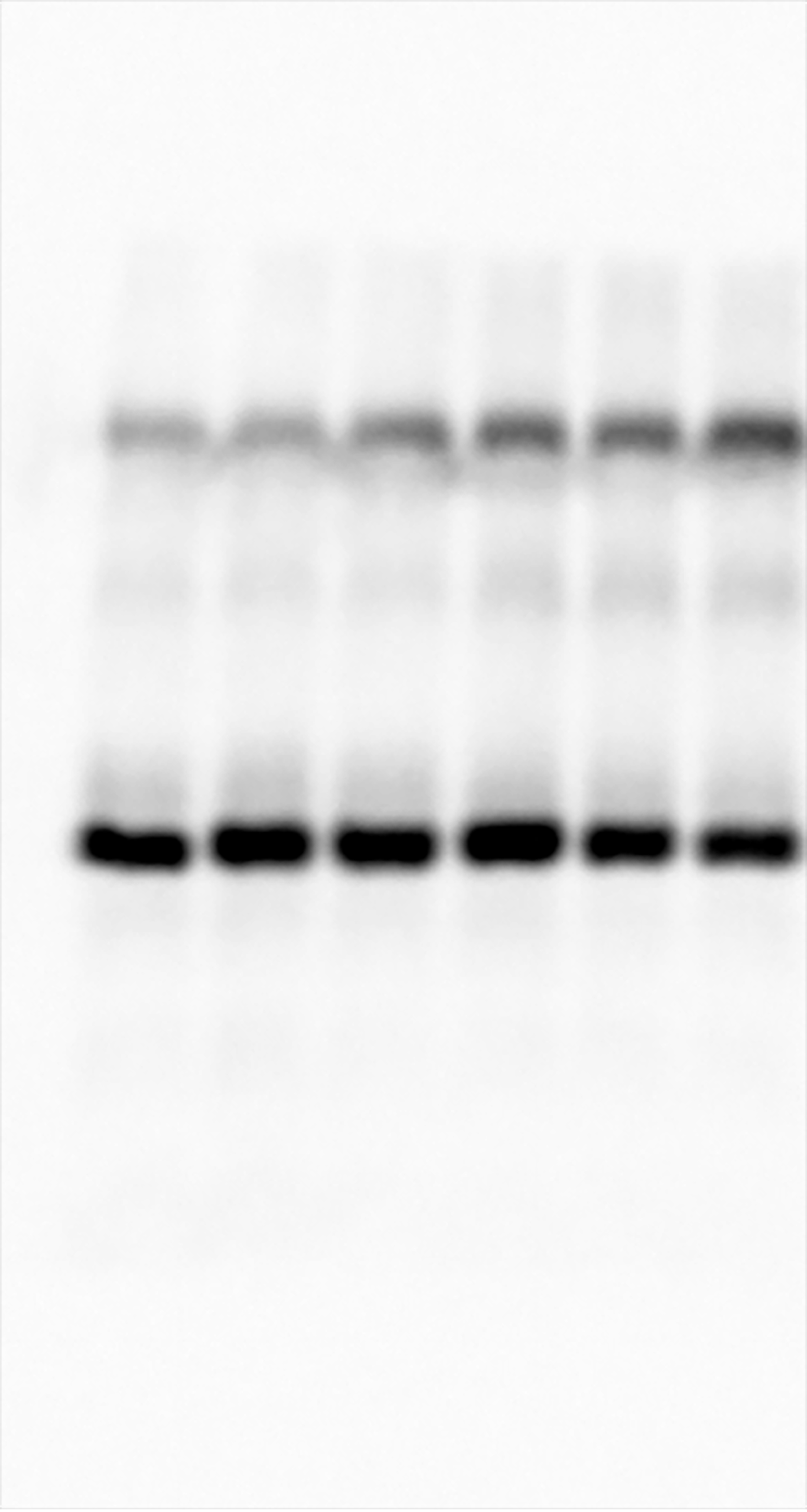

Supplement: Supplementary file 9 — Source data Fig. 6 [file 44321_2024_146_MOESM9_ESM.zip › Source data Fig. 6 (MOESM9)/Fig. 6E/3xTg-AD-PSD95-GAPDH-insolution.tif]

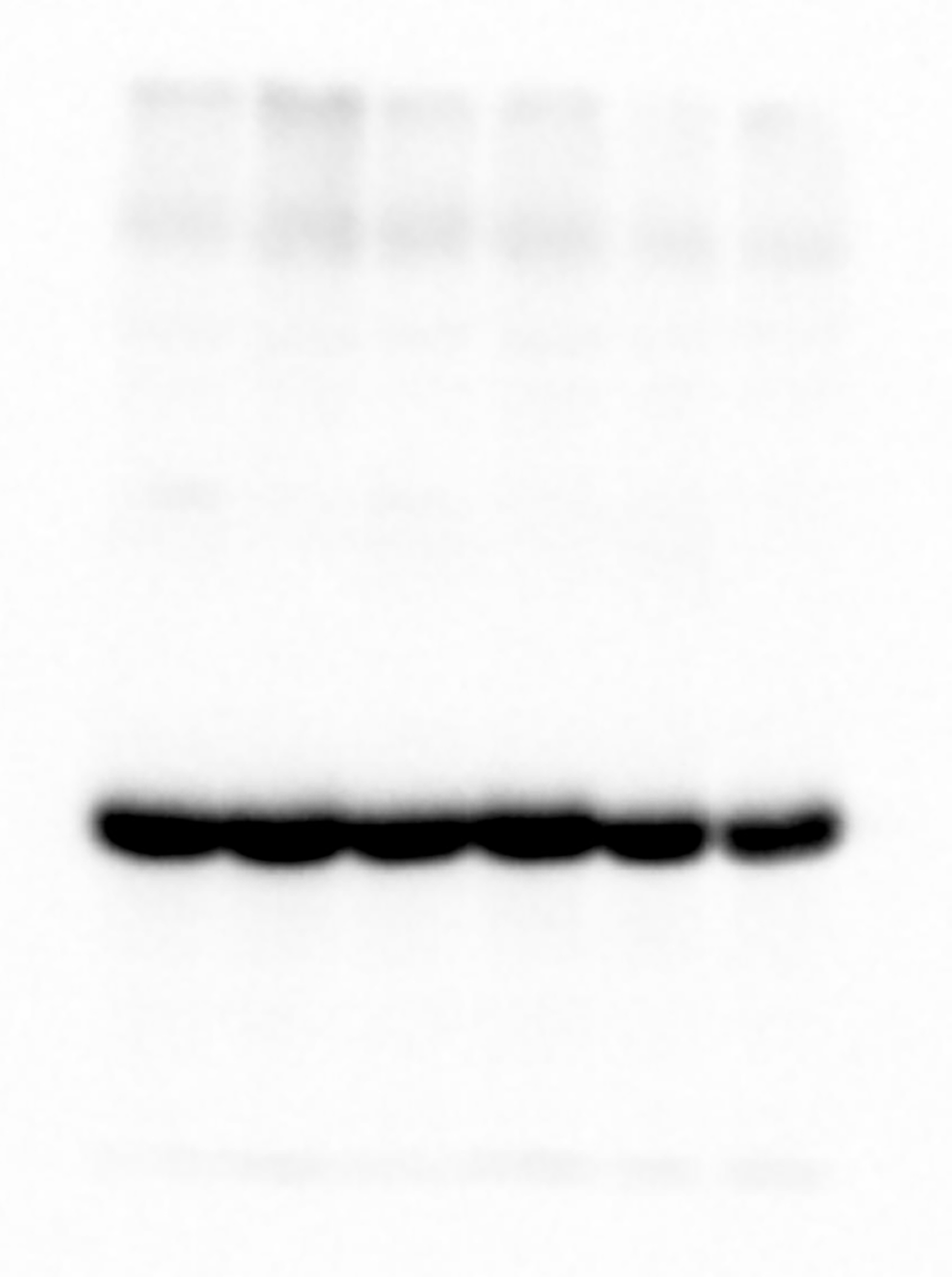

Supplement: Supplementary file 9 — Source data Fig. 6 [file 44321_2024_146_MOESM9_ESM.zip › Source data Fig. 6 (MOESM9)/Fig. 6E/3xTg-AD-PSD95-GAPDH-solution.tif]

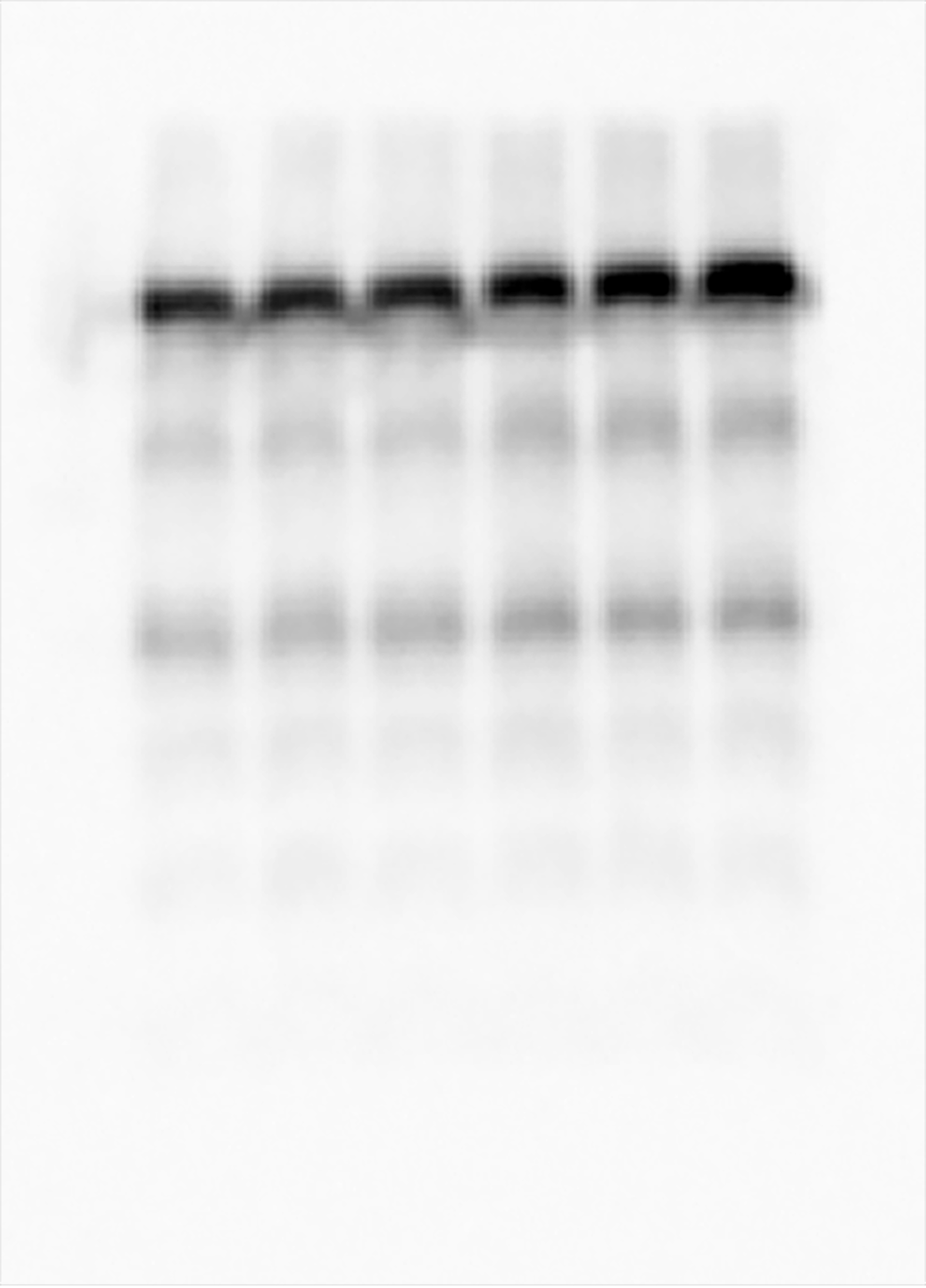

Supplement: Supplementary file 9 — Source data Fig. 6 [file 44321_2024_146_MOESM9_ESM.zip › Source data Fig. 6 (MOESM9)/Fig. 6E/3xTg-AD-PSD95-insolution.tif]

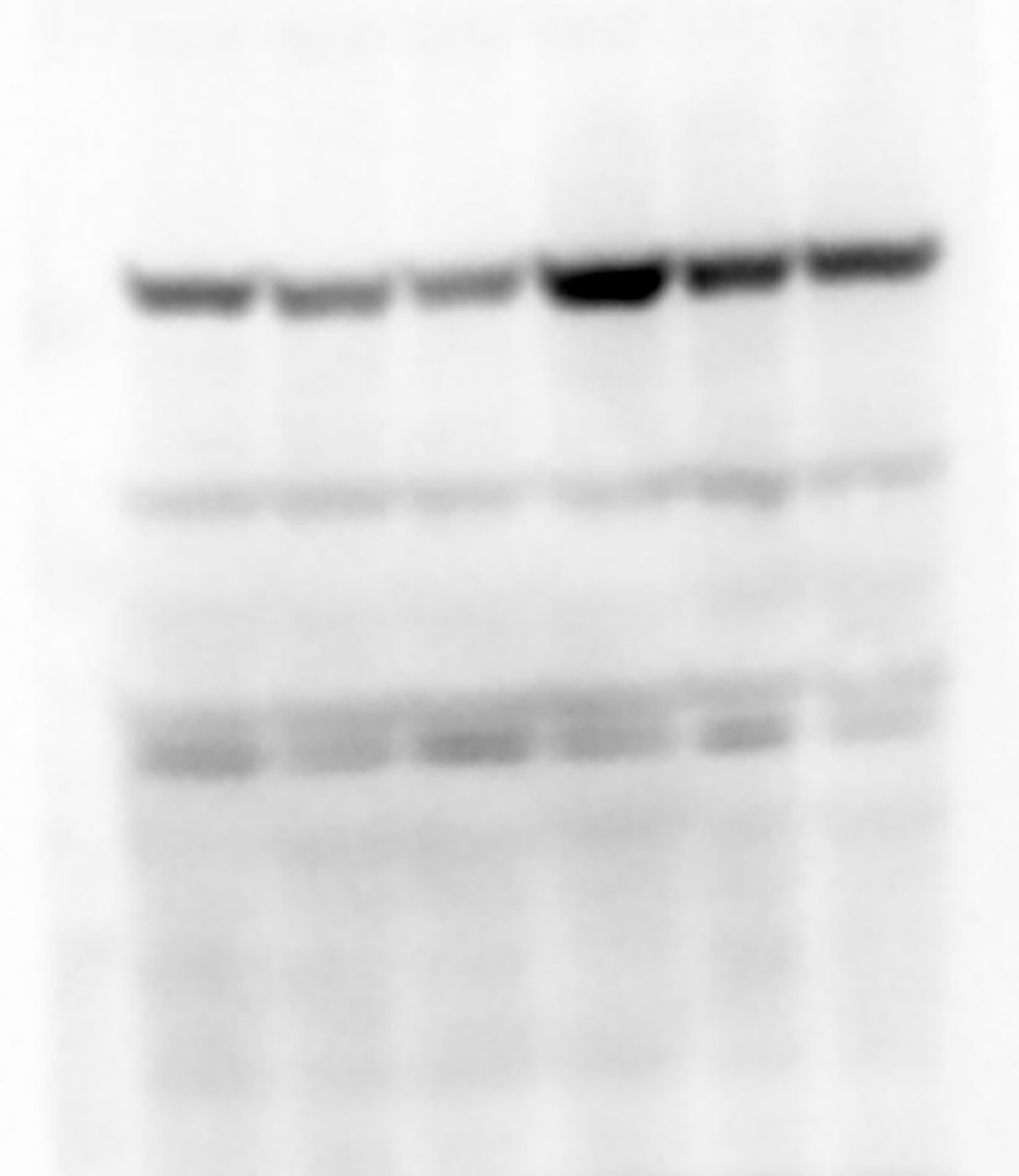

Supplement: Supplementary file 9 — Source data Fig. 6 [file 44321_2024_146_MOESM9_ESM.zip › Source data Fig. 6 (MOESM9)/Fig. 6E/3xTg-AD-PSD95-solution.tif]

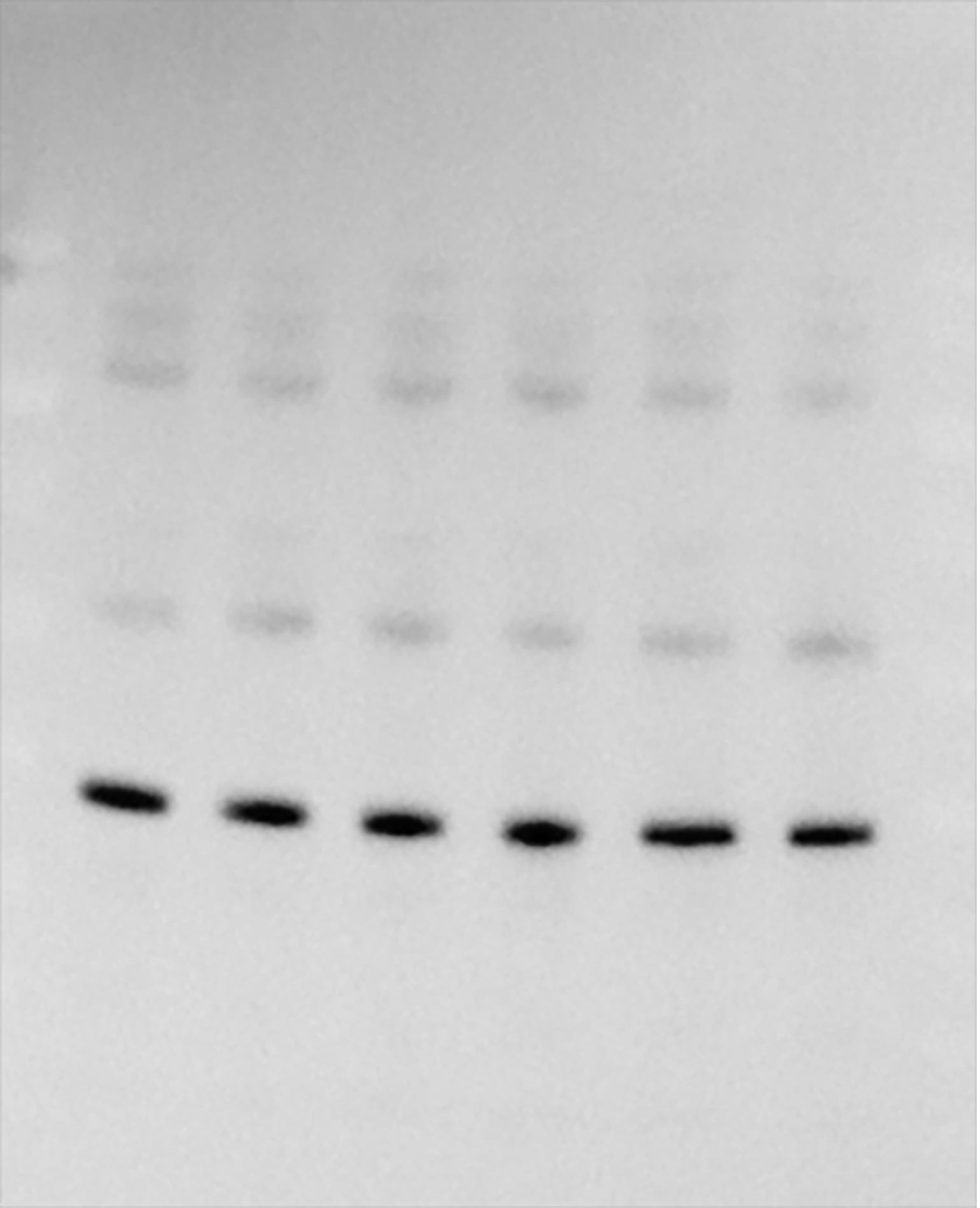

Supplement: Supplementary file 9 — Source data Fig. 6 [file 44321_2024_146_MOESM9_ESM.zip › Source data Fig. 6 (MOESM9)/Fig. 6E/3xTg-AD-SYN1-GAPDH-insolution.tif]

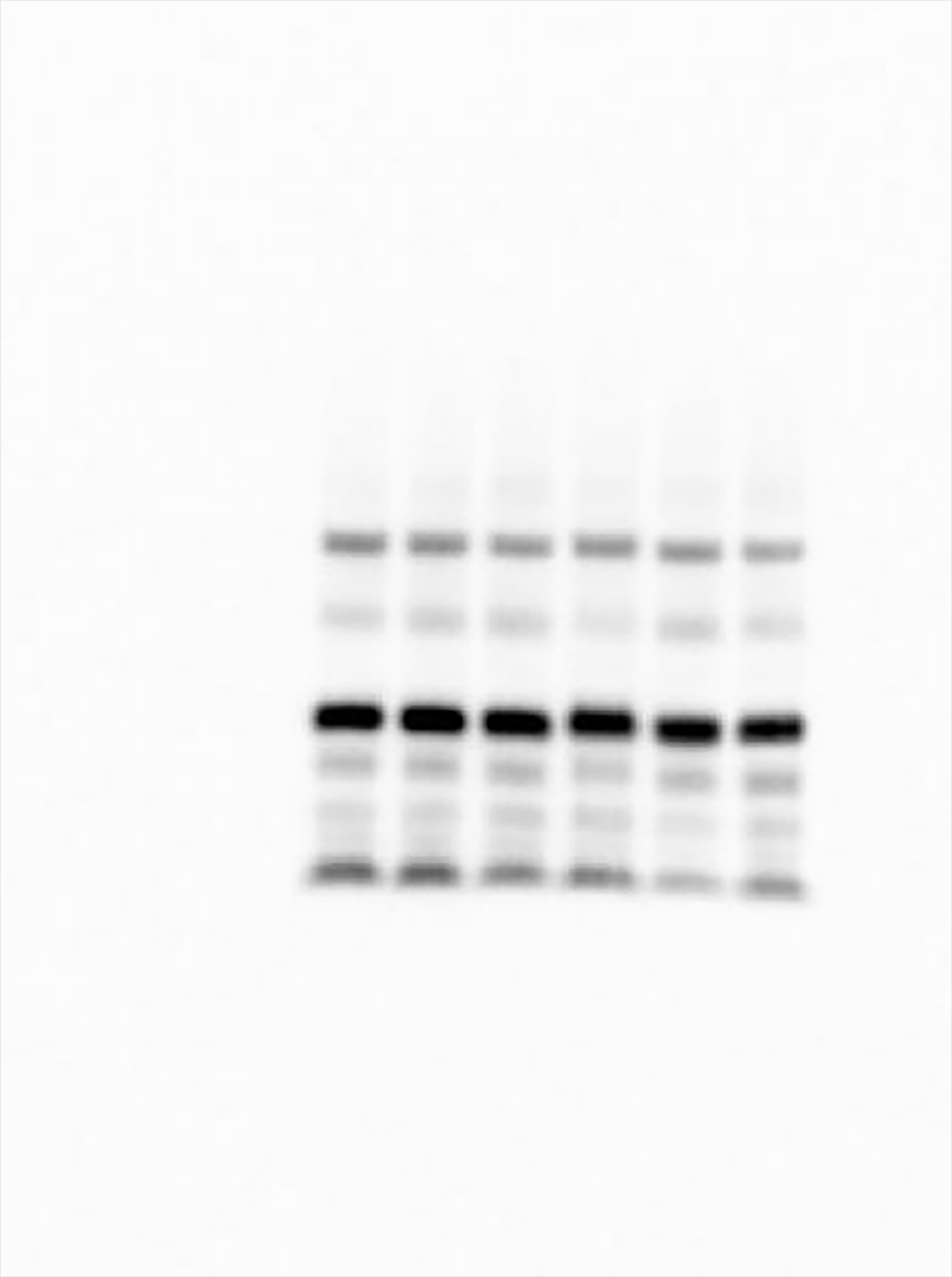

Supplement: Supplementary file 9 — Source data Fig. 6 [file 44321_2024_146_MOESM9_ESM.zip › Source data Fig. 6 (MOESM9)/Fig. 6E/3xTg-AD-SYN1-GAPDH-solution.tif]

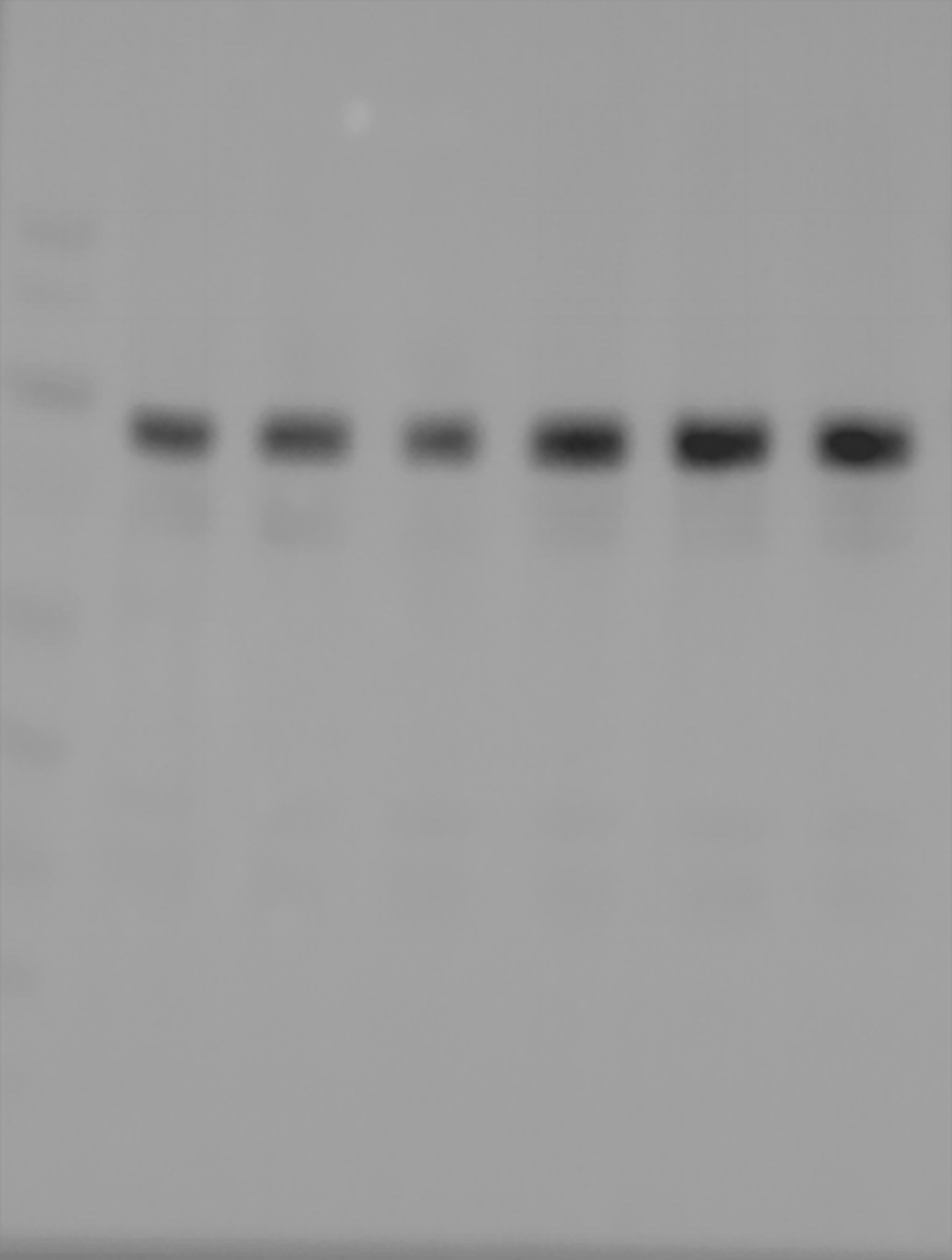

Supplement: Supplementary file 9 — Source data Fig. 6 [file 44321_2024_146_MOESM9_ESM.zip › Source data Fig. 6 (MOESM9)/Fig. 6E/3xTg-AD-SYN1-insolution.tif]

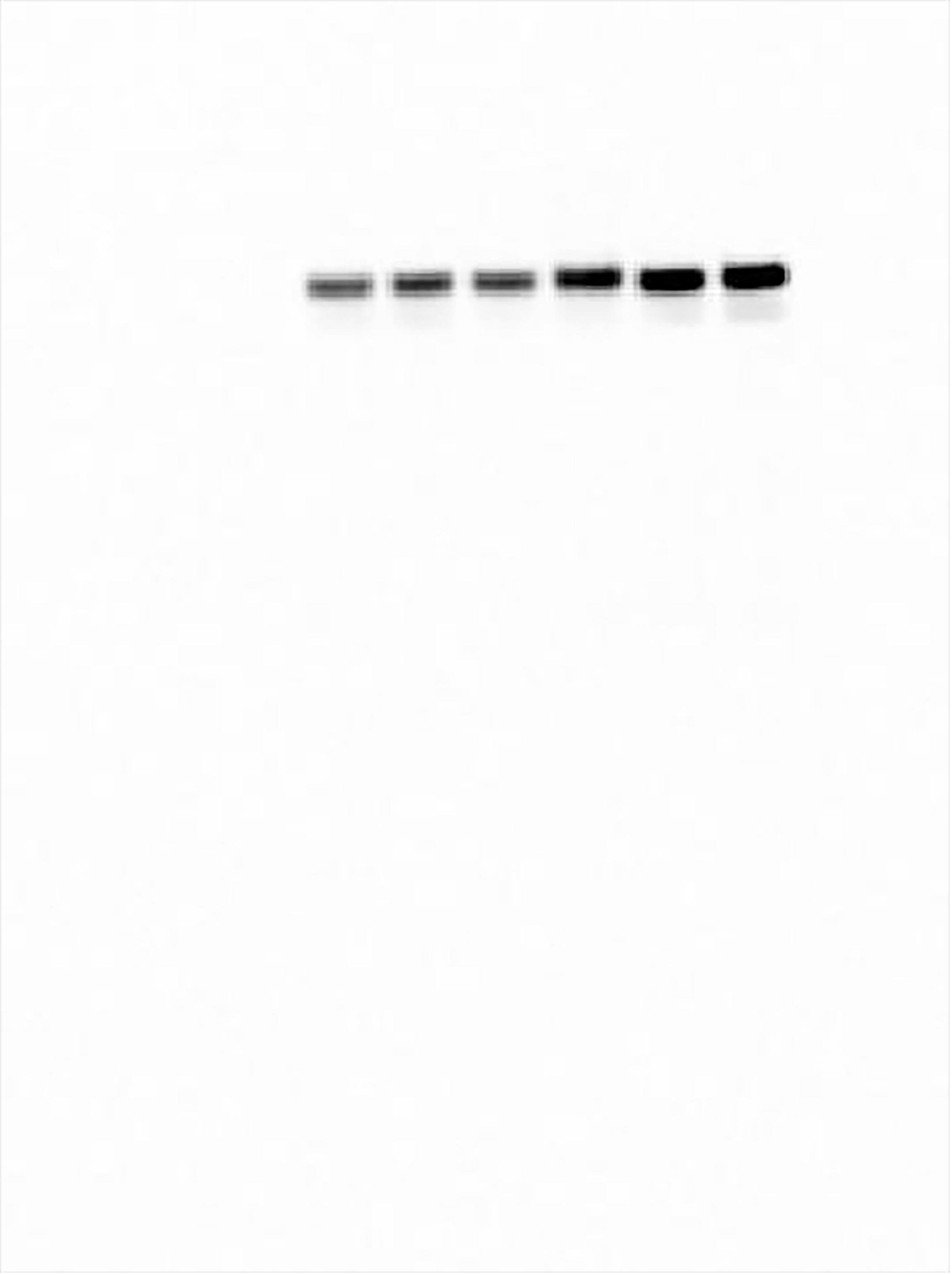

Supplement: Supplementary file 9 — Source data Fig. 6 [file 44321_2024_146_MOESM9_ESM.zip › Source data Fig. 6 (MOESM9)/Fig. 6E/3xTg-AD-SYN1-solution.tif]

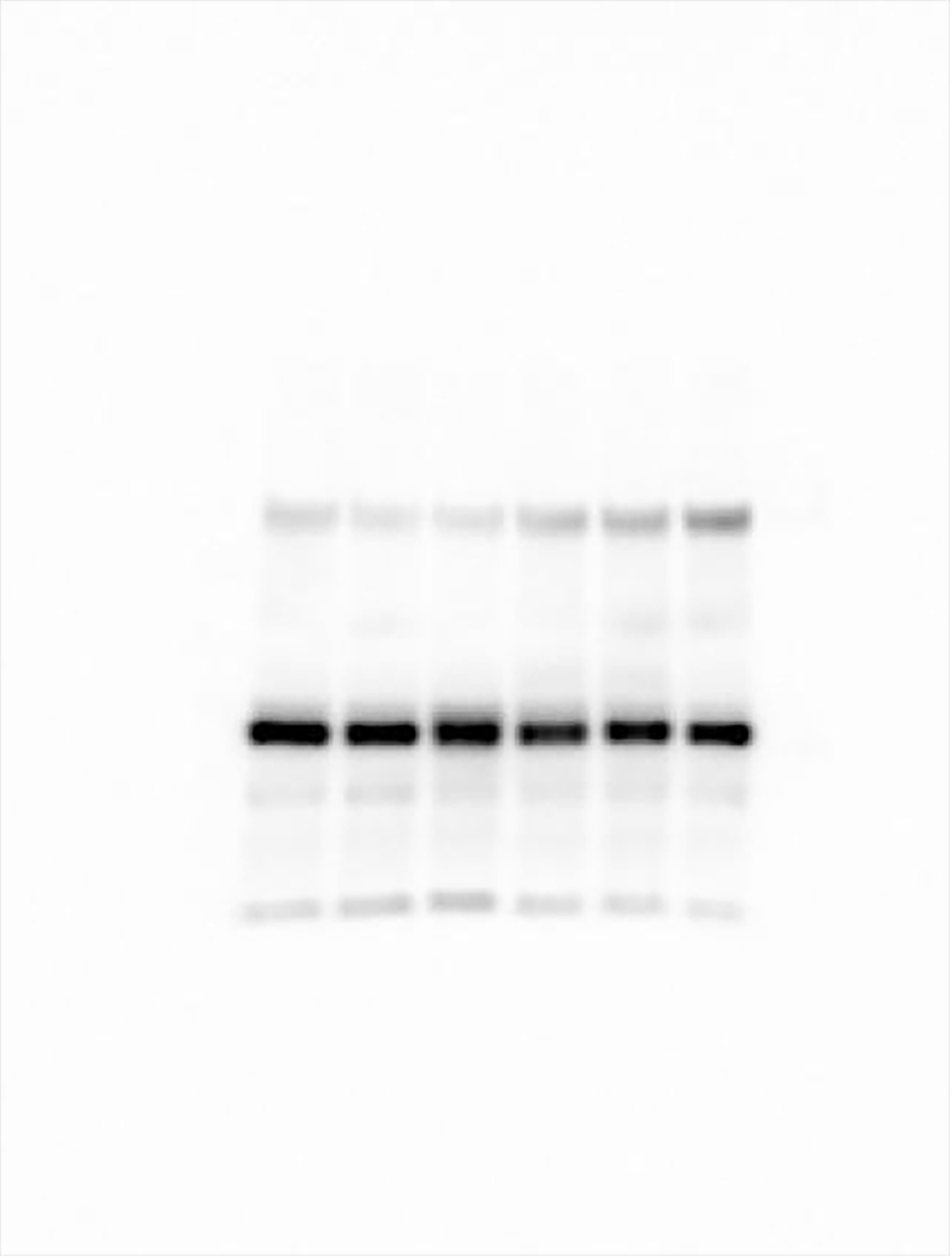

Supplement: Supplementary file 9 — Source data Fig. 6 [file 44321_2024_146_MOESM9_ESM.zip › Source data Fig. 6 (MOESM9)/Fig. 6E/5xFAD-PSD95-GAPDH-insolution.tif]

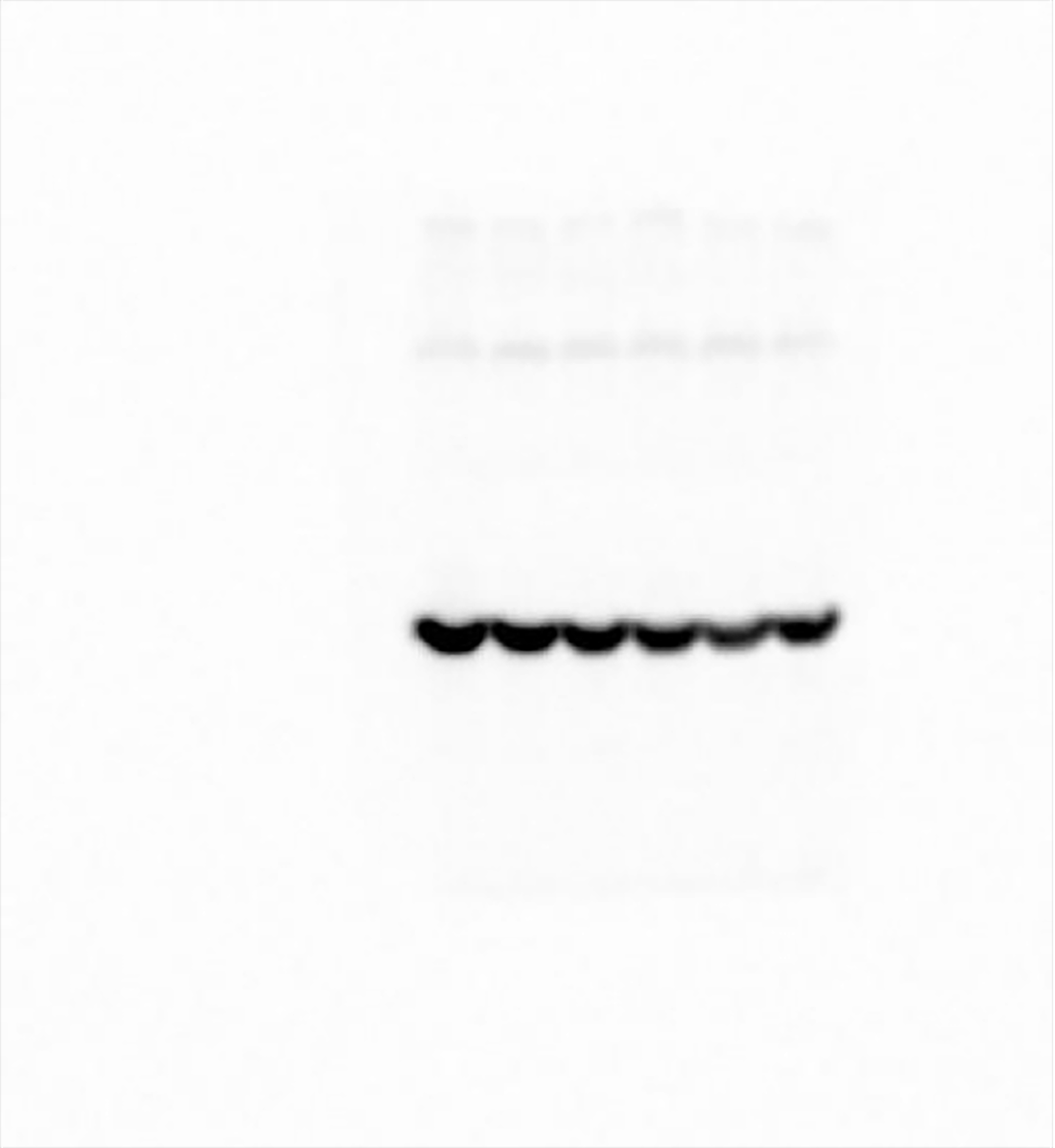

Supplement: Supplementary file 9 — Source data Fig. 6 [file 44321_2024_146_MOESM9_ESM.zip › Source data Fig. 6 (MOESM9)/Fig. 6E/5xFAD-PSD95-GAPDH-solution.tif]

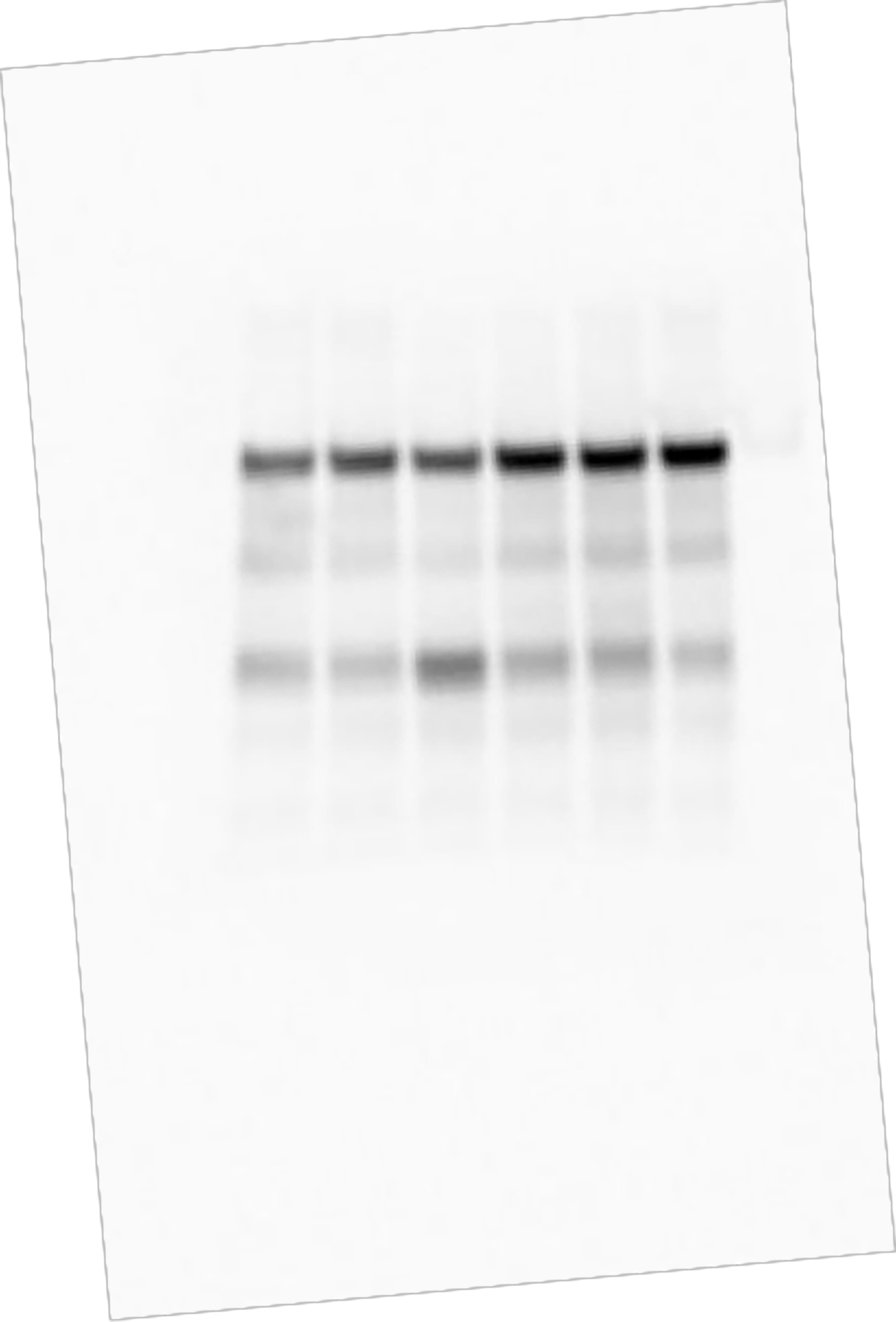

Supplement: Supplementary file 9 — Source data Fig. 6 [file 44321_2024_146_MOESM9_ESM.zip › Source data Fig. 6 (MOESM9)/Fig. 6E/5xFAD-PSD95-insolution.tif]

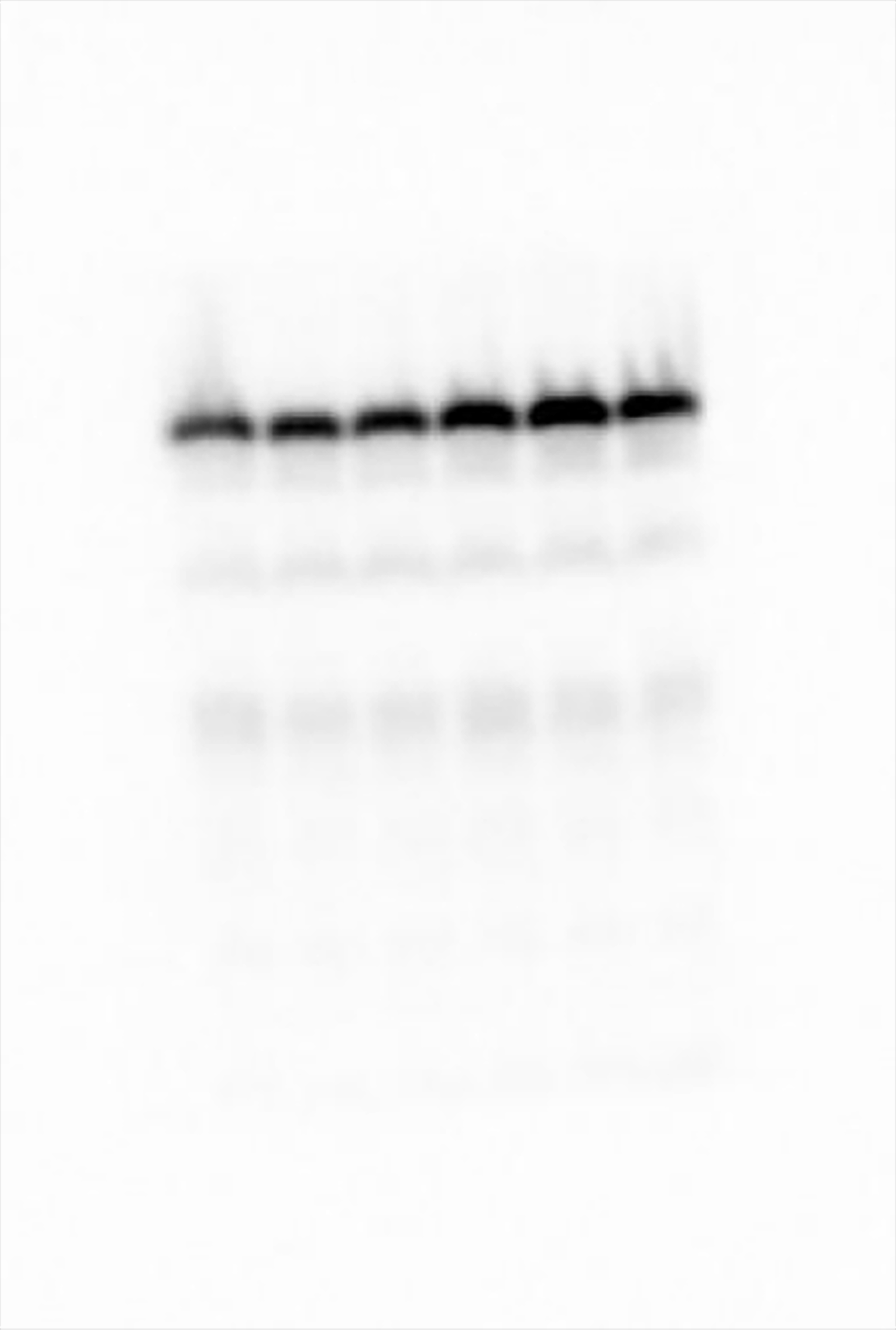

Supplement: Supplementary file 9 — Source data Fig. 6 [file 44321_2024_146_MOESM9_ESM.zip › Source data Fig. 6 (MOESM9)/Fig. 6E/5xFAD-PSD95-solution.tif]
