# Supplementary material for: Targeting USP11 regulation by a novel lithium-organic coordination compound improves neuropathologies and cognitive functions in Alzheimer transgenic mice
Source: EMBO Mol Med. 2024 Oct 11;16(11):2856–81. doi: 10.1038/s44321-024-00146-7 (PMC11555261; doi:10.1038/s44321-024-00146-7)
Supplement: Supplementary file 4 — Source data Fig. 2 [file 44321_2024_146_MOESM4_ESM.zip › Fig. 2/Fig2.pdf]

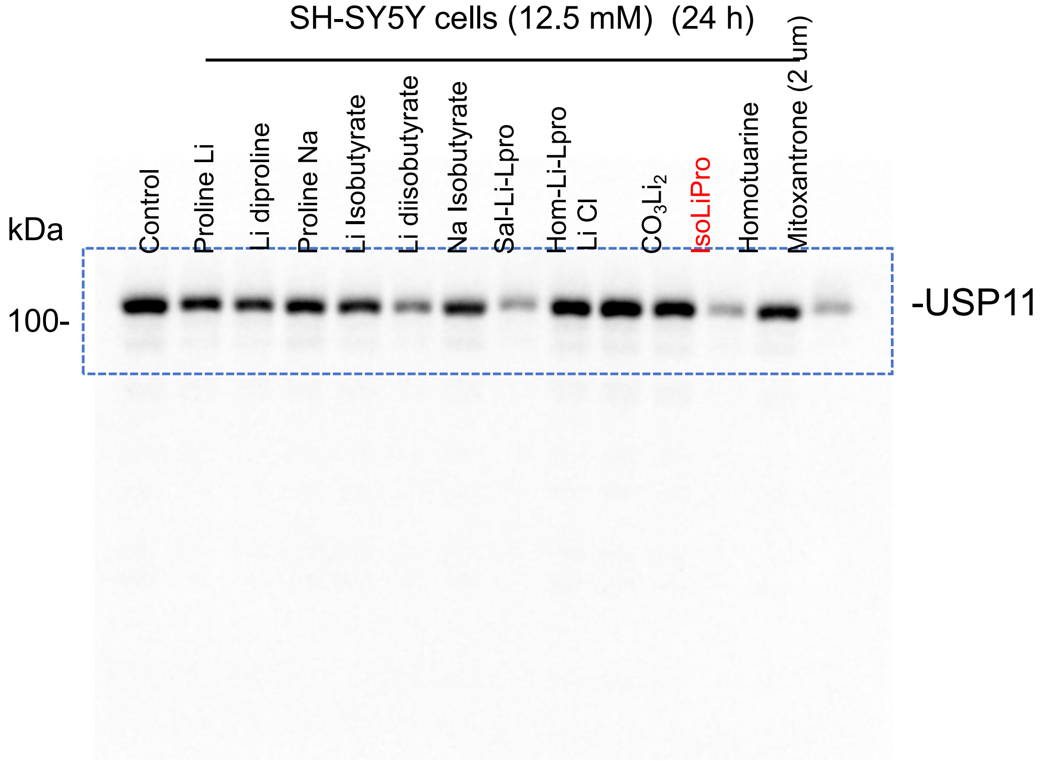


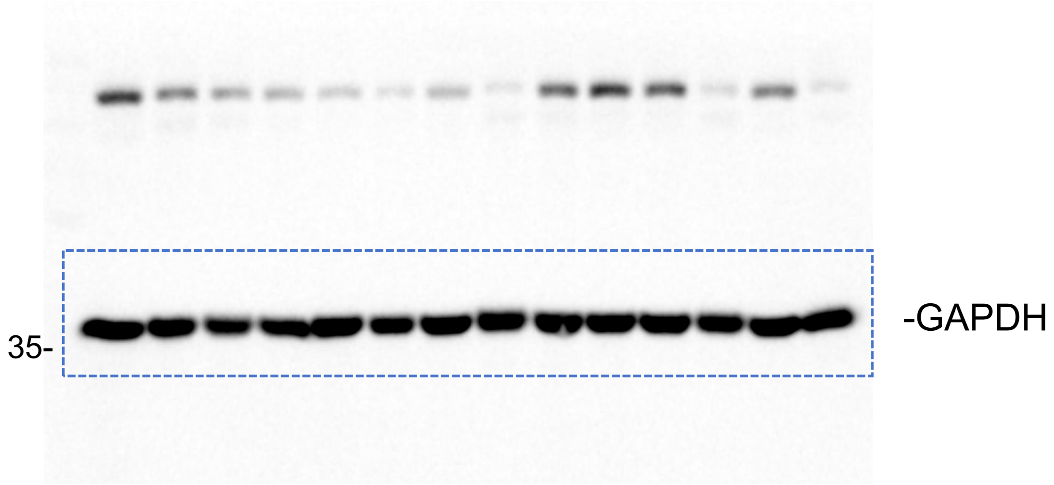


**Full unedited gel for Fig.** 2 **A**

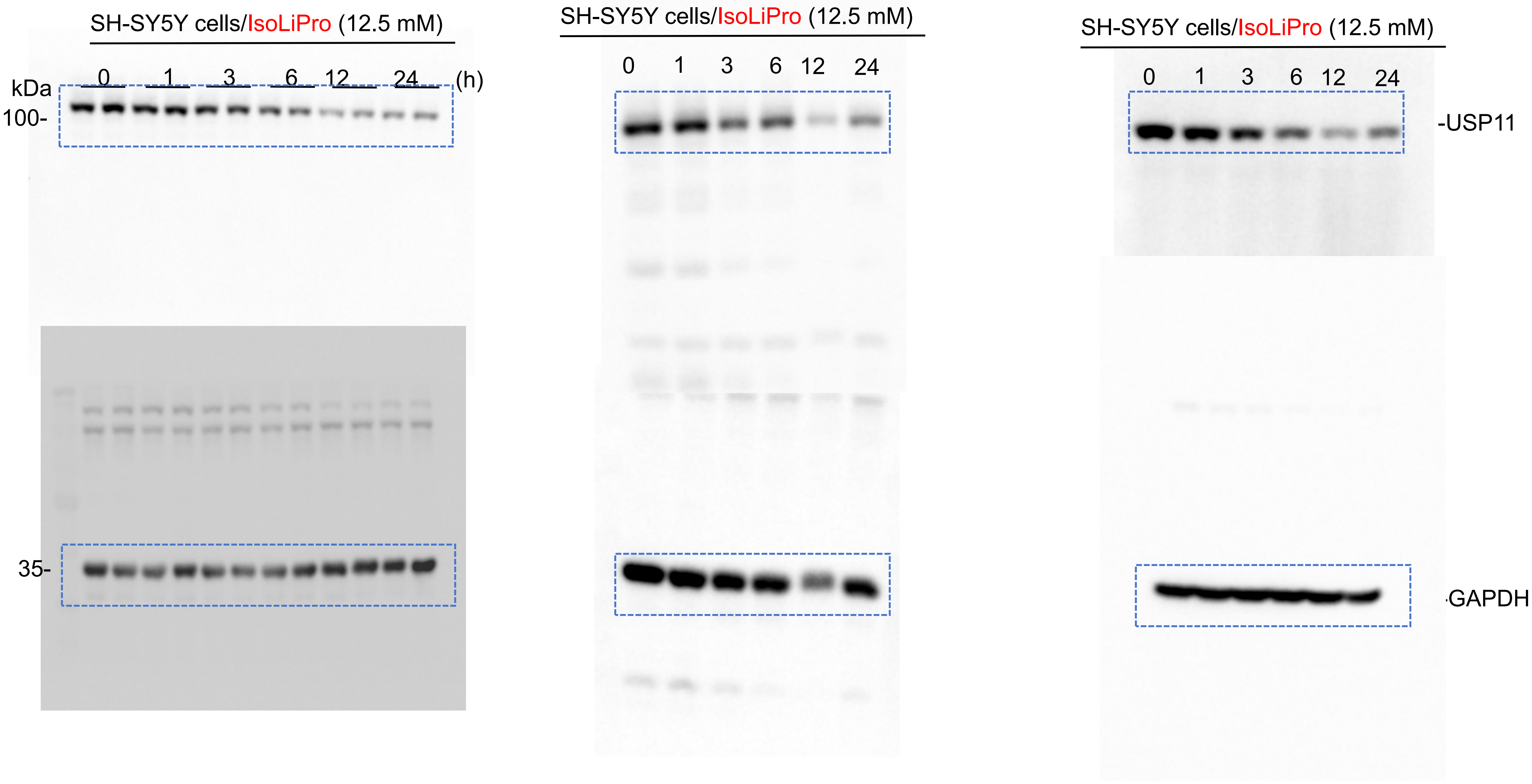


**Full unedited gel for Fig. 2 B**

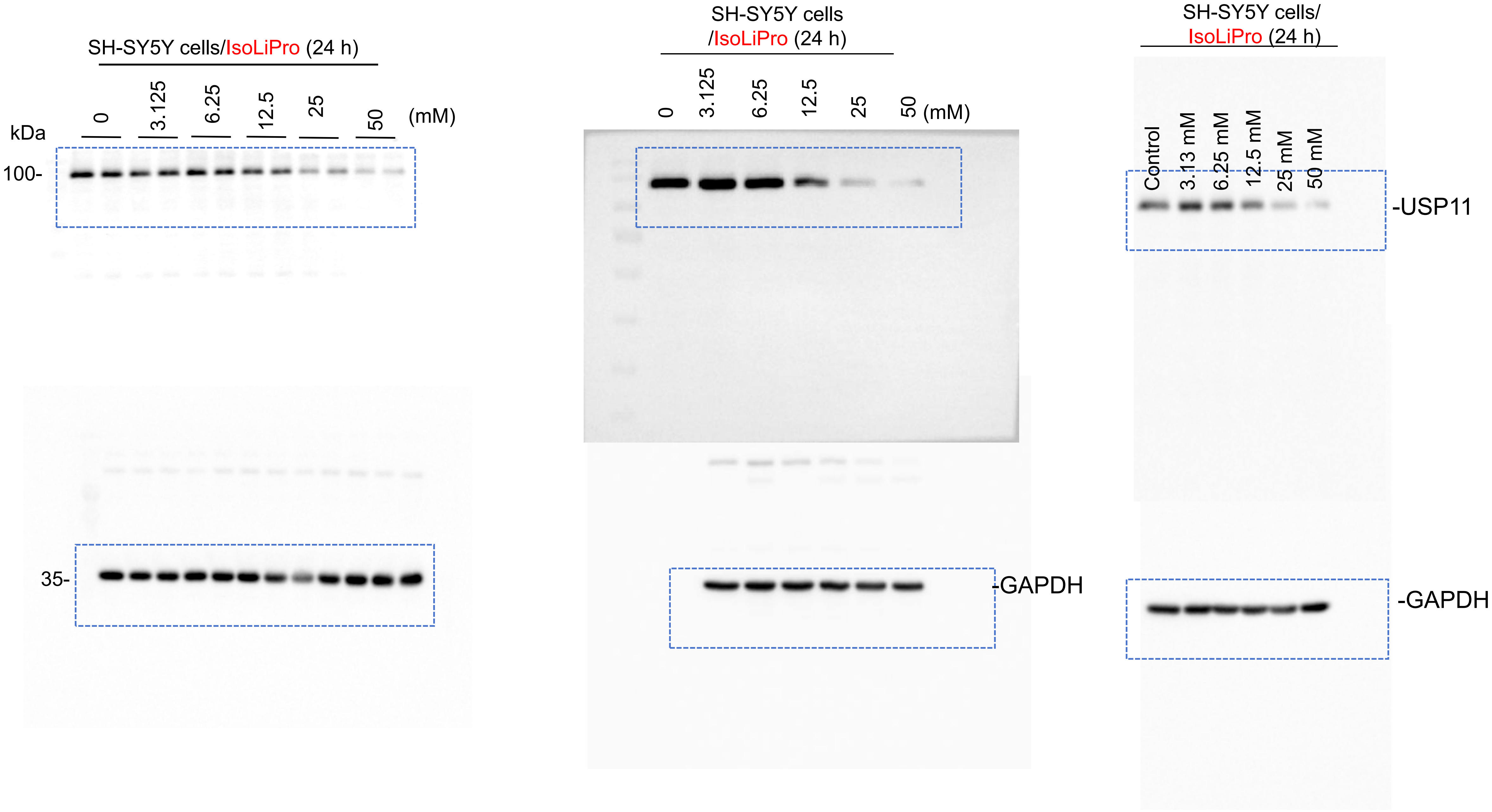


**Full unedited gel for Fig. 2 B**

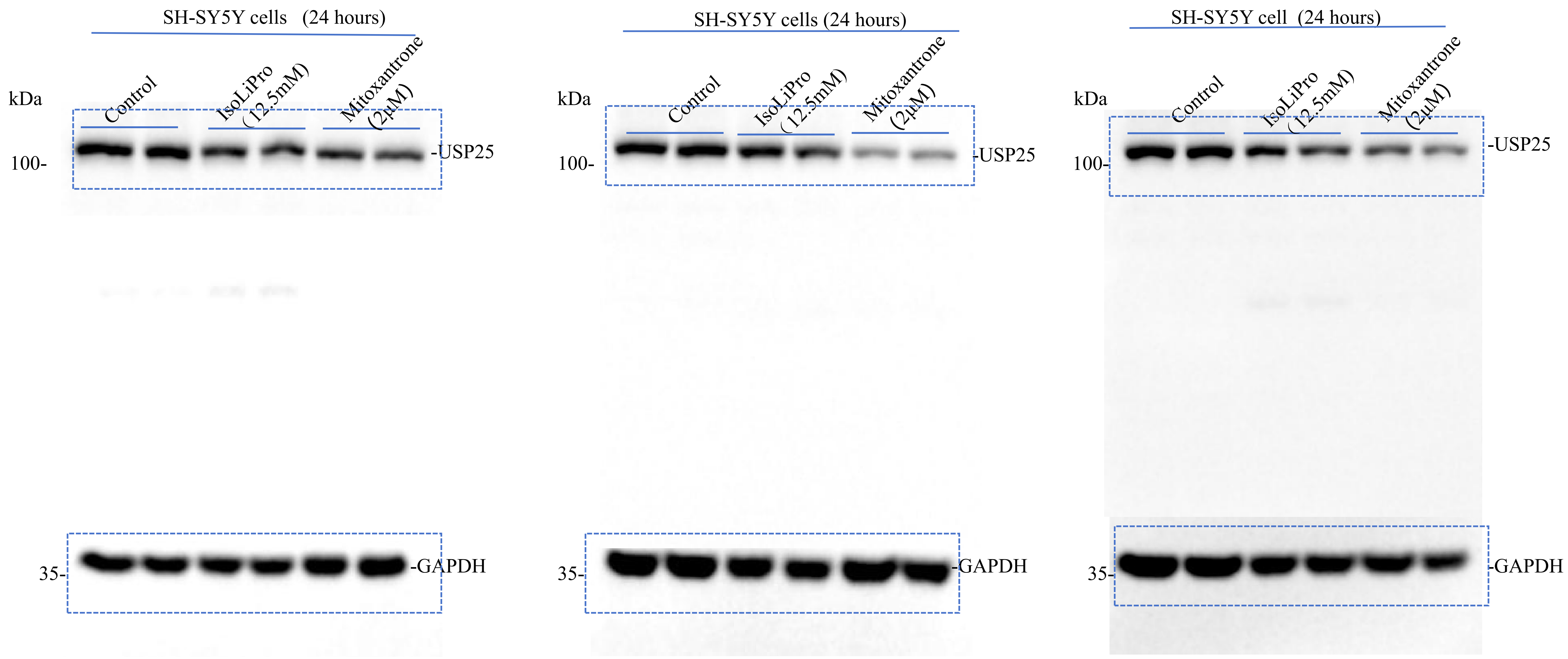


**Full unedited gel for Fig. 2 D**

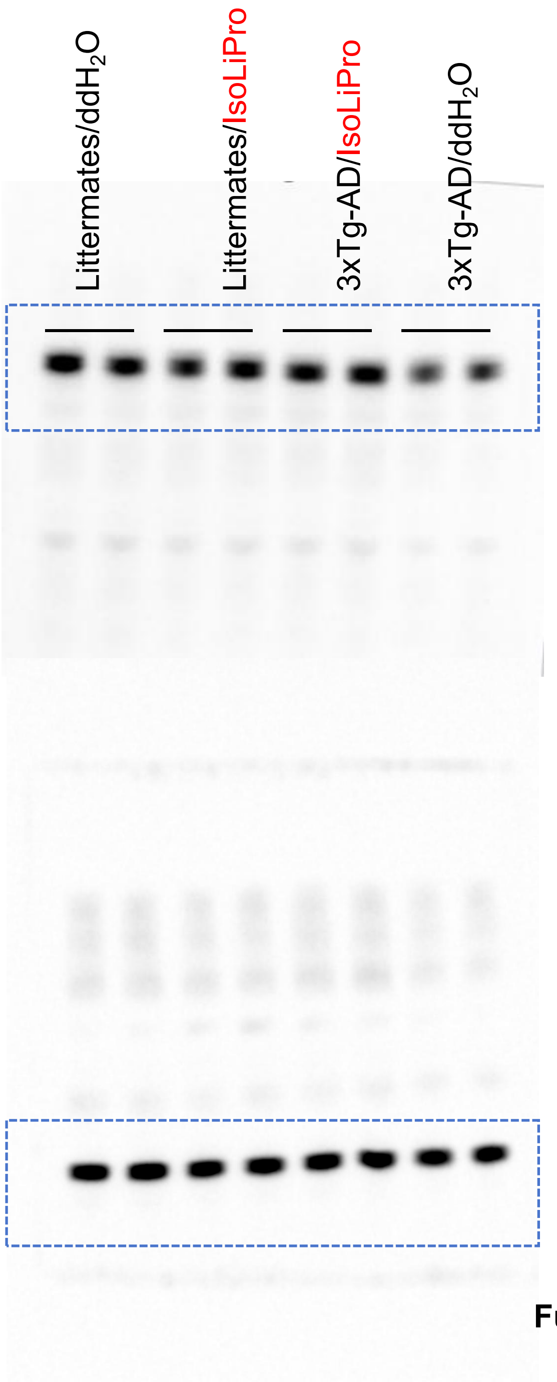


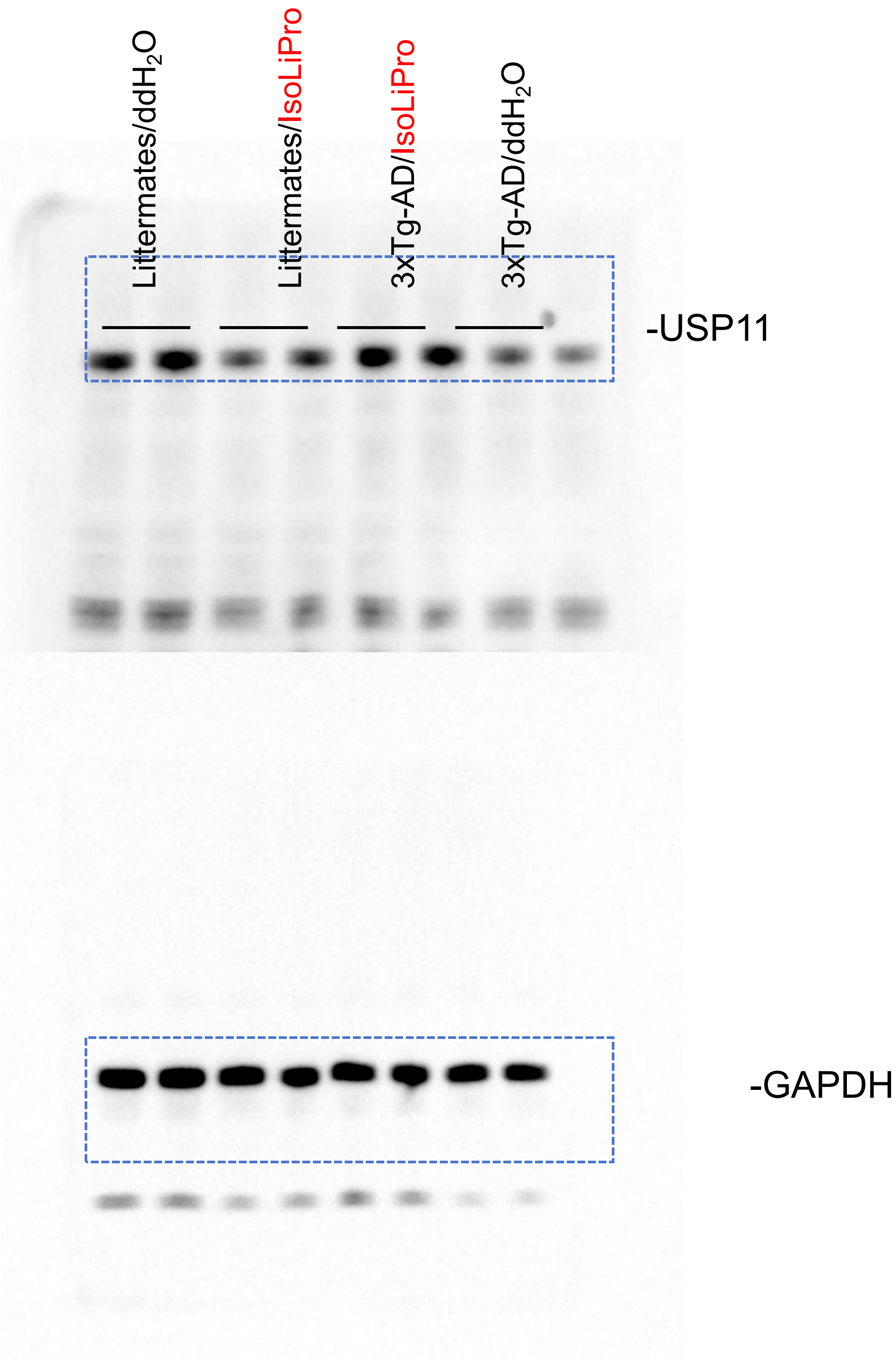


**Full unedited gel for Fig. 2F**

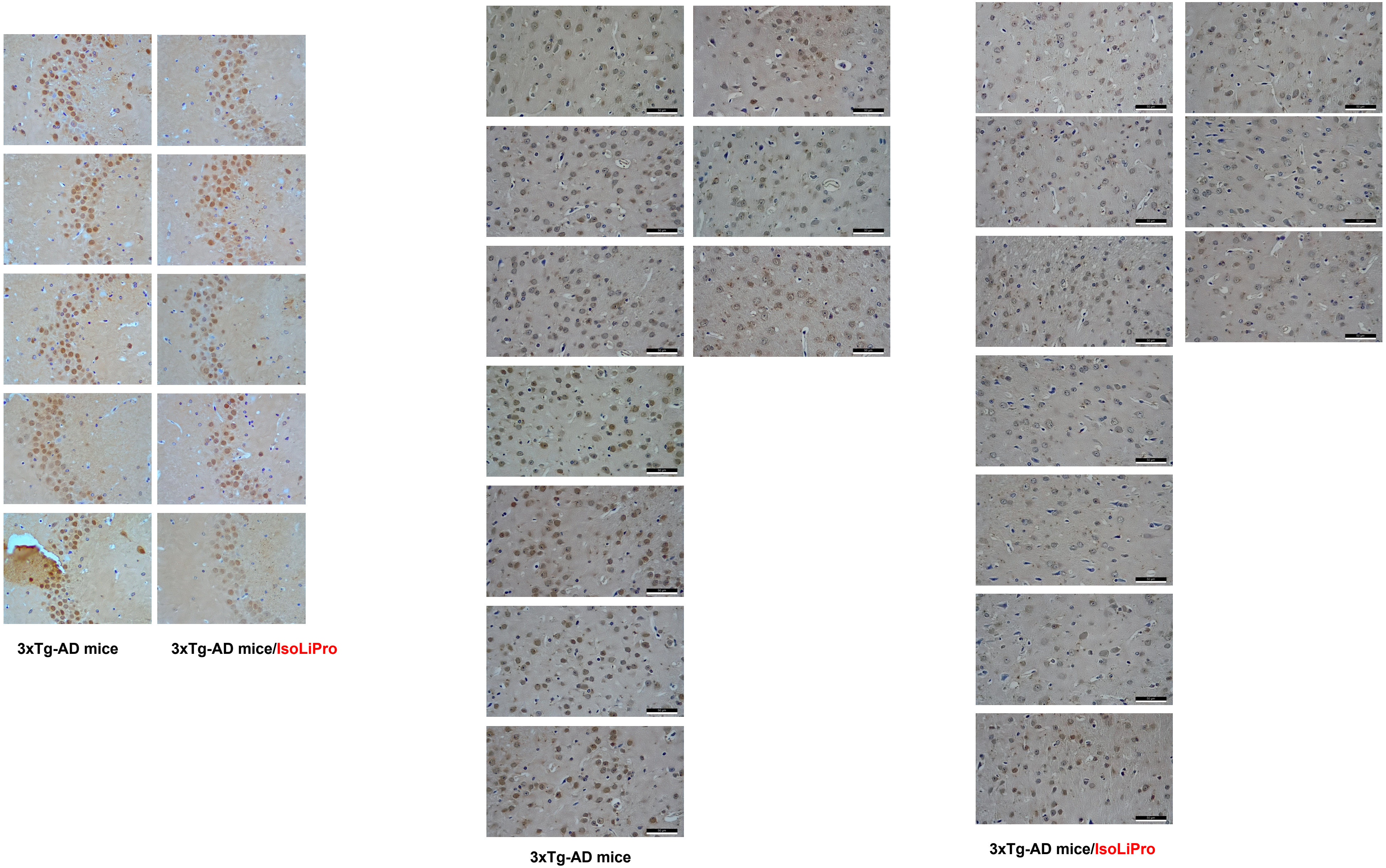
3xTg-AD mice
3xTg-AD mice/IsoLiPro
3xTg-AD mice
3xTg-AD mice/IsoLiPro

**Fig.** 2 **G**
