# Supplementary material for: Targeting USP11 regulation by a novel lithium-organic coordination compound improves neuropathologies and cognitive functions in Alzheimer transgenic mice
Source: EMBO Mol Med. 2024 Oct 11;16(11):2856–81. doi: 10.1038/s44321-024-00146-7 (PMC11555261; doi:10.1038/s44321-024-00146-7)
Supplement: Supplementary file 5 — Source data Fig. 3 [file 44321_2024_146_MOESM5_ESM.zip › Fig. 3/Fig. 3.pdf]

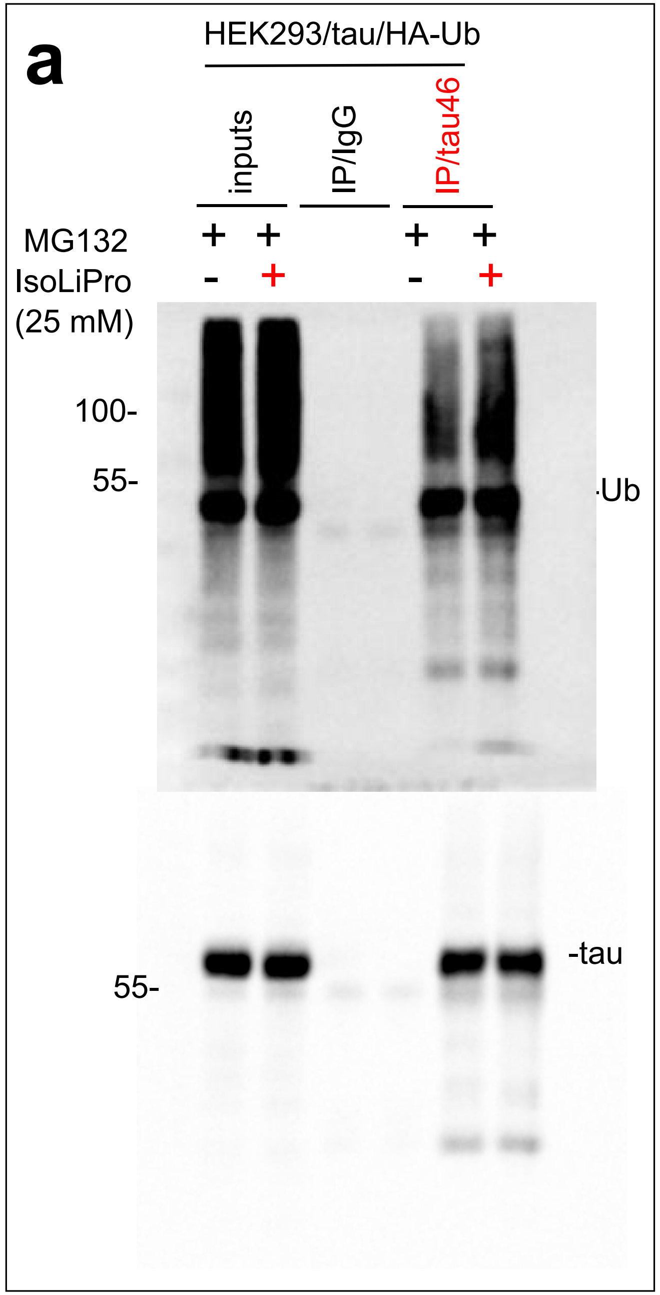

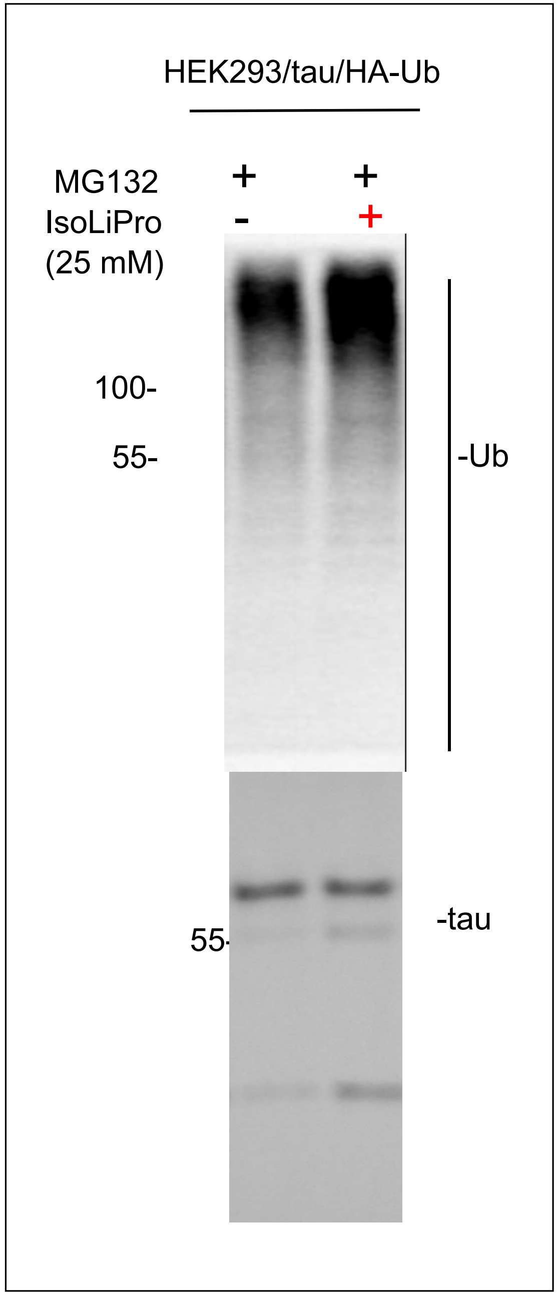

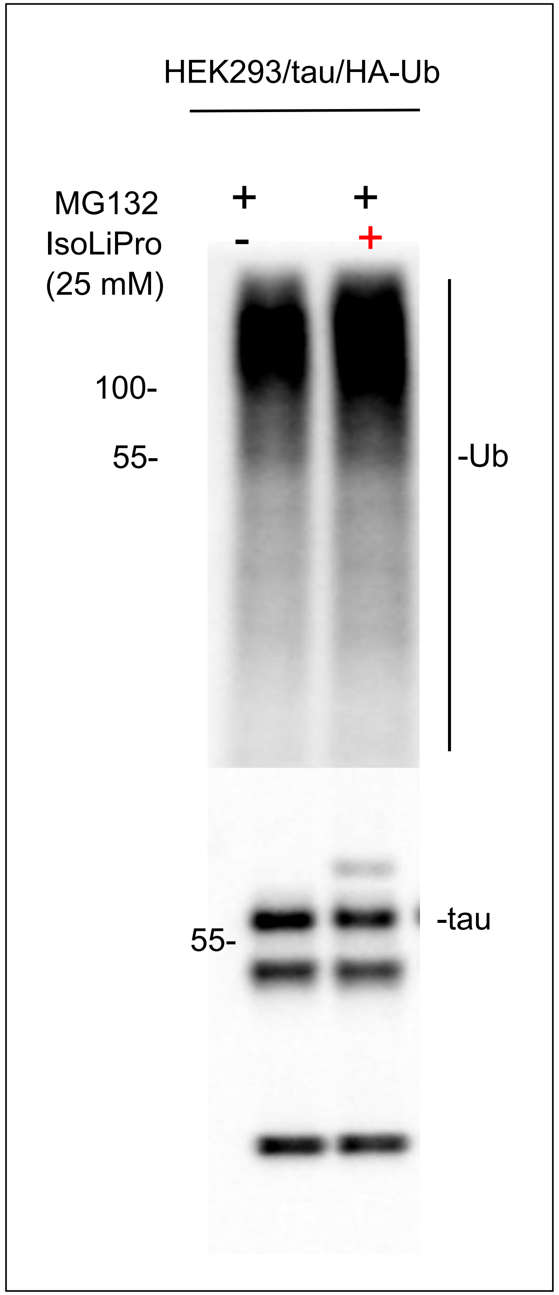

Full unedited gel for Fig. 3A

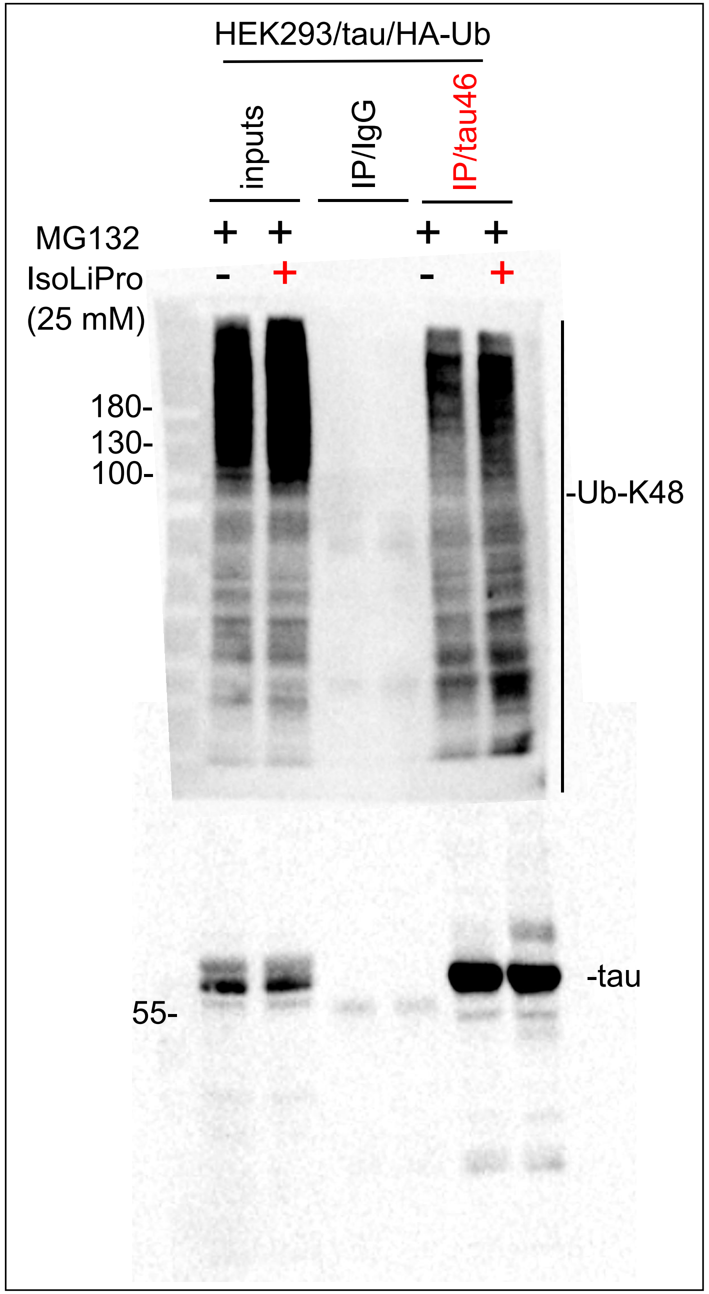

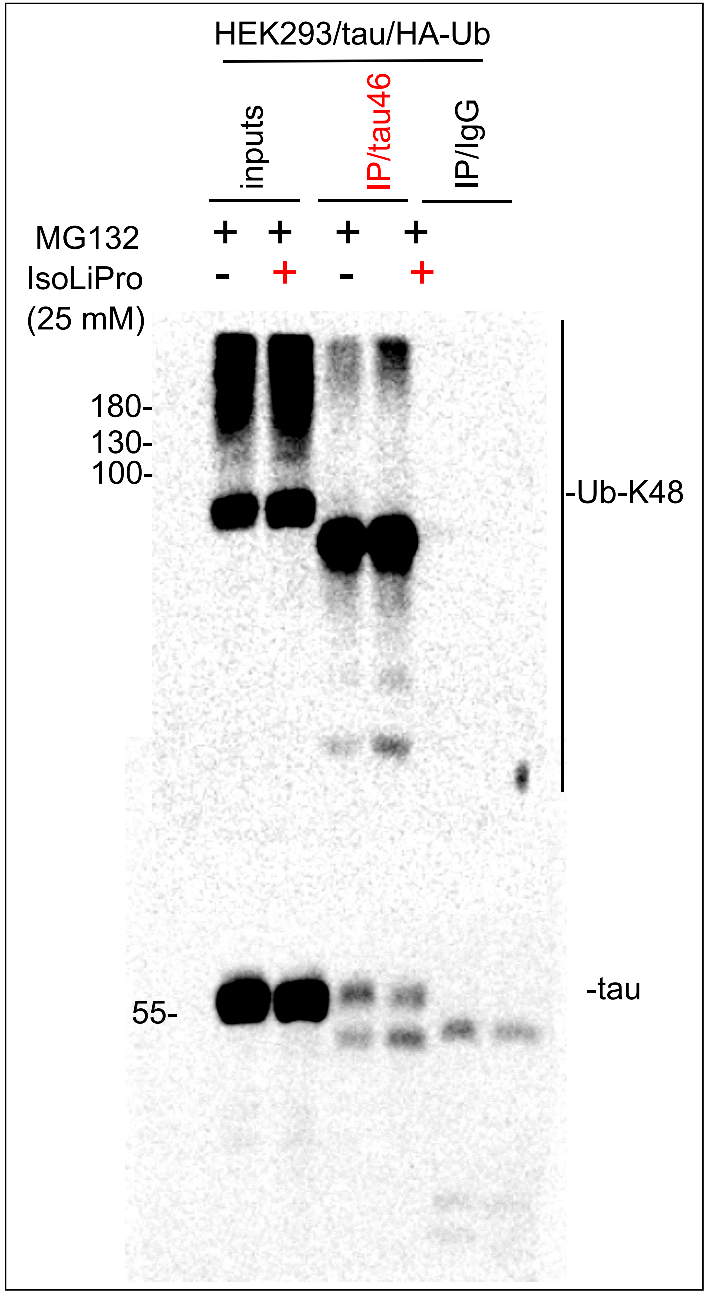

Full unedited gel for Figures 3B

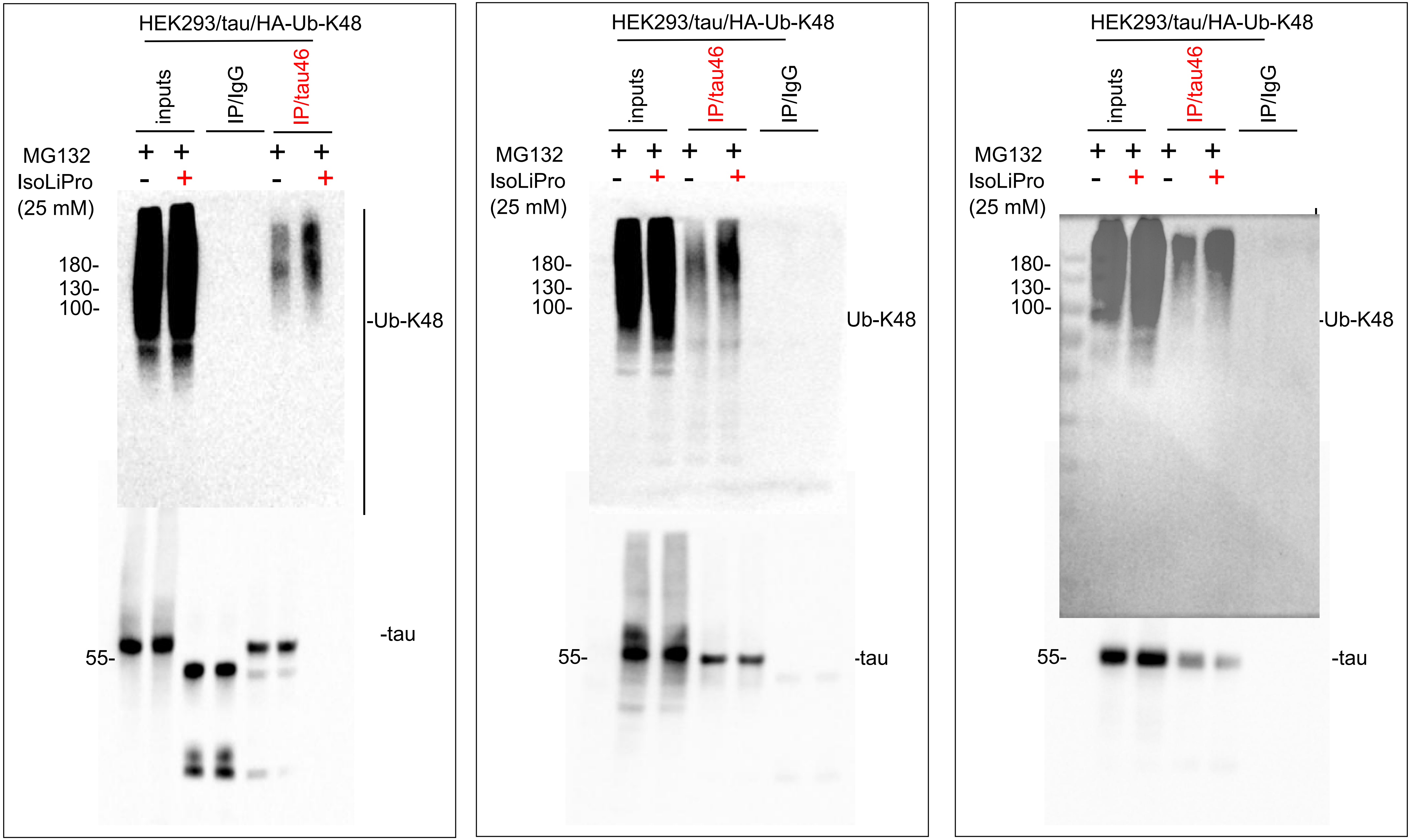

Full unedited gel for Fig. 3B

Full unedited gel for Figures 5d

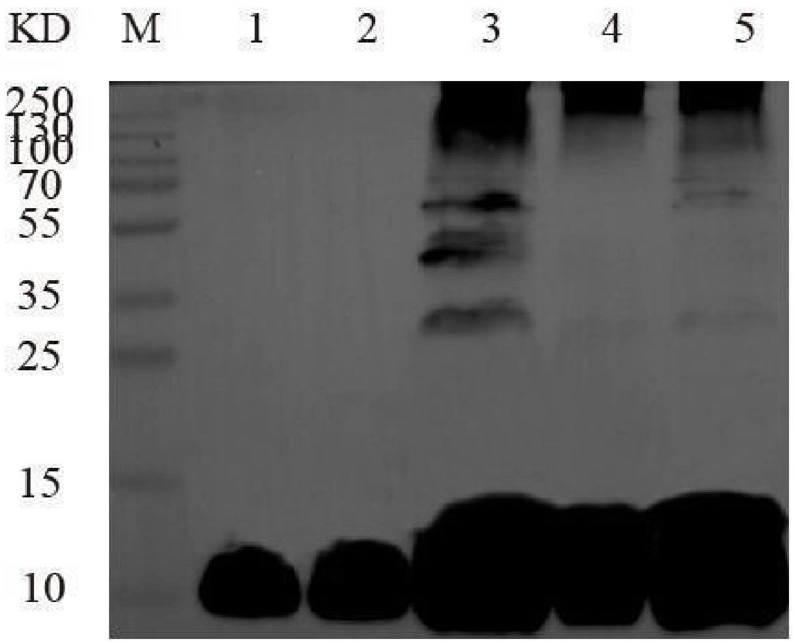

Full unedited gel for Fig. 3D
