# Supplementary material for: Targeting USP11 regulation by a novel lithium-organic coordination compound improves neuropathologies and cognitive functions in Alzheimer transgenic mice
Source: EMBO Mol Med. 2024 Oct 11;16(11):2856–81. doi: 10.1038/s44321-024-00146-7 (PMC11555261; doi:10.1038/s44321-024-00146-7)
Supplement: Supplementary file 8 — Source data Fig. 5 [file 44321_2024_146_MOESM8_ESM.zip › Fig. 5/Fig. 5.pdf]

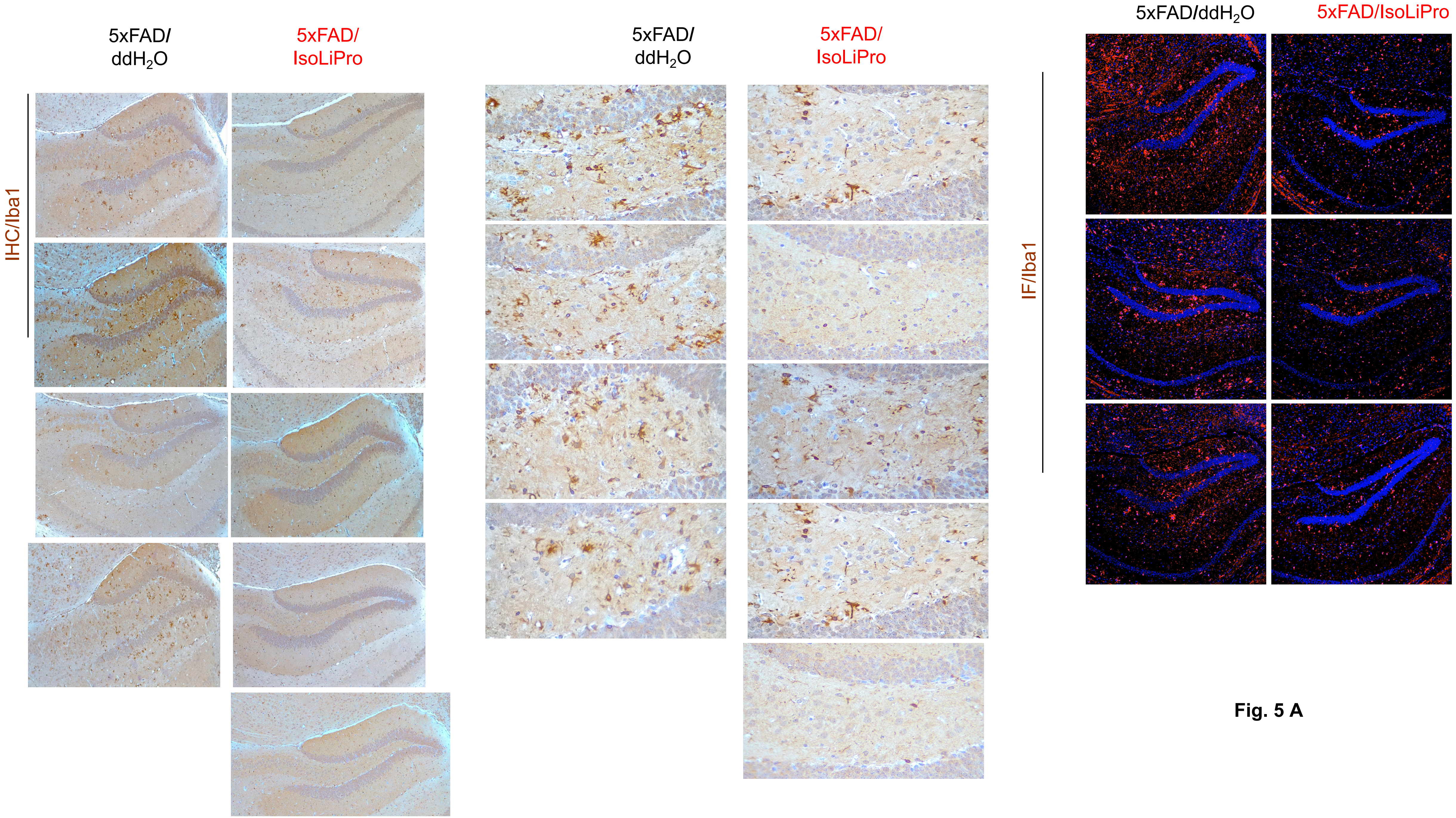
5xFAD/ ddH2O
5xFAD/ IsoLiPro
IHC/Iba1
5xFAD/ ddH2O
5xFAD/ IsoLiPro
5xFAD/ddH2O
5xFAD/IsoLiPro
IF/Iba1

Fig. 5 A

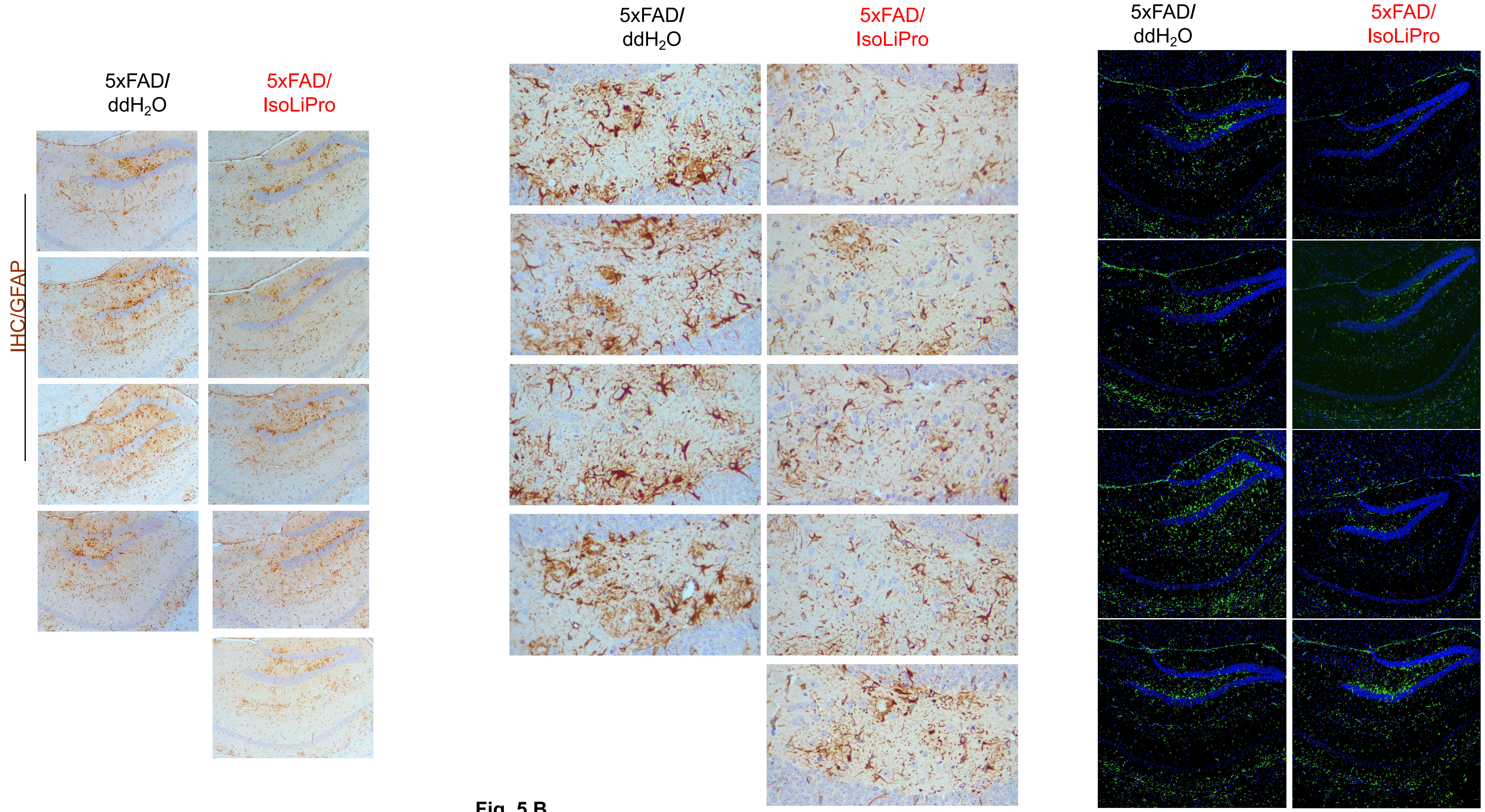
5xFAD/ ddH$_2$O
5xFAD/ IsoLiPro
IHC/GFAP
5xFAD/ ddH$_2$O
5xFAD/ IsoLiPro
5xFAD/ ddH$_2$O
5xFAD/ IsoLiPro

Fig. 5 B

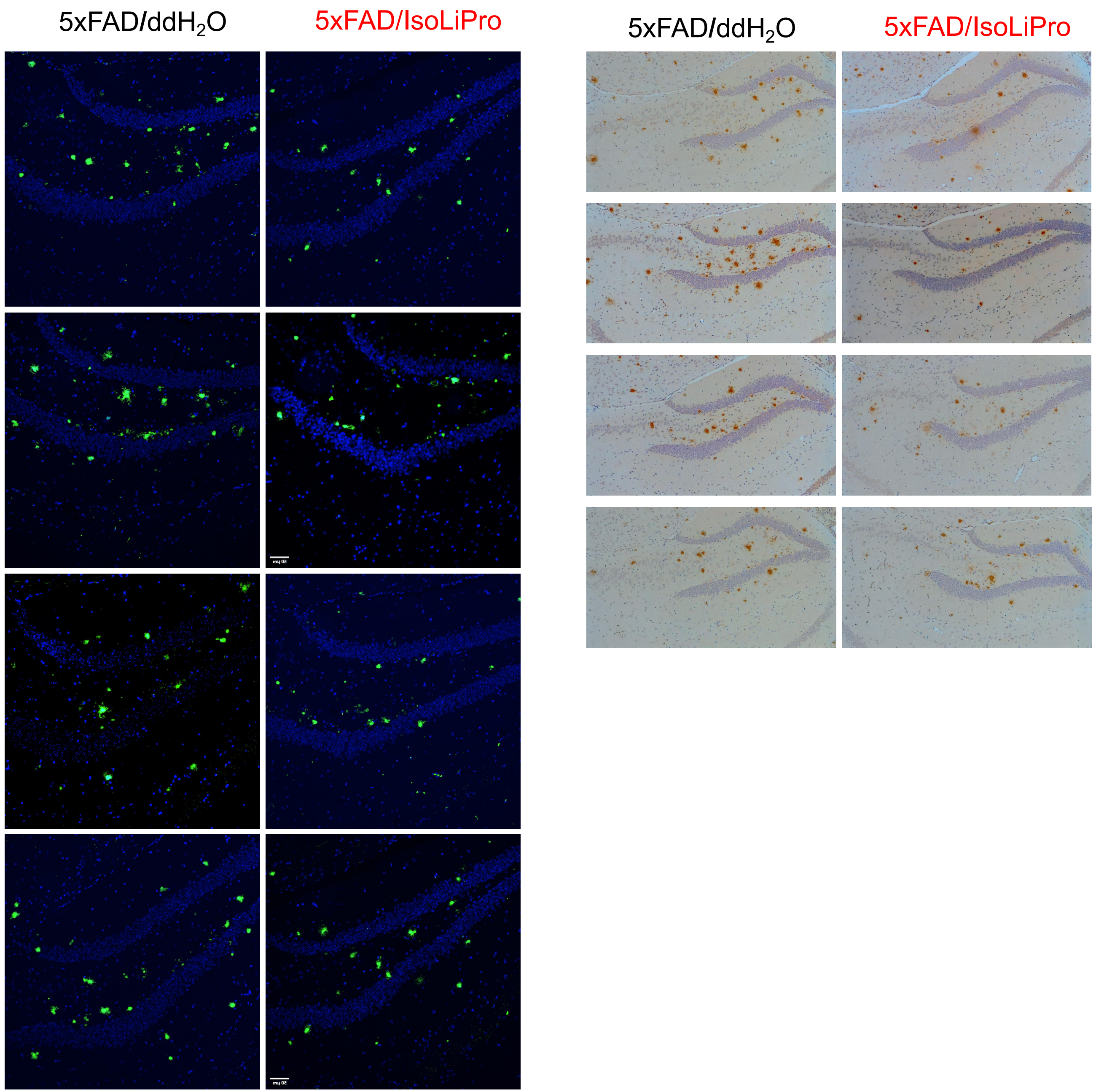

Fig. 5 E
