# Supplementary material for: Targeting USP11 regulation by a novel lithium-organic coordination compound improves neuropathologies and cognitive functions in Alzheimer transgenic mice
Source: EMBO Mol Med. 2024 Oct 11;16(11):2856–81. doi: 10.1038/s44321-024-00146-7 (PMC11555261; doi:10.1038/s44321-024-00146-7)
Supplement: Supplementary file 9 — Source data Fig. 6 [file 44321_2024_146_MOESM9_ESM.zip › Source data Fig. 6 (MOESM9)/Fig. 6.pdf]

Full unedited gel for Figures 8c

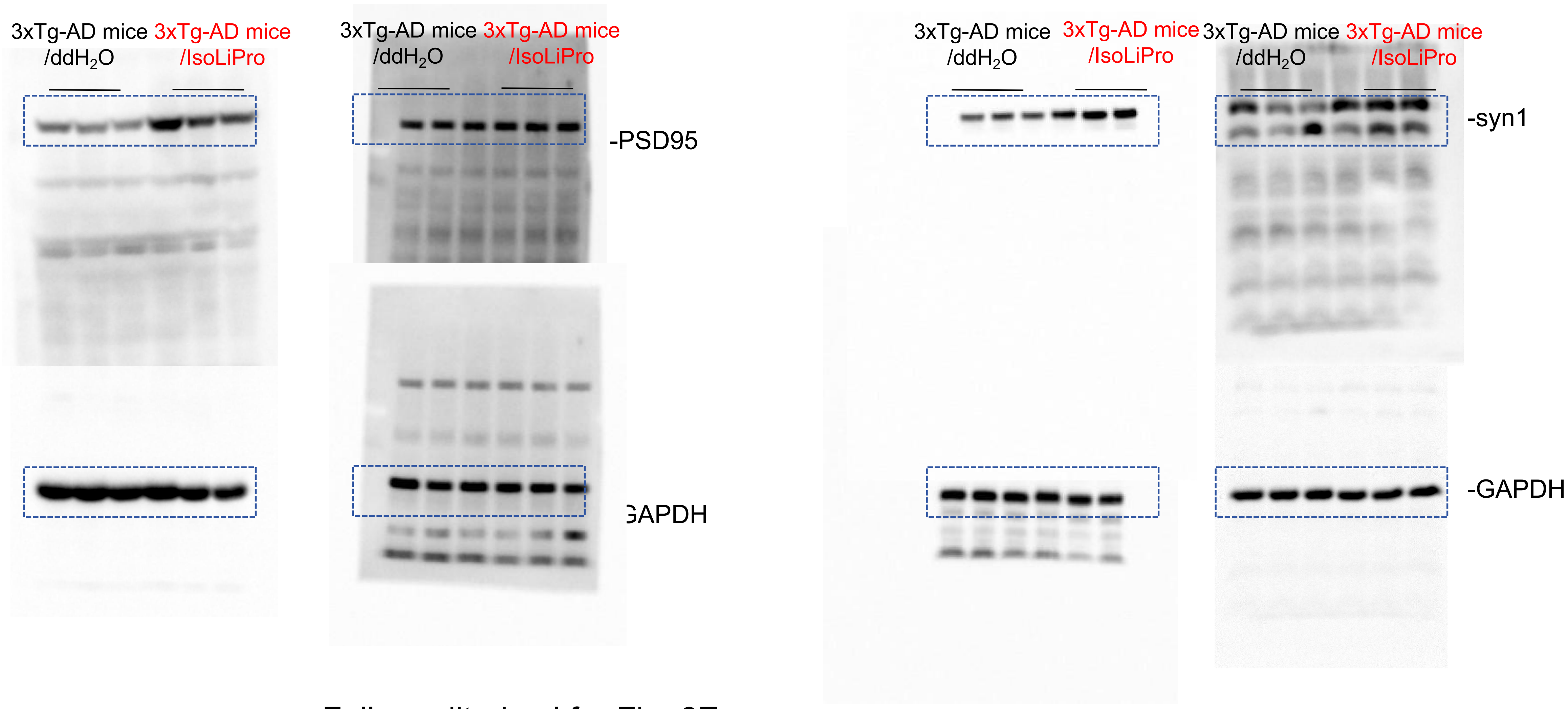

Full unedited gel for Fig. 6E

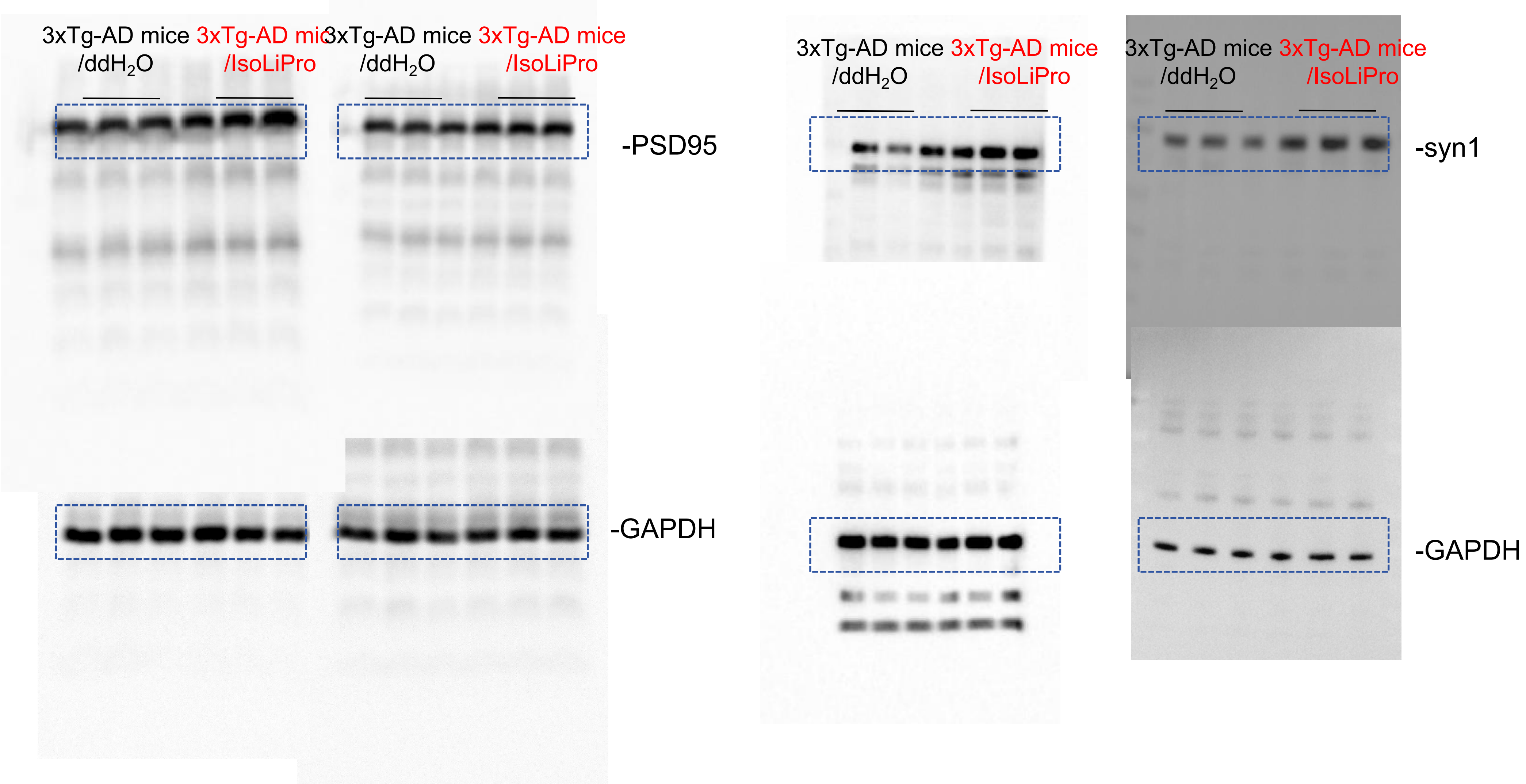

Full unedited gel for Fig. 6E

Full unedited gel for Figures 8c

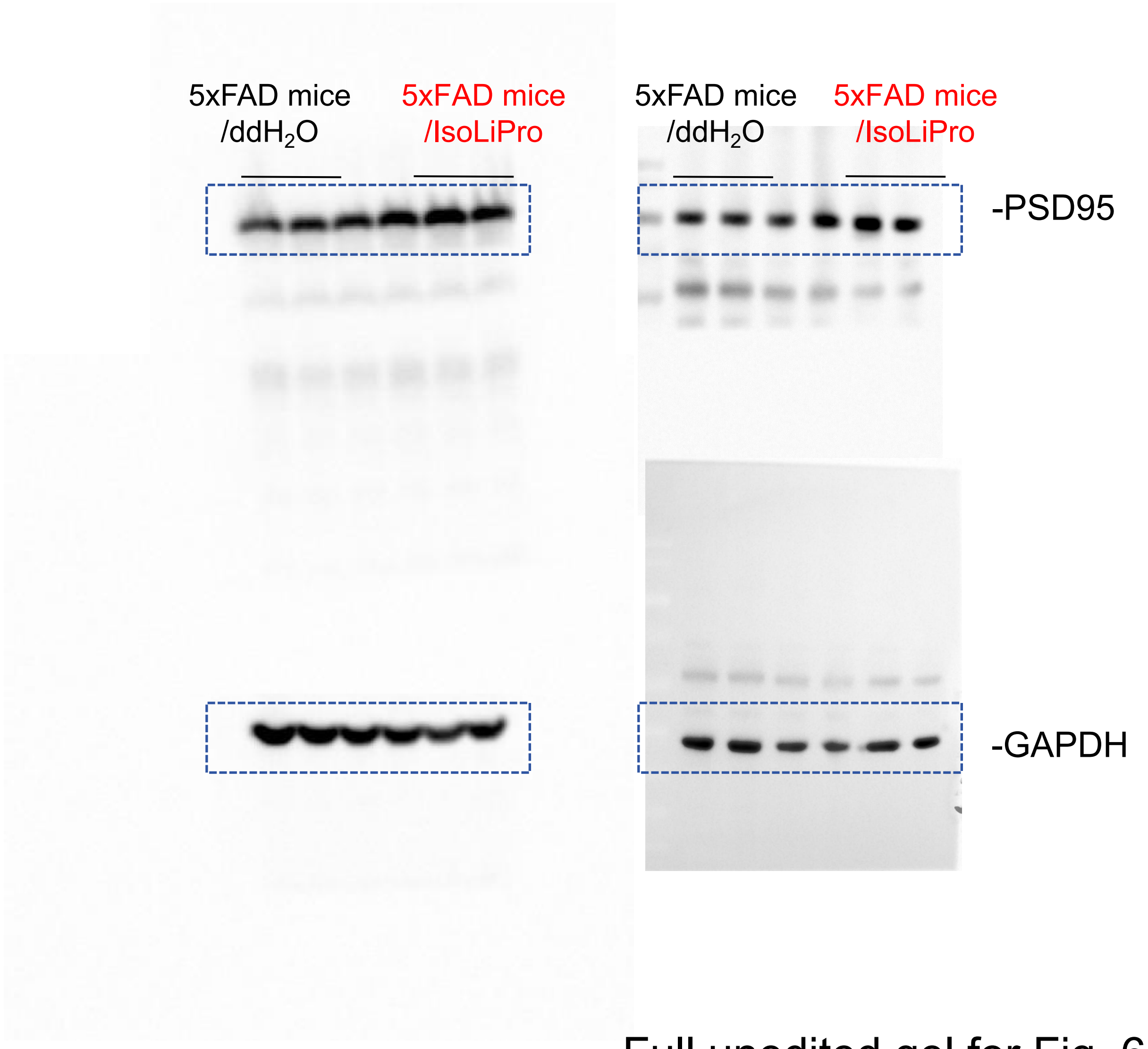

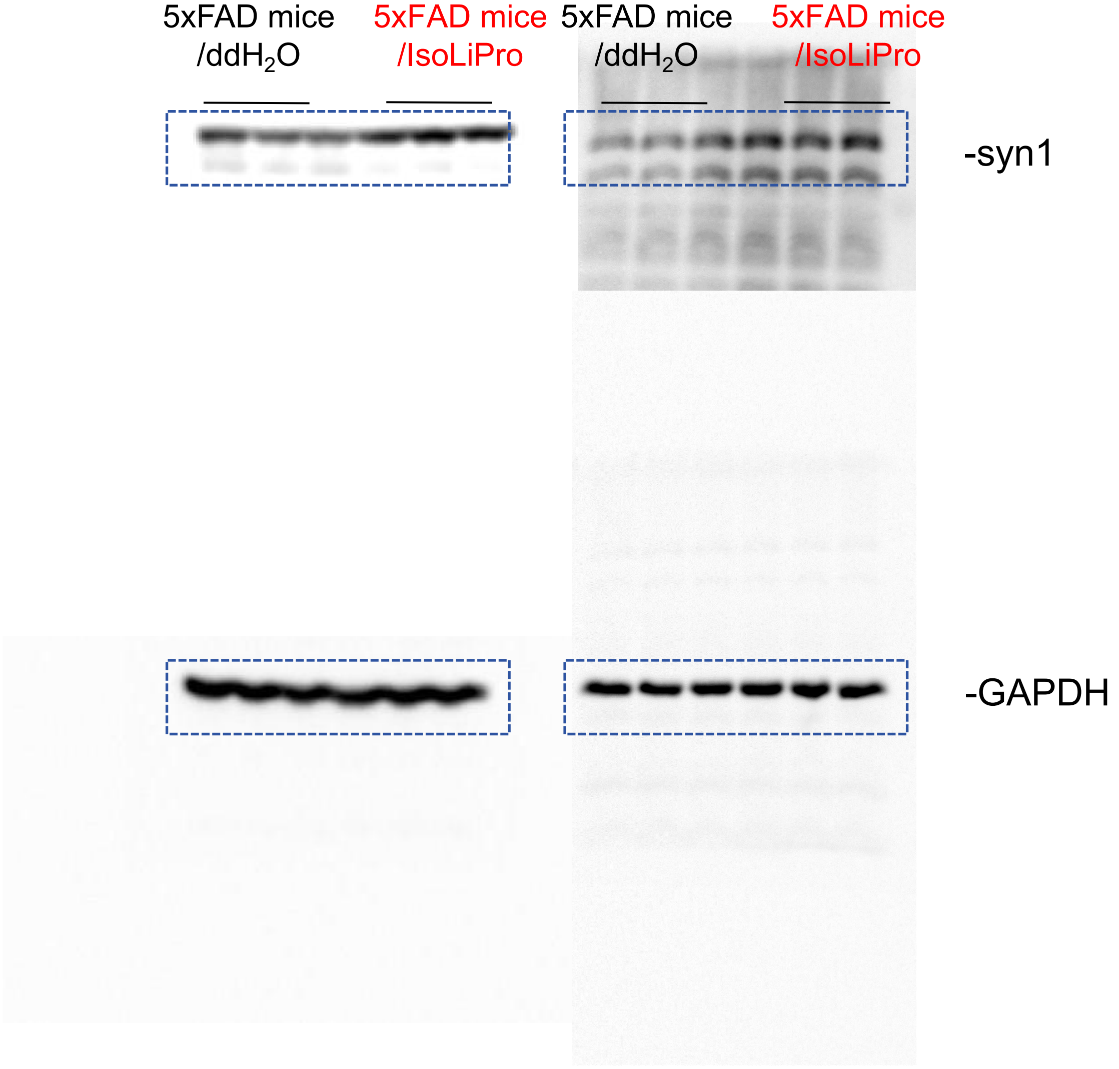

Full unedited gel for Fig. 6E

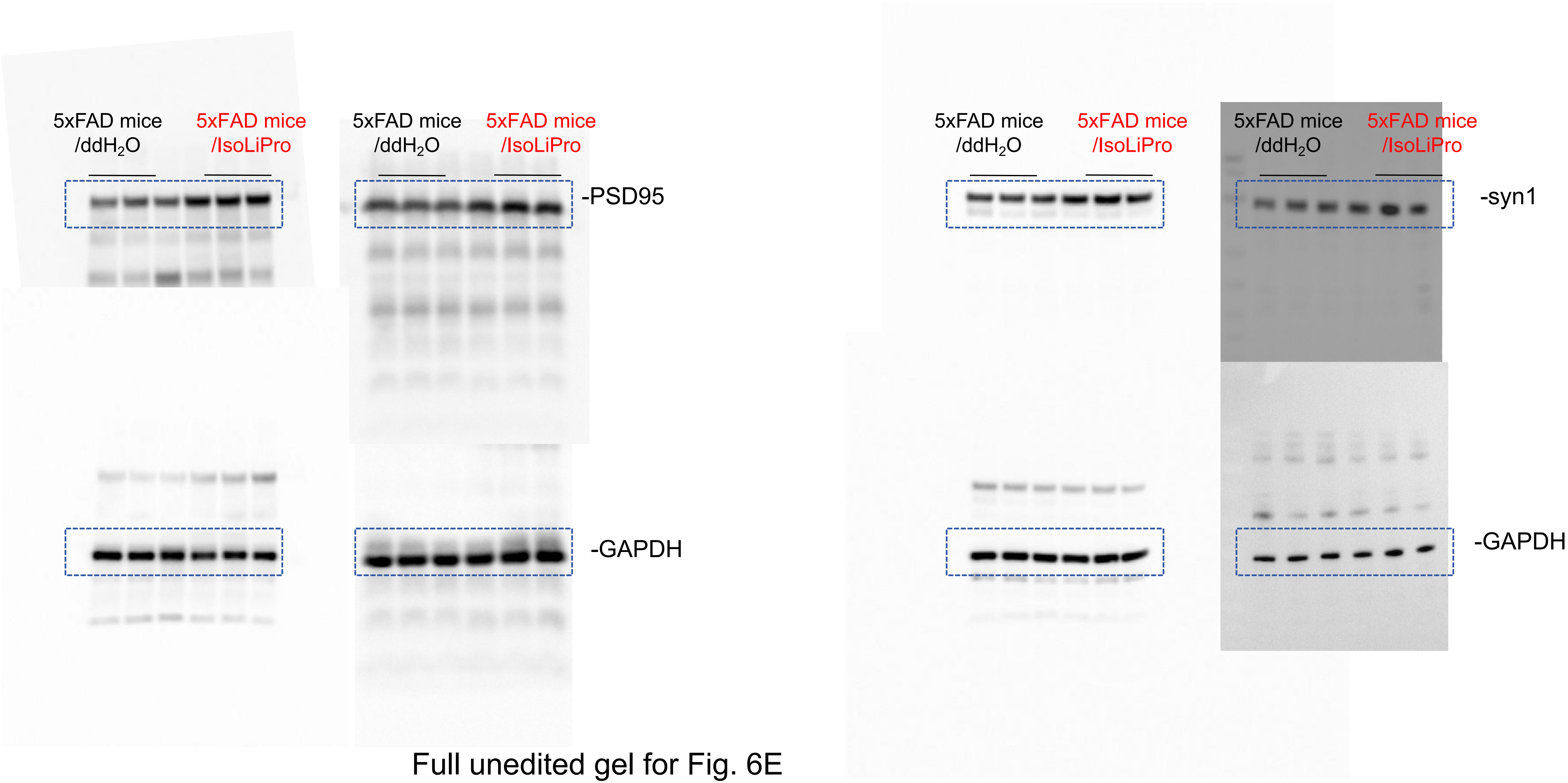

Full unedited gel for Fig. 6E

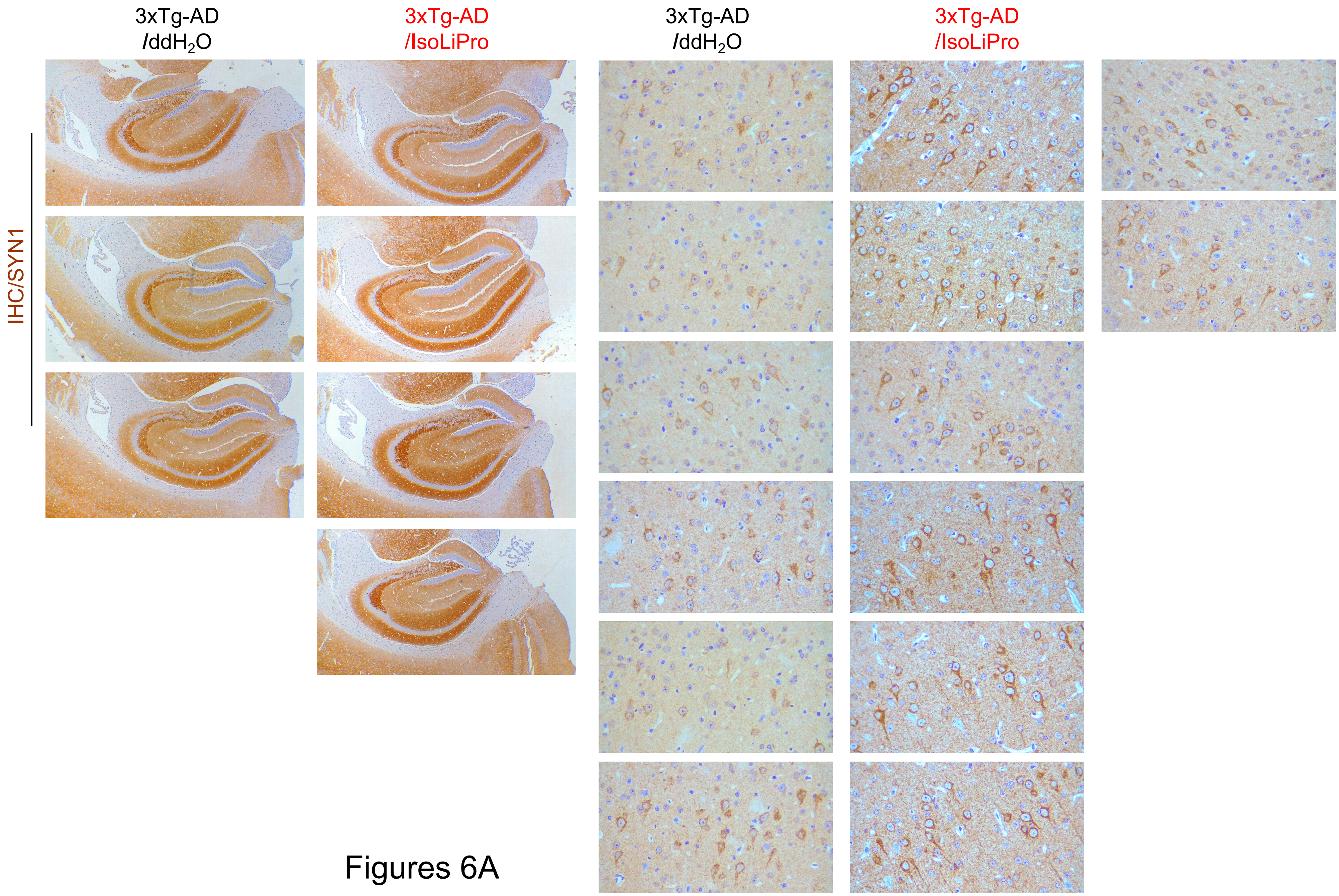
3xTg-AD
/ddH2O
3xTg-AD
/IsoLiPro
3xTg-AD
/ddH2O
3xTg-AD
/IsoLiPro
IHC/SYN1

Figures 6A

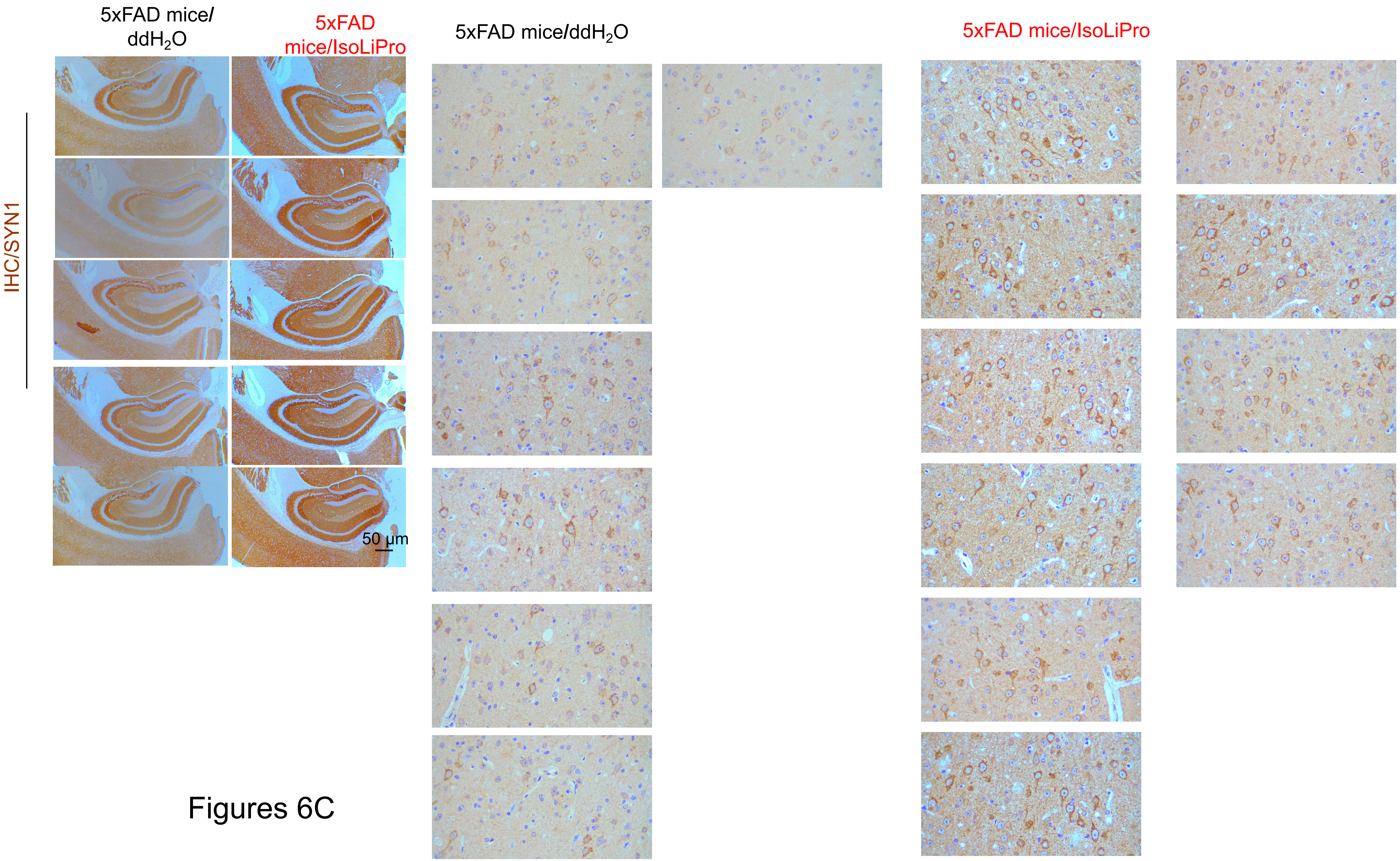
5xFAD mice/ ddH2O
5xFAD mice/IsoLiPro
IHC/SYN1
50 µm
5xFAD mice/ddH2O
5xFAD mice/IsoLiPro

Figures 6C
