# Supplementary figures and images for: Targeting USP11 regulation by a novel lithium-organic coordination compound improves neuropathologies and cognitive functions in Alzheimer transgenic mice (part 2 of 2)
Source: EMBO Mol Med. 2024 Oct 11;16(11):2856–81. doi: 10.1038/s44321-024-00146-7 (PMC11555261; doi:10.1038/s44321-024-00146-7)

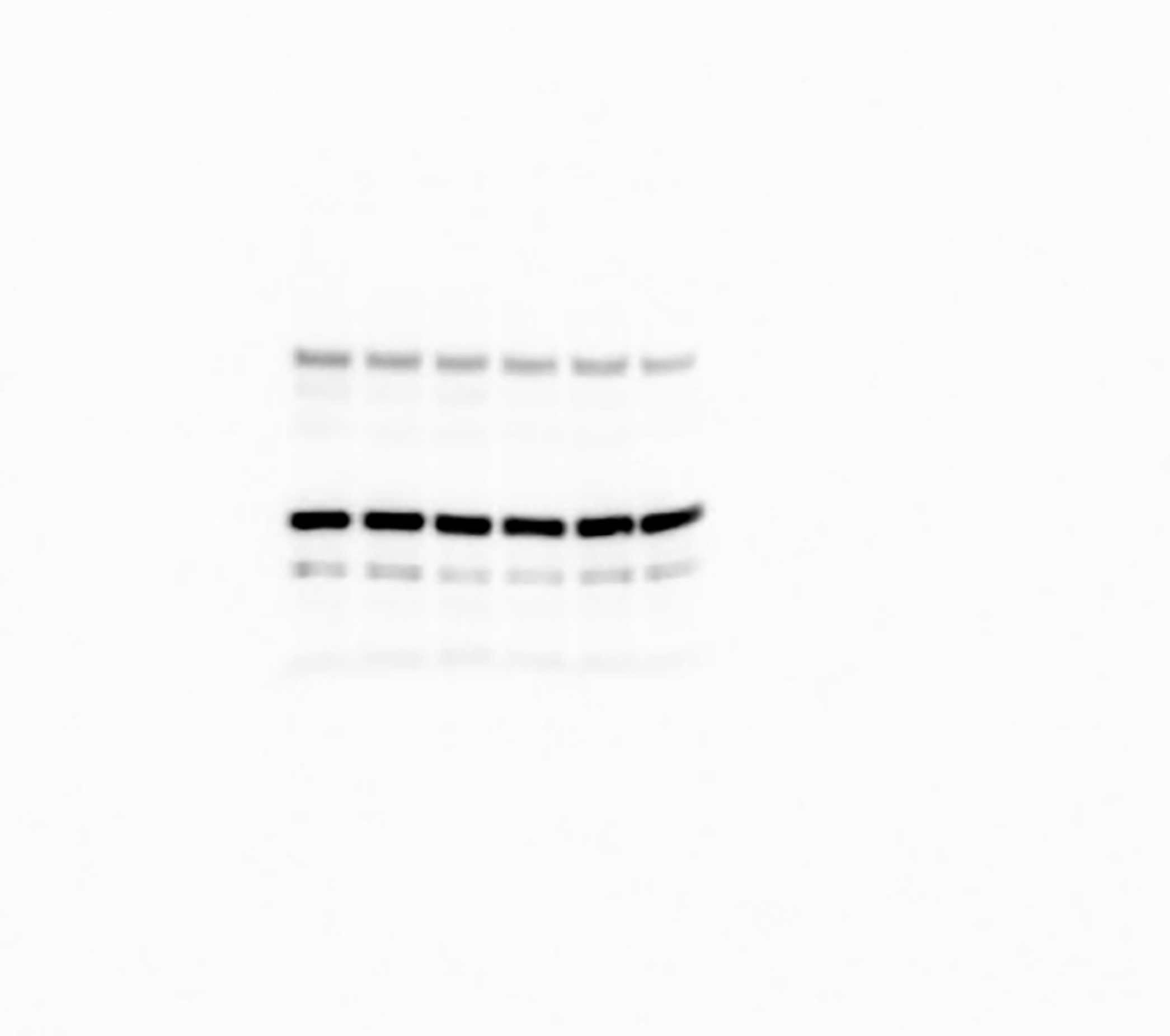

Supplement: Supplementary file 9 — Source data Fig. 6 [file 44321_2024_146_MOESM9_ESM.zip › Source data Fig. 6 (MOESM9)/Fig. 6E/5xFAD-SYN1-GAPDH-insolution.tif]

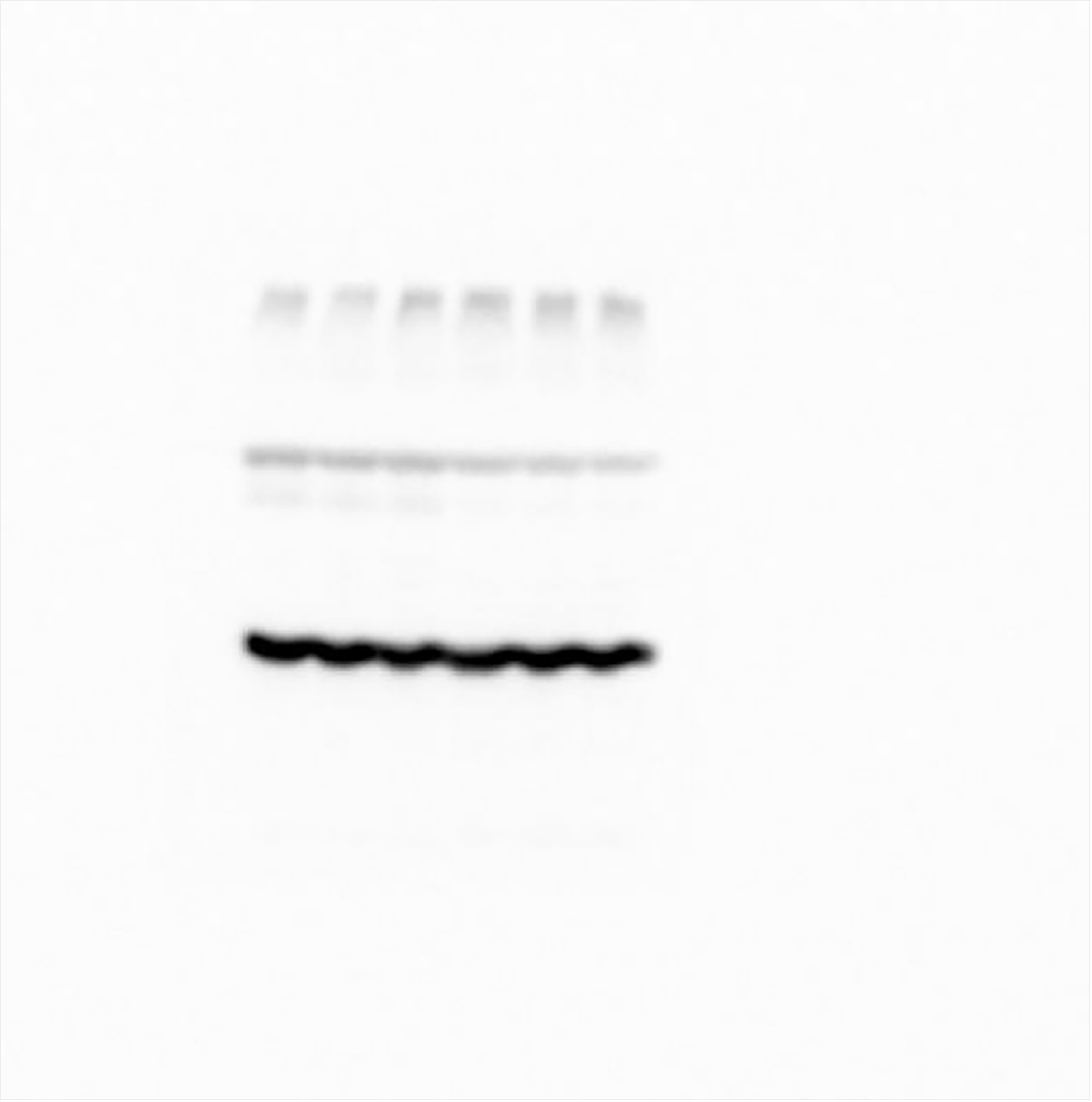

Supplement: Supplementary file 9 — Source data Fig. 6 [file 44321_2024_146_MOESM9_ESM.zip › Source data Fig. 6 (MOESM9)/Fig. 6E/5xFAD-SYN1-GAPDH-solution.tif]

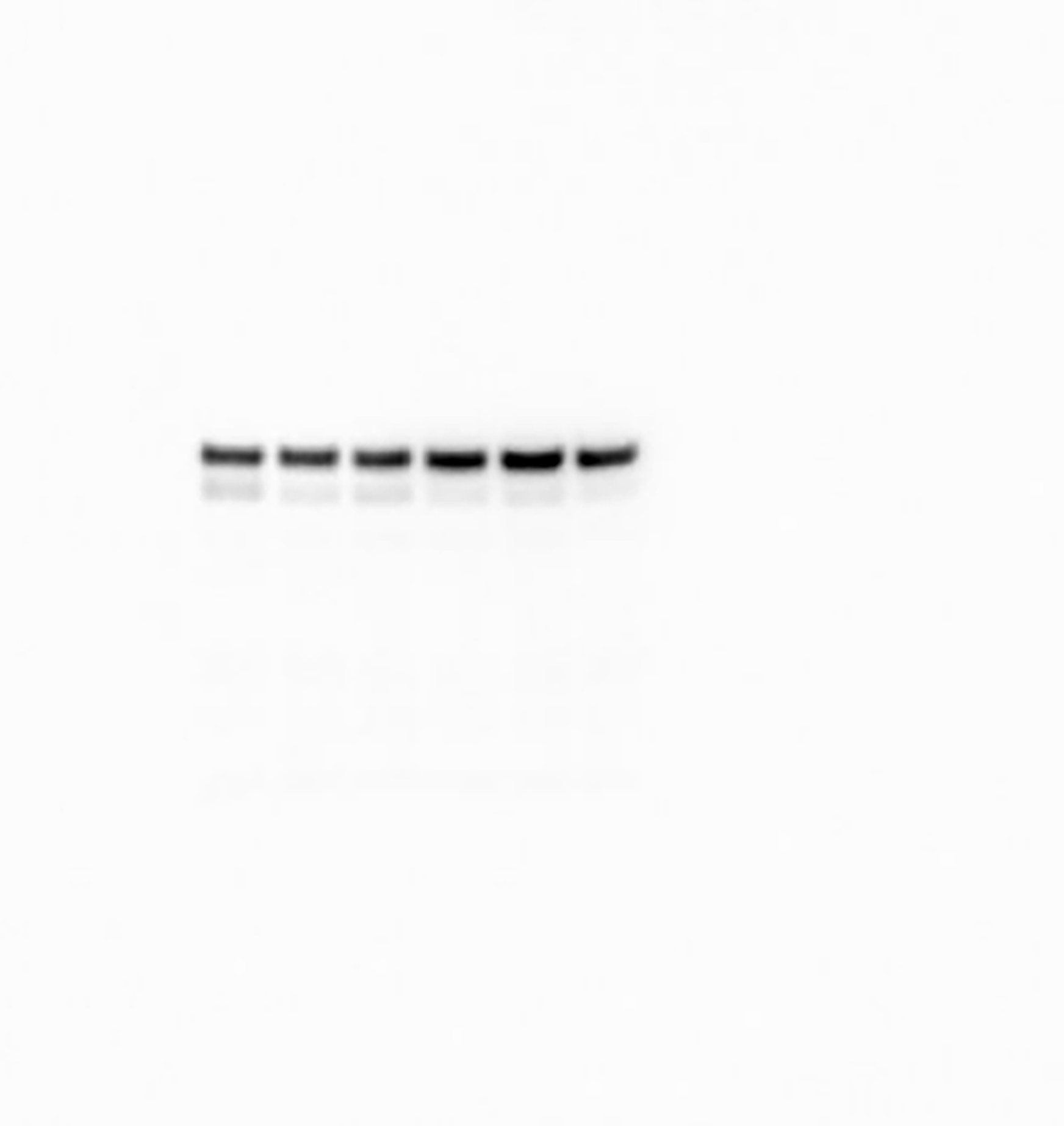

Supplement: Supplementary file 9 — Source data Fig. 6 [file 44321_2024_146_MOESM9_ESM.zip › Source data Fig. 6 (MOESM9)/Fig. 6E/5xFAD-SYN1-insolution.tif]

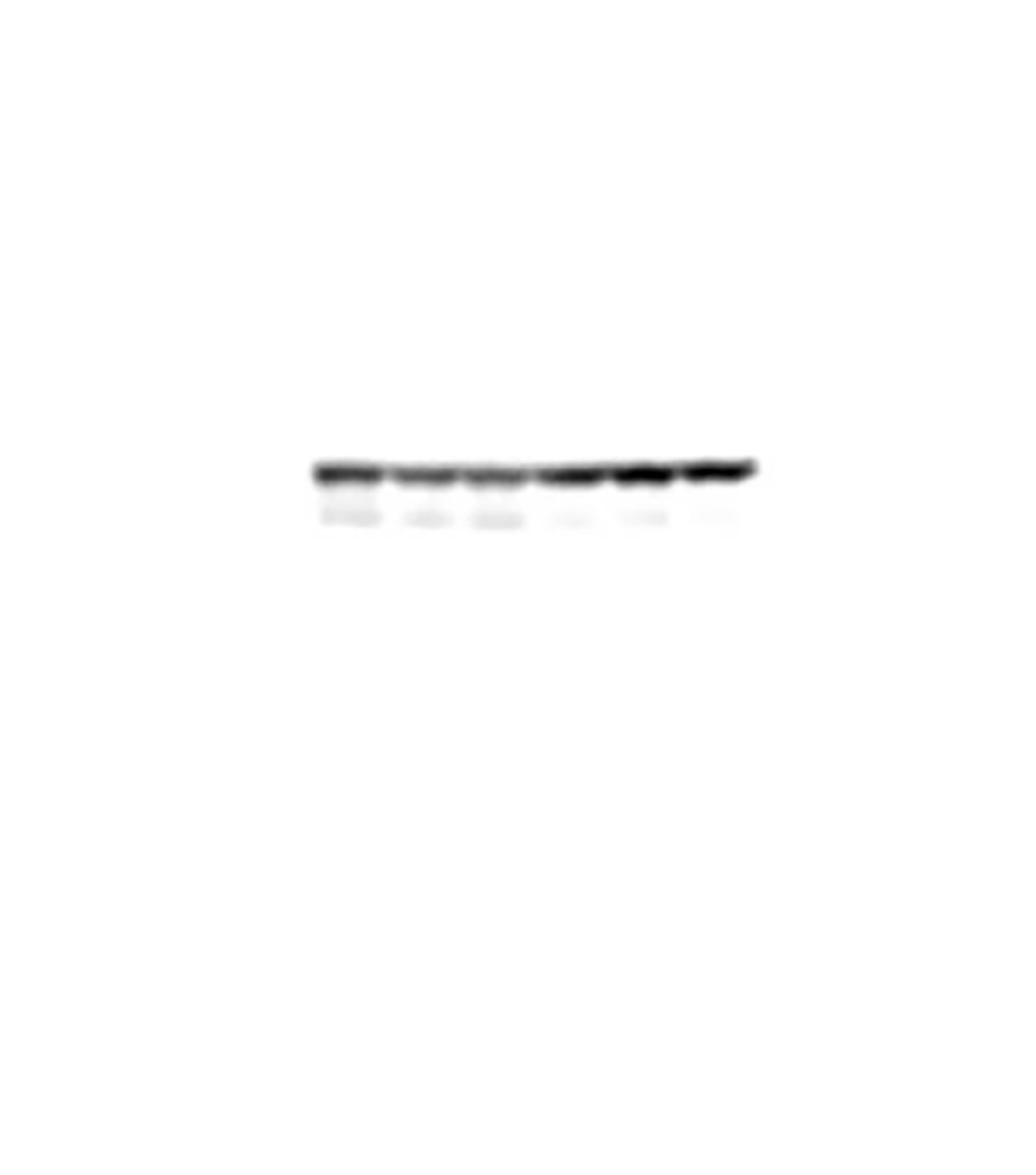

Supplement: Supplementary file 9 — Source data Fig. 6 [file 44321_2024_146_MOESM9_ESM.zip › Source data Fig. 6 (MOESM9)/Fig. 6E/5xFAD-SYN1-solution.tif]

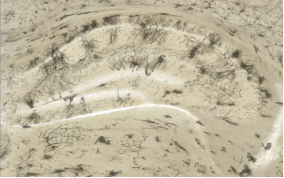

Supplement: Supplementary file 9 — Source data Fig. 6 [file 44321_2024_146_MOESM9_ESM.zip › Source data Fig. 6 (MOESM9)/Fig. 6G/3xTg-AD mice-ddH2O.tif]

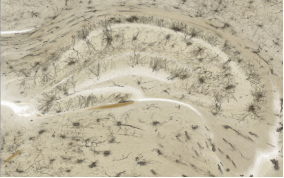

Supplement: Supplementary file 9 — Source data Fig. 6 [file 44321_2024_146_MOESM9_ESM.zip › Source data Fig. 6 (MOESM9)/Fig. 6G/3xTg-AD mice-IsoLiPro.tif]
