# Supplementary material for: Targeting USP11 regulation by a novel lithium-organic coordination compound improves neuropathologies and cognitive functions in Alzheimer transgenic mice
Source: EMBO Mol Med. 2024 Oct 11;16(11):2856–81. doi: 10.1038/s44321-024-00146-7 (PMC11555261; doi:10.1038/s44321-024-00146-7)
Supplement: Supplementary file 13 — Expanded View Figures [file 44321_2024_146_MOESM13_ESM.pdf]

Expanded View Figures

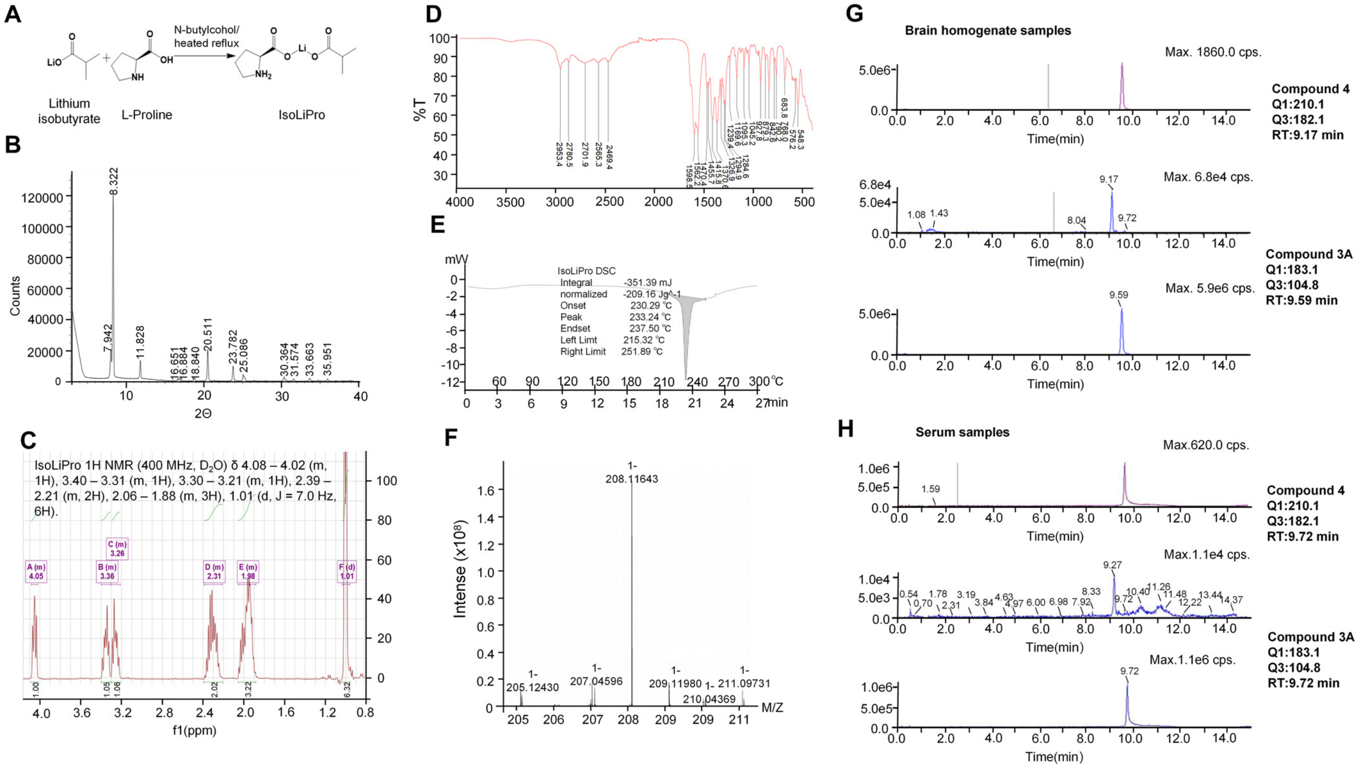

Figure EV1. Synthesis and characterization of IsoLiPro.

(**A**) Reaction diagram - Reaction for IsoLiPro. (**B**) Powder X-ray diffraction analysis of n-butanol as a single solvent in a powder with IsoLiPro. (**C**) ^{1}H NMR spectrum of IsoLiPro in MeOD solvent. (**D**) Spectrum FT-IR of IsoLiPro. (**E**) Differential scanning calorimetry analysis of IsoLiPro. (**F**) Ion mass-to-charge ratios of IsoLiPro molecules tested with MRMS of Bruker solarix in negative ion mode. The detection of IsoLiPro (MW:210.1) whole molecule in brain homogenate (**G**) and serum (**H**) using tandem quadrupole mass spectrometry.

© The Author(s)

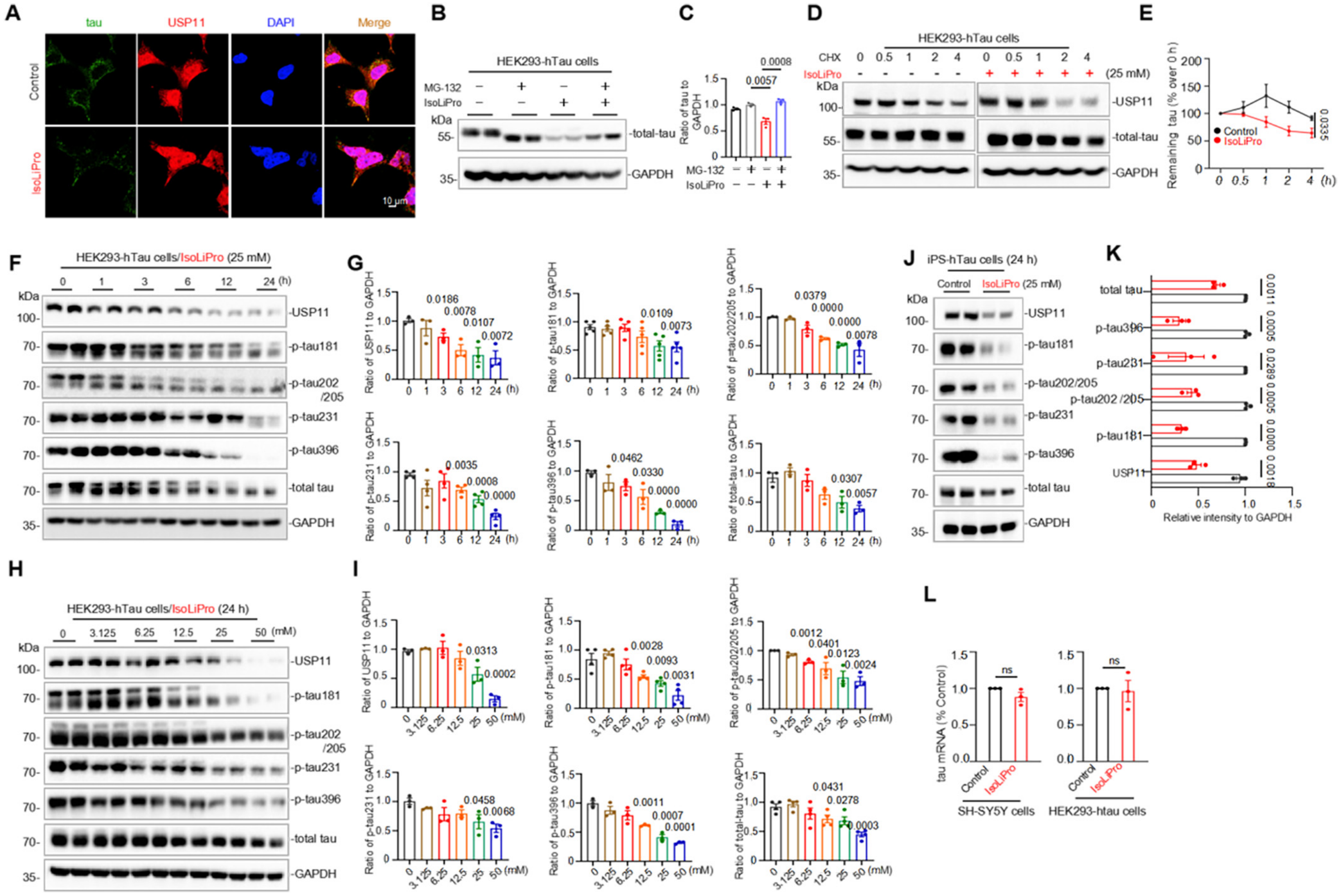

Figure EV2. IsoLiPro markedly reduces levels of total and phosphorylated tau in cultured cells overexpressing wild-type full-length human tau.

(**A**) IsoLiPro significantly reduces the co-localization of USP11 (red) and tau (green) in HEK293-hTau cells measured by immunofluorescence (IF). (**B, C**) IsoLiPro (25 mM for 24 h) markedly accelerates tau clearance, altered by inhibition of the proteasomal- (MG132, 10 μM) degradation signaling pathways in HEK293-hTau cells. Data were represented as means ± SEM ($n = 3$ samples per group). *P* values were calculated using one-way ANOVA followed by Tukey's HSD test. (**D, E**) IsoLiPro markedly enhances tau turnover in HEK293-hTau cells (CHX, 100 μg/mL). Data were represented as means ± SEM ($n = 3$ samples per group). *P* values were calculated using two-way ANOVA, with comparisons made against the Control group. (**F–I**) IsoLiPro markedly reduces phospho- and total-tau levels in a time- (**F, G**) and dose-dependent (**H, I**) manner in HEK293-hTau cells. Data were represented as means ± SEM ($n = 3$ to 5 samples per group). *P* values were calculated using multiple *t*-tests, with comparisons made against the 0 h or 0 mM group. (**J, K**) IsoLiPro significantly inhibits phospho- and total-tau levels in iPS-hTau cells. Data were represented as means ± SEM ($n = 3$ samples per group). *P* values were calculated using a two-tailed *t*-test, with comparisons made against the Control group. (**L**) IsoLiPro markedly lowers total tau protein levels without affecting mRNA levels in SH-SY5Y or HEK293-htau cells. Data were represented as means ± SEM ($n = 3$ samples per group). *P* values were calculated using a two-tailed *t*-test, with comparisons made against the Control group. Source data are available online for this figure.

 © The Author(s)

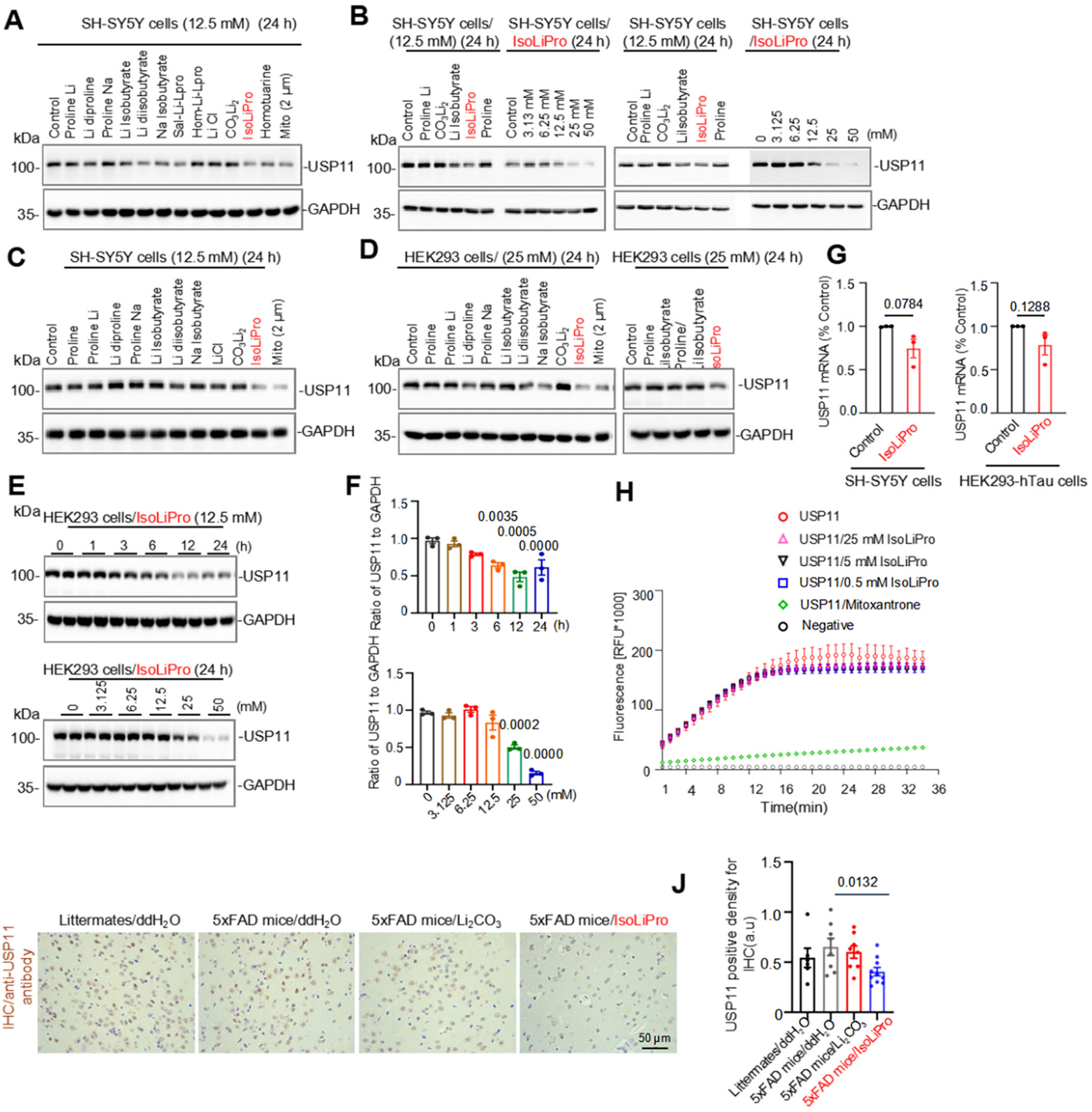

Figure EV3. IsoLiPro markedly decreases USP11 protein levels in SH-SY5Y and HEK293 cells.

(**A–C**) Representative blots depicting the alterations in USP11 protein levels following treatment with diverse small molecule drugs in SH-SY5Y cells. (**D–F**) Representative blots illustrate the changes in USP11 protein levels upon treatment with a range of small molecule drugs in HEK293 cells. Data were represented as means ± SEM ($n = 3$ samples per group). *P* values were calculated using multiple *t*-tests, with comparisons made against the 0 h or 0 mM group. (**G**) USP11 mRNA level alteration after IsoLiPro treatment. Data were represented as means ± SEM ($n = 3$ samples per group). *P* values were calculated using a two-tailed *t*-test, with comparisons made against the Control group. (**H**) Representative Ub-AMC cleavage assay of USP11. The fluorescence signal (RFU*103) is plotted against the time [min]. Data were represented as means ± SEM ($n = 3$ samples per group). (**I**, **J**) Immunohistochemical images revealing USP11 protein levels in the cortex of 5xFAD mice. Data were represented as means ± SEM ($n = 3$ to 5 mice per group, with two cortical sections analyzed from each mouse). *P* values were calculated using multiple *t*-tests, with comparisons made against the 5xFAD mice/ddH$_2$O group. Source data are available online for this figure.

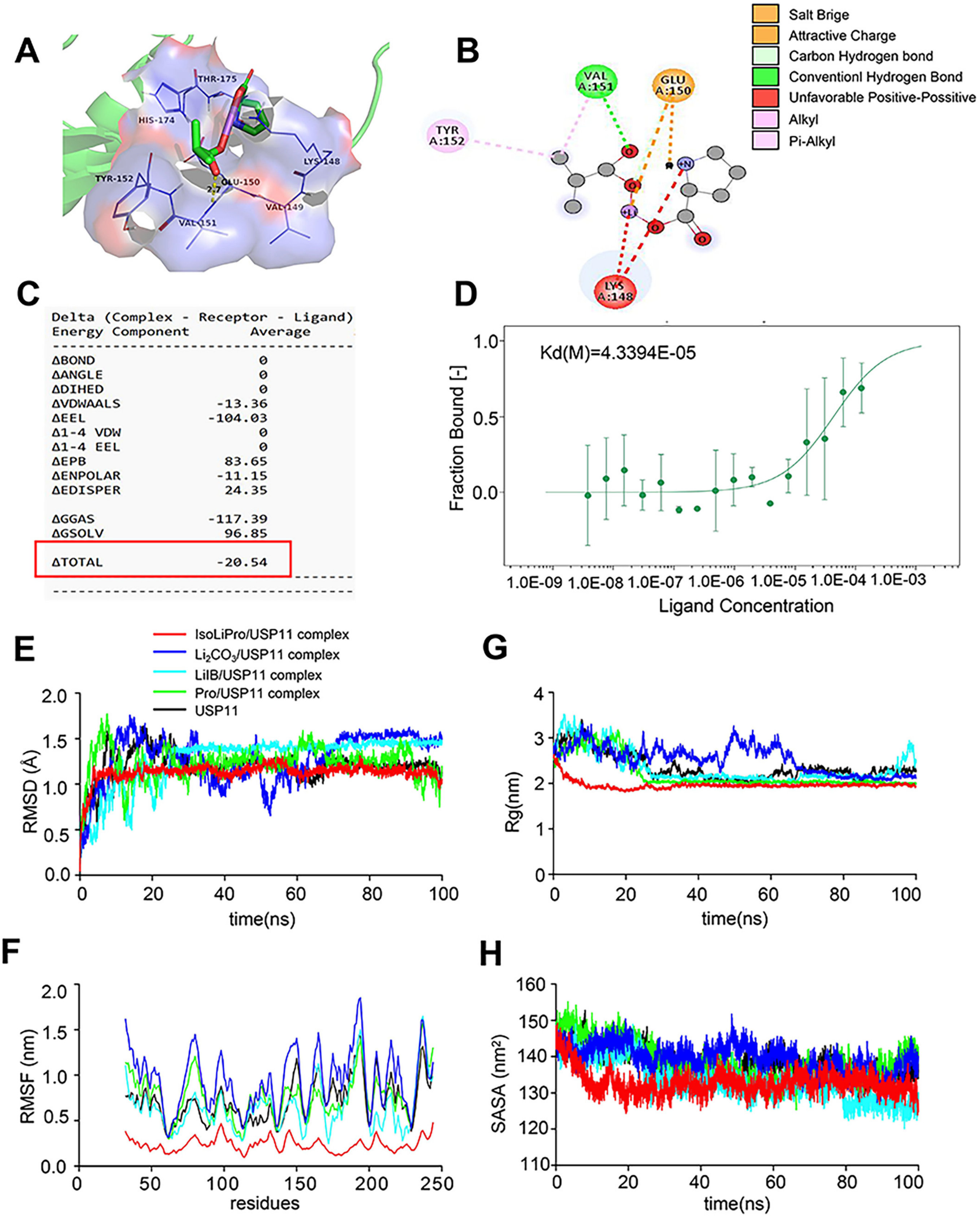
A
THR-175
HIS-174
LYS-148
TYR-152
GLU-150
VAL-149
VAL-151
B
Salt Brige
Attractive Charge
Carbon Hydrogen bond
Conventionl Hydrogen Bond
Unfavorable Positive-Possitive
Alkyl
Pi-Alkyl
VAL A:151
GLU A:150
TYR A:152
LYS A:148
C
Delta (Complex - Receptor - Ligand)
Energy Component Average
ΔBOND 0
ΔANGLE 0
ΔDIHED 0
ΔVDWAALS -13.36
ΔEEL -104.03
Δ1-4 VDW 0
Δ1-4 EEL 0
ΔEPB 83.65
ΔENPOLAR -11.15
ΔEDISPER 24.35
ΔGGAS -117.39
ΔGSOLV 96.85
ΔTOTAL -20.54
D
Kd(M)=4.3394E-05
Fraction Bound [-]
Ligand Concentration
E
IsoLiPro/USP11 complex
Li2CO3/USP11 complex
LiIB/USP11 complex
Pro/USP11 complex
USP11
RMSD (Å)
time(ns)
G
Rg(nm)
time(ns)
F
RMSF (nm)
residues
H
SASA (nm²)
time(ns)

◀ **Figure EV4. Binding affinity of IsoLiPro to USP11.**

(**A**) The 3D image illustrates IsoLiPro's binding to the USP11 protein. (**B**) The 2D image reveals IsoLiPro's interaction with several amino acids, including Lys148, Glu150, Val151, and Tyr152. (**C**) The binding free energy between IsoLiPro and USP11 was calculated using the MM-PBSA method. (**D**) Microscale thermophoresis (MST) results: IsoLiPro binding to USP11 protein yielded a dissociation (Kd) of 4.3394E-05 M. Data were represented as means ± SEM ($n = 3$ samples per group). (**E–H**) Analysis of root mean square deviation (RMSD) (**E**), root mean square fluctuation (RMSF) (**F**), radius of gyration (Rg) (**G**), and solvent accessible surface area (SASA) (**H**) of USP11 and its complexes with IsoLiPro, LiIB, Li_2CO_3, Pro during 100 ns MD simulations. Source data are available online for this figure.

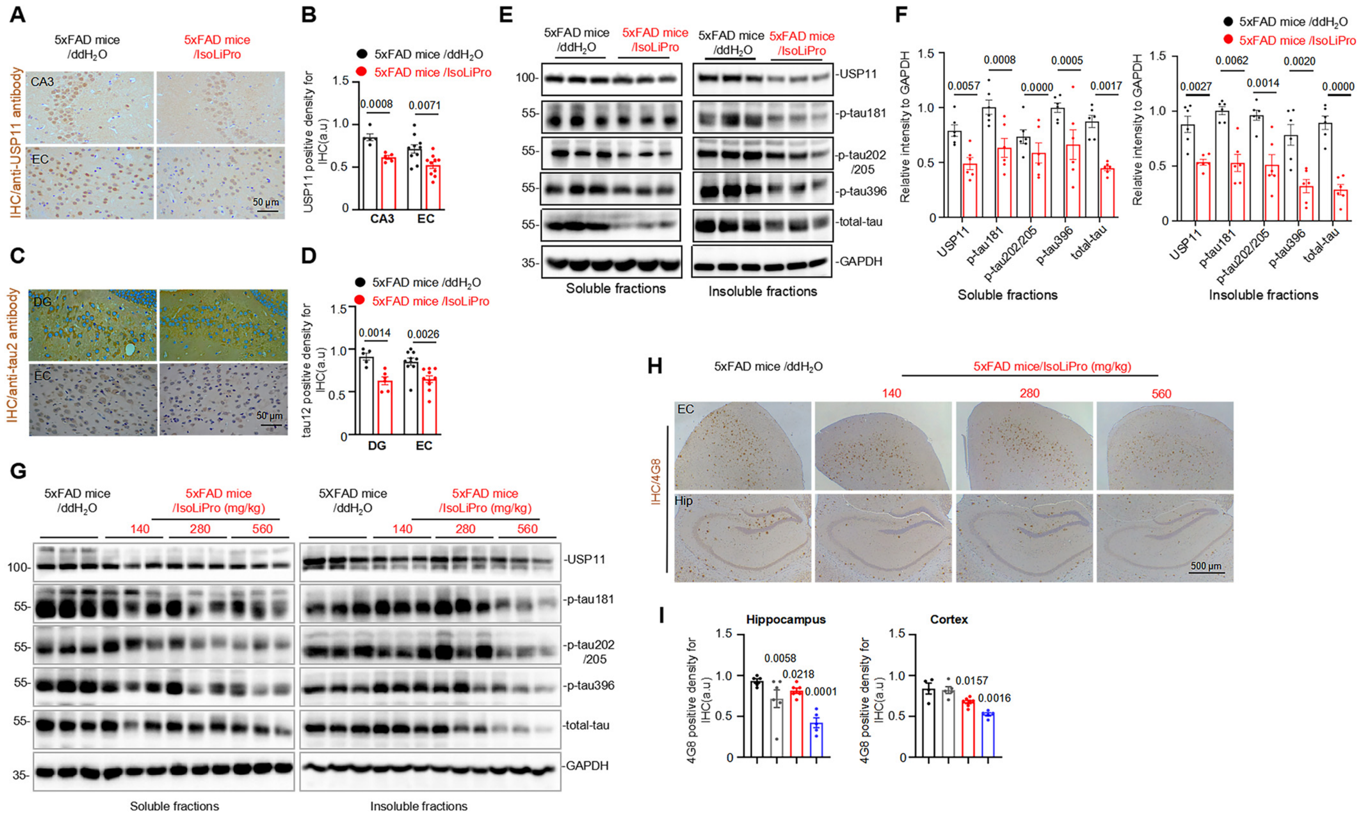

Figure EV5. IsoLiPro markedly induces tau clearance in the brain of 5xFAD mice.

(**A**, **B**) In 10-week-old 5xFAD mice, a 12-week oral administration of IsoLiPro (560 mg/kg) significantly decreased USP11 levels in both the hippocampus and cortex, as assessed by IHC. Data were represented as means ± SEM ($n = 5$ mice per group, with two cortical sections analyzed from each mouse). P values were calculated using a two-tailed t-test, with comparisons made against the 5xFAD mice/ddH$_2$O group. (**C**, **D**) Oral gavage IsoLiPro notably decreased the levels of total-tau in the hippocampus and cortex of the 5xFAD mice measured by IHC. Data were represented as means ± SEM ($n = 5$ mice per group, with two cortical sections analyzed from each mouse). P values were calculated using a two-tailed t-test, with comparisons made against the 5xFAD mice/ddH$_2$O group. (**E**, **F**) Oral gavage IsoLiPro significantly decreased total tau and the tau phosphorylated at multiple-doses AD-related sites (soluble and insoluble fractions) measured by Western blotting. Data were represented as means ± SEM ($n = 6$ mice per group). P values were calculated using a two-tailed t-test, with comparisons made against the 5xFAD mice/ddH$_2$O group. (**G**) Western blot analysis was utilized to validate the effect of orally administered IsoLiPro at varying concentrations on the expression levels of total tau and the tau phosphorylated at multiple-doses AD-related sites (soluble and insoluble fractions). (**H**, **I**) Representative IHC images illustrate alterations in 4G8-positive amyloid plaques in 5xFAD mice subsequent to treatment with different concentrations of IsoLiPro. Data are represented as means ± SEM ($n = 3$ to 5 mice per group). Some samples from the 5xFAD mice/ddH2O and 5xFAD mice/560 mg/kg groups were re-used from Fig. 5E. P values were calculated using multiple t-tests, with comparisons made against the 5xFAD mice/ddH$_2$O. Source data are available online for this figure.

 © The Author(s)
