# Supplementary material for: Targeting USP11 regulation by a novel lithium-organic coordination compound improves neuropathologies and cognitive functions in Alzheimer transgenic mice
Source: EMBO Mol Med. 2024 Oct 11;16(11):2856–81. doi: 10.1038/s44321-024-00146-7 (PMC11555261; doi:10.1038/s44321-024-00146-7)
Supplement: Supplementary file 6 — Source data Fig. 4A,C,G [file 44321_2024_146_MOESM6_ESM.zip › Fig. 4G.zip!/Fig. 4G/Fig. 4.pdf]

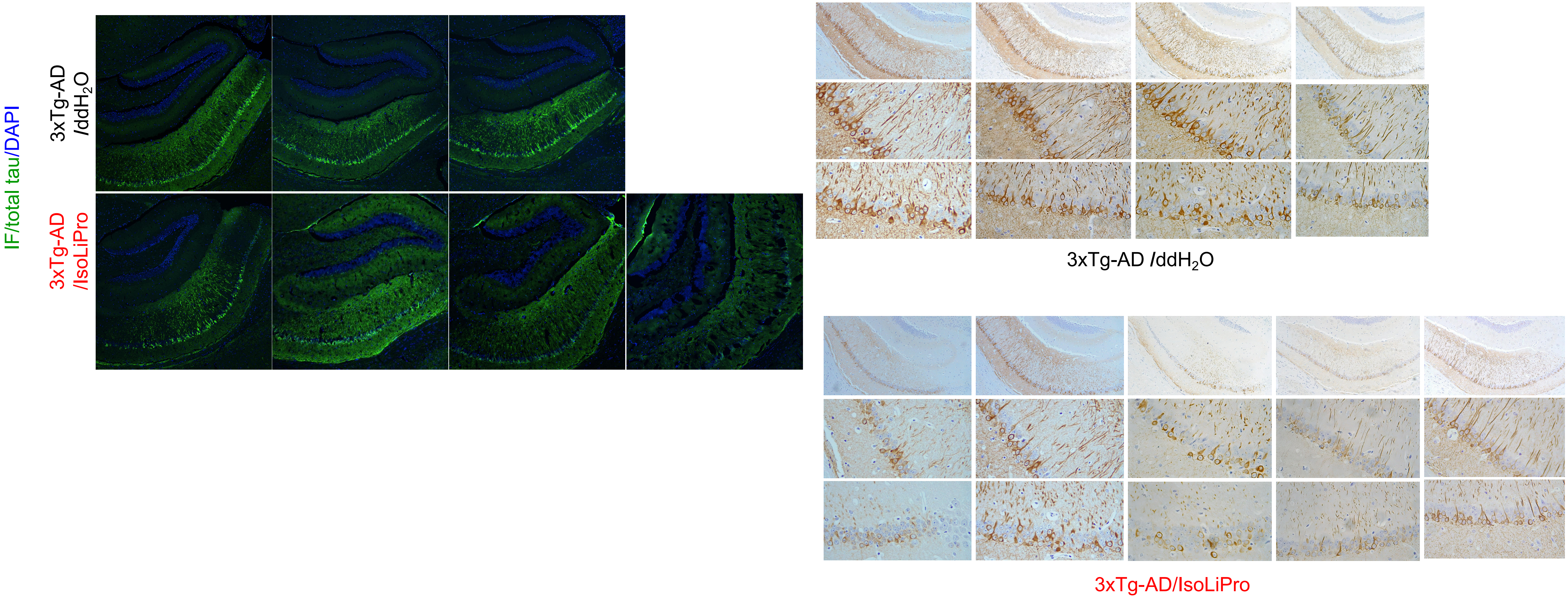
IF/total tau/DAPI
3xTg-AD /ddH$_2$O
3xTg-AD /IsoLiPro
3xTg-AD /ddH$_2$O
3xTg-AD/IsoLiPro

Figure. 4A

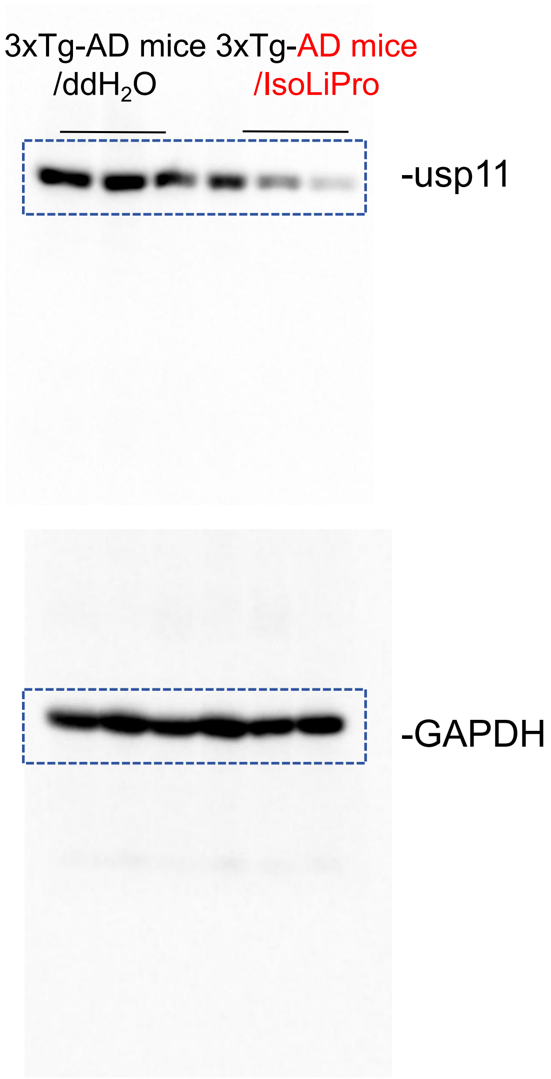

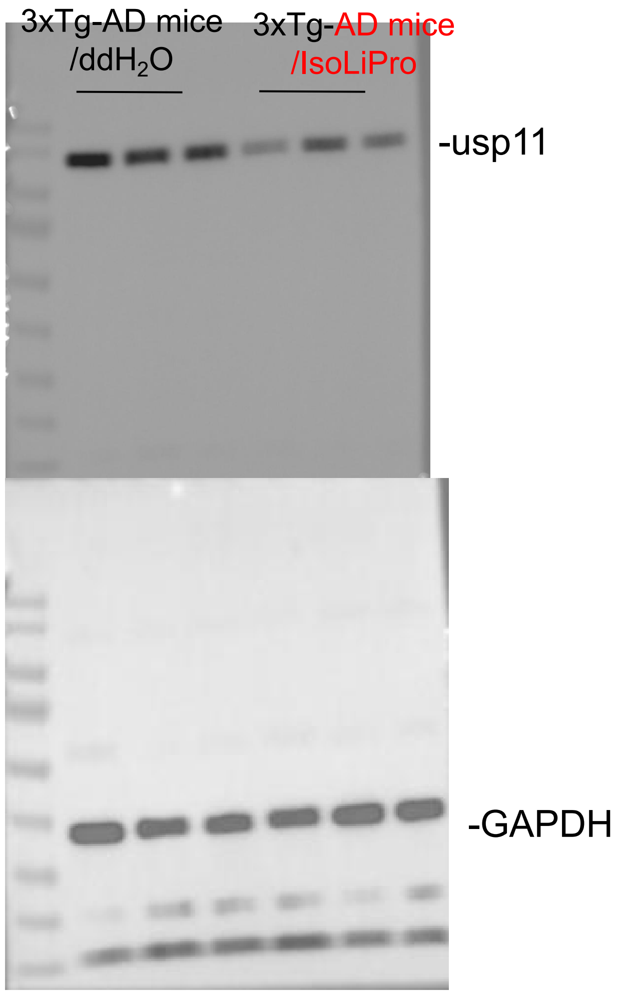

Full unedited gel for Fig. 4G

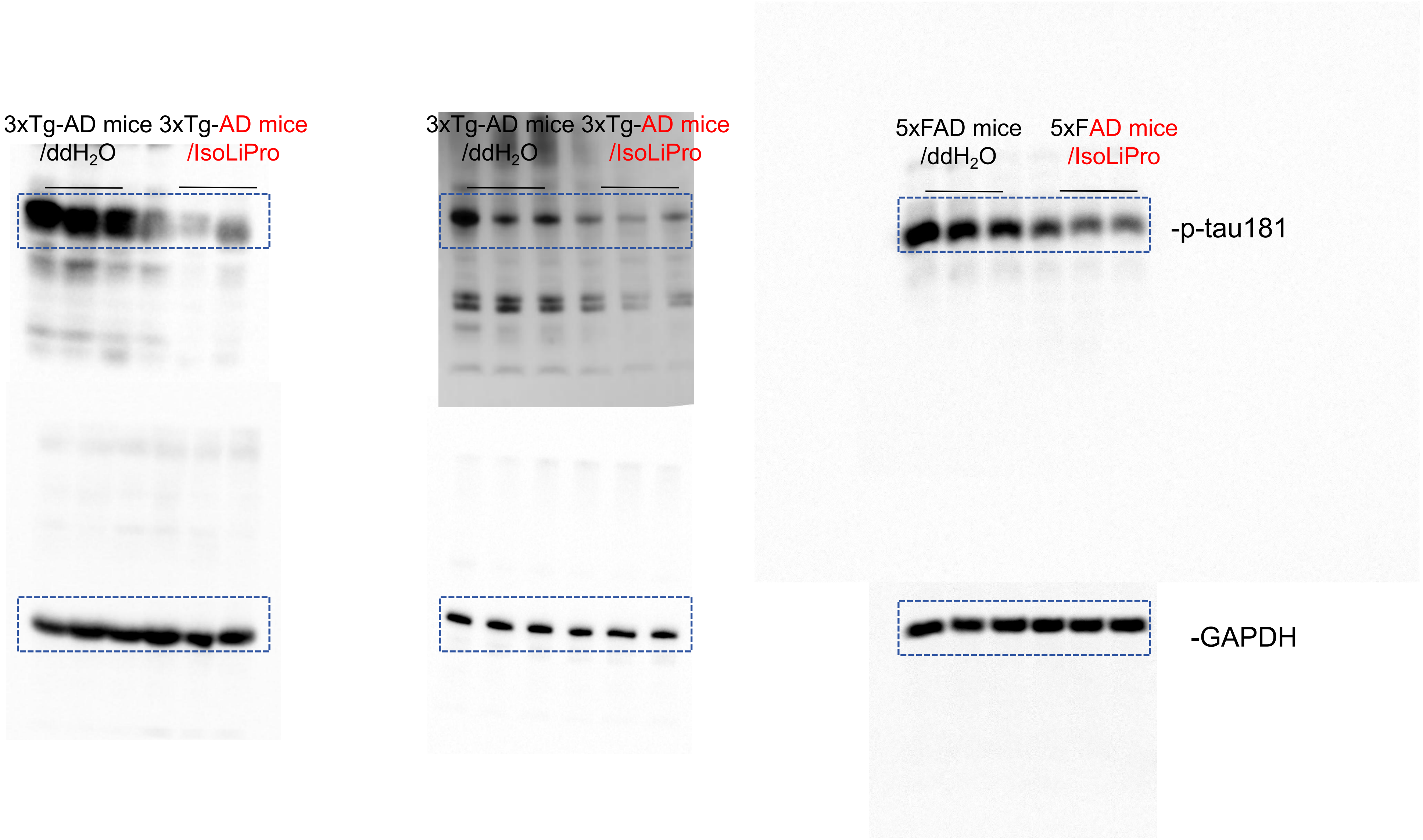

Full unedited gel for Fig. 4G

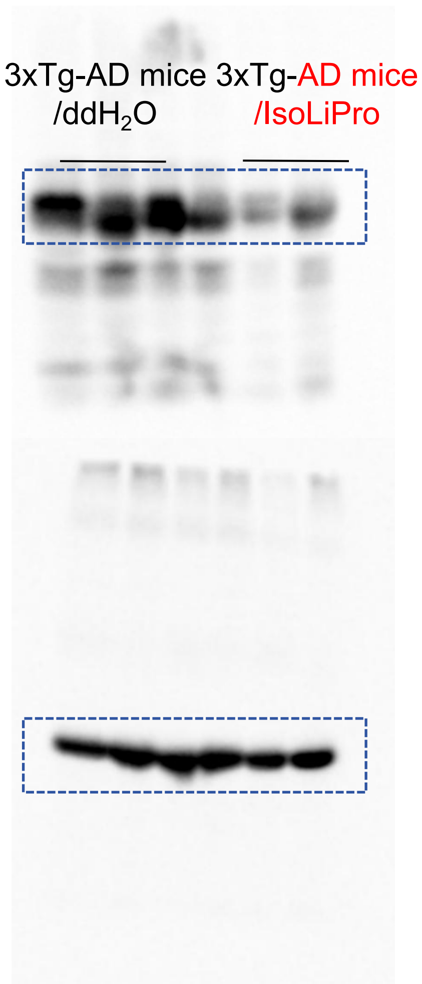

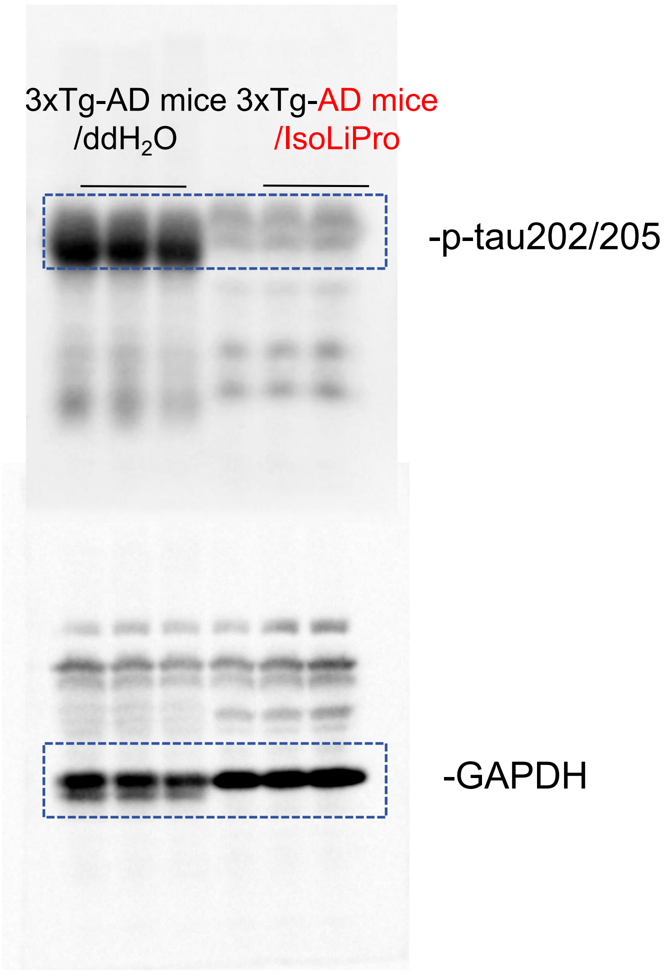

Full unedited gel for Fig. 4G

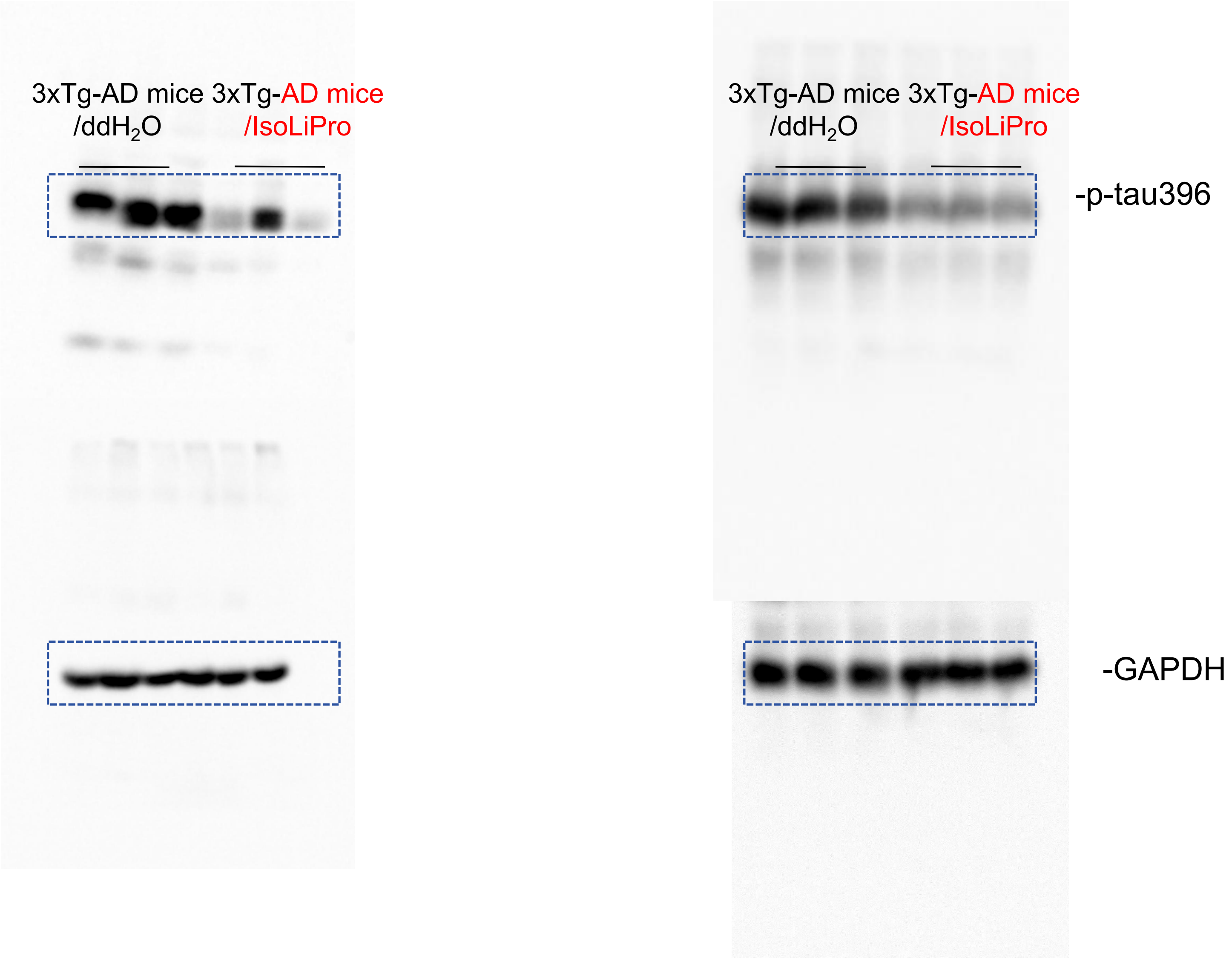

Full unedited gel for Fig. 4G

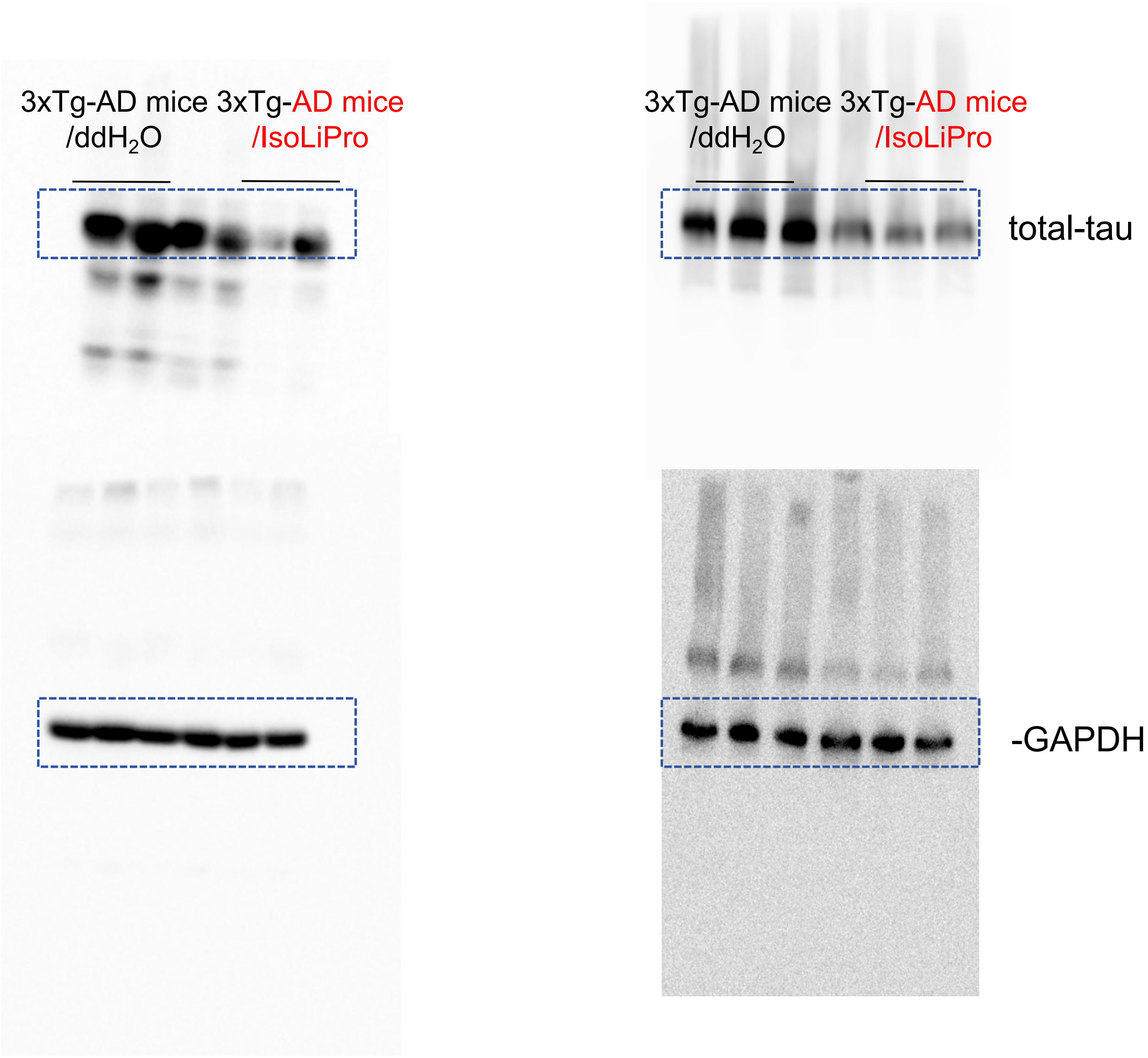

Full unedited gel for Fig. 4G

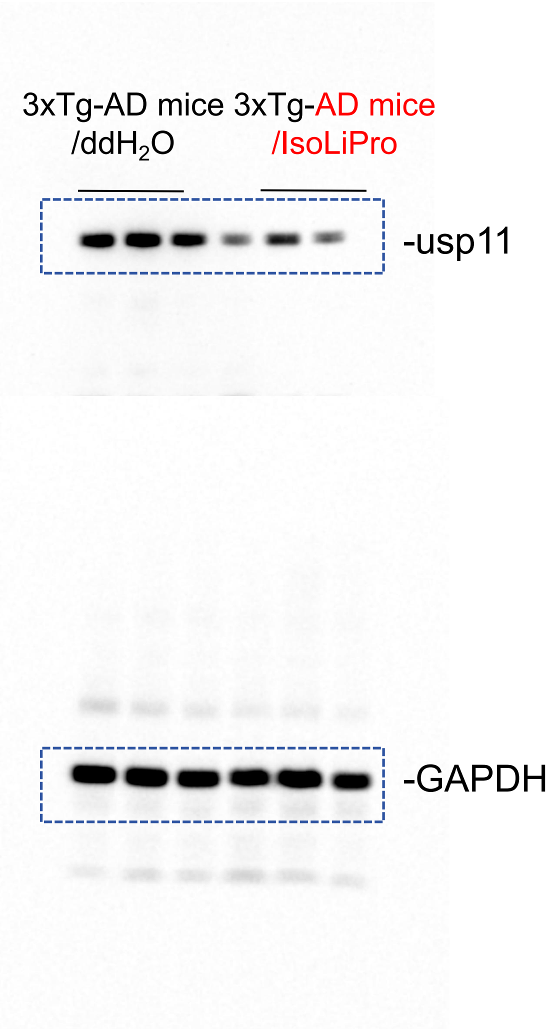

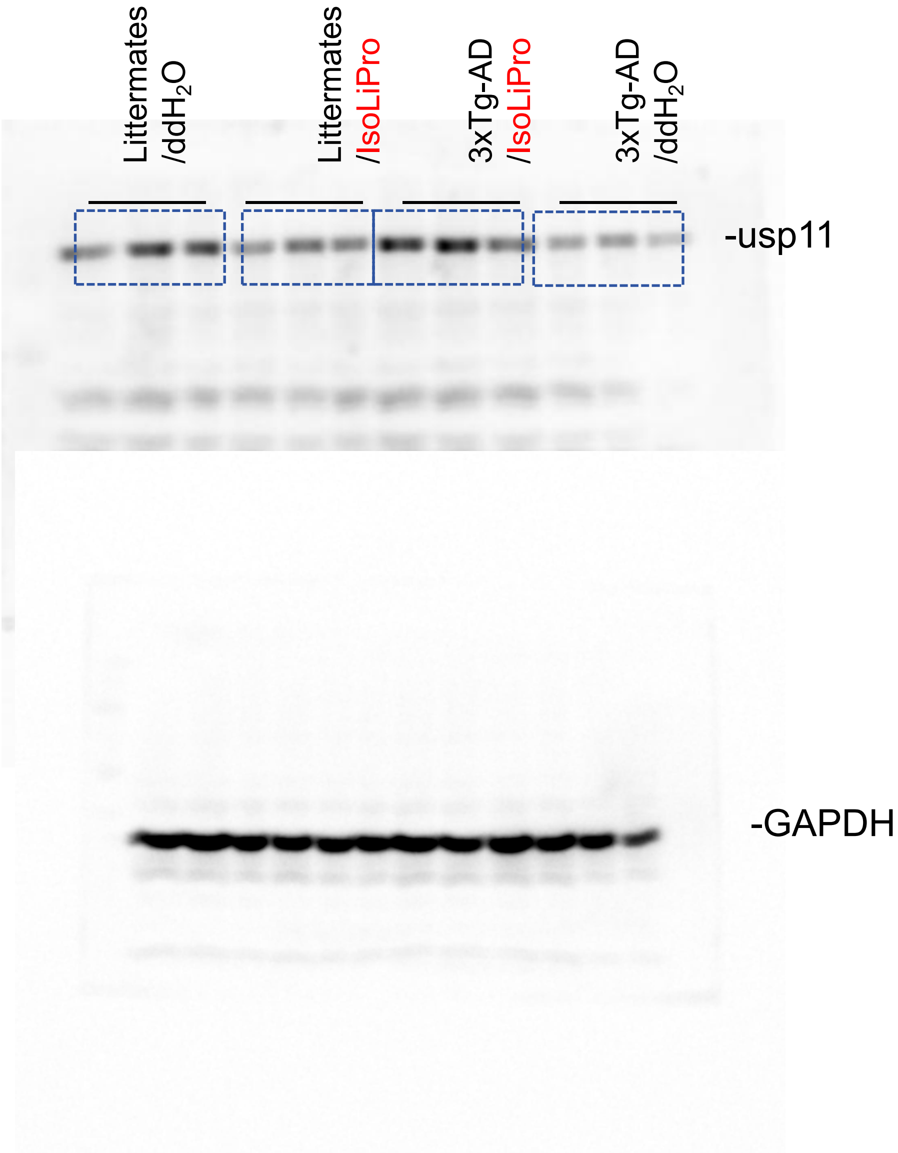

Full unedited gel for Fig. 4G

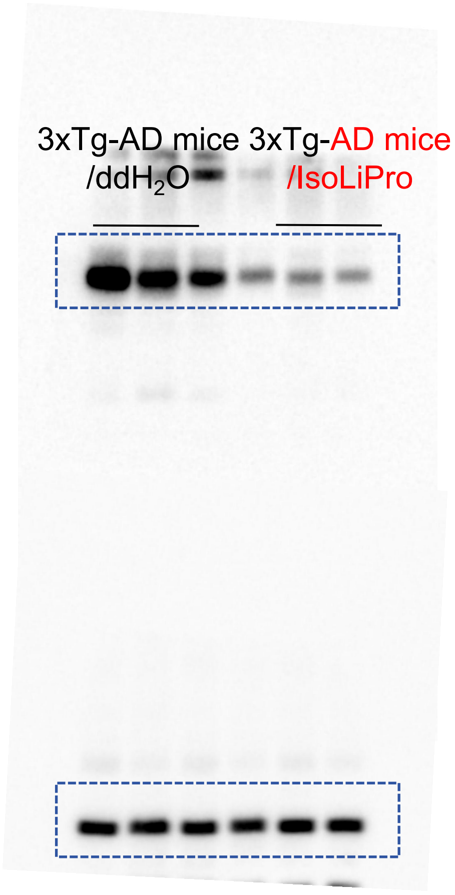

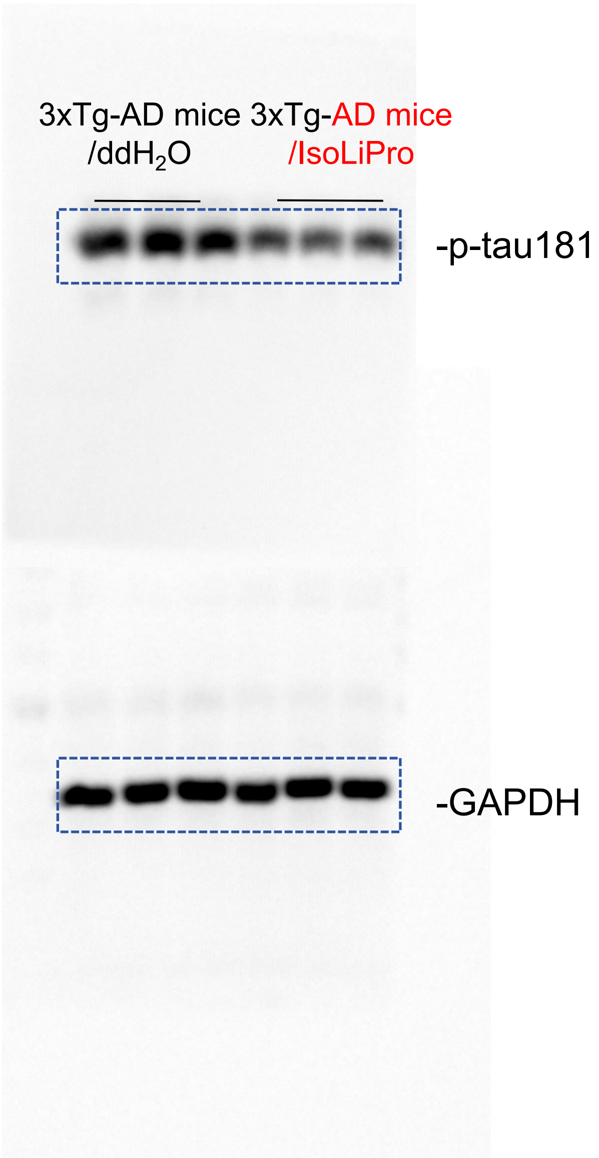

Full unedited gel for Fig. 4G

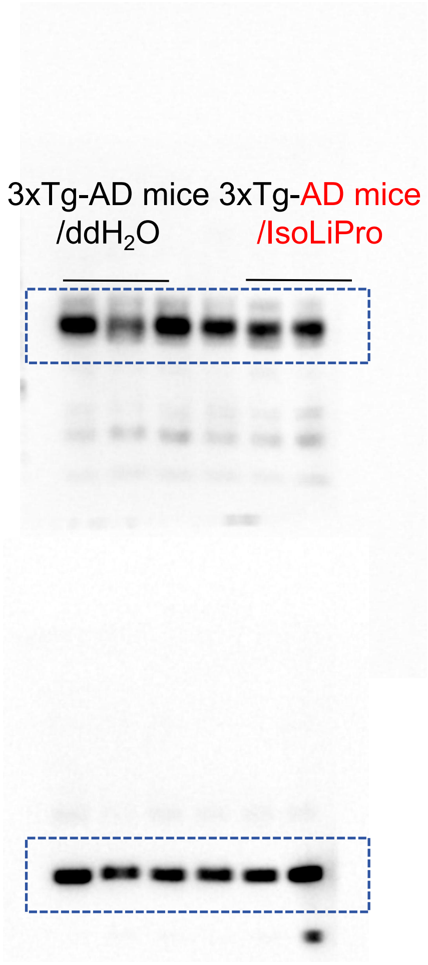

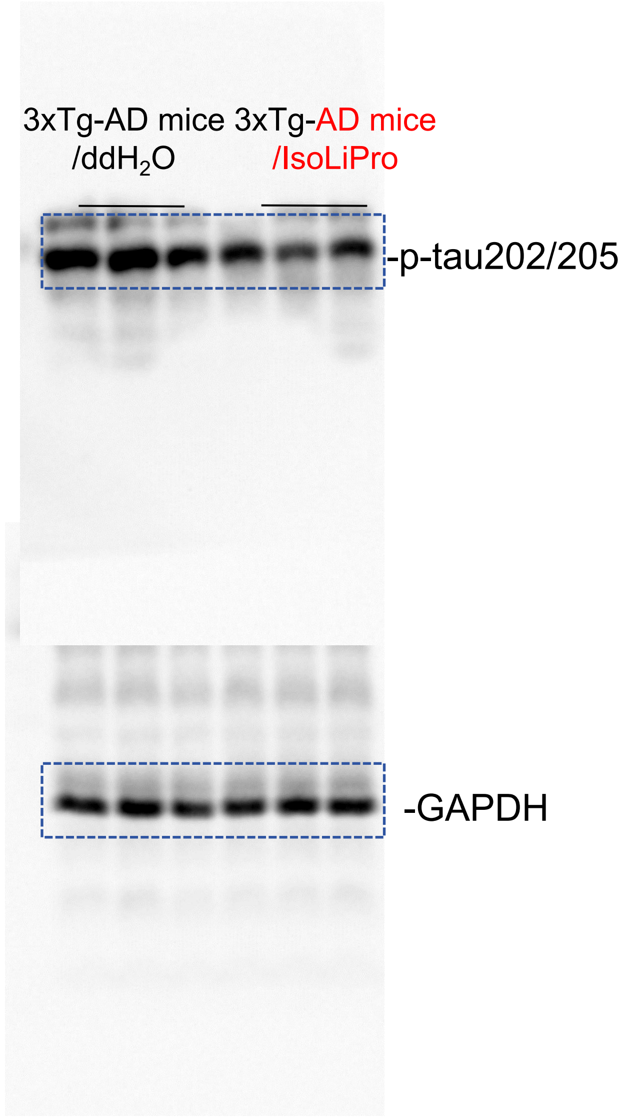

Full unedited gel for Fig.4G

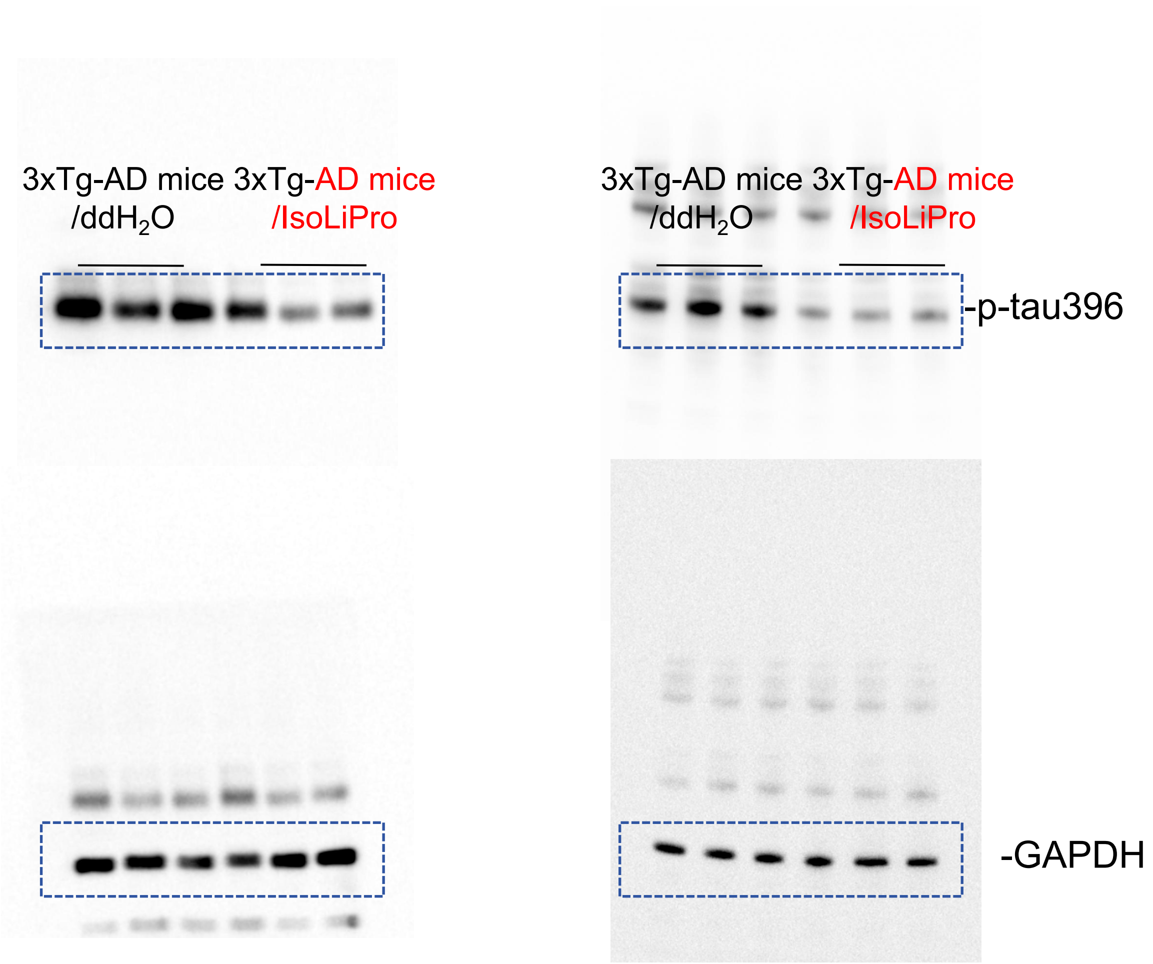

Full unedited gel for Fig. 4G

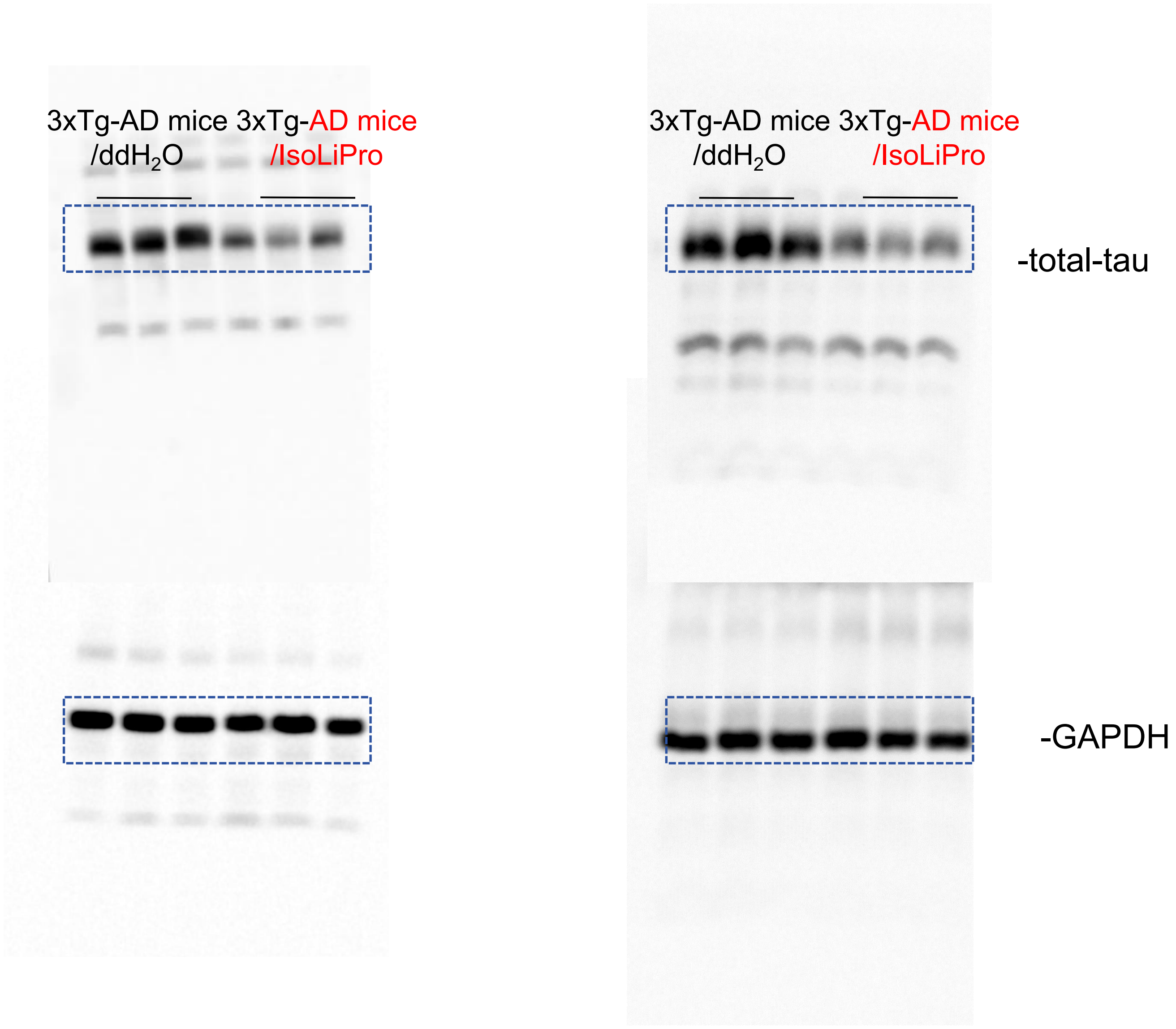

Full unedited gel for Fig. 4G
